# Metagenomics detection and characterisation of viruses in faecal samples from Australian wild birds

**DOI:** 10.1038/s41598-018-26851-1

**Published:** 2018-06-06

**Authors:** Jessy Vibin, Anthony Chamings, Fiona Collier, Marcel Klaassen, Tiffanie M. Nelson, Soren Alexandersen

**Affiliations:** 1 Geelong Centre for Emerging Infectious Diseases, Geelong, Victoria, 3220 Australia; 2 Deakin University, Geelong, Victoria, 3220 Australia; 3 Barwon Health, University Hospital Geelong, Geelong, Victoria 3220 Australia

## Abstract

We present an optimised metagenomics method for detection and characterisation of all virus types including single and double stranded DNA/RNA and enveloped and non-enveloped viruses. Initial evaluation included both spiked and non-spiked bird faecal samples as well as non-spiked human faecal samples. From the non-spiked bird samples (Australian Muscovy duck and Pacific black ducks) we detected 21 viruses, and we also present a summary of a few viruses detected in human faecal samples. We then present a detailed analysis of selected virus sequences in the avian samples that were somewhat similar to known viruses, and had good quality (Q20 or higher) and quantity of next-generation sequencing reads, and was of interest from a virological point of view, for example, avian coronavirus and avian paramyxovirus 6. Some of these viruses were closely related to known viruses while others were more distantly related with 70% or less identity to currently known/sequenced viruses. Besides detecting viruses, the technique also allowed the characterisation of host mitochondrial DNA present and thus identifying host species, while ribosomal RNA sequences provided insight into the “ribosomal activity microbiome”; of gut parasites; and of food eaten such as plants or insects, which we correlated to non-avian host associated viruses.

## Introduction

Detection and characterisation of viruses by metagenomics (VM) is a relatively new technique that takes advantage of the sensitivity of next-generation sequencing (NGS) while being broadly non-specific for any particular virus present in a given sample. Virus communities (viromes) are structurally and functionally diverse and distinct to habitats in hosts and environments, and prior to VM there was limited understanding of the virome present in human and non-human hosts. Now with the growing use of VM, information on eukaryotic viruses, prokaryotic viruses and even viruses that infect other viruses are increasing^1–5^. The objectives of the physical preparation of samples for metagenomic sequencing of viruses are (i) to eliminate as many nucleic acids from the host and other elements like bacteria, fungi, parasites and, (ii) to ensure that as much of the virus nucleic acids are retained during the entire process and (iii) to generate good quality NGS reads.

Different approaches have been applied based on, for example, the sample type and the sequencing platforms used^1,6–10^. Among such proposed protocols, one designated NetoVIR by Conceição-Neto, N *et al*.^6^ was optimised on mock communities and later tested on actual and complex biological samples by the same research group^11–13^. The latter testing was performed without spiking the biological samples with known viruses, which would have served as a useful positive control to validate the protocol. As the overall aim of our project was to detect and characterise viruses in faecal samples from wild birds in Australia, we initially evaluated and compared the existing protocol (NetoVIR), including some additional modifications, and determined the optimum procedure for robust virus detection and characterisation in spiked and non-spiked bird faecal samples. Furthermore, we adopted the protocol downstream of the nucleic acid purification for library preparation and sequencing on the Ion Torrent platform. Using the optimised method, we identified viruses present in selected faecal samples from a Muscovy duck (designated as MUD) and a group of Pacific black ducks (designated as MAD) and from selected human faecal samples to show the broad applicability of the method. Finally, we demonstrate that additional information can be extracted from the sequencing reads, e.g. derived from mitochondrial DNA and ribosomal RNA present in the samples, allowing identification of host species, parasites present, food eaten and faecal bacterial populations present.

## Results

### Method Optimisation

#### Real-time polymerase chain reaction (PCR) assays of marker viruses

Semi-quantitative assays for all marker viruses (see Table S1 of Supplementary material 1 for details) used for validation of the enrichment techniques were optimised using the primers mentioned in Table S2 of Supplementary material 1.

#### Real-time PCRs of marker viruses during method optimisation

**Figure 1:**
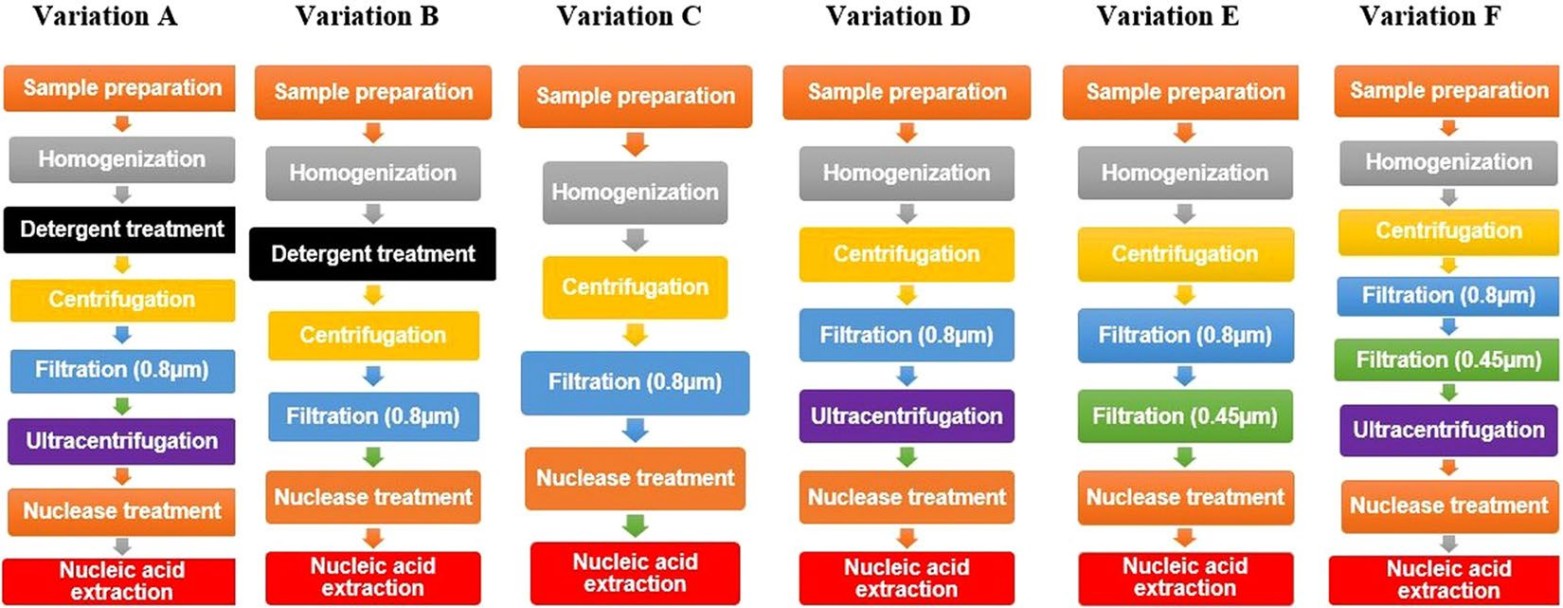
Method variations with a different combination of virus enrichment techniques carried out. The figure gives the flowchart of the different virus enrichment techniques used among the six variations A to F for enriching the faecal sample with virus particles. The sample preparation, homogenisation, centrifugation and filtration using 0.8 µm PES filter to remove larger particles, nuclease treatment and finally nucleic acid extraction remained constant in each variation. However, detergent treatment, ultracentrifugation and filtration using 0.45 µm filter were tried in different combinations to identify the optimal method for virus detection from faecal samples.

The spiked MUD faecal sample was subjected to all the enrichment variations (see Fig. 1 for details), and nucleic acids extracted, and real-time PCR assays carried out to determine which method retained the maximum number and amount of the marker viruses (Table S3 of Supplementary material 1). Variations A and B were discontinued as infectious bronchitis virus (IBV, a relatively large enveloped virus with no inner capsid and the RNA genome only protected by a so-called nucleoprotein^14^) was not retained during the virus enrichment process.

#### Next-generation sequencing (NGS)

The spiked MUD sample was taken through all the steps to NGS for variations C, D, E and F. Variation D from the spiked sample provided the highest number of quality NGS sequence reads for the marker viruses (Table S4 of Supplementary material 1) with a minimum mapping quality threshold of 20 (Q20). Methods C, E and F performed similarly, with method C slightly outperforming methods E and F. Variation D was the method of choice for subsequent virus detection and characterisation from bird faecal samples. However, variation C (similar to variation D but without ultracentrifugation) was also carried out for these samples for comparison and further validation of the method.

### Detection of viruses in bird faecal samples

We identified 9 and 12 viruses from the MUD and MAD faecal samples respectively [Tables 1 and 2]. Both single and double stranded DNA and RNA viruses, as well as enveloped and non-enveloped viruses were identified. As mentioned above we carried out both variation C and D for the faecal samples. When the sample was partitioned for variation C and D before homogenization as we did during the optimisation, in contrast to after method optimisation and selection, variation C provided the most quality virus reads for some viruses (data not shown). However, on subsequent repeats, when the sample was divided after proper homogenization, variation D proved to be the best method for virus enrichment, detection and characterisation suggesting that proper homogenisation of samples, as included as part of the final optimised protocol for variations C and D, is important to ensure a homogeneous distribution of virus particles in the sample before further processing.

**Table 1:** Viruses detected in the Muscovy duck (MUD) faecal sample.

Virus	Family	Characteristics	NCBI virus reference sequence used for mapping	Percentage of identity to the closest virus for individual NGS reads	Likely source
Ngewotan virus (Nam Dinh like virus)	*Nidovirales; Mesoniviridae; Alphamesonivirus*	Monopartite, linear ssRNA (+) genome; enveloped, spherical, about 60–80 nm in diameter.	MF176279	96–100%	Mosquitoes
Hubei chryso- like virus 1	unclassified RNA viruses; *Chrysoviridae* related	dsRNA genome; 4 segments	MF176261-MF176264	99–100%	Mosquitoes
Culex Negev-like virus 3 (Biggie/Goutanap virus like)	unclassified RNA viruses; Negev virus related	ssRNA positive-strand genome	MF176277	92–99%	Mosquitoes
Virus related to Hubei reo-like virus 7	unclassified RNA viruses	dsRNA genome	KX884635	88–95%	Mosquitoes
Israeli acute paralysis like virus	*Dicistroviridae; Aparavirus*	Monopartite, linear ssRNA(+) genome; non enveloped, icosahedral capsid, about 30 nm in diameter	NC009025	98–99%	Bees
Virus related to invertebrate iridescent virus 30	*Iridoviridae; Iridovirus*	Linear, dsDNA genome, polyhedral virions, capsid present, envelope may or may not be present	NC023611	77–92%	Moths
black grass cryptic like virus 2 like virus	*Partitiviridae;* unclassified RNA viruses	Segmented dsRNA genome other details unknown	NC026799	76–98%	Plants
Virus related to Hordeum vulgare endornavirus	*Endornaviridae; Alphaendornavirus*	Linear dsRNA genome, No true capsid.	NC028949	82%	Plants
Enterobacteria phage phi 92	*Myoviridae*	Linear, dsDNA genome; Non-enveloped, head-tail structure	FR775895	97–99%	Bacteria

This table displays the viruses detected and characterised in the MUD faecal sample. The virus family and its characteristics are shown. We identified these viruses using the reference sequences mentioned in column 4 to which the NGS reads were mapped. We found how much the reads of our viruses are identical to the closest virus from the NCBI dataset using MEGA 6 or 7 software. However, for individual reads generated by NGS, we used BLASTN to identify the closest virus from the NCBI dataset. Their likely source of origin in our sample was determined using the NGS reads generated and correlating them from literature.

**Table 2:** Viruses detected in the six juvenile Pacific black duck (MAD) faecal sample pool.

Virus	Family	Characteristics	NCBI virus reference sequence used for mapping	Percentage of identity to the closest virus for individual NGS reads	Likely source
Avian paramyxovirus 6	*Paramyxoviridae, Avulavirus*	Negative-stranded RNA linear genome; enveloped, spherical. Diameter of about 150 nm.	AB759118	95–98%	Host
Gammacoronavirus	*Coronaviridae, Coronavirinae; Gammacoronavirus*	Monopartite, linear ssRNA(+) genome; enveloped, spherical, about 120 nm in diameter	KM454473	81–100%	Host
Deltacoronavirus	*Coronaviridae, Coronavirinae; Deltacoronavirus*	Monopartite, linear ssRNA(+) genome; enveloped, spherical, about 120 nm in diameter	NC016994	82–89%	Host
Virus related to chicken/duck/goose megrivirus	*Picornaviridae, Megrivirus;* unclassified *Megrivirus*.	Monopartite, linear ssRNA(+) genome; non-enveloped, spherical, about 30 nm in diameter	KC663628 and NC023857	77–83%	Host
Rotavirus G	*Reoviridae, Sedoreovirinae; Rotavirus*	Segmented linear dsRNA genome; non-enveloped with a double capsid structure	NC021580	80–89%	Host
Virus related to goose adenovirus 4 and duck adenovirus 2	*Adenoviridae; Aviadenovirus*	Non-segmented, linear dsDNA; non-enveloped capsid with a pseudo T = 25 icosahedral symmetry	NC024486 and NC017979	75–87%	Host
Virus related to duck dependovirus/AAV	*Parvoviridae; Parvovirinae; Dependoparvovirus*	Linear, ssDNA genome, Non-enveloped, round, T = 1 icosahedral symmetry, 18–26 nm in diameter with capsid	KX583629	88%	Host
Avian encephalomyelitis virus	*Picornaviridae; Tremovirus*	Monopartite, linear ssRNA(+) genome; non-enveloped, spherical, about 30 nm in diameter	AY517471	82–86%	Host
Avian calicivirus: related to chicken/goose calicivirus	*Caliciviridae*	Monopartite, linear ssRNA(+) genome, non-enveloped, capsid of about 27–40 nm in diameter, with T = 3 icosahedral symmetry	NC024078	70–84%	Host
Virus related to Hubei picorna-like virus 19	Unclassified RNA viruses	positive sense ssRNA genome	KX883724	79–82%	Leech
Virus related to Hubei picorna-like virus 51	Unclassified RNA viruses	positive sense ssRNA genome	KX883953	98%	Dragonfly
Bacteriophage related to Enterobacteria phage N4	*Caudovirales; Podoviridae*	Linear, dsDNA genome; non-enveloped, head-tail structure	EF056009	79–87%	Bacteria

This table displays the viruses detected and characterised in the MAD faecal sample pool. The virus family and its characteristics are shown. We identified these viruses using the reference sequences mentioned in column 4 to which the NGS reads were mapped. We found how much the reads of our viruses are identical to the closest virus from the NCBI dataset using MEGA 6 or 7 software. However, for individual reads generated by NGS, we used BLASTN to identify the closest virus from the NCBI dataset. Their likely source of origin in our sample was determined using the NGS reads generated and correlating them from literature.

#### Viruses characterised in the Australian Muscovy duck sample (MUD)

The nine viruses that were characterised from the MUD faecal sample turned out to provide an insight into the diet of the duck as none of them appeared to be avian viruses, but several of them likely from e.g. mosquitoes eaten by the Muscovy duck [Table 1]. These viruses belong to taxonomic families *Mesoniviridae, Dicistroviridae, Iridoviridae, Partitiviridae, Endornaviridae, Myoviridae* or related to taxonomically unclassified RNA viruses. We also included a single bacteriophage that was found in a high amount to show that our method also extends to bacteriophage characterisation if needed.

#### Viruses characterised in Australian juvenile Pacific black ducks sample (MAD)

In contrast to the above MUD sample, several avian host associated viruses were found in the MAD faecal sample [Table 2]. We characterised a total of 12 viruses from the NGS data generated which belonged to virus families of *Paramyxoviridae, Coronaviridae, Picornaviridae, Reoviridae, Adenoviridae, Parvoviridae, Caliciviridae, Podoviridae* or were related to known, but taxonomically unclassified RNA viruses. Among the 12 viruses, nine were avian host associated/infecting viruses while we also detected one leech and one dragonfly virus, likely derived from food eaten by the ducks. While several possible bacteriophages were identified in this sample, we only present results for one bacteriophage as an example, the Enterobacteria phage N4 like virus, which is not known to integrate into its host’s DNA, and therefore the reads identified being most likely from true virus particles [Table 2].

### Partial genome and evolutionary analysis of selected viruses

Among the 21 viruses found in the MUD and MAD faecal samples, a more in-depth analysis of the assembled sequences of 10 viruses was performed. This included both RNA and DNA viruses that had areas of good coverage and good quality NGS reads as described in the methods. The consensus sequences generated have been submitted to NCBI. Description of the sequences on the representative phylogenetic trees of three avian viruses from MAD (avian paramyxovirus 6, avian deltacoronavirus and avian adenovirus) and one virus from MUD (segment 1 of Hubei chryso-like virus 1) faecal samples are given in Table 3, and others are given in the Supplementary Material 2.

**Table 3:** Description of the sequences on the representative phylogenetic trees generated for four selected viruses analysed using MEGA 6 or 7 software.

Figure and Table	Long Name (Format: Sample-virus-protein-length-quality-coverage-year)	Short name (Format: Sample-virus-protein-length)	Coverage	No. of nucleotides	Mapping quality threshold	NCBI accession number
**Avian Paramyxovirus 6 from MAD faecal sample**						
2 and 4	MAD-Avian-paramyxovirus-6-large-polymerase-protein-459nt-Q32-C-9-192-2016	MAD-APMV6-Pol-459nt	9–192	459	32	MH000419
3 and 5	MAD-Avian-paramyxovirus-6-hemagglutinin-neuraminidase-1839nt-Q32-C-5-103_2016	MAD-APMV6-HN-1839nt	5–103	1839	32	MH000415
4 and 6	MAD-Avian-paramyxovirus-6-fusion-protein-651nt-Q32-C-2-8-2016	MAD-APMV6-FP-651nt	2–8	651	32	MH000412
**Long Name**		**Short Name (Format: NCBI accession number-virus-country/state)**	**Country of collection**	**Collection date**		
**NCBI sequences taken for phylogenetic analysis for APMV6**						
AB759118-Avian-paramyxovirus-6-viral-cRNA-complete-genome-strain:red-necked-stint/Japan/8KS0813/2008		AB759118-APMV6-JP	Japan	2008		
GQ406232-Avian-paramyxovirus-6-strain-duck/Italy/4524-2/07-complete-genome		GQ406232-APMV6-IT	Italy	2007		
KP762799-Avian-paramyxovirus-6-isolate-red-crested-pochard/Balkhash/5842/2013-complete-genome		KP762799-APMV6-KZ	Kazakhstan	2013		
AY029299-Avian-paramyxovirus-6-complete-genome		AY029299-APMV6-TW	Taiwan	—		
KT962980-Avian-paramyxovirus-6-isolate-teal/Novosibirsk_region/455/2009-complete-genome		KT962980-APMV6-RU	Russia	2009		
JN571486-Avian-paramyxovirus-6-strain-APMV6/mallard/Belgium/12245/07-nucleoprotein(NP)-phosphoprotein(P)-matrix-protein(M)-fusion-protein(F)-small-hydrophobic-protein(SH)-hemagglutinin-neuramis		JN571486-APMV6-BE	Belgium	2007		
KF267717-Avian-paramyxovirus-6-isolate-mallard/Jilin/127/2011-complete-genome		KF267717-APMV6-CN	China	2011		
**Deltacoronavirus from the MAD faecal sample**						
5 and 8	MAD-Deltacoronavirus-Orf1a-10650nt-Q20-C-6-2326−2016	MAD-DCoV-Orf1a-10650nt	6–2326	10650	20	MH013332
6 and 9	MAD-Deltacoronavirus-Orf1b-polymerase-2076nt-Q20-C-5-475-2016	MAD-DCoV-RPP-2076nt	5–475	2076	20	MH013331
7 and 10	MAD-Deltacoronavirus-spike-glycoprotein-3702nt-Q20-C-36-6274-2016	MAD-DCoV-SP-3702nt	36–6274	3702	20	MH013337
**NCBI sequences taken for phylogenetic analysis for DCoV**						
JQ065049-Common-moorhen coronavirus-HKU21-strain-HKU21-8295-complete-genome		JQ065049-MCoV-CN	China	2007		
JQ065046-Magpie-robin-coronavirus HKU18-strain-HKU18-chu3-complete-genome		JQ065046-MrCoV-CN	China	2007		
FJ376622-Munia-coronavirus-HKU13-3514-complete-genome		FJ376622-MuCoV-CN	China	2007		
FJ376621-Thrush-coronavirus-HKU12-600-complete-genome		FJ376621-TCoV-CN	China	2007		
MF431743-Porcine-deltacoronavirus-strain-SD-complete-genome		MF431743-PDCoV-CN	China	2014		
FJ376620-Bulbul-coronavirus-HKU11-796-complete-genome		FJ376620-BCoV-CN	China	2007		
JQ065047-Night-heron-coronavirus-HKU19-strain-HKU19-6918-complete genome		JQ065047-NhCoV-CN	China	2007		
JQ065048-Wigeon-coronavirus-HKU20-strain-HKU20-9243-complete-genome		JQ065048-WCoV-CN	China	2008		
**Figure and Table**	**Long Name (Format: Sample-virus-protein-length-quality-coverage-year)**	**Short name (Format: Sample-virus-protein-length-year)**	**Coverage**	**No. of nucleotides**	**Mapping quality threshold**	**NCBI accession number**
**Virus related to goose adenovirus 4 (GoA4) and/or duck adenovirus 2 (DuA2) from the MAD faecal sample**						
GoA4						
8 and 11	MAD-Adenovirus-encapsidation-protein-IVa2-279nt-Q20-C-4-69-2016	MAD-AV-IVa2-279nt	4–69	279	20	MH028885
DuA2						
9 and 12	MAD-Adenovirus-III-177nt-Q20-C-21-261-2016	MAD-AV-III-177nt	21–261	177	20	MH028886
10 and 13	MAD-Adenovirus-pVIII-114nt-Q32-C-19-67-2016	MAD-AV-pVIII-114nt	19–67	114	32	MH028887
**NCBI sequences taken for phylogenetic analysis for AV**						
KR135164-Duck-adenovirus-2-strain-CH-GD-12-2014-complete-genome		KR135164-DAd2-CN	China	2014		
JF510462-Goose-adenovirus-4-strain-P29-complete-genome		JF510462-GAd4-HU	Hungary	—		
FN824512-Pigeon-adenovirus-1-complete-genome-strain-IDA4		FN824512-PAd1-NL	Netherlands	1995		
KC493646-Fowl-adenovirus-5-strain-340-complete-genome		KC493646-FAd5-IE	Ireland	1970		
**Hubei chryso-like virus 1 from the MUD faecal sample**						
11 and 14	MUD-Hubei-chryso-like-virus-1-seg1-RdRp-1496nt-Q20-C-4-58-2016	MUD-HCLV1-s1-Rp-1496nt	4–58	1496	20	MH085092
**Long Name**		**Short Name (Format: NCBI accession number-virus-segment-country/state)**	**Country or State of collection**	**Collection date**		
**NCBI sequences taken for phylogenetic analysis for HCLV1**						
Segment 1						
MF176368-Hubei-chryso-like-virus-1-strain-mosWSgb49785-segment1-complete-sequence		MF176368-HCLV1-s1-WA	Western Australia	2015		
MF176309-Hubei-chryso-like-virus-1-strain-mos191gb77171-segment1-complete-sequence		MF176309-HCLV1-s1-WA	Western Australia	2015		
MF176261-Hubei-chryso-like-virus-1-strain-mos172gb42656-segment1-complete-sequence		MF176261-HCLV1-s1-WA	Western Australia	2015		
MF176388-Hubei-chryso-like-virus-1-strain-mosWSX51080-segment-1-complete-sequence		MF176388-HCLV1-s1-WA	Western Australia	2015		
MF176280-Hubei-chryso-like-virus-1-strain-mos172X13576-segment1-complete-sequence		MF176280-HCLV1-s1-WA	Western Australia	2015		
KX882962-Hubei-chryso-like-virus-1-strain-mosHB233224-hypothetical-protein-gene-partial-cds		KX882962-HCLV1-CN	China	2013		

The table gives details of the sequences used for phylogenetic analysis by the Maximum Likelihood method of APMV6, DCoV, AV and HCLV1. The first half of the table for each virus provides details to identify the sample from which the virus was isolated, protein encoded by the consensus sequences that are being analysed, the length of the consensus sequences and gene being analysed, minimum mapping quality threshold used in IGV for the generation of the consensus sequences, the coverage of the consensus sequences from IGV, the year of collection of the sample and the NCBI accession number assigned to the particular consensus sequence. Corresponding short names have been used in the phylogenetic trees which contain the sample, virus, protein and the number of nucleotides. The second half of the table for each virus provides the details of the sequences that were used for the comparative molecular phylogenetic analysis. They were found to be the most closely related sequences to the consensus sequences generated using BLASTN. Corresponding short names have been used in the phylogenetic trees which contain the NCBI accession number, virus and the country of collection. The collection date is given as retrieved from the corresponding NCBI nucleotide reference dataset which together with the country of collection can provide an insight into the possible evolution of the virus through the years.

### In the MAD sample

#### Avian paramyxovirus 6 (APMV6)

**Figure 2:**
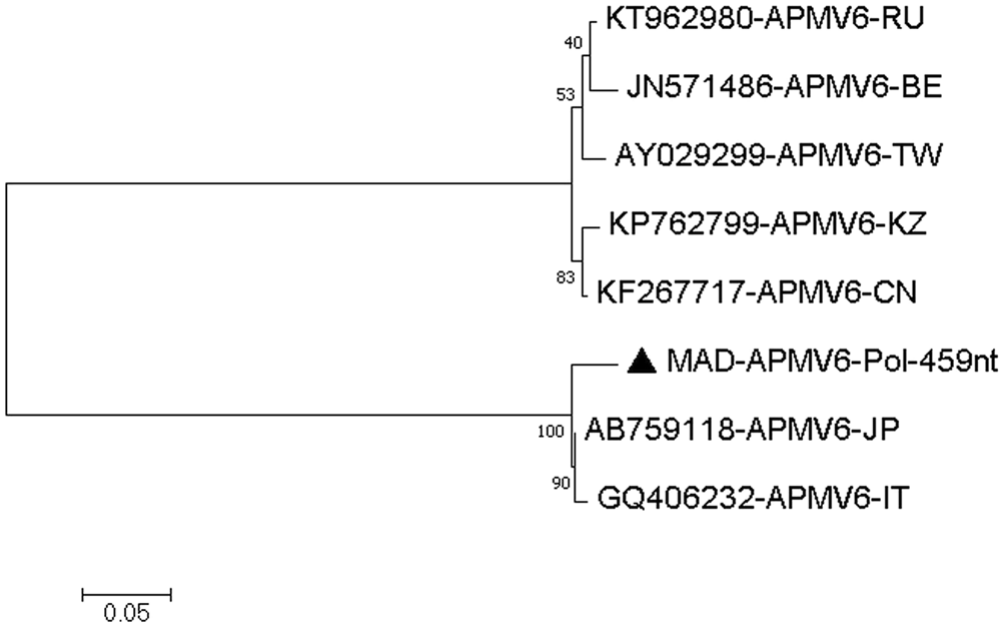
Molecular Phylogenetic analysis by Maximum Likelihood method of APMV6 partial Pol gene. The evolutionary history was inferred by using the Maximum Likelihood method based on the Tamura 3-parameter model^70^. The tree with the highest log likelihood (−1237.61) is shown. The percentage of trees in which the associated taxa clustered together is shown next to the branches. Initial tree(s) for the heuristic search were obtained automatically by applying Neighbor-Join and BioNJ algorithms to a matrix of pairwise distances estimated using the Maximum Composite Likelihood (MCL) approach, and then selecting the topology with superior log likelihood value. A discrete Gamma distribution was used to model evolutionary rate differences among sites (5 categories (+G, parameter = 0.5082)). The tree is drawn to scale, with branch lengths measured in the number of substitutions per site. The analysis involved 8 nucleotide sequences. Codon positions included were 1st+2nd+3rd+Noncoding. All positions containing gaps and missing data were eliminated. There were a total of 459 positions in the final dataset. Evolutionary analyses were conducted in MEGA7^63^.

**Figure 3:**
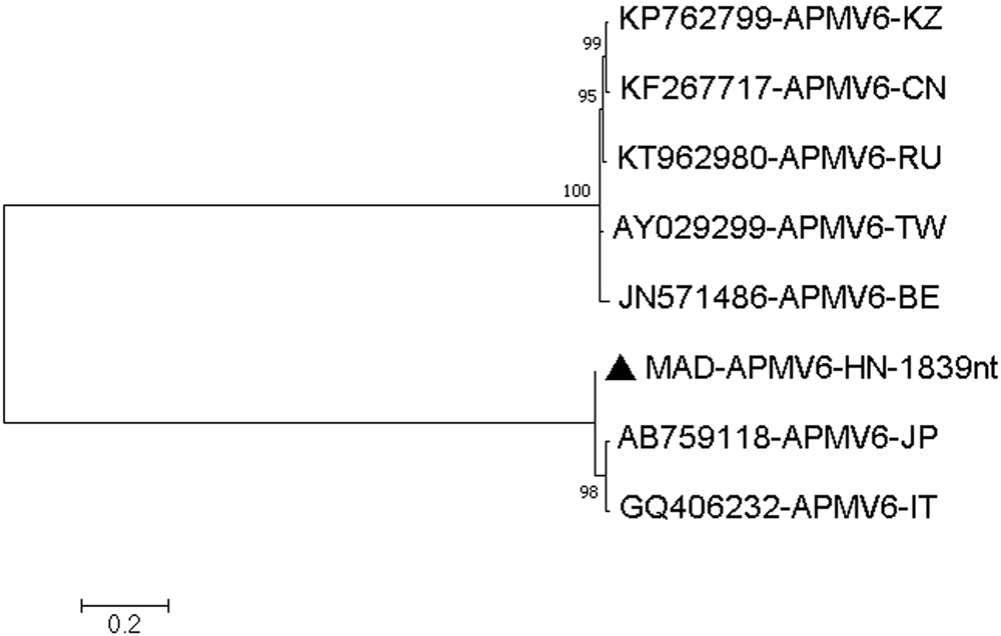
Molecular Phylogenetic analysis by Maximum Likelihood method of APMV6 complete HN gene. The evolutionary history was inferred by using the Maximum Likelihood method based on the Tamura 3-parameter model^70^. The tree with the highest log likelihood (−5515.26) is shown. The percentage of trees in which the associated taxa clustered together is shown next to the branches. Initial tree(s) for the heuristic search were obtained automatically by applying Neighbor-Join and BioNJ algorithms to a matrix of pairwise distances estimated using the Maximum Composite Likelihood (MCL) approach, and then selecting the topology with superior log likelihood value. The rate variation model allowed for some sites to be evolutionarily invariable ([+*I*], 54.95% sites). The tree is drawn to scale, with branch lengths measured in the number of substitutions per site. The analysis involved 8 nucleotide sequences. Codon positions included were 1st+2nd+3rd+Noncoding. All positions containing gaps and missing data were eliminated. There were a total of 1842 positions in the final dataset. Evolutionary analyses were conducted in MEGA7^63^.

**Figure 4:**
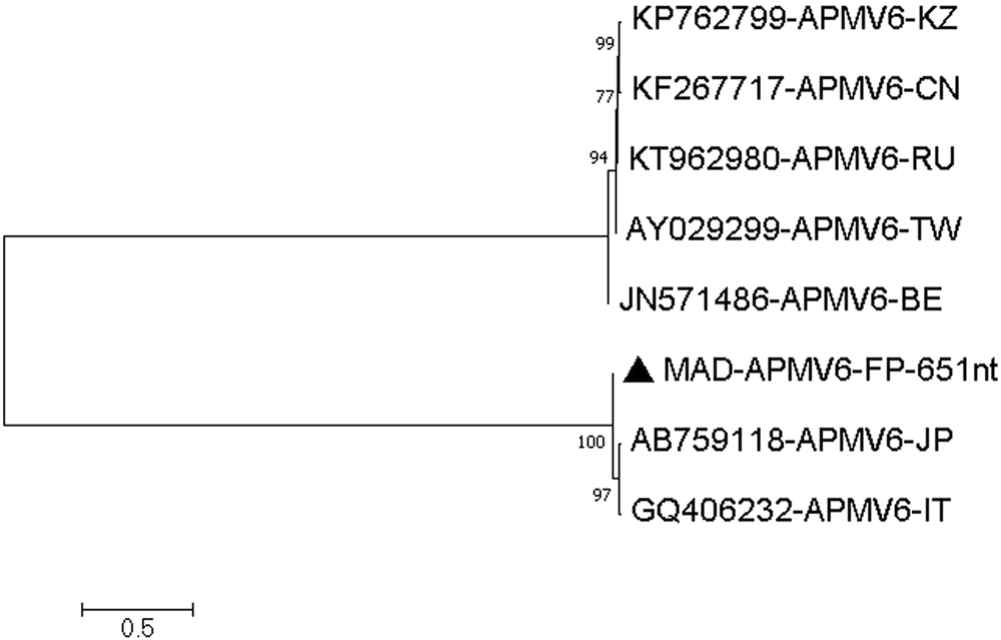
Molecular Phylogenetic analysis by Maximum Likelihood method of APMV6 partial FP gene The evolutionary history was inferred by using the Maximum Likelihood method based on the Kimura 2-parameter model^71^. The tree with the highest log likelihood (−1837.17) is shown. The percentage of trees in which the associated taxa clustered together is shown next to the branches. Initial tree(s) for the heuristic search were obtained automatically by applying Neighbor-Join and BioNJ algorithms to a matrix of pairwise distances estimated using the Maximum Composite Likelihood (MCL) approach, and then selecting the topology with superior log likelihood value. A discrete Gamma distribution was used to model evolutionary rate differences among sites (5 categories (+G, parameter = 0.4985)). The rate variation model allowed for some sites to be evolutionarily invariable ([+I], 59.29% sites). The tree is drawn to scale, with branch lengths measured in the number of substitutions per site. The analysis involved 8 nucleotide sequences. Codon positions included were 1st+2nd+3rd+Noncoding. All positions containing gaps and missing data were eliminated. There were a total of 651 positions in the final dataset. Evolutionary analyses were conducted in MEGA7^63^.

The MAD sample had many high-quality reads (Q32 or higher) that could be mapped to avian paramyxovirus type 6 (APMV-6 AB759118), a ssRNA virus belonging to the family *Paramyxoviridae*, with individual reads being 95–98% identical to AB759118 (APMV-6 red-necked stint-Japan-2008)^15^. We assembled several areas of good coverage including 459 nucleotides of the polymerase gene (coverage 9–192), 1839 nucleotides covering the complete hemagglutinin-neuraminidase (HN) gene (coverage 5–103) and 651 nucleotides of the fusion (F) gene (coverage 2–8). For the polymerase gene, the similarity was 447/459 (97.3%) and 444/459 (96.7%) to AB759118 (APMV-6 red-necked stint-Japan-2008)^15^ and GQ406232 (APMV-6 duck-Italy-07)^16^, but only around 75% similar to the other known lineage of APMV-6, the so-called Hong Kong 1977 lineage^17^ [Fig. 2 and Table 4]. For the HN gene, the similarity was 1787/1839 (97.1%) and 1785/1839 (97.0%) to AB759118 (APMV-6 red-necked stint-Japan-2008) and GQ406232 (APMV-6 duck-Italy-07), but only around 71% similar to the other known lineage of APMV-6 [Fig. 3 and Table 5]. For the fusion gene, the similarity was 631/651 (96.9%) and 630/651 (96.7%) to AB759118 (APMV-6 red-necked stint-Japan-2008) and GQ406232 (APMV-6 duck-Italy-07), but only around 73% similar to the other known lineage of APMV-6 [Fig. 4 and Table 6]. Thus, the APMV-6 present in our sample from the Australian juvenile Pacific black ducks (MAD) was closely related to the red-necked stint-Japan-2008 and duck-Italy-07 lineage of APMV-6 and only distantly related to the other major lineage of APMV-6. Furthermore, our Australian APMV-6 also has an additional basic amino acid around the fusion protein cleavage site, REPR-L rather than PEPR-L, characteristic for the Japan/Italy 2007/2008 lineage of APMV-6^17^. APMV6 sequences covering other regions of the APMV6 genome similarly found that the virus belonged to the Japan-Italy lineage **[**Figures S1.1–S1.23 and Table S1.1–S1.23 of the Supplementary Material 2].

**Table 4:** Estimates of Evolutionary Divergence between Sequences.

**MAD-APMV6-Pol-459nt**							
AB759118-APMV6-JP	12						
GQ406232-APMV6-IT	15	3					
KP762799-APMV6-KZ	114	113	114				
AY029299-APMV6-TW	116	115	116	15			
KT962980-APMV6-RU	115	114	115	12	9		
JN571486-APMV6-BE	114	115	116	18	11	8	
KF267717-APMV6-CN	116	115	116	5	12	9	15

The number of base differences between sequences are shown. The analysis involved 8 nucleotide sequences. Codon positions included were 1st+2nd+3rd+Noncoding. All positions containing gaps and missing data were eliminated. There were a total of 459 positions in the final dataset. Evolutionary analyses were conducted in MEGA7.

**Table 5:** Estimates of Evolutionary Divergence between Sequences.

**MAD-APMV6-HN-1839nt**							
AB759118-APMV6-JP	52						
GQ406232-APMV6-IT	54	24					
KP762799-APMV6-KZ	532	541	537				
AY029299-APMV6-TW	532	541	537	47			
KT962980-APMV6-RU	536	542	538	35	44		
JN571486-APMV6-BE	535	540	538	72	50	63	
KF267717-APMV6-CN	537	544	540	24	51	39	74

The number of base differences between sequences are shown. The analysis involved 8 nucleotide sequences. Codon positions included were 1st+2nd+3rd+Noncoding. All positions containing gaps and missing data were eliminated. There were a total of 1842 positions in the final dataset. Evolutionary analyses were conducted in MEGA7.

**Table 6:** Estimates of Evolutionary Divergence between Sequences.

**MAD-APMV6-FP-650nt**							
AB759118-APMV6-JP	20						
GQ406232-APMV6-IT	21	7					
KP762799-APMV6-KZ	174	174	174				
AY029299-APMV6-TW	172	175	173	14			
KT962980-APMV6-RU	174	176	174	12	6		
JN571486-APMV6-BE	169	176	174	32	22	26	
KF267717-APMV6-CN	173	173	173	4	14	12	32

The number of base differences between sequences are shown. The analysis involved 8 nucleotide sequences. Codon positions included were 1st+2nd+3rd+Noncoding. All positions containing gaps and missing data were eliminated. There were a total of 651 positions in the final dataset. Evolutionary analyses were conducted in MEGA7.

#### Gammacoronavirus

The MAD faecal sample had high-quality reads (Q32 or higher) that could be mapped to duck coronavirus (KM454473) with individual reads being 81–100% identical to KM454473; a duck gammacoronavirus isolate DK/GD/27/2014 isolated from ducks in China in 2014^18^. We assembled several areas of good coverage including 1071 nucleotides (coverage 4–65) of the NSP12 (RNA dependant RNA polymerase (RdRP)) gene, and two areas of the spike (S) gene of 524 nucleotides (coverage 3–19) and 469 nucleotides (coverage 3–13), respectively.

For the NSP12 RdRp gene, our sequence had 1030/1071 nucleotides (96.1%) match to KM454473 and 886–917/1071 nucleotides (82.7–95.6%) match to other related gammacoronaviruses. For the Spike gene, the similarity for the area of 524 nucleotides was 470/524 nucleotides (89.6%) and 365–381/524 nucleotides (69.6–72.7%) and for the area of 469 nucleotides, the similarity was 431/469 nucleotides (91.8%) and 336–343/469 nucleotides (71.6–73.1%) when aligned to KM454473 and other related gammacoronaviruses, respectively **[**Figures S2.1–S2.3 and Tables S2.1–S2.3 of the Supplementary Material 2**]**. At the amino acid level, the 357 amino acids of the NSP12 RdRp gene had 353/357 amino acids (98.8%) and 326–346/357 amino acids (91.3–96.9%) identity to KM454473 and to other related gammacoronaviruses, respectively. Thus, a gammacoronavirus was detected in the Pacific black ducks which is somewhat related to a duck gammacoronavirus from China with a similarity at the nucleotide level of more than 96% in the NSP12 RdRp gene, but only 89–92% in the Spike gene.

#### Deltacoronavirus

Samples from the Pacific black ducks (MAD) had high-quality reads (Q20 or higher) with individual reads being 82–89% identical to JQ065048; a wigeon deltacoronavirus isolate from Hong Kong collected in 2008^19^. A 201-nucleotide segment of the spike (S) gene was assembled. This had 81.5% (164/201) similarity to wigeon deltacoronavirus and 69.6–74.1% (140–149/201) similarity to the next nearest deltacoronaviruses (Figure S3.1 and Table S3.1 of the Supplementary Material 2). At the amino acid level, the 67 amino acids encoded by this small region had 62/67 amino acids (92.5%) and 50–55/67 amino acids (74.6–82.0%) identity to JQ065048 and other related deltacoronaviruses, respectively (Figure S3.2 and Table S3.2 of the Supplementary Material 2). We also assembled two small areas of the NSP12 RdRp gene (Q20 or higher and coverage 3–30 and 4–21, respectively) having 166/205 nucleotides (81.0%) and 146–159/205 nucleotides (71.2–77.5%) for one area and 96/117 nucleotides (82.0%) and 89–94/117 nucleotides (76.0–80.3%) for the other area, match to JQ065048 and other related deltacoronaviruses, respectively (Figures S3.3, S3.4 and Table S3.3, S3.4 of the Supplementary Material 2).

Two large contigs of 14822 and 8322 nucleotides and two smaller contigs spanning parts of the deltacoronavirus Orf1ab replicase gene and the S1-S2 (Spike) and E, M and NS6 genes were obtained from the assemblerSPAdes analysis, and the NGS reads mapped to these contigs using the TMAP assembler to determine the coverage depth. From further analysis, we initially selected an area of 1131 nucleotides (coverage 290–1700) from the Spike gene and 2121 nucleotides (coverage 39–1266) from the Orf1ab gene which overlaps with NSP7, NSP8, NSP9 and NSP10. These sequences were then analysed using MEGA and aligned with ClustalW by codons. For the selected Spike gene segment alignment, the deltacoronavirus sequence had a 738/1131 nucleotides (65.2%) and 695–710/1131 nucleotides (61.4–62.7%) match to JQ065048 and other related deltacoronaviruses, respectively. At the amino acid level, this segment had a 277/376 amino acids (73.7%), and 227–248/376 amino acids (61.5–66.0%) match to JQ065048 and other related deltacoronaviruses, respectively (Figures S3.5, S3.6 and Table S3.5, S3.6 of the Supplementary Material 2). The selected Orf1ab segment of the deltacoronavirus sequence had a 1414/2121 nucleotides (66.6%) and 1203–1317/2121 nucleotides (56.7–62%) match to JQ065048 and other related deltacoronaviruses, respectively. At the amino acid level, the sequence had a 463/706 amino acids (65.6%), and 377–388/706 amino acids (53.4–55.0%) match to JQ065048 and other related deltacoronaviruses, respectively (Figures S3.7, S3.8 and Tables S3.7, S3.8 of the Supplementary Material 2).

**Figure 5:**
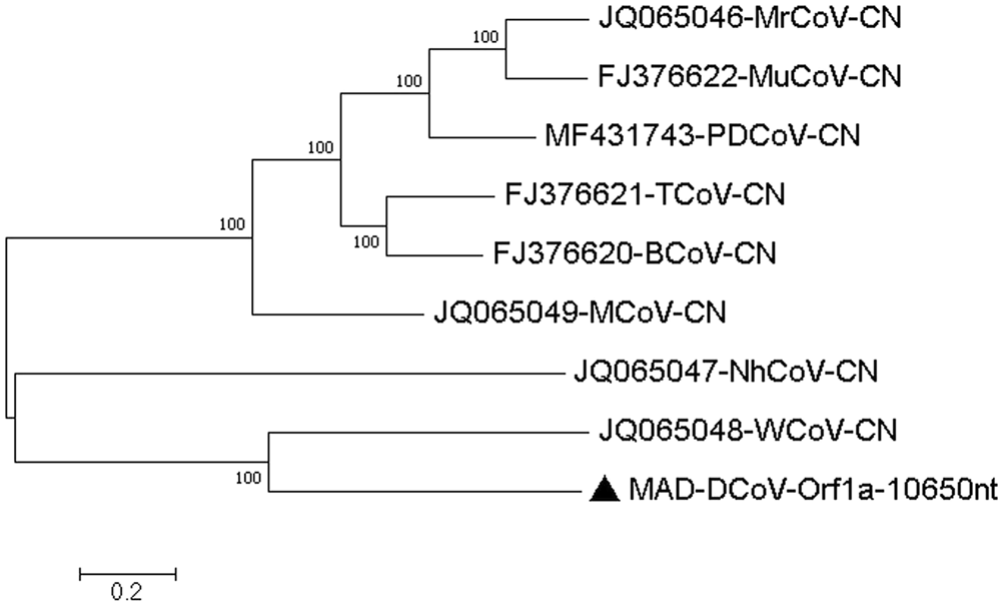
Molecular Phylogenetic analysis by Maximum Likelihood method of DCoV Orf1a gene The evolutionary history was inferred by using the Maximum Likelihood method based on the General Time Reversible model^72^. The tree with the highest log likelihood (−87186.89) is shown. The percentage of trees in which the associated taxa clustered together is shown next to the branches. Initial tree(s) for the heuristic search were obtained automatically by applying Neighbor-Join and BioNJ algorithms to a matrix of pairwise distances estimated using the Maximum Composite Likelihood (MCL) approach, and then selecting the topology with superior log likelihood value. A discrete Gamma distribution was used to model evolutionary rate differences among sites (5 categories (+G, parameter = 1.4644)). The tree is drawn to scale, with branch lengths measured in the number of substitutions per site. The analysis involved 9 nucleotide sequences. All positions containing gaps and missing data were eliminated. There were a total of 10137 positions in the final dataset. Evolutionary analyses were conducted in MEGA7^63^.

**Figure 6:**
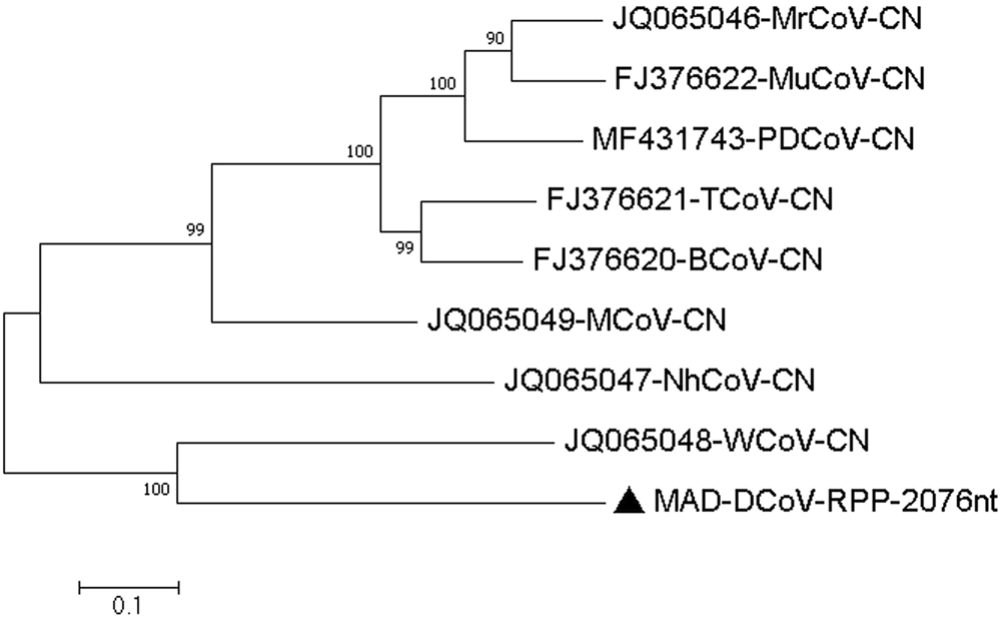
Molecular Phylogenetic analysis by Maximum Likelihood method of DCoV partial RPP gene The evolutionary history was inferred by using the Maximum Likelihood method based on the General Time Reversible model^72^. The tree with the highest log likelihood (−12370.44) is shown. The percentage of trees in which the associated taxa clustered together is shown next to the branches. Initial tree(s) for the heuristic search were obtained automatically by applying Neighbor-Join and BioNJ algorithms to a matrix of pairwise distances estimated using the Maximum Composite Likelihood (MCL) approach, and then selecting the topology with superior log likelihood value. A discrete Gamma distribution was used to model evolutionary rate differences among sites (5 categories (+*G*, parameter = 0.5773)). The rate variation model allowed for some sites to be evolutionarily invariable ([+*I*], 25.10% sites). The tree is drawn to scale, with branch lengths measured in the number of substitutions per site. The analysis involved 9 nucleotide sequences. All positions containing gaps and missing data were eliminated. There were a total of 2076 positions in the final dataset. Evolutionary analyses were conducted in MEGA7^63^.

**Figure 7:**
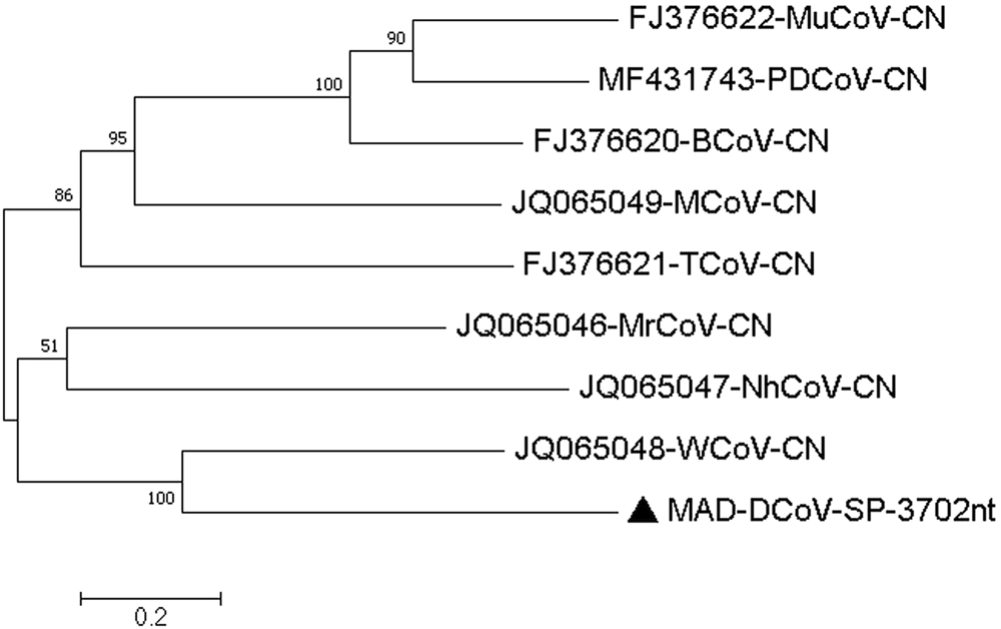
Molecular Phylogenetic analysis by Maximum Likelihood method of DCoV partial SP gene The evolutionary history was inferred by using the Maximum Likelihood method based on the General Time Reversible model^72^. The tree with the highest log likelihood (−30555.18) is shown. The percentage of trees in which the associated taxa clustered together is shown next to the branches. Initial tree(s) for the heuristic search were obtained automatically by applying Neighbor-Join and BioNJ algorithms to a matrix of pairwise distances estimated using the Maximum Composite Likelihood (MCL) approach, and then selecting the topology with superior log likelihood value. A discrete Gamma distribution was used to model evolutionary rate differences among sites (5 categories (+G, parameter = 1.7612)). The rate variation model allowed for some sites to be evolutionarily invariable ([+I], 11.02% sites). The tree is drawn to scale, with branch lengths measured in the number of substitutions per site. The analysis involved 9 nucleotide sequences. All positions containing gaps and missing data were eliminated. There were a total of 3312 positions in the final dataset. Evolutionary analyses were conducted in MEGA7^63^.

We then expanded the analysis to larger areas of these mapped contigs (Q value at 20 or above and coverage of at least 5 and for some areas up to 6400), dividing areas into open reading frames (to avoid stop codons and thus making Clustal W codon based alignment possible). For all selected areas, the Wigeon coronavirus JQ065058 was the closest related sequence, and the percentage identities to the Wigeon and other related deltacoronavirus sequences at the nucleotide and amino acid levels are shown in Table 7. Selected phylogenetic trees are shown in Figs 5–7 and Tables 8–10 while other phylogenetic trees are shown in Figures S3.9–S3.23 and Tables S3.9–S3.23 of the Supplementary Material 2.

**Table 7:** Assembled deltacoronavirus contigs divided into partial protein-coding genes identified using BLASTX and compared to the closest related deltacoronaviruses in the NCBI database.

	Length nucleotides	Percentage Identity nucleotides*	Percentage identity amino acids*
Orf1a (NSP 3, 4, 5, 7, 8, 9 & 10)	10650	51.3–**60.2**	41.2–**55.2**
Orf1bStart (NSP 10, 12)	1095	60.5–**71.1**	61.5–**75.5**
Orf1b-pol (NSP 12)	2076	67.4–**73.2**	76.6–**81.9**
Orf1b (NSP11)	1914	63.4–**68.7**	65.6–**74.6**
Orf1b (NSP11 & 13)	2619	58.6–**65.4**	58.1–**67.5**
Spike-S1-S2	3702	56.5–**61.5**	48.8–**59.2**
E	261	53.6–**71.6**	40.7–**74.4**
M	654	56.4–**70.2**	53.2–**78.9**
NS6	276	49.3–**69.6**	38.0–**60.9**

The table displays the percentage identity of nucleotides and amino acids of generated deltacoronavirus contigs to the closest related deltacoronaviruses in the NCBI database (Wigeon deltacoronavirus JQ065048). The percentage was determined using MEGA 6 software. *The highest percentage identity is indicated in boldface and was consistently found when compared to the Wigeon deltacoronavirus JQ065048.

**Table 8:** Estimates of Evolutionary Divergence between Sequences.

JQ065049-MCoV-CN								
JQ065046-MrCoV-CN	3984							
FJ376622-MuCoV-CN	3769	2302						
FJ376621-TCoV-CN	3388	3579	3397					
MF431743-PDCoV-CN	3814	2979	2991	3311				
FJ376620-BCoV-CN	3500	3464	3356	2534	3223			
JQ065047-NhCoV-CN	4883	5173	5054	4962	5073	4838		
JQ065048-WCoV-CN	4855	5174	5085	4987	5107	4956	5052	
**MAD-DCoV-Orf1a-10650nt**	4782	5188	5127	4983	5148	4980	5100	4240

The number of base differences between sequences are shown. The analysis involved 9 nucleotide sequences. Codon positions included were 1st+2nd+3rd+Noncoding. All positions containing gaps and missing data were eliminated. There were a total of 10137 positions in the final dataset. Evolutionary analyses were conducted in MEGA7.

**Table 9:** Estimates of Evolutionary Divergence between Sequences.

JQ065049-MCoV-CN								
JQ065046-MrCoV-CN	497							
FJ376622-MuCoV-CN	465	269						
FJ376621-TCoV-CN	434	403	356					
MF431743-PDCoV-CN	504	323	327	388				
FJ376620-BCoV-CN	447	393	366	287	385			
JQ065047-NhCoV-CN	590	613	577	597	585	573		
JQ065048-WCoV-CN	572	656	625	615	634	599	628	
**MAD-DCoV-RPP-2076nt**	566	676	629	618	659	627	629	557

The number of base differences between sequences are shown. The analysis involved 9 nucleotide sequences. Codon positions included were 1st+2nd+3rd+Noncoding. All positions containing gaps and missing data were eliminated. There were a total of 2076 positions in the final dataset. Evolutionary analyses were conducted in MEGA7.

**Table 10:** Estimates of Evolutionary Divergence between Sequences.

JQ065049-MCoV-CN								
JQ065046-MrCoV-CN	1521							
FJ376622-MuCoV-CN	1431	1531						
FJ376621-TCoV-CN	1428	1547	1504					
MF431743-PDCoV-CN	1447	1566	1040	1493				
FJ376620-BCoV-CN	1341	1543	1102	1484	1058			
JQ065047-NhCoV-CN	1535	1509	1590	1507	1552	1564		
JQ065048-WCoV-CN	1489	1507	1533	1551	1544	1542	1599	
**MAD-DCoV-SP-3072nt**	1548	1563	1612	1598	1594	1602	1593	1427

The number of base differences between sequences are shown. The analysis involved 9 nucleotide sequences. Codon positions included were 1st+2nd+3rd+ Noncoding. All positions containing gaps and missing data were eliminated. There were a total of 3312 positions in the final dataset. Evolutionary analyses were conducted in MEGA7.

#### Virus related to goose adenovirus 4 (GoA4) and duck adenovirus 2 (DuA2)

**Figure 8:**
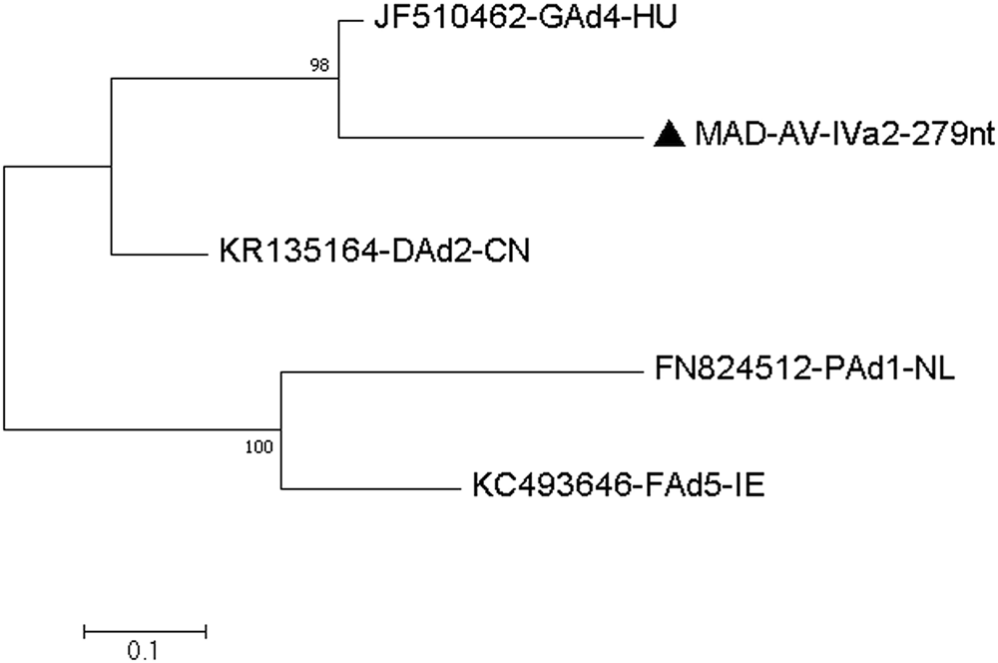
Molecular Phylogenetic analysis by Maximum Likelihood method of AV partial IVa2 gene The evolutionary history was inferred by using the Maximum Likelihood method based on the Kimura 2-parameter model^71^. The tree with the highest log likelihood (−1171.71) is shown. The percentage of trees in which the associated taxa clustered together is shown next to the branches. Initial tree(s) for the heuristic search were obtained automatically by applying Neighbor-Join and BioNJ algorithms to a matrix of pairwise distances estimated using the Maximum Composite Likelihood (MCL) approach, and then selecting the topology with superior log likelihood value. A discrete Gamma distribution was used to model evolutionary rate differences among sites (5 categories (+G, parameter = 0.8585)). The tree is drawn to scale, with branch lengths measured in the number of substitutions per site. The analysis involved 5 nucleotide sequences. Codon positions included were 1st+2nd+3rd+Noncoding. All positions containing gaps and missing data were eliminated. There were a total of 279 positions in the final dataset. Evolutionary analyses were conducted in MEGA7^63^.

**Figure 9:**
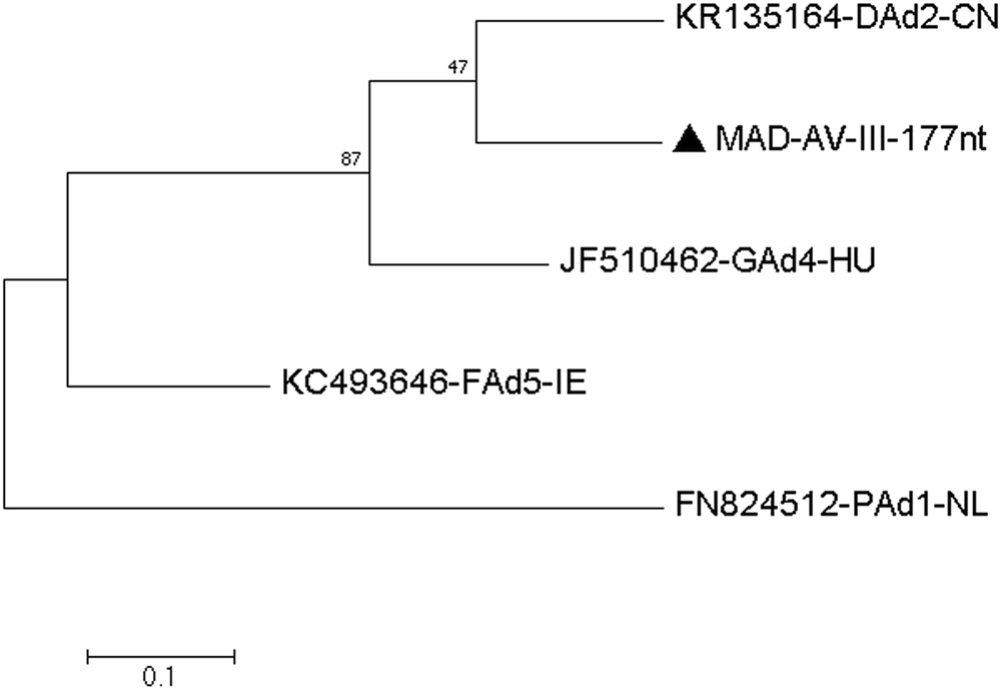
Molecular Phylogenetic analysis by Maximum Likelihood method of AV partial III gene The evolutionary history was inferred by using the Maximum Likelihood method based on the Kimura 2-parameter model^71^. The tree with the highest log likelihood (−805.13) is shown. The percentage of trees in which the associated taxa clustered together is shown next to the branches. Initial tree(s) for the heuristic search were obtained automatically by applying Neighbor-Join and BioNJ algorithms to a matrix of pairwise distances estimated using the Maximum Composite Likelihood (MCL) approach, and then selecting the topology with superior log likelihood value. A discrete Gamma distribution was used to model evolutionary rate differences among sites (5 categories (+ G, parameter = 1.2536)). The tree is drawn to scale, with branch lengths measured in the number of substitutions per site. The analysis involved 5 nucleotide sequences. Codon positions included were 1st+2nd+3rd+Noncoding. All positions containing gaps and missing data were eliminated. There were a total of 177 positions in the final dataset. Evolutionary analyses were conducted in MEGA7^63^.

**Figure 10:**
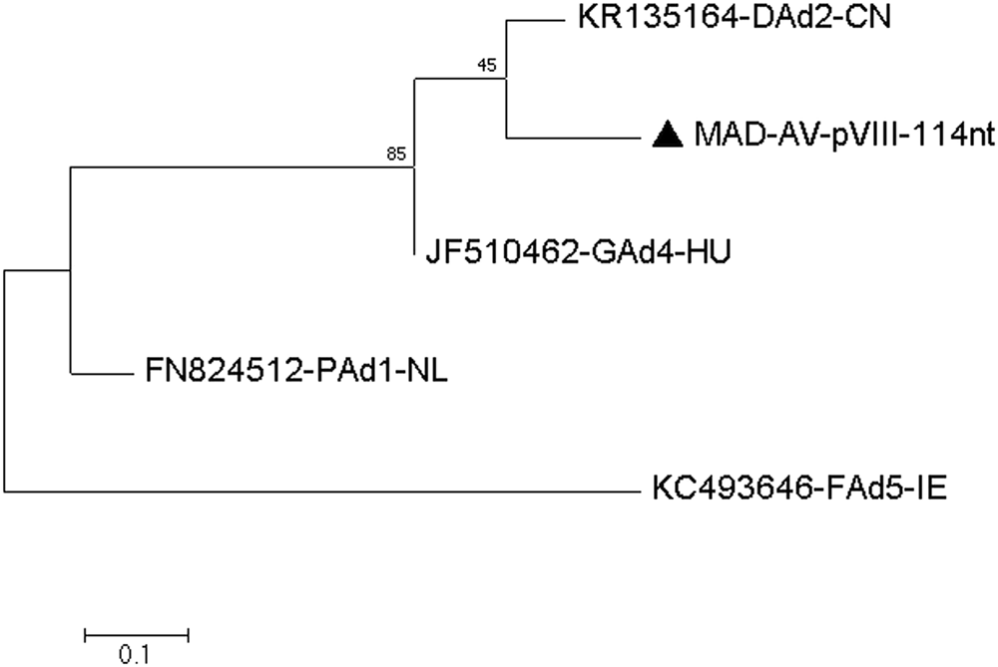
Molecular Phylogenetic analysis by Maximum Likelihood method of AV partial pVIII gene The evolutionary history was inferred by using the Maximum Likelihood method based on the Tamura 3-parameter model^70^. The tree with the highest log likelihood (−415.13) is shown. The percentage of trees in which the associated taxa clustered together is shown next to the branches. Initial tree(s) for the heuristic search were obtained automatically by applying Neighbor-Join and BioNJ algorithms to a matrix of pairwise distances estimated using the Maximum Composite Likelihood (MCL) approach, and then selecting the topology with superior log likelihood value. The rate variation model allowed for some sites to be evolutionarily invariable ([+I], 62.84% sites). The tree is drawn to scale, with branch lengths measured in the number of substitutions per site. The analysis involved 5 nucleotide sequences. Codon positions included were 1st+2nd+3rd+Noncoding. All positions containing gaps and missing data were eliminated. There were a total of 114 positions in the final dataset. Evolutionary analyses were conducted in MEGA7^63^.

A dsDNA virus belonging to the family *Adenoviridae* and approximately 75–86% identical to goose adenovirus 4 (JF510462) from Hungary^20^ and duck adenovirus 2 (KR135164) from China^21^ was found in the MAD faecal sample. The assembled sequences were analysed further including a 279 nucleotides area of the IVa2 protein coding region (Q20 or higher, coverage 4–69), a 177 nucleotides area of the IU34_gp11 gene (Q20 or higher, coverage 21–261) and a 114 nucleotides area of the IU34_gp21 gene (Q32 or higher, coverage 19–67). We found the similarity of assembled sequences to be 229/279 nucleotides (82%) match to the GoA4 and 210/279 nucleotides (75.26%) match to the DuA2 [Fig. 8 and Table 11], 143/177 nucleotides (80.7%) match to the DuA2, and 140/177 nucleotides (79.0%) match to the GoA4 [Fig. 9 and Table 12], 99/114 nucleotides (86.8%) match to the DuA2, and 96/114 nucleotides (84.2%) match to the GoA4 [Fig. 10 and Table 13], respectively. All other sequences covering other regions of the adenovirus genome similarly found that this virus is related to both GoA4 and DuA4 **[**Figures S4.1–S4.10 and Tables S4.1–S4.10 of the Supplementary Material 2.

**Table 11:** Estimates of Evolutionary Divergence between Sequences.

KR135164-DAd2-CN				
JF510462-GAd4-HU	53			
FN824512-PAd1-NL	83	89		
KC493646-FAd5-IE	78	79	66	
**MAD-AV-IVa2–279nt**	69	50	112	90

The number of base differences between sequences are shown. The analysis involved 5 nucleotide sequences. Codon positions included were 1st+2nd+3rd+Noncoding. All positions containing gaps and missing data were eliminated. There were a total of 279 positions in the final dataset. Evolutionary analyses were conducted in MEGA7.

**Table 12:** Estimates of Evolutionary Divergence between Sequences.

KR135164-DAd2-CN				
JF510462-GAd4-HU	40			
FN824512-PAd1-NL	61	76		
KC493646-FAd5-IE	55	50	63	
**MAD-AV-III-177nt**	34	37	78	54

The number of base differences between sequences are shown. The analysis involved 5 nucleotide sequences. Codon positions included were 1st+2nd+3rd+Noncoding. All positions containing gaps and missing data were eliminated. There were a total of 177 positions in the final dataset. Evolutionary analyses were conducted in MEGA7.

**Table 13:** Estimates of Evolutionary Divergence between Sequences.

KR135164-DAd2-CN				
JF510462-GAd4-HU	13			
FN824512-PAd1-NL	27	22		
KC493646-FAd5-IE	29	33	28	
**MAD-AV-pVIII-114nt**	15	18	28	30

The number of base differences between sequences are shown. The analysis involved 5 nucleotide sequences. Codon positions included were 1st+2nd+3rd+Noncoding. All positions containing gaps and missing data were eliminated. There were a total of 114 positions in the final dataset. Evolutionary analyses were conducted in MEGA7.

#### Virus related to duck dependovirus/adeno-associated virus (AAV)

A ssDNA virus belonging to the family *Parvoviridae* was identified in the MAD faecal sample. This virus was found to be related to a duck Adeno-associated virus (KX583629) from China^21^ with individual reads being 84–93% identical to KX583629. Several regions of this virus including 94-nucleotides area of the rep protein (Q20 or higher, coverage 4–5), 279-nucleotides area of the capsid protein (Q32 or higher, coverage 7–27) and a 142-nucleotides area of the capsid protein (Q32 or higher, coverage 3–4) were assembled. We matched these regions to KX583629 and other adeno-associated viruses respectively. We found the 94-nucleotides area of rep protein had 88/94 nucleotides (93.6%), the 279-nucleotides area of the capsid protein had 250/279 nucleotides (89.6%), and the 124-nucleotides area of the capsid protein had 120/142 nucleotides (84.5%) match to the adeno-associated virus from China. Thus, in our analysis, we found the adeno-associated virus from the Pacific black ducks from Australia to be somewhat related to the duck adeno-associated virus from China^21^. Representative phylogenetic trees generated using the consensus sequences are shown in Figures S5.1–S5.3 and Tables S5.1–S5.3 in the Supplementary Material 2.

#### Rotavirus G

The faecal sample from the Pacific black ducks had good quality individual reads (Q32 or higher) with 80–89% identity to Rotavirus G chicken segment 2 (NC021580) and 4 (JQ920005) (Note: Designated as segment 4 as per the submitted NCBI reference sequence, but appear to be VP3 and possibly segment 3) isolated from Germany^22^ and Rotavirus G pigeon segment 1 and 8 (KC876010 and KC876006 respectively) isolated from China^23^. Rotavirus G is a segmented, dsRNA virus belonging to the Reoviridae family. For this virus, good coverage of 3 or higher with a mapping quality threshold of 20 or higher was not obtained. To the best of our knowledge, these sequences are the first assembled reads of a Rotavirus G from Australia. Hence, to undergo a phylogenetic analysis of the virus, the mapping quality threshold was dropped to 10 or higher to get coverage of 3 and above. Three regions each from segment 1, 3/4 and 8 with individual areas being a 230-nucleotides area of the VP1 protein of segment 1 (coverage 3–14), a 162-nucleotides area of the VP3 protein of segment 3/4 (coverage 3–7) and a 149-nucleotides area of the NSP2 protein of segment 8 (coverage 3–6) were analysed. The 230-nucleotides area of the VP1 protein had a 186/230 nucleotides (80.8%) match to segment 1 of KC876010 Rotavirus G pigeon from China, the 162 nucleotides area of the VP3 protein had a 142/162 nucleotides (87.6%) match to segment 3/4 for Rotavirus G chicken from Germany and the 149-nucleotides area of the NSP2 protein had a 130/149 nucleotides (87.2%) match to segment 8 of Rotavirus G pigeon from China **[**Figures S6.1–S6.3 and Tables S6.1–S6.3 in the Supplementary Material 2]. This analysis shows that the Pacific black duck rotavirus G is somewhat related to rotavirus G of chicken and pigeon and could be a variant of rotavirus G that have evolved and adapted to infect ducks. To the best of our knowledge, this is the first Rotavirus G sequences to be assembled from a duck sample.

### In the MUD sample

#### Hubei chryso-like virus 1

**Figure 11:**
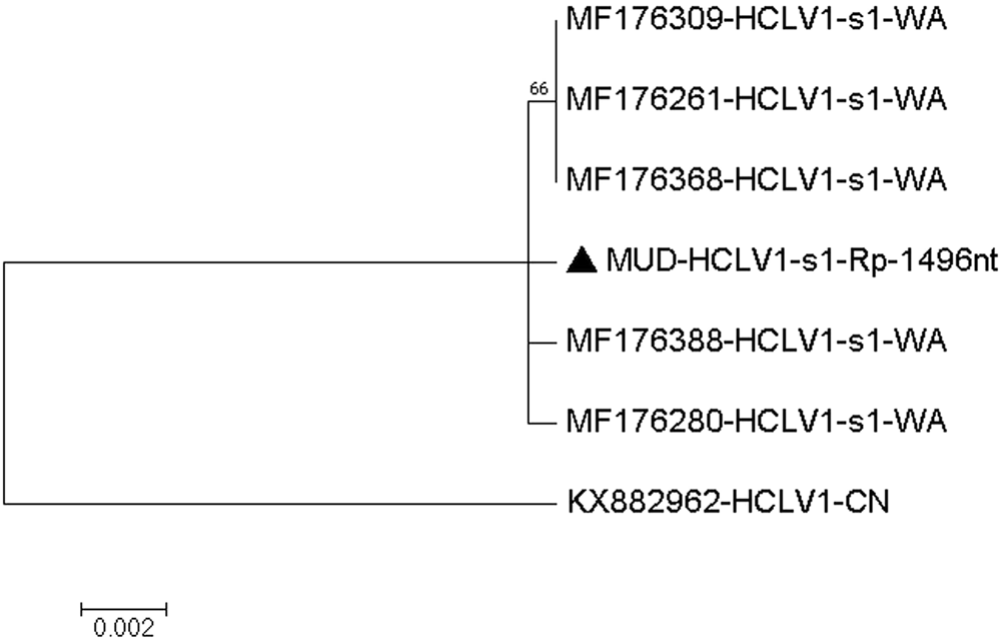
Molecular Phylogenetic analysis by Maximum Likelihood method of HCLV1 partial Rp gene The evolutionary history was inferred by using the Maximum Likelihood method based on the Tamura 3-parameter model^70^. The tree with the highest log likelihood (−2284.45) is shown. The percentage of trees in which the associated taxa clustered together is shown next to the branches. Initial tree(s) for the heuristic search were obtained automatically by applying Neighbor-Join and BioNJ algorithms to a matrix of pairwise distances estimated using the Maximum Composite Likelihood (MCL) approach, and then selecting the topology with superior log likelihood value. The tree is drawn to scale, with branch lengths measured in the number of substitutions per site. The analysis involved 7 nucleotide sequences. Codon positions included were 1st+2nd+3rd+Noncoding. All positions containing gaps and missing data were eliminated. There were a total of 1496 positions in the final dataset. Evolutionary analyses were conducted in MEGA7^63^.

Highly identical, and good quality reads to the four segments of the Hubei chryso-like virus 1 initially identified in China^1^ and recently also identified in mosquitoes from Western Australia^24^ were found in the Muscovy duck (MUD) sample. We assembled several areas of good quality reads (Q20 or higher) for segment 1 including a 1496-nucleotides area of the polymerase gene (coverage 4–58) with 1494/1496 nucleotides (99.8%) match to segment 1 of Hubei chryso-like virus 1 (MF176261, MF176388, MF176368, MF176309 and MF176280) from Western Australia^24^ and 1458/1496 nucleotides (97.4%) match to segment 1 of the Hubei chryso-like virus 1 from China^1^ (KX882962) (Fig. 11 and Table 14). Another 2175-nucleotides area of the putative protease gene (Q32 or higher, coverage 4–71) from segment 2 of the virus was also analysed. For this gene, it was found to have 2166–2168/2175 nucleotide (99.5–99.6%) match to Hubei chryso-like virus 1, segment 2 (MF176389, MF176262, MF176310, MF176281 and MF176369) from Western Australia; it should be mentioned that for the Hubei chryso-like virus 1 from China mentioned above, only the sequence for segment 1 is available. For segment 3 of Hubei chryso-like virus 1, a 536-nucleotides area of a hypothetical protein area (Q32 or higher, coverage 9–42) were analysed. It had 532–534/536 nucleotides (99.2–99.6%) match to Hubei chryso-like virus 1, segment 3 (MF176390, MF176263, MF176370 and MF176282) from mosquitoes in Western Australia. Finally, a 1938-nucleotides area of a hypothetical protein area from segment 4 of Hubei chryso-like virus 1 (Q32 or higher, coverage 3–121) was analysed. It had 1930–1932/1938 nucleotides (99.5–99.6%) match to segment 4 of the other Hubei chryso-like virus 1 sequences from Western Australia (MF176391, MF176312, MF176283, MF176264 and MF176371) **[**Figures. S7.1–S7.3 and Tables S7.1–S7.3 of the Supplementary Material 2]. Thus, the sequences in our MUD sample for Hubei chryso-like virus 1 is nearly identical to the Western Australia Hubei chryso-like virus 1 strains and based on the available segment 1 sequence, also relatively closely related to the virus from Hubei in China (KX882962).

**Table 14:** Estimates of Evolutionary Divergence between Sequences.

**MUD-HCLV1-s1-Rp-1496nt**						
MF176388-HCLV1-s1-WA	2					
MF176368-HCLV1-s1-WA	2	2				
MF176309-HCLV1-s1-WA	2	2	0			
MF176280-HCLV1-s1-WA	2	2	2	2		
MF176261-HCLV1-s1-WA	2	2	0	0	2	
KX882962-HCLV1-CN	38	38	38	38	38	38

The number of base differences between sequences are shown. The analysis involved 7 nucleotide sequences. Codon positions included were 1st+2nd+3rd+Noncoding. All positions containing gaps and missing data were eliminated. There were a total of 1496 positions in the final dataset. Evolutionary analyses were conducted in MEGA7.

#### Culex Negev-like virus 3

The MUD sample had good quality reads (Q32 or higher) that were assembled and found to be closely related to Culex Negev-like virus 3 which is a ssRNA virus detected in mosquitoes from Western Australia (MF176277)^24^. For this virus, two regions from the polymerase gene with a mapping quality threshold of 32 or higher including a 531-nucleotides area (coverage 3–22) and a 275-nucleotides area (coverage 5–30) and one region from the membrane protein encoding region of 456-nucleotides area (coverage 3–19) were analysed. The polymerase sequences were found to have a 529/531 nucleotides (99.6%), and a 271/275 nucleotides (98.5%) match to the Culex Negev-like virus 3 from Western Australia (MF176277). The membrane protein encoding region had a 451/456 nucleotides (98.9%) match to the Culex Negev-like virus 3 from Western Australia (MF176277). Our sequences were also found to be somewhat closely related to Biggievirus (MF281708; MF281709 and KX924639) as evident from the phylogenetic tree given in Figures S8.1–S8.3 and Tables S8.1–S8.3 of the Supplementary Material 2.

#### Hubei reo-like virus 7-like virus

From the MUD faecal sample, virus sequences with similarity to the Hubei reo-like virus 7, an unclassified dsRNA virus was assembled. We were able to generate good quality reads (Q20 or higher) distributed throughout the genome. As this virus is a relatively newly characterised virus from mosquitoes in China^1^ and not closely related to any other hitherto sequenced viruses, our sequences only resemble that from China. From the polymerase gene of the virus, partial gene sequences that were assembled from the NGS dataset including a 240-nucleotides area (Q20 or higher, coverage 3–11), a 184-nucleotides area (Q32 or higher, coverage 3–6) and a 117-nucleotides area (Q32 or higher, coverage 4–5) were analysed. We found that the assembled sequences of the polymerase gene had a 227/240 nucleotides (94.5%) match, a 161/184 nucleotides (87.5%) match and a 111/117 nucleotides (94.8%) match to the Hubei reo-like virus 7 (KX884635) isolated from China **[**Figures S9.1–S9.3 and Tables S9.1–S9.3 of the Supplementary Material 2]. Interestingly, the translated amino acid sequences of these nucleotide sequences were highly conserved with 78/80 amino acids; 61/61 amino acids and 38/39 amino acids respectively (data not shown).

#### Enterobacteria phage phi92

As an example, a sequence of 277 nucleotides with coverage of 2–4 and a Q value of 32 or higher was assembled from an Enterobacteria phage phi92. This belonged to the potential tail fibre protein with glycosidase activity/carbohydrate binding module. This sequence was 273/277 nucleotides (98.6%) identical to enterobacteria phage phi92 (FR775895)^25^, 270/277 (97.5%) to enterobacteria phage ECGD1 (KU522583) and between 210–223/277 (75.8–80.5%) similar to other Escherichia and Salmonella phages (see Figure S10.1 and Table S10.1 of the Supplementary Material 2). At the amino acid level the similarity to enterobacteria phages was even more striking, with the sequence from our Muscovy duck having 92/92 amino acids (100%) identity to both enterobacteria phage phi92 (FR775895) and enterobacteria phage ECGD1 (KU522583) and only a 73–77/92 amino acids (79.3–83.6%) match to other Escherichia and Salmonella phages (see Figure S10.2 and Table S10.2 of the Supplementary Material 2).

### Use of the optimised method on human faecal samples

To demonstrate that the optimised virus enrichment and NGS sequencing method can be utilised on other samples, we analysed human stool samples collected from 3 children aged 3 months (sample ST4), 18 months (ST5) and 4 years (ST6). The same protocol was followed (as for the duck samples), however, the analysis of NGS reads was restricted (alignment by the Ion S5 TMAP) to virus sequences that only included human single-stranded (ss) DNA viruses (32 human ssDNA virus species selected from NCBI on 7 Aug 2017), as well as known picornavirus reference sequences (180 picornavirus reference sequences downloaded from NCBI on 4 Aug 2017) in the NCBI databases.

#### ssDNA viruses detected

Samples ST4 and ST5 had good quality reads (Q10 or higher and coverage of 10–15) to a 116 nucleotide region of the 5′ untranslated region of torque teno mini virus. ST4 had a 113/116 (97.4%) and 111/116 (95.6%) match to KM259873 torque teno mini virus ALA22, and KT163899 torque teno virus isolate P13–4, respectively; and ST5 had 112/116 (96.8%) and 110/116 (94.8%) match to KT163899 torque teno virus isolate P13-4 and KM259873 torque teno mini virus ALA22^26^, respectively. Sample ST6 had a single high-quality read (Q value 46) with 198/208 (95.1%) nucleotide match to the hypothetical capsid coding region of MF118166 human faecal virus Tarto^27^. Representative phylogenetic trees are presented in Figures S11.1–S11.2 and Tables S11.1 and S11.2 of the Supplementary material 2.

#### Picornaviruses detected

Sample ST4 had reasonable quality reads (Q8 or higher and coverage of 5–6) with 157/163 (96.3%) nucleotide match to KY983589 human rhinovirus C11 strain SC3107. Sample ST6 had good quality reads (Q10 or higher and a coverage of 2–4) with 204/212 (96.2%), 185/212 (87.2%) and 184/212 (86.7%) nucleotide match to the 2 C region of AB747252 Saffold virus isolate Pak-3290^28^, EU681176 cardiovirus D/VI2229/2004^29^ and JN652233 Saffold virus strain S19, respectively. This sample, ST6, also had good reads (Q10 and above and coverage of 3–18) with 296/422 (70.1%), 405/422 (95.9%) and 403/422 (95.4%) nucleotide match to the VP3 region and 150/261 (57.4%), 250/261 (95.7%) and 251/261 (96.1%) nucleotide match to the VP1 region of AB747252 Saffold virus isolate Pak-3290, EU681176 cardiovirus D/VI2229/2004 and JN652233 Saffold virus strain S19, respectively. Taken together this indicates that a Saffold/cardiovirus is present in sample ST6 although recombination among strains appears to be part of its ancestry. The representative phylogenetic trees are shown in Supplementary material 2 Figures S11.3–S11.6 and Tables S11.3–S11.6.

From the contigs, a few larger contigs were selected that by BLASTN alignment contained sequences with a high degree of identity to human viruses or known bacteriophages. Contigs and closely related sequences in the NCBI databases were then used to map back the NGS reads using the TMAP assembler. Assembled BAM files were then analysed using IGV and areas with reasonable coverage and a Q value at 20 or higher selected for further analysis of consensus sequences by using BLASTN and BLASTX.

From sample ST4 a sequence of 909 nucleotides in length was assembled with a coverage of 3–52 reads and a close nucleotide match to rotavirus sequences in the NCBI databases, e.g. 908–909/909 nucleotides match (99.9–100%) to GU565088, the VP4 gene of a reassortant rotavirus vaccine strain (USA RotaTeq)^30^ and 880/909 nucleotides match (96.8%) to U53923, the VP4 gene from a bovine rotavirus isolated in the USA^31^ more than 20 years ago. It should be noted that the rotavirus sequence in our sample was almost certainly from the vaccine virus although at one position the GU565088 sequence in NCBI reportedly has a G nucleotide while our sequence had 12 reads (55%) with a G in this position and 10 reads with an A indicating a mixed nucleotide at this position albeit with a slight G nucleotide majority. Given that the RotaTeq vaccine is used in infants in Australia from 2 months of age, and the high genetic similarity to the vaccine virus VP4 gene, it is most likely that the detected rotavirus was a vaccine virus.

From sample ST5 we were able to assemble and map a sequence of 7044 nucleotides in length with coverage of 2–189 and a nucleotide match to norovirus sequences in the NCBI databases. It was found to have 7027/7044 nucleotides match (99.7%) to MG002634 and MG002632 Norovirus from Queensland, Australia in 2017 while showing 6743–6746/7044 nucleotides match (95.7%) to JX459901 and JX459904 (and covering 94.4% of the full norovirus genome), a GII norovirus from New South Wales, Australia in 2011^32^.

We also did phylogenetic inferences of these sequence datasets by Maximum Likelihood (ML) phylogenetic analyses using the MEGA 6/7 software, and the representative phylogenetic trees are shown in Supplementary material 2 Figures S11.7–S11.8 and Tables S11.7–S11.8.

### Additional information extracted from the NGS reads

In addition to virus enrichment, the optimised method also, (i) preserves a minor amount of host mitochondria/mitochondrial DNA, and, (ii) enriches for other small stable structures resistant or partially resistant to nucleases, such as, e.g. ribosomes. Thus, further scrutiny of the obtained NGS reads outside looking for viruses, can provide valuable information.

#### Species sampled

In the faecal samples from the Muscovy duck (*Cairina moschata*) there were good quality reads with a coverage of 23–66 and a 114/114 (100%) nucleotides match to the *Cairina moschata* mitochondrial COX1 gene (FJ808630)^33^ and only 104/114 (91.2%) to *Anas platyrhynchos* COX1 gene (KT803699) while the samples from the Pacific black ducks (coverage of 67–192) had a 114/114 (100%) nucleotides match to *Anas platyrhynchos* COX1 gene (KT803699) and only 104/114 (91.2%) to the *Cairina moschata* COX1 gene (FJ808630). For the mitochondrial cytochrome B gene (coverage 16–114), the samples from the Muscovy duck (*Cairina moschata*) had a 996/996 (100%) match to *Cairina moschata* (EU755254)^34^ but only around 89% match to *Anas platyrhynchos* while the samples from the Pacific black ducks had a 1012/1012 (100%) match to *Anas platyrhynchos* cytochrome B gene (KJ883269)^35^ and only around 88% to *Cairina moschata*. Consequently, the results “mined” from the NGS results supported the species of the birds sampled noting that Pacific black ducks (*Anas superciliosa*) are genotypically similar to the mallard (*Anas platyrhynchos*) or the bird that we sampled could be a cross-breed^36^.

#### Food eaten by the birds sampled and possible correlation to non-host viruses detected

As this was not the focus of this particular study, only a few examples are summarised; however, further mining of the data is likely to provide further insight into how much information can be extracted in order to get the full value of generated reads.

Consensus reads from the *Cairina moschata* samples showed a meager number of reads with matches of e.g. 139/139 (100%) and 176/177 ((99.4%) nucleotides match to *Culex* (quinquefasciatus) partial 28 S RNA (KY087523), while no such matches could be found in the Pacific black ducks sample that in contrast had many reads mapping to the 28 S RNA of tapeworms and other cestodes with, e.g. 167/167 (100%) nucleotides matches. Other reads could also be mapped to the 28 S ribosomal RNA of plants such as, e.g. triticum, sorghum, Oryza and mays. The finding of a low number of reads mapping to the ribosomal RNA of *Culex* mosquitoes is consistent with the abundant finding of *Culex* mosquito viruses in the *Cairina moschata* (MUD) sample and as no reads could be mapped to the *Culex* mitochondrial COX1 gene, indicate that the mosquito-associated viruses detected in the *Cairina moschata* faecal sample came from mosquitoes being eaten and going through the intestinal tract (thus completely digesting any mitochondria/mitochondrial DNA while not completely digesting ribosomes) rather than from potential mosquito contamination of the faecal sample before collection.

#### The “ribosomal activity microbiome”

The analysis of the NGS data using the ThermoFisher Ion Reporter microbiome software provided insight into the active bacterial communities present in collected faecal samples although did not necessarily correlate with results obtained by a conventional DNA extraction 16SrRNA microbiome diversity profiling (data not shown). Furthermore, an overall analysis of repeated samples indicated that while detection of viruses was very robust over time, the “ribosomal activity microbiome” is susceptible to freeze-thawing with the ratio of reads mapped to Gram-negative bacteria (particularly phylum Bacteroidetes) decreasing when compared to Gram-positive bacteria (particularly phylum Firmicutes).

## Discussion

The optimum protocol for the metagenomics detection and characterisation of viruses from a given sample should allow detection of all types of viruses, including single and double stranded RNA/DNA, circular, linear or segmented genome viruses; enveloped and non-enveloped viruses; and small and large viruses present. To establish such a protocol for the enrichment, detection and characterisation of a maximum number of viruses in complex biological samples such as faecal samples, we used a method previously described by others on a mock community called NetoVIR as the starting point and tested different enrichment techniques using various biophysical methods^6,37,38^. The use of marker viruses representing the different virus types enabled us to evaluate and optimise a metagenomics method suitable for detecting and characterising viruses from complex biological samples, in this study faecal samples.

The main aim was to minimise host and non-virus RNA/DNA and maintain as much virus RNA/DNA as possible to optimise subsequent random amplification of nucleic acids followed by NGS detection and characterisation. The method which maintained a maximum number of viruses and obtained high-quality and a higher number of NGS reads were found to be variation D; equivalent to the NetoVIR method with an added ultracentrifugation step to further concentrate and enrich for particles resistant to nucleases. However, variation C, or NetoVIR^6^ itself, also produced good quality NGS reads for the viruses, although in lower amounts compared to variation D. Careful homogenization was significant for the homogeneous distribution of virus particles in the samples. Initial centrifugation and filtration using the 0.8 µm PES filter as described in NetoVIR removed larger particles while virus particles and some small particles such as ribosomes and some mitochondria were not filtered out and were subsequently spun down and concentrated using ultracentrifugation and thus enriching the sample. Harsh nuclease treatment ensured the reduction of free nucleic acids and other less protected nucleic acids. Finally, the nucleic acid extraction method did allow the extraction of genomes of both DNA and RNA viruses from the sample.

Although detergent treatment may enhance enrichment of certain viruses, it was found to be disadvantageous for some enveloped viruses and thus not incorporated in the final protocol. We assume that the detergent disrupted the structure of the enveloped, non-inner capsid containing virus particles causing the nuclease to digest the virus nucleic acids. Filtration using 0.45 µm filters might also have negatively affected the virus enrichment process, as we were not able to produce many good reads for the marker viruses BTV, BVDV and IBV. Both our filtration results using 0.45 µm filtration and the detergent results, although in the NetoVIR study^6^ the authors used chloroform, correlate well with how the mock communities reacted to these enrichment techniques.

Unlike NetoVIR^6^, we used the SeqPlex RNA amplification kit instead of the Whole Transcriptome Amplification Kit 2 (WTA2, Sigma) for cDNA synthesis, amplification and primer removal. While library preparation and NGS was carried out using Nextera XT DNA Library Preparation kit (Illumina) and HiSeq™ 2500 platform (Illumina) in NetoVIR^6^, we used Ion Plus Fragment Library Kit (Thermo Fisher Scientific) and Ion Torrent S5XL System (Thermo Fisher Scientific). However, a recent study has shown that the selection of different sequencing platforms and corresponding library preparation methods have a minimal impact on viral metagenomes^39^.

While we detected and characterised 9 avian host associated viruses in the MAD faecal sample, it is noteworthy that no avian viruses were detected in the MUD faecal sample. We speculate that this is because of the age of the bird (a mature older bird of unknown age as compared to the MAD samples being from juveniles) and it, despite being a wild bird living outside, was from an urban-like environment with less interaction with many other wild birds.

Avian paramyxovirus 6 (APMV6) was first isolated in 1977 from a healthy duck at a domestic duck farm in Hong Kong and considered as the prototype strain for the entire serotype^40^. The virus seems to be non-pathogenic to chickens but can cause mild respiratory disease and a drop in egg production in turkeys^41^. While only distantly related to the Hong Kong lineage of APMV6 mentioned above, the APMV6 detected in the MAD faecal sample is around 95–98% identical to another lineage of APMV6 isolated from Japan^15^ and Italy^16^ in the year 2008 and 2007, respectively. Interestingly, the detection of the genetically similar APMV6 in Japan was from a red-necked stint (*Calidris ruficolis*), a migratory bird species which breeds in the Arctic, but migrates in the non-breeding season as far south as New Zealand and Southern Australia including the location from which the APMV6 from this study was collected. The fusion protein of the paramyxoviruses directs the membrane fusion^42^ and paramyxoviruses where the fusion protein cleavage site does not possess the furin recognition site (RXK/RR-F) are usually limited to the respiratory tract, and hence the sequence at this site contributes to the virulence of these viruses^42,43^. The sequences of the fusion protein in our APMV6 from the MAD faecal sample has an additional basic amino acid around the fusion protein cleavage site (REPR-L) characteristic for the Japan/Italy 2007/2008 lineage as compared to the Hong Kong lineage of APMV-6^17^ and hence any further changes at this site could potentially make the virus highly virulent.

Gammacoronaviruses and deltacoronaviruses both from the family *Coronaviridae*, have been previously detected in wild birds in other parts of the world^44^. Avian coronaviruses frequently cause severe infections in poultry in many countries^45^. Coronaviruses have also been associated with inter-species spill over with recent examples being Severe acute respiratory syndrome coronavirus and Middle Eastern respiratory syndrome coronavirus. The gammacoronavirus detected in the MAD faecal sample was more closely related to the duck gammacoronavirus from China in 2014^18^ than to the other gammacoronavirus sequences available. While we observed more than 96% identity in the Orf1ab polyprotein gene, the Spike gene identity was only 89–92% to the duck gammacoronavirus. For the deltacoronavirus detected in the MAD sample, the closest relative was the wigeon deltacoronavirus from Hong Kong collected in 2008^19^. The Spike protein of the coronaviruses mediates the entry of the virus particles into cells, and mutations in or recombination of this region are important for host species specificity and potential cross-species transmissibility of the virus^42,43^. These are the first wild bird coronaviruses from Australia to have their spike genes sequenced and further analysis to understand the relationship between wild bird coronaviruses is needed. A detailed study of the prevalence of coronaviruses among Australian wild birds (including the MAD sample) using PCR amplification of a small fragment of the Orf1ab gene with a detailed description and phylogenetic analysis of a small fragment of around 300 nucleotides is described elsewhere^46^. However, while that study did detect both gamma and deltacoronaviruses, the PCRs included were not able to detect coinfections (i.e. birds having both a gamma and a deltacoronavirus) while it is worth mentioning that such coinfection can be readily detected using our metagenomics method as evident from the NGS data generated in the present study.

In the MAD faecal sample, we also characterised two DNA viruses, one a virus related to DuA2 and/or GoA4 virus and another virus related to an adeno-associated (parvo) virus. The consensus sequences of the adenovirus that we assembled were related (75–86%) to both the DuA2 and GoA4 virus. The DuA2 (KR135164) was characterised in China in the year 2014 and found to be closely related to the GoA4 (JF510462) isolated from Hungary^20^. Avian adenoviruses have great variability in their pathogenesis and virulence. The goose adenovirus 4 has been associated with hepatitis and hydropericardium^20^. The virus related to adeno-associated (parvo) virus from the Pacific black ducks was somewhat related to a duck adeno-associated virus from China in 2015. It is currently unclear whether the adeno-associated (parvo) virus detected in the MAD sample can replicate without helper virus or not, however, we consider it likely that the adenovirus (related to the DuA2 and GoA4 mentioned above) detected in the same sample, may act as a helper virus for enhanced replication as adeno-associated parvoviruses often need a helper virus for its replication^47^. As both viruses are present simultaneously and possibly enhance replication of the parvovirus present, this co-infection may be associated with disease in the host although this is currently speculative and will require further studies before any conclusion can be reached as to its significance in regards to the health of infected birds.

Rotavirus G has also been previously found in birds^48^. Although infection with this particular serotype has not been considered fatal, infection from another rotavirus serotype such as Rotavirus A can be lethal to the birds in extreme cases^49,50^. Even though rotavirus infections in avian species are associated with diarrhoea, cross-species transmission of the avian rotaviruses is usually very rare^49^. To the best of our knowledge, our sequences are the first assembled reads of a Rotavirus G from Australia and from a duck sample, which could explain why we had to drop the mapping quality threshold to 10 to get a coverage of 3 and above for the generation of consensus sequences. Nevertheless, even then, we were only able to generate consensus sequences for only segments 1, 3/4 and 8. We believe the sequences for one segment that matched to segment 4 of the NCBI reference (JQ920005) isolated from Germany^22^ is in reality segment 3 as it encodes for the VP3 protein of the rotavirus, which universally is accepted to be part of segment 3. Nevertheless, it appears that the rotavirus detected in the MAD sample is a new rotavirus and may have included reassortment in its evolutionary history as we were only able to map 3 of the gene segments and they, in turn, mapped most closely to different reference sequences.

Other avian host associated viruses we identified in the Pacific black ducks sample (MAD) are related to avian megrivirus, avian encephalomyelitis virus and avian calicivirus. Among them, the avian megrivirus and avian encephalomyelitis virus belong to the virus family *Picornaviridae*. The avian viruses in this virus family have been associated with different diseases such as duck viral hepatitis, turkey viral hepatitis and avian encephalomyelitis^49,51^. Avian encephalomyelitis virus is a highly infective virus to bird species such as chickens, partridge, turkey, quail, guineafowl and pheasants. They can severely affect the neuro-system and has high mortality rates^49^. However, the pathogenicity of avian calicivirus is still unclear although it has been isolated from chickens and turkeys having various diseases like the runting and stunting syndrome and enteric diseases^52,53^. The actual pathogenicity, if any, of the related viruses here detected at very low levels in the MAD sample is currently unknown.

The non-avian host associated viruses that were characterised in both MUD and MAD faecal samples were likely part of the food eaten by the ducks. For example, for the mosquito viruses detected in our MUD faecal sample, our detection of partial 28 S RNA of *Culex* mosquito in the sample supports this speculation. Ngewotan virus (Nam Dinh like virus), Hubei chryso-like virus 1, Culex Negev-like virus 3 (Biggie/Goutanap virus-like) and a virus related to Hubei reo-like virus 7 detected and characterised in the MUD sample are recently identified in mosquitoes^1,24^. All the mosquito viruses, except for the virus related to Hubei reo-like virus 7 (which was first characterised in China^1^) are nearly 100% identical to the viruses detected in Western Australia^24^, approximately 3000 km from Geelong, Victoria where the MUD faecal sample was collected. These viruses being RNA viruses, which can have high mutation rates due to the low polymerase fidelity^54^, a nearly 100% identity is rather interesting. The mode of travel of these viruses could be from birds that have eaten the infected mosquitoes and travel long distances and then could be depositing these viruses in their faeces. We also cannot rule out that the infected mosquitoes may be taken from one region to another by human means such as aircrafts, trains, vehicles or by the wind. Independent of mode, the close identity of these mosquito viruses from distant locations indicate that such viruses may be easily dispersed over great distances.

Virus related to invertebrate iridescent virus 30 has previously been found in moths^55^ and viruses related to Hubei picorna-like virus 19 and Hubei picorna-like virus 51, here detected and characterised in the MAD sample, were recently identified in leech and dragonfly, respectively^1^. Presence of plant viruses such as a virus related to black-grass cryptic like virus 2 and a virus related to Hordeum vulgare endornavirus is also likely to be from the food eaten. Only a single long good quality read with high similarity to the Israeli acute paralysis virus was detected. This virus may cause devastating infection in bees (colony collapse) when associated with varroa mite infestations. This virus has previously been detected in Australian bees, however, as Australia is free of varroa mites, is not thought to cause any disease in bees here^56,57^. Nevertheless, the finding in the MUD sample likely suggests that it could be from a bee eaten by the Muscovy duck.

Some of the other viruses identified in the duck samples belonging to the virus families *Picornaviridae*, *Adenoviridae*, *Parvoviridae*, *Partitiviridae*, *Endornaviridae*, *Myoviridae*, *Iridoviridae* and *Calciviridae* were only around 70–80% similar to the closet sequences in the database (see Tables 1 and 2). A closer examination of these related/like viruses may lead to the identification and full characterisation.

Birds may serve as hosts, carriers or transporters of parasites and pathogens^58^. Birds that migrate through the East Asian-Australasian flyway may transmit viruses to and from Australia. The similarity of some of our sequences of the avian and non-avian host associated viruses to the viruses that were characterised in e.g. Japan and China supports this theory. For example, the APMV6 phylogenies suggest that despite Australian ducks not migrating, they still are part of the global circulation of duck viruses, and that migrating shorebirds may likely be the vector for such viruses entering and exiting from Australia. It is also noteworthy to mention that during the BLASTN search, except for the Hubei chryso-like virus 1, Ngewotan virus (Nam Dinh like virus) and Culex Negev-like virus 3 very recently described in Western Australia, none of the top matched sequences were to similar strains or related viruses from Australia. Hence to the best of our knowledge we are the first to provide virus sequences from Australia for avian paramyxovirus 6, avian adeno-associated parvovirus and avian calicivirus.

The optimised virus enrichment metagenomics method presented here was able to not only provide information on viruses within the gut of a bird, but also identify the species of the bird, indicate dietary preferences and identify bacterial populations present from a fresh faecal sample. It is therefore a non-invasive technique that could have application to ecological and wildlife health surveys, and can be applied to many different species, including humans. Furthermore, the technique could be applied to different biological samples including saliva and tissue samples (data not shown) and be of use to medical and veterinary diagnostics.

The “ribosomal activity microbiome” that was revealed using the optimised method may provide valuable information about the composition of live and active bacteria in faecal samples. Ribosomal RNA is partially protected from nuclease activity^59^, and as the optimised method allows ribosomes to be enriched together with the virus particles, the ribosomal profile of the faecal microbiome can also be investigated. These results provide a indication of the “ribosomal activity microbiome”, i.e. the microbiome of active bacteria in the final deposited feces as active bacteria have a much higher number of ribosomes per bacteria (up to 70000 ribosomes or more per bacteria) than resting or dead bacteria (7000 or fewer ribosomes per bacteria)^60,61^. Although the results based on this method cannot necessarily be directly compared to microbial assessment by 16 S DNA diversity profiling, understanding of the active bacteria may be important^62^. The decrease that was observed in the Bacteroidetes to Firmicutes ratio with repeated freeze-thaw cycles may also be useful to assess the prior freeze-thaw/storage history of a sample.

Finally, we also would like to mention, that in addition to the bird and human faecal samples described here, the optimized method has been tested on dog faecal and saliva samples and on pig lung/tracheal swab samples with good results (data not shown) indicating that the method may have broad applicability for different sample types and species.

## Materials and Methods

### Samples and marker viruses for spiking samples

Fresh faecal samples from one Muscovy duck (MUD) and a pooled sample from six juvenile Pacific black ducks (MAD) were collected in November and December 2016 from south-eastern Victoria, Australia (Geelong and Connewarre), respectively. These samples were stored at −80 °C within 1–3 hours of its collections and until its processing. Bird sample collection was approved under Deakin University’s Animal Ethics Committee project number B43–2016 and Department of Environment, Land, Water and Planning permit number 1008206.

Marker viruses for spiking samples (Table S1 of Supplementary material 1) were obtained from the CSIRO-Australian Animal Health Laboratory and the Elizabeth Macarthur Agricultural Institute. All virus stocks were stored at −80 °C.

Human stool samples collected from 3 children aged 3 months (sample ST4), 18 months (ST5) and 4 years (ST6) from Geelong were also used to test the optimised method. The study involving these samples here were performed in accordance with relevant guidelines and regulations and was deemed negligible or low risk by the Barwon Health Human Research Ethics Committee and therefore exempt from full committee review (project no: 17.119).

The MUD faecal sample was selected for spiking with the marker viruses. A non-spiked MUD sample acted as control for the optimisation of the best protocol for metagenomics of viruses. All faecal samples were then processed according to the optimised protocol without any spiking. The detailed steps of the final optimised protocol are given in the supplementary material (Supplementary material 3). However, the steps in the protocol are briefly described below.

### Real-time polymerase chain reaction (PCR) assays for marker viruses

We initially optimised real-time PCR assays for the marker viruses to detect and quantify them during the virome detection and characterisation protocol development. Virus-specific quantitative assays were either developed or retrieved from the literature (see Table S2 of Supplementary material 1 for details), and quantity required to spike the faecal samples assessed based on these assays. All quantitative assays for RNA viruses were carried out using Power SYBR® Green RNA-to-CT™ 1-Step kit (Thermofisher Scientific) using the following conditions: 48 °C for 30 min, 95 °C for 10 min, 40 cycles of 95 °C for 20 s, 54 °C for 30 s, 72 °C for 50 s, followed by a melt curve stage of 95 °C for 15 s, 60 °C for 1 min and 95 °C for 15 s and finally hold at 4°C. In case of BTV, an initial RNA denaturation step of 95 °C for 3 min followed by placing the sample in −20 °C cold ethanol was also carried out just before real-time PCR. All quantitative PCR assays for DNA viruses were carried out using PowerUp SYBR Green Master Mix (Thermofisher Scientific) using the following conditions: 95 °C for 10 min, 40 cycles of 95 °C for 20 s, 54 °C for 30 s, 72 °C for 50 s, followed by a melt curve stage of 95 °C for 15 s, 60 °C for 1 min and 95 °C for 15 s and finally hold at 4 °C. Quantitative PCR was performed on QuantStudio™ 6 Flex Real-Time PCR System (Applied Biosystems).

### Virus enrichment from samples

Removing other impurities such as host cells, bacteria, food particles and free nucleic acids from the faecal samples and enriching for virus particles followed by nucleic acid extraction is the first step in metagenomics of viruses. For this purpose, we tested different enrichment techniques using various biophysical methods^6,37,38^. Six method variations from A to F with a different combination of virus enrichment techniques using various biophysical methods were carried out (Fig. 1) on the spiked MUD faecal sample. We took the already tested protocol called the NetoVIR^6^ as our starting method for virus enrichment and designated it as ‘variation C’. We used the MUD faecal sample and incorporated two additional potential enrichment steps to this method, being detergent treatment (variation A & B) and ultracentrifugation (variation A, D & F) and also tested filtration using 0.45 µm filters (variation E & F) as described, but not recommended as optimum on their mock community, by the authors of NetoVIR^6^. This enabled us to evaluate, compare and develop an optimised protocol for virus community analysis from a biological sample. The combination of biophysical enrichment techniques that maintained the maximum number and levels of marker viruses was selected to proceed for cDNA synthesis, amplification, library preparation and NGS. The method which then gave the maximum number and obtained high-quality NGS reads of the marker viruses was then selected as the final method to conduct metagenomics of project samples for the detection of viruses.

#### Sample preparation

A 1:10 dilution of the bird and human faecal samples were prepared using sterile phosphate buffered saline (PBS). The samples were vortexed thoroughly prior to downstream processing.

#### Homogenization (During method optimisation stage)

A volume of 2 mL of the faecal samples were taken for variations involving ultracentrifugation, while for other variations only 500 µL was taken to maximise handling efficiency and protect virus nucleic acid during extraction. Homogenization was carried out using TissueLyser II homogenizer (Qiagen) at 25 Hz for 2 min.

#### Homogenization (After method optimisation)

A volume of 2 mL of the faecal samples were taken for homogenization. After homogenization, the sample was divided into two, one tube containing 1725µL which was processed according to Variation D and another containing 250 µL which was processed according to Variation C.

#### Detergent Treatment

To the homogenised sample of variation A and B, 0.25% (vol/vol) Nonidet P-40 detergent was added. It was centrifuged at 10,000 rpm for 30 min at 4 °C, and the supernatant was collected and processed downstream.

#### Centrifugation

The samples were centrifuged at 17,000 g for 3 min at room temperature, and the supernatant was collected for downstream processing.

#### Filtration

The samples were filtered through 0.8 µm polyethersulfone (PES) spin filters at 17,000 g for 1 min or until all of the sample had passed through the filter. The filtrate was taken and processed downstream.

During method optimisation stage, the spike MUD faecal sample under variation E and F were then filtered through 0.45 µm PES syringe filters, and the filtrate processed downstream.

#### Ultracentrifugation

The samples of variation A, D and F were then ultracentrifuged using a Beckman Coulter Airfuge Ultracentrifuge at 178,000 g for 1 hour (30 psi for 1 hour) at room temperature. The supernatant was discarded, and the pellet was suspended in 130 µL of sterile 1XPBS.

#### Nuclease treatment

To 130 µL of the sample, 7 µL of 20 × buffer (1 M Tris (pH8), 100 mM calcium chloride, 30 mM magnesium chloride), 2 µL of benzonase nuclease (Purity > 90%, Millipore) and 1 µL of micrococcal nuclease (New England Biolabs) was added. After mixing gently, the sample was incubated at 37 °C for 2 h. The nuclease reaction was stopped by adding 3 µL of 500 mM ethylenediaminetetraacetic acid (EDTA).

#### Virus nucleic acid extraction

Virus nucleic acids were extracted using the QIAamp Viral RNA Mini Kit (Qiagen), importantly without adding any carrier RNA. Both DNA and RNA virus nucleic acids were extracted using this kit. The quantity of the isolated nucleic acids was determined using Nanodrop and in selected cases a Bioanalyzer, however, the quantity of extracted nucleic acids was as expected found to be very low after the enrichment and nuclease treatment process.

### Next-generation sequencing

Virus nucleic acids extracted from the spiked MUD faecal sample that undergone variations C, D, E and F were processed downstream as these methods showed presence or detection of all spiked marker viruses. Other samples were also processed as described below. Extracted virus nucleic acids were subjected to cDNA synthesis, amplification and primer removal using SeqPlex RNA Amplification Kit (Sigma) from all the samples according to the kit instructions. An initial RNA denaturation step of 95 °C for 3 min followed by placing the sample in −20 °C cold ethanol was carried out just before cDNA synthesis to ensure the denaturation of the nucleic acids of any double stranded RNA viruses. The quantity and quality of the amplified product were checked using the Bioanalyzer.

Library preparation was carried out using Ion Plus Fragment Library Kit (Thermo Fisher Scientific), Ion Xpress Barcode Adapters 1–96 Kit (Thermo Fisher Scientific), Agencourt AMPure XP kit (Beckman Coulter) and Ion Library TaqMan™ Quantitation Kit (Thermo Fisher Scientific). A volume of 20 µL of the cleaned product was used for end repair using the end repair enzyme in Ion Plus Fragment Library Kit. The sample was then purified using Agencourt AMPure XP kit at a 1.8X sample to bead ratio and eluted in 25 µL of Low TE buffer. Following elution, barcoded libraries were prepared using Ion Plus Fragment Library Kit and Ion Xpress Barcode Adapters 1–96 Kit as per the kit instructions. A 1.5X sample to bead ratio of the Agencourt AMPure Kit was used to purify the library. Then it was eluted in 20 µL of Low TE buffer. Quantification of unamplified libraries was performed as per the protocol from Ion Library TaqMan™ Quantitation Kit using QuantStudio™ 6 Flex Real-Time PCR System. Based on the concentration estimated, the dilution that resulted in a concentration of ~100 pM was then calculated and made. Libraries were then pooled prior to loading onto Ion 530 Chips using the Ion Chef Instrument. Following template preparation, the chips were run on the Ion Torrent S5XL System (Thermo Fisher Scientific) as per company protocols. NGS and associated reactions were performed at the Geelong Centre for Emerging Infectious Diseases (GCEID), Geelong, Victoria, Australia. We ran NGS for the spiked MUD faecal sample for the variations C to F only once and generated around 3.4 million reads. Approximately 4 million reads were generated for the non-spiked MUD faecal samples on the first run during which it partly acted as a control, and 7 million reads generated for the MAD faecal sample. For further analysis, the MUD and MAD faecal samples were rerun, and about 9.5 and 7.1 million reads were generated respectively. However, NGS for the human samples were run only once as it was a demonstration of the optimised protocol only and about 7.1 million reads were generated.

### Next generation sequence analyses

Although the NGS data provided an insight into the virome of the bird faecal samples, we narrowed our focus to virus sequences that were somewhat similar to already known viruses, in most cases had good quality and quantity NGS reads, could serve as examples of the utility of the method, and/or were of interest from a virological point of view. All available virus sequences were downloaded from the NCBI GenBank genetic sequence database (May 2017), and a local BLAST database created. Bacterial plasmid sequences were downloaded from the NCBI Reference Sequence Database (RefSeq) repository and 16 S, 18 S, 23 S 25 S, 5 S and 5.8 S ribosomal sequences were downloaded from GenBank. These were used to create plasmid and ribosomal BLAST databases respectively. Virus sequences from the NCBI RefSeq database were downloaded and queried against the plasmid and ribosomal RNA databases using a megablast query and an e-value cut-off of 1 × 10^−10^. Any virus sequences which matched either plasmid or ribosomal sequences were removed, and the remaining virus sequences used to create a reference sequence virus BLAST database. The BLAST package version 2.6.0 from NCBI was used to create the local databases and perform the database queries.

A BLASTN query was performed on the reads from each sample against the initial virus database with an e-value cut-off score of 1 × 10^−3^. A BLASTN query against the virus reference sequence database was performed with an e-value cut-off of 1 × 10^−10^. A TBLASTX query against the virus reference sequence database was performed with an e-value cut-off of 1 × 10^−10^. BLAST query results files were converted into spreadsheet files, sorted by virus matches, and a list of potential virus targets created for each sample. The list was then manually inspected to identify viruses of interest.

Mapping of sequence reads was performed against reference genomes of the viruses of interest using TMAP plugin on the Ion Torrent server. The full or partial consensus sequences of the viruses were obtained from TMAP using Integrative Genomics Viewer software (IGV) (Broad Institute, MA, USA). Consensus sequences were generated from mapped sequences with a mapping quality score of 20 or higher and a coverage depth of at least 3.

AssemblerSPAdes 5.6.0 plugin on the Ion Torrent Server was used to generate contigs from the sequence reads of each sample. The contigs were queried against the virus reference sequence database using BLASTN and TBLASTX to identify virus sequences as described above. Contigs were then used as references in TMAP and trimmed to regions with a mapping quality of 20 or higher and a coverage depth of at least 3. The contigs of deltacoronavirus from the MAD faecal sample and selected viruses from the human samples were then selected for more in-depth analysis using MEGA software^63^. All the analysed sequences of the viruses found in MUD, MAD and human faecal samples are submitted to the NCBI GenBank database under the accession numbers MG991677-MG991679; MH000403-MH000428; MH013331-MH013339; MH028875-MH028887; MH085089-MH085101; MH107865- MH107867 and MH188072-MH188081.

### Phylogenetic analysis of selected virus sequences

Among the identified viruses, 10 viruses which provided good quality and coverage of the NGS reads mapped to NCBI virus genomes as references and also covered the different virus genome types (dsRNA, ssRNA, dsDNA and ssDNA viruses) were selected for more detailed analysis. For these viruses, NGS reads were further analysed using MEGA software^63^ as previously described in our laboratory^64,65^. To understand phylogenetic relationships among the virus sequences, they were compared to the NCBI database using nucleotide BLAST (Basic Local Alignment Search Tool)^66,67^ and aligned using Clustal-W^68^ and some cases using Clustal-W (Codons) or Muscle using the MEGA 6 or 7 software^63,69^. Then the sequences were manually trimmed to put them in frame. Maximum Likelihood phylogenetic analyses were conducted using the MEGA 6 or 7 software^63,69^, and the best model for generating a phylogenetic tree was determined (data not shown). The robustness of different nodes was assessed by bootstrap analysis using 1000 replicates for nucleotide alignments and 500 replicates for amino acid alignments. The distance between the branches based on the number of nucleotide differences was calculated using the same software.

### The “ribosomal activity microbiome” detection

Reads were uploaded and analysed on the ThermoFisher Ion Reporter microbiome software, and around 0.2–4.6% of all reads (depending on the sample and specific settings of the software) mapped to bacterial 16 S RNA sequences in the curated MicroSEQ® 16 S reference library v2013.1 and curated Greengenes v13.5 databases available in the Ion Reporter software. This gave a good insight into the active bacterial communities present in collected faecal samples.

### Data availability

All sequences analysed have been deposited in NCBI GenBank under accession numbers MG991677-MG991679; MH000403-MH000428; MH013331-MH013339; MH028875-MH028887; MH085089-MH085101; MH107865- MH107867 and MH188072-MH188081. Other datasets generated or analysed during the current study are available from the corresponding author on reasonable request.

## Electronic supplementary material

Supplementary Material
