# Supplementary material for: Metagenomics detection and characterisation of viruses in faecal samples from Australian wild birds
Source: Sci Rep. 2018 Jun 6;8:8686. doi: 10.1038/s41598-018-26851-1 (PMC5989203; doi:10.1038/s41598-018-26851-1)
Supplement: Supplementary file 1 — Supplementary Material [file 41598_2018_26851_MOESM1_ESM.pdf]

# Supplementary Material 1

## Metagenomics detection and characterisation of viruses in faecal samples from Australian wild birds

### Running Title of the Supplementary Material

Results of method optimisation for metagenomics of viruses.


### Authors

Jessy Vibin[1,2], Anthony Chamings[1,2], Fiona Collier[1,3], Marcel Klaassen[2], Tiffanie M Nelson[1,2], Soren Alexandersen*[1,2,3]

1 Geelong Centre for Emerging Infectious Diseases, Geelong, VIC 3220, Australia;

2 Deakin University, Geelong, Victoria, 3220, Australia;

3 Barwon Health, Geelong, VIC 3220, Australia;

*Corresponding Author: soren.alexandersen@deakin.edu.au

**Table S1. The marker viruses, their properties and source used for spiking the faecal samples.**

| Virus | Family | Characteristics | Sample type | Reference genome NCBI ID# |
|---|---|---|---|---|
| Bluetongue virus, Serotype 1 (BTV)* | *Reoviridae* | dsRNA virus; segmented linear genome; non-enveloped and icosahedral about 80 nm in diameter. | Tissue culture isolate | KM099511 |
| Bovine viral diarrhoea virus (BVDV)* | *Flaviviridae* | Monopartite, linear, ssRNA (+) genome; enveloped, spherical, about 50 nm in diameter. | Tissue culture isolate | NC 001461 |
| Porcine circovirus 2 (PCV2) * | *Circoviridae* | Monopartite, circular, ssDNA genome; non-enveloped, icosahedral about 20 nm in diameter. | Frozen infected swine spleen | NC 005148 |
| Infectious laryngotracheitis virus (ILTV)* | *Herpesviridae* | Monopartite, linear, dsDNA genome; enveloped, spherical to pleomorphic, 120-200 nm in diameter, nucleocapsid present | Pooled tracheal swabs in viral transport medium | NC006623 |
| Infectious bronchitis virus (IBV)+ | *Coronaviridae* | Monopartite, linear ssRNA (+) genome; enveloped, spherical, about 120 nm in diameter, nucleocapsid present | Tissue culture isolate | KF460437 |

*Source: Elizabeth Macarthur Agriculture Institute, NSW, Australia
+Source: AAHL, Victoria, Australia
#The reference genomes from NCBI listed served to map the NGS reads in IGV.

The table gives the details of the viruses that acted as marker viruses for protocol optimisation. Each of these viruses was isolated/present from/in different samples.

**Table S2. Primers for marker virus real-time PCR assays.**

| Virus | Primers | Reference |
|---|---|---|
| BTV | FP: GCGTTCGAAGTTTACATCAAT<br>RP: CAGTCATCTCTCTAGACACTCTATAATTACG | [1] |
| BVDV | FP: GRAGTCGTCARTGGTTCGAC<br>RP: TCAACTCCATGTGCCATGTAC | [2] |
| PCV2 | FP: TGGCCCGACGTATTCTGATT<br>RP: CAGCTGGGACAGCAGTTGAG | [3] |
| ILTV | FP: GCACGTCACGGACGATTGT<br>RP: GCCGCCTCGTCTTTTGC | Courtesy of Dr P. D. Kirkland, Elizabeth Macarthur Agricultural Institute, NSW |
| IBV | FP: CAAGCAGATGCTCAAGTGGA<br>RP: CTCACGCTGTTGTGACACCT | This study. NCBI reference sequence used DQ490206.1 |

The table displays the primers used for all the marker viruses and the origin of those primers. Both forward and reverse primers for each assay are shown. Primers for BTV, BVDV and PCV2 were taken from the literature. Primer sequences for ILTV were kindly provided by Dr P D Kirkland from the Elizabeth Macarthur Agricultural Institute. We designed the forward and reverse primers for IBV.

**Table S3. Ct values of marker viruses during the start of the protocol and after the nucleic acid extraction step.**

| Virus | Variation A | | Variation B | | Variation C | | Variation D | | Variation E | | Variation F | |
|---|---|---|---|---|---|---|---|---|---|---|---|---|
| Step | Start* | End# | Start | End | Start | End | Start | End | Start | End | Start | End |
| PCV2 | 16 | 16 | 16 | 12 | 17 | 13.5 | 17 | 16.5 | 17 | 15 | 19 | 14 |
| IBV | 28 | 39 | 28 | 36 | 28.5 | 26 | 28 | 26 | 27 | 24 | 27 | 21 |
| ILTV | 28 | 29 | 28 | 25 | 31 | 26 | 31 | 29 | 32 | 29 | 32 | 31 |
| BVDV | 31 | 30 | 31 | 35 | 31 | 28 | 31 | 27 | 30 | 28 | 30 | 28 |
| BTV | 29 | 28 | 29 | 29 | 30 | 26 | 30 | 24.5 | 31 | 26 | 31 | 25 |

*Nucleic acid isolated after homogenization of the spiked sample
# Nucleic acid isolated at the end of the variations

The table displays the Ct value of marker viruses, which were used as a reference for identifying how different virus enrichment combinations affected each virus type. For the “Start” Ct value, we took the spiked sample after homogenization and isolated the nucleic acid as per the described method. The “End” Ct value was determined using the nucleic acids isolated at the end stage of each variation. As there was an increase in more than 3Ct values, for one of the marker virus (IBV), we discontinued variation A and B. All other variations were processed for NGS.

**Table S4: Number of high-quality NGS reads generated for the marker viruses with a minimum mapping quality threshold of 20**

| No. of reads / Virus | Variation C | Variation D | Variation E | Variation F |
|---|---|---|---|---|
| BTV | 14 | 33 | 8 | 13 |
| BVDV | 13 | 50-100 | 16 | 5 |
| PCV2 | 50-150 | 50-150 | 50-150 | 50-150 |
| ILTV | 37 | 39 | 39 | 33 |
| IBV | 13 | 50-100 | 18 | 9 |

The table displays the number of high quality (Q20 or higher) NGS reads obtained for each marker virus from the spiked sample variations C to F. This was calculated manually from IGV. We used the reference genome mentioned in Table S1 for each of the marker viruses.

References


1. Shaw, A. E. *et al.* Development and initial evaluation of a real-time RT-PCR assay to detect bluetongue virus genome segment 1. *J. Virol. Methods* **145,** 115–126 (2007).

2. Hoffmann, B., Depner, K., Schirrmeier, H. & Beer, M. A universal heterologous internal control system for duplex real-time RT-PCR assays used in a detection system for pestiviruses. *J. Virol. Methods* **136,** 200–9 (2006).

3. Opriessnig, T. *et al.* Effect of vaccination with selective bacterins on conventional pigs infected with type 2 porcine circovirus. *Vet. Pathol.* **40,** 521–9 (2003).

# Supplementary Material 2

**Metagenomics detection and characterisation of viruses in faecal samples from Australian wild birds**

**Running Title of the Supplementary Material**

Representative phylogenetic trees of the consensus sequences generated and analysed using MEGA 6 or 7 software


**Authors**

Jessy Vibin[1,2], Anthony Chamings[1,2], Fiona Collier[1,3], Marcel Klaassen[2], Tiffanie M Nelson[1,2], Soren Alexandersen*[1,2,3]

1 Geelong Centre for Emerging Infectious Diseases, Geelong, VIC 3220, Australia;

2 Deakin University, Geelong, Victoria, 3220, Australia;

3 Barwon Health, Geelong, VIC 3220, Australia;

*Corresponding Author: soren.alexandersen@deakin.edu.au

1. **Avian Paramyxovirus 6 from MAD faecal sample**

| Figure and Table | Long Name (Format: Sample-virus-protein-length-quality-coverage-year) | Short name (Format: Sample-virus-protein-length) | Coverage | No. of nucleotides | Mapping quality threshold | NCBI accession number |
|---|---|---|---|---|---|---|
| S1.1 | MAD-Avian-paramyxovirus-6-nucleocapsid-protein-153nt-Q32-C-8-20-2016 | MAD-APMV6-NCP-153nt | 8-20 | 153 | 32 | MH000403 |
| S1.2 | MAD-Avian-paramyxovirus-6-nucleocapsid-protein-187nt-Q32-C-92-204-2016 | MAD-APMV6-NCP-187nt | 92-204 | 187 | 32 | MH000404 |
| S1.3 | MAD-Avian-paramyxovirus-6-nucleocapsid-protein-270nt-Q32-C-4-26-2016 | MAD-APMV6-NCP-270nt | 4-26 | 270 | 32 | MH000405 |
| S1.4 | MAD-Avian-paramyxovirus-6-nucleocapsid-protein-410nt-Q32-C-3-20-2016 | MAD-APMV6-NCP-410nt | 3-20 | 410 | 32 | MH000406 |
| S1.5 | MAD-Avian-paramyxovirus-6-phosphoprotein-211nt-Q32-C-6-72-2016 | MAD-APMV6-PP-211nt | 6-72 | 211 | 32 | MH000407 |
| S1.6 | MAD-Avian-paramyxovirus-6-phosphoprotein-167nt-Q32-C-3-17-2016 | MAD-APMV6-PP-167nt | 3-17 | 167 | 32 | MH000408 |
| S1.7 | MAD-Avian-paramyxovirus-6-matrix-protein-297nt-Q32-C-4-23-2016 | MAD-APMV6-MP-297nt | 4-23 | 297 | 32 | MH000409 |
| S1.8 | MAD-Avian-paramyxovirus-6-matrix-protein-241nt-Q32-C-3-24-2016 | MAD-APMV6-MP-241nt | 3-24 | 241 | 32 | MH000410 |
| S1.9 | MAD-Avian-paramyxovirus-6-matrix-protein-132nt-Q32-C-16-24-2016 | MAD-APMV6-MP-132nt | 16-24 | 132 | 32 | MH000411 |
| S1.10 | MAD-Avian-paramyxovirus-6- | MAD-APMV6-FP-276nt | 3-8 | 276 | 32 | MH000413 |

| | | | | | | |
|---|---|---|---|---|---|---|
| | fusion-protein-276nt-Q32-C-3-8-2016 | | | | | |
| S1.11 | MAD-Avian-paramyxovirus-6-small-hydrophobic-protein-351nt-Q32-C-4-19-2016 | MAD-APMV6-SHP-351nt | 4-19 | 351 | 32 | MH000414 |
| S1.12 | MAD-Avian-paramyxovirus-6-large-polymerase-protein-617nt-Q32-C-16-115-2016 | MAD-APMV6-Pol-617nt | 16-115 | 617 | 32 | MH000416 |
| S1.13 | MAD-Avian-paramyxovirus-6-large-polymerase-protein-496nt-Q32-C-11-53-2016 | MAD-APMV6-Pol-496nt | 11-53 | 496 | 32 | MH000417 |
| S1.14 | MAD-Avian-paramyxovirus-6-large-polymerase-protein-702nt-Q32-C-4-37-2016 | MAD-APMV6-Pol-702nt | 4-37 | 702 | 32 | MH000418 |
| S1.15 | MAD-Avian-paramyxovirus-6-large-polymerase-protein-199nt-Q32-C-3-19-2016 | MAD-APMV6-Pol-199nt | 3-19 | 199 | 32 | MH000420 |
| S1.16 | MAD-Avian-paramyxovirus-6-large-polymerase-protein-380nt-Q32-C-20-52-2016 | MAD-APMV6-Pol-380nt | 20-52 | 380 | 32 | MH000421 |
| S1.17 | MAD-Avian-paramyxovirus-6-large-polymerase-protein-204nt-Q32-C-6-19-2016 | MAD-APMV6-Pol-204nt | 6-19 | 204 | 32 | MH000422 |
| S1.18 | MAD-Avian-paramyxovirus-6-large-polymerase-protein-241nt-Q32-C-3-9-2016 | MAD-APMV6-Pol-241nt | 3-9 | 241 | 32 | MH000423 |
| S1.19 | MAD-Avian-paramyxovirus-6-large-polymerase-protein-284nt-Q32-C-4-18-2016 | MAD-APMV6-Pol-284nt | 4-18 | 284 | 32 | MH000424 |

| | | | | | | |
|---|---|---|---|---|---|---|
| S1.20 | MAD-Avian-paramyxovirus-6-large-polymerase-protein-383nt-Q32-C-21-187-2016 | MAD-APMV6-Pol-383nt | 21-187 | 383 | 32 | MH000425 |
| S1.21 | MAD-Avian-paramyxovirus-6-large-polymerase-protein-507nt-Q32-C-3-35-2016 | MAD-APMV6-Pol-507nt | 3-35 | 507 | 32 | MH000426 |
| S1.22 | MAD-Avian-paramyxovirus-6-large-polymerase-protein-343nt-Q32-C-3-19-2016 | MAD-APMV6-Pol-343nt | 3-19 | 343 | 32 | MH000427 |
| S1.23 | MAD-Avian-paramyxovirus-6-large-polymerase-protein-156nt-Q32-C-3-31-2016 | MAD-APMV6-Pol-156nt | 3-31 | 156 | 32 | MH000428 |
| NCBI sequences taken for phylogenetic analysis | | | | | | |
| Long Name | | Short Name (Format: NCBI accession number-virus-country/state) | Country of collection | Collection date | | |
| AB759118-Avian-paramyxovirus-6-viral-cRNA-complete-genome-strain:red-necked-stint/Japan/8KS0813/2008 | | AB759118-APMV6-JP | Japan | 2008 | | |
| GQ406232-Avian-paramyxovirus-6-strain-duck/Italy/4524-2/07-complete-genome | | GQ406232-APMV6-IT | Italy | 2007 | | |
| KP762799-Avian-paramyxovirus-6-isolate-red-crested-pochard/Balkhash/5842/2013-complete-genome | | KP762799-APMV6-KZ | Kazakhstan | 2013 | | |
| AY029299-Avian-paramyxovirus-6-complete-genome | | AY029299-APMV6-TW | Taiwan | - | | |
| KT962980-Avian-paramyxovirus-6-isolate-teal/Novosibirsk_region/455/2009-complete-genome | | KT962980-APMV6-RU | Russia | 2009 | | |
| JN571486-Avian-paramyxovirus-6-strain-APMV6/mallard/Belgium/12245/07-nucleoprotein(NP)-phosphoprotein(P)-matrix-protein(M)-fusion-protein(F)- | | JN571486-APMV6-BE | Belgium | 2007 | | |

| | | | |
|---|---|---|---|
| small-hydrophobic-protein(SH)-hemagglutinin-neuramis | | | |
| KF267717-Avian-paramyxovirus-6-isolate-mallard/Jilin/127/2011-complete-genome | KF267717-APMV6-CN | China | 2011 |

The table points towards the molecular phylogenetic analysis by Maximum Likelihood method of APMV6. The first half of the table provides details to identify the sample from which the virus was isolated, protein that encodes the consensus sequences that are being analysed, the length of the consensus sequences thus providing the depth of the gene being analysed, minimum mapping quality threshold used in IGV for the generation of the consensus sequences, the coverage of the consensus sequences from IGV, the year of collection of the sample and the NCBI accession number assigned to the particular consensus sequence. Corresponding short names have been used in the phylogenetic trees which contain the sample, virus, protein and the number of nucleotides. The second half of the table provides the details of the sequences that were used for the molecular phylogenetic analysis. They were found to be the most closely related sequences to the consensus sequences generated using BLASTN. Corresponding short names have been used in the phylogenetic trees which contain the NCBI accession number, virus and the country of collection. The collection date is given as retrieved from the corresponding NCBI nucleotide reference dataset which together with the country of collection can provide an insight into the possible evolution of the virus through the years.

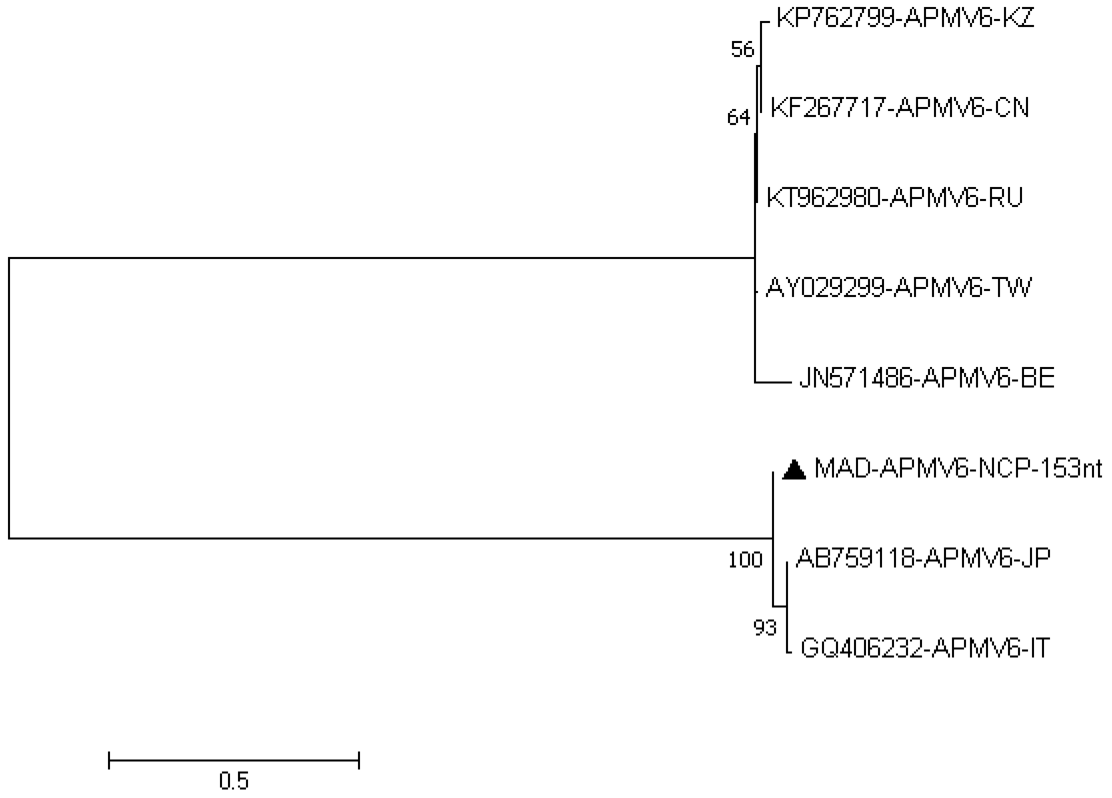


**Figure S1.1. Molecular Phylogenetic analysis by Maximum Likelihood method of APMV6 partial NCP gene**
The evolutionary history was inferred by using the Maximum Likelihood method based on the Kimura 2-parameter model[1]. The tree with the highest log likelihood (-427.58) is shown. The percentage of trees in which the associated taxa clustered together is shown next to the branches. Initial tree(s) for the heuristic search were obtained automatically by applying Neighbor-Join and BioNJ algorithms to a matrix of pairwise distances estimated using the Maximum Composite Likelihood (MCL) approach, and then selecting the topology with superior log likelihood value. A discrete Gamma distribution was used to model evolutionary rate differences among sites (5 categories (+G, parameter = 0.4716)). The rate variation model allowed for some sites to be evolutionarily invariable ([+I], 63.10% sites). The tree is drawn to scale, with branch lengths measured in the number of substitutions per site. The analysis involved 8 nucleotide sequences. Codon positions included were 1st+2nd+3rd+Noncoding. All positions containing gaps and missing data were eliminated. There were a total of 153 positions in the final dataset. Evolutionary analyses were conducted in MEGA7[2].

**Table S1.1. Estimates of Evolutionary Divergence between Sequences**
The number of base differences per sequence from between sequences are shown. The analysis involved 8 nucleotide sequences. Codon positions included were 1st+2nd+3rd+Noncoding. All positions containing gaps and missing data were eliminated. There were a total of 153 positions in the final dataset. Evolutionary analyses were conducted in MEGA7.

| **MAD-APMV6-NCP-153nt** | | | | | | | |
|---|---|---|---|---|---|---|---|
| AB759118-APMV6-JP | 4 | | | | | | |
| GQ406232-APMV6-IT | 5 | 1 | | | | | |
| KP762799-APMV6-KZ | 34 | 37 | 36 | | | | |
| AY029299-APMV6-TW | 35 | 38 | 37 | 5 | | | |
| KT962980-APMV6-RU | 36 | 39 | 38 | 3 | 2 | | |
| JN571486-APMV6-BE | 36 | 37 | 38 | 13 | 10 | 10 | |
| KF267717-APMV6-CN | 35 | 38 | 37 | 2 | 3 | 1 | 11 |

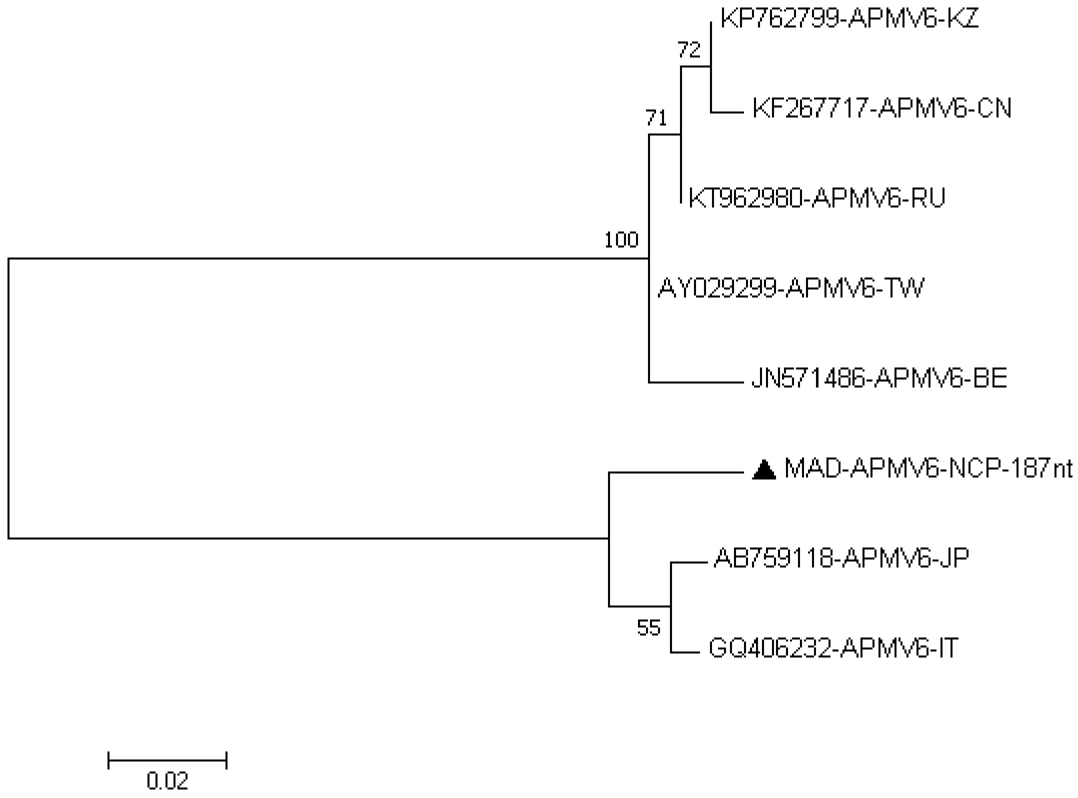


**Figure S1.2. Molecular Phylogenetic analysis by Maximum Likelihood method of APMV6 partial NCP gene**
The evolutionary history was inferred by using the Maximum Likelihood method based on the Kimura 2-parameter model[1]. The tree with the highest log likelihood (-466.22) is shown. The percentage of trees in which the associated taxa clustered together is shown next to the branches. Initial tree(s) for the heuristic search were obtained automatically by applying Neighbor-Join and BioNJ algorithms to a matrix of pairwise distances estimated using the Maximum Composite Likelihood (MCL) approach, and then selecting the topology with superior log likelihood value. The tree is drawn to scale, with branch lengths measured in the number of substitutions per site. The analysis involved 8 nucleotide sequences. Codon positions included were 1st+2nd+3rd+Noncoding. All positions containing gaps and missing data were eliminated. There were a total of 187 positions in the final dataset. Evolutionary analyses were conducted in MEGA7[2].

**Table S1.2: Estimates of Evolutionary Divergence between Sequences**
The number of base differences per sequence from between sequences are shown. The analysis involved 8 nucleotide sequences. Codon positions included were 1st+2nd+3rd+Noncoding. All positions containing gaps and missing data were eliminated. There were a total of 187 positions in the final dataset. Evolutionary analyses were conducted in MEGA7.

| **MAD-APMV6-NCP-187nt** | | | | | | | |
|---|---|---|---|---|---|---|---|
| AB759118-APMV6-JP | 7 | | | | | | |
| GQ406232-APMV6-IT | 7 | 2 | | | | | |
| KP762799-APMV6-KZ | 39 | 37 | 36 | | | | |
| AY029299-APMV6-TW | 37 | 35 | 34 | 2 | | | |
| KT962980-APMV6-RU | 38 | 36 | 35 | 1 | 1 | | |
| JN571486-APMV6-BE | 38 | 38 | 37 | 5 | 3 | 4 | |
| KF267717-APMV6-CN | 40 | 38 | 37 | 1 | 3 | 2 | 6 |

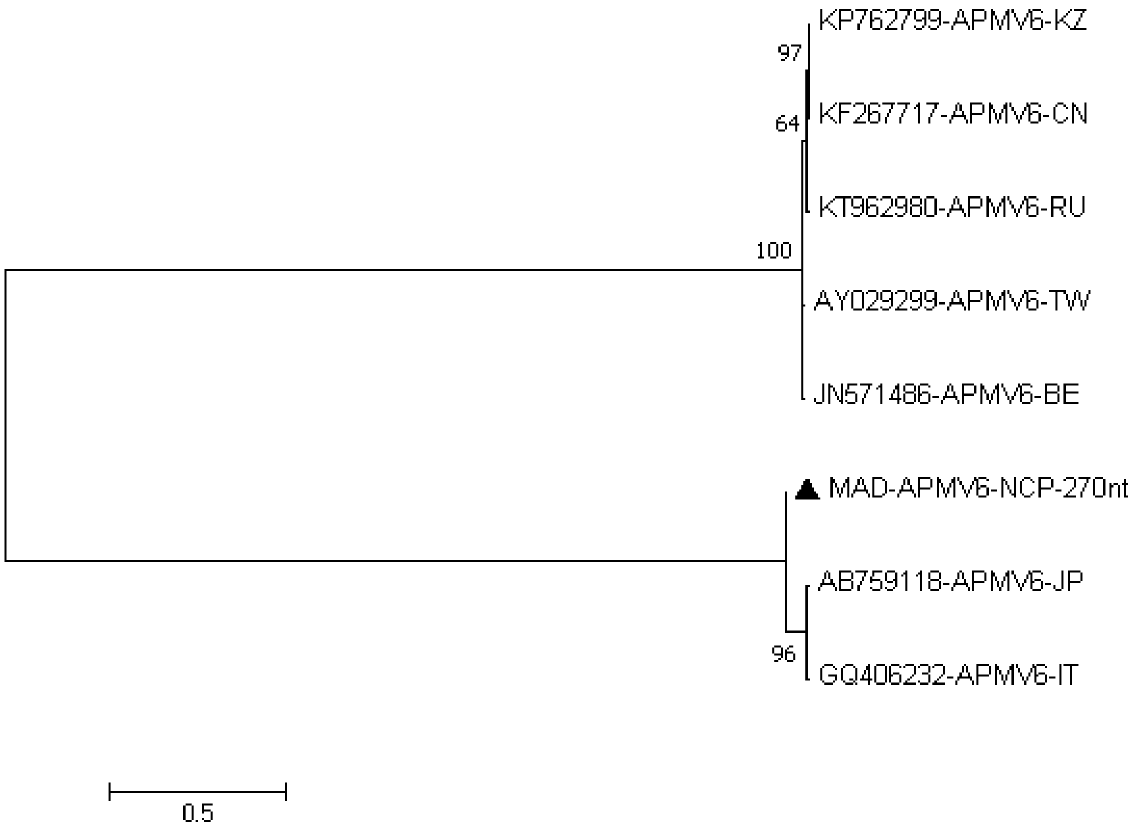


**Figure S1.3. Molecular Phylogenetic analysis by Maximum Likelihood method of APMV6 partial NCP gene**
The evolutionary history was inferred by using the Maximum Likelihood method based on the Kimura 2-parameter model[1]. The tree with the highest log likelihood (-733.10) is shown. The percentage of trees in which the associated taxa clustered together is shown next to the branches. Initial tree(s) for the heuristic search were obtained automatically by applying Neighbor-Join and BioNJ algorithms to a matrix of pairwise distances estimated using the Maximum Composite Likelihood (MCL) approach, and then selecting the topology with superior log likelihood value. A discrete Gamma distribution was used to model evolutionary rate differences among sites (5 categories (+G, parameter = 0.4529)). The rate variation model allowed for some sites to be evolutionarily invariable ([+I], 64.25% sites). The tree is drawn to scale, with branch lengths measured in the number of substitutions per site. The analysis involved 8 nucleotide sequences. Codon positions included were 1st+2nd+3rd+Noncoding. All positions containing gaps and missing data were eliminated. There were a total of 270 positions in the final dataset. Evolutionary analyses were conducted in MEGA7[2].

**Table S1.3. Estimates of Evolutionary Divergence between Sequences**
The number of base differences per sequence from between sequences are shown. The analysis involved 8 nucleotide sequences. Codon positions included were 1st+2nd+3rd+Noncoding. All positions containing gaps and missing data were eliminated. There were a total of 270 positions in the final dataset. Evolutionary analyses were conducted in MEGA7.

| **MAD-APMV6-NCP-270nt** | | | | | | | |
|---|---|---|---|---|---|---|---|
| AB759118-APMV6-JP | 15 | | | | | | |
| GQ406232-APMV6-IT | 15 | 2 | | | | | |
| KP762799-APMV6-KZ | 64 | 66 | 67 | | | | |
| AY029299-APMV6-TW | 62 | 64 | 65 | 5 | | | |
| KT962980-APMV6-RU | 63 | 63 | 64 | 4 | 5 | | |
| JN571486-APMV6-BE | 61 | 63 | 64 | 5 | 2 | 5 | |
| KF267717-APMV6-CN | 64 | 66 | 67 | 0 | 5 | 4 | 5 |

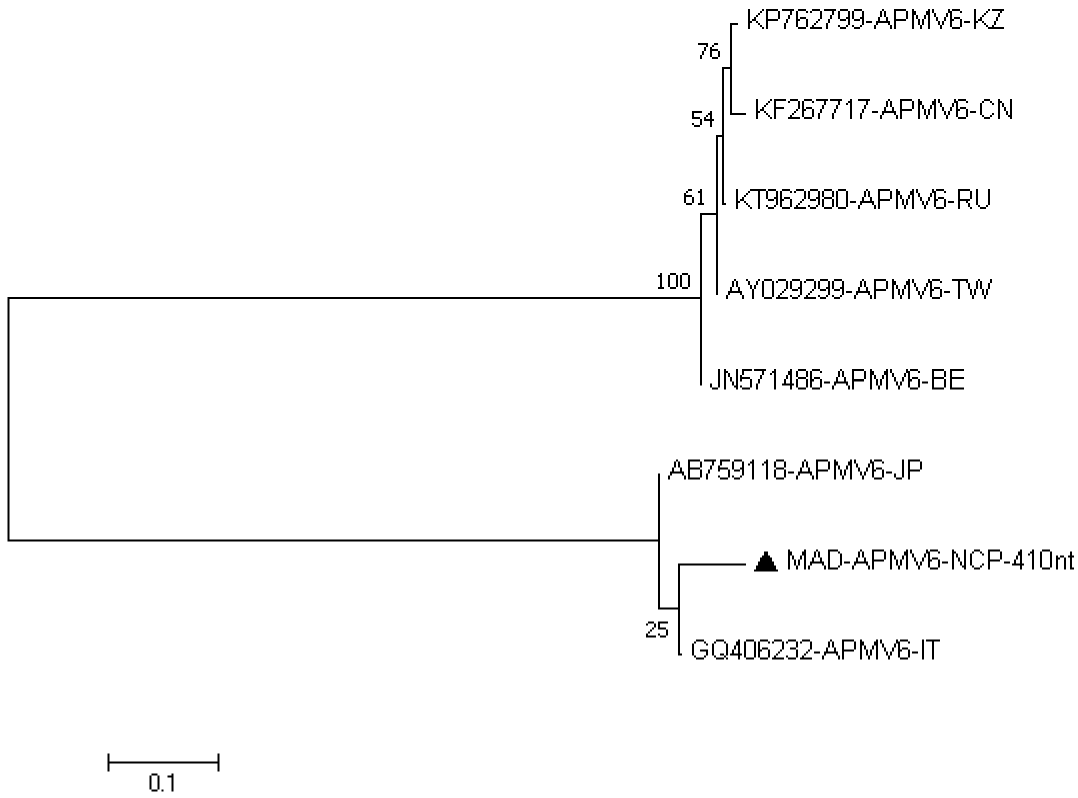


**Figure S1.4. Molecular Phylogenetic analysis by Maximum Likelihood method of APMV6 partial NCP gene**
The evolutionary history was inferred by using the Maximum Likelihood method based on the Kimura 2-parameter model[1]. The tree with the highest log likelihood (-1125.76) is shown. The percentage of trees in which the associated taxa clustered together is shown next to the branches. Initial tree(s) for the heuristic search were obtained automatically by applying Neighbor-Join and BioNJ algorithms to a matrix of pairwise distances estimated using the Maximum Composite Likelihood (MCL) approach, and then selecting the topology with superior log likelihood value. A discrete Gamma distribution was used to model evolutionary rate differences among sites (5 categories (+G, parameter = 0.3877)). The tree is drawn to scale, with branch lengths measured in the number of substitutions per site. The analysis involved 8 nucleotide sequences. Codon positions included were 1st+2nd+3rd+Noncoding. All positions containing gaps and missing data were eliminated. There were a total of 410 positions in the final dataset. Evolutionary analyses were conducted in MEGA7[2].

**Table S1.4. Estimates of Evolutionary Divergence between Sequences**
The number of base differences per sequence from between sequences are shown. The analysis involved 8 nucleotide sequences. Codon positions included were 1st+2nd+3rd+Noncoding. All positions containing gaps and missing data were eliminated. There were a total of 410 positions in the final dataset. Evolutionary analyses were conducted in MEGA7.

| **MAD-APMV6-NCP-410nt** | | | | | | | |
|---|---|---|---|---|---|---|---|
| AB759118-APMV6-JP | 26 | | | | | | |
| GQ406232-APMV6-IT | 22 | 8 | | | | | |
| KP762799-APMV6-KZ | 100 | 92 | 94 | | | | |
| AY029299-APMV6-TW | 98 | 94 | 96 | 7 | | | |
| KT962980-APMV6-RU | 100 | 92 | 94 | 6 | 3 | | |
| JN571486-APMV6-BE | 95 | 92 | 94 | 13 | 6 | 9 | |
| KF267717-APMV6-CN | 102 | 94 | 96 | 7 | 10 | 9 | 16 |

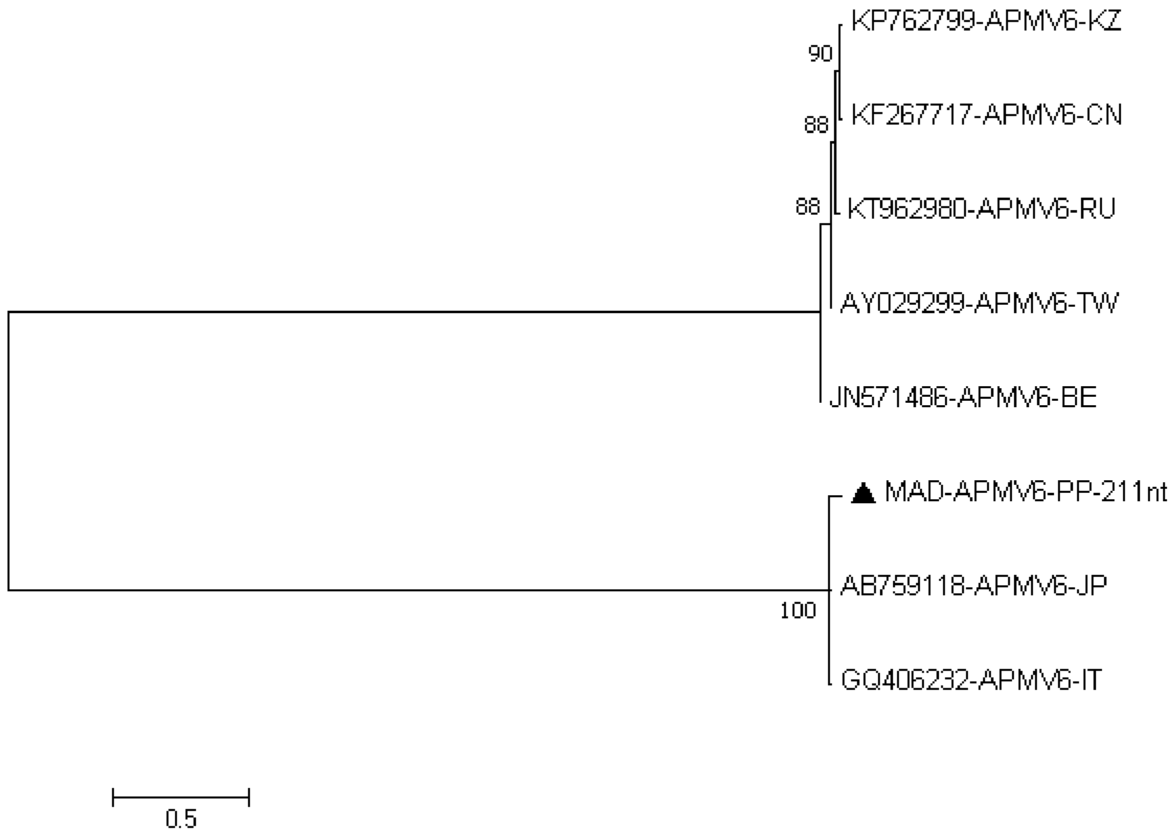


**Figure S1.5. Molecular Phylogenetic analysis by Maximum Likelihood method of APMV6 partial PP gene**
The evolutionary history was inferred by using the Maximum Likelihood method based on the Tamura-Nei model[3]. The tree with the highest log likelihood (-682.39) is shown. The percentage of trees in which the associated taxa clustered together is shown next to the branches. Initial tree(s) for the heuristic search were obtained automatically by applying Neighbor-Join and BioNJ algorithms to a matrix of pairwise distances estimated using the Maximum Composite Likelihood (MCL) approach, and then selecting the topology with superior log likelihood value. The rate variation model allowed for some sites to be evolutionarily invariable ([+I], 57.35% sites). The tree is drawn to scale, with branch lengths measured in the number of substitutions per site. The analysis involved 8 nucleotide sequences. Codon positions included were 1st+2nd+3rd+Noncoding. All positions containing gaps and missing data were eliminated. There were a total of 211 positions in the final dataset. Evolutionary analyses were conducted in MEGA7[2].

**Table S1.5. Estimates of Evolutionary Divergence between Sequences**
The number of base differences per sequence from between sequences are shown. The analysis involved 8 nucleotide sequences. Codon positions included were 1st+2nd+3rd+Noncoding. All positions containing gaps and missing data were eliminated. There were a total of 211 positions in the final dataset. Evolutionary analyses were conducted in MEGA7.

| **MAD-APMV6-PP-211nt** | | | | | | | |
|---|---|---|---|---|---|---|---|
| AB759118-APMV6-JP | 10 | | | | | | |
| GQ406232-APMV6-IT | 10 | 4 | | | | | |
| KP762799-APMV6-KZ | 63 | 65 | 64 | | | | |
| AY029299-APMV6-TW | 62 | 62 | 61 | 7 | | | |
| KT962980-APMV6-RU | 64 | 64 | 63 | 6 | 5 | | |
| JN571486-APMV6-BE | 60 | 58 | 61 | 13 | 7 | 12 | |
| KF267717-APMV6-CN | 63 | 65 | 64 | 4 | 7 | 6 | 13 |

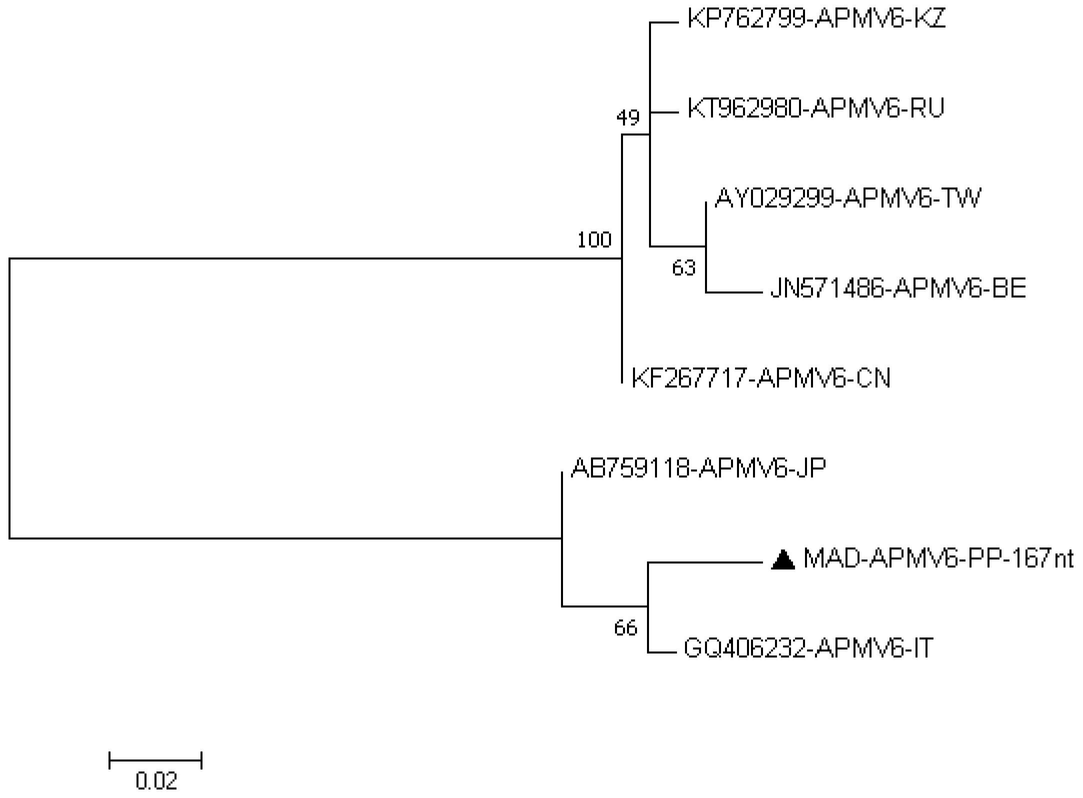


**Figure S1.6. Molecular Phylogenetic analysis by Maximum Likelihood method of APMV6 partial PP gene**
The evolutionary history was inferred by using the Maximum Likelihood method based on the Kimura 2-parameter model[1]. The tree with the highest log likelihood (-436.43) is shown. The percentage of trees in which the associated taxa clustered together is shown next to the branches. Initial tree(s) for the heuristic search were obtained automatically by applying Neighbor-Join and BioNJ algorithms to a matrix of pairwise distances estimated using the Maximum Composite Likelihood (MCL) approach, and then selecting the topology with superior log likelihood value. The tree is drawn to scale, with branch lengths measured in the number of substitutions per site. The analysis involved 8 nucleotide sequences. Codon positions included were 1st+2nd+3rd+Noncoding. All positions containing gaps and missing data were eliminated. There were a total of 167 positions in the final dataset. Evolutionary analyses were conducted in MEGA7[2].

**Table S1.6. Estimates of Evolutionary Divergence between Sequences**
The number of base differences per sequence from between sequences are shown. The analysis involved 8 nucleotide sequences. Codon positions included were 1st+2nd+3rd+Noncoding. All positions containing gaps and missing data were eliminated. There were a total of 167 positions in the final dataset. Evolutionary analyses were conducted in MEGA7.

| **MAD-APMV6-PP-167nt** | | | | | | | |
|---|---|---|---|---|---|---|---|
| AB759118-APMV6-JP | 7 | | | | | | |
| GQ406232-APMV6-IT | 5 | 4 | | | | | |
| KP762799-APMV6-KZ | 38 | 36 | 38 | | | | |
| AY029299-APMV6-TW | 38 | 36 | 38 | 3 | | | |
| KT962980-APMV6-RU | 38 | 36 | 38 | 2 | 3 | | |
| JN571486-APMV6-BE | 38 | 36 | 38 | 5 | 2 | 5 | |
| KF267717-APMV6-CN | 36 | 34 | 36 | 2 | 3 | 2 | 5 |

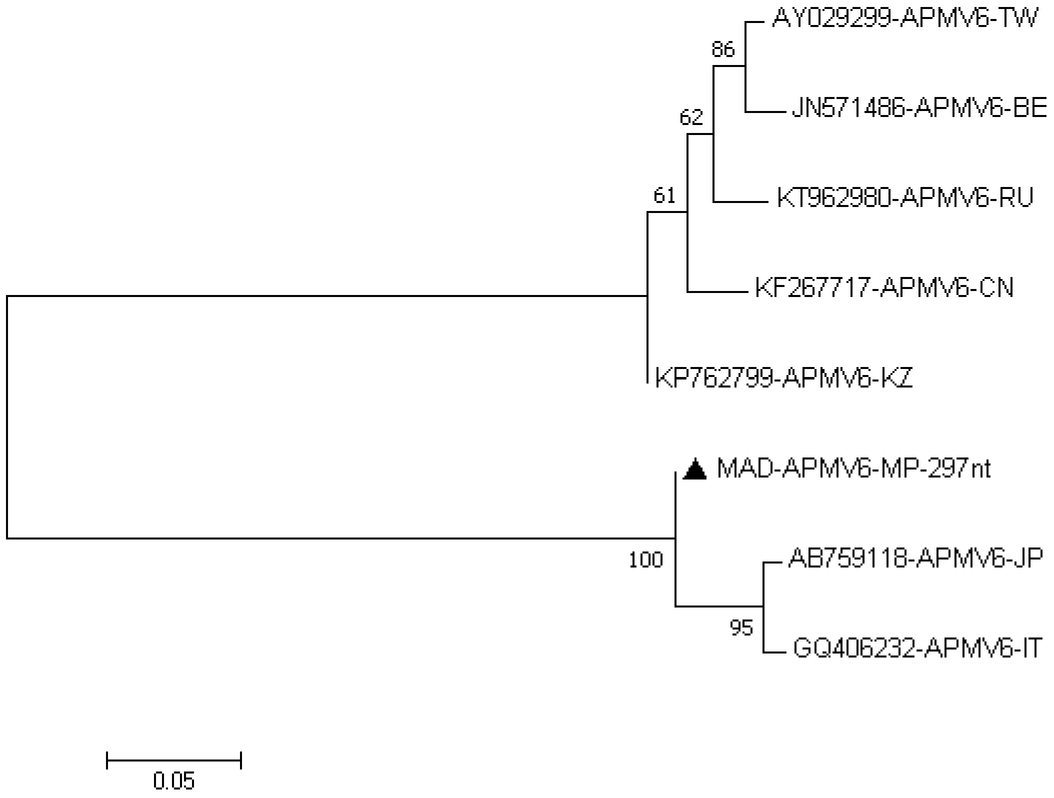


**Figure S1.7. Molecular Phylogenetic analysis by Maximum Likelihood method of APMV6 partial MP gene**
The evolutionary history was inferred by using the Maximum Likelihood method based on the Kimura 2-parameter model[1]. The tree with the highest log likelihood (-855.36) is shown. The percentage of trees in which the associated taxa clustered together is shown next to the branches. Initial tree(s) for the heuristic search were obtained automatically by applying Neighbor-Join and BioNJ algorithms to a matrix of pairwise distances estimated using the Maximum Composite Likelihood (MCL) approach, and then selecting the topology with superior log likelihood value. A discrete Gamma distribution was used to model evolutionary rate differences among sites (5 categories (+G, parameter = 0.3705)). The tree is drawn to scale, with branch lengths measured in the number of substitutions per site. The analysis involved 8 nucleotide sequences. Codon positions included were 1st+2nd+3rd+Noncoding. All positions containing gaps and missing data were eliminated. There were a total of 297 positions in the final dataset. Evolutionary analyses were conducted in MEGA7[2].

**Table S1.7. Estimates of Evolutionary Divergence between Sequences**
The number of base differences per sequence from between sequences are shown. The analysis involved 8 nucleotide sequences. Codon positions included were 1st+2nd+3rd+Noncoding. All positions containing gaps and missing data were eliminated. There were a total of 297 positions in the final dataset. Evolutionary analyses were conducted in MEGA7.

| | | | | | | | |
|---|---|---|---|---|---|---|---|
| **MAD-APMV6-MP-297nt** | | | | | | | |
| AB759118-APMV6-JP | 10 | | | | | | |
| GQ406232-APMV6-IT | 11 | 4 | | | | | |
| KP762799-APMV6-KZ | 59 | 63 | 64 | | | | |
| AY029299-APMV6-TW | 68 | 68 | 71 | 11 | | | |
| KT962980-APMV6-RU | 64 | 64 | 67 | 12 | 10 | | |
| JN571486-APMV6-BE | 67 | 67 | 70 | 12 | 6 | 12 | |
| KF267717-APMV6-CN | 66 | 66 | 69 | 10 | 13 | 13 | 15 |

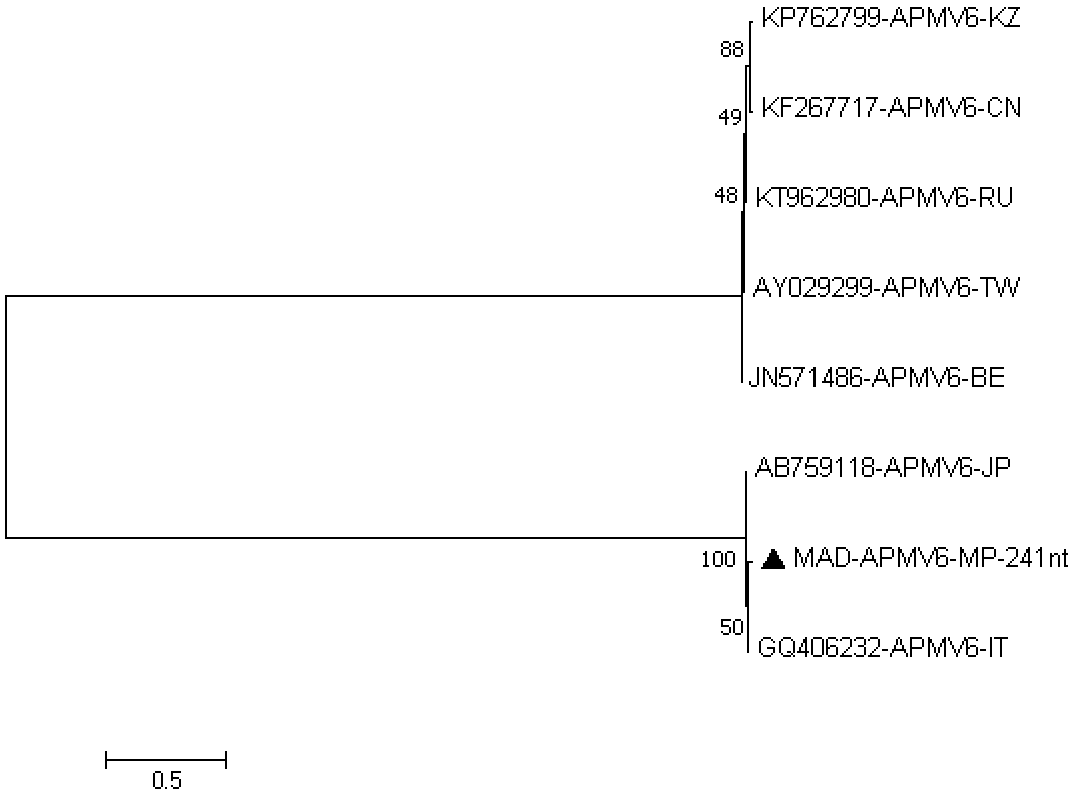


**Figure S1.8. Molecular Phylogenetic analysis by Maximum Likelihood method of APMV6 partial MP gene**
The evolutionary history was inferred by using the Maximum Likelihood method based on the Kimura 2-parameter model[1]. The tree with the highest log likelihood (-656.08) is shown. The percentage of trees in which the associated taxa clustered together is shown next to the branches. Initial tree(s) for the heuristic search were obtained automatically by applying Neighbor-Join and BioNJ algorithms to a matrix of pairwise distances estimated using the Maximum Composite Likelihood (MCL) approach, and then selecting the topology with superior log likelihood value. A discrete Gamma distribution was used to model evolutionary rate differences among sites (5 categories (+G, parameter = 0.5414)). The rate variation model allowed for some sites to be evolutionarily invariable ([+I], 0.00% sites). The tree is drawn to scale, with branch lengths measured in the number of substitutions per site. The analysis involved 8 nucleotide sequences. Codon positions included were 1st+2nd+3rd+Noncoding. All positions containing gaps and missing data were eliminated. There were a total of 241 positions in the final dataset. Evolutionary analyses were conducted in MEGA7[2].

**Table S1.8. Estimates of Evolutionary Divergence between Sequences**
The number of base differences per sequence from between sequences are shown. The analysis involved 8 nucleotide sequences. Codon positions included were 1st+2nd+3rd+Noncoding. All positions containing gaps and missing data were eliminated. There were a total of 241 positions in the final dataset. Evolutionary analyses were conducted in MEGA7.

| | | | | | | | |
|---|---|---|---|---|---|---|---|
| **MAD-APMV6-MP-241nt** | | | | | | | |
| AB759118-APMV6-JP | 7 | | | | | | |
| GQ406232-APMV6-IT | 4 | 3 | | | | | |
| KP762799-APMV6-KZ | 61 | 60 | 61 | | | | |
| AY029299-APMV6-TW | 60 | 59 | 60 | 8 | | | |
| KT962980-APMV6-RU | 61 | 60 | 61 | 6 | 2 | | |
| JN571486-APMV6-BE | 59 | 58 | 59 | 9 | 3 | 5 | |
| KF267717-APMV6-CN | 62 | 61 | 62 | 3 | 7 | 5 | 10 |

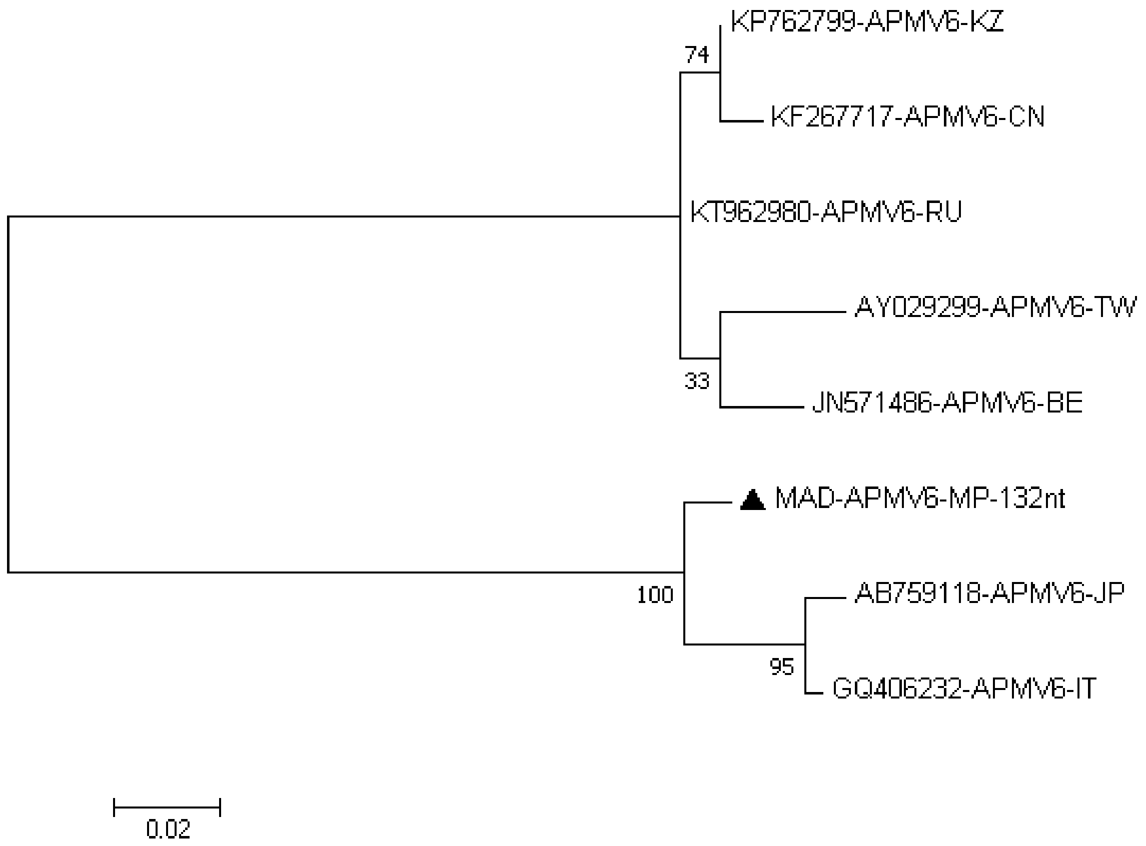


**Figure S1.9. Molecular Phylogenetic analysis by Maximum Likelihood method of APMV6 partial MP gene**
The evolutionary history was inferred by using the Maximum Likelihood method based on the Kimura 2-parameter model[1]. The tree with the highest log likelihood (-357.56) is shown. The percentage of trees in which the associated taxa clustered together is shown next to the branches. Initial tree(s) for the heuristic search were obtained automatically by applying Neighbor-Join and BioNJ algorithms to a matrix of pairwise distances estimated using the Maximum Composite Likelihood (MCL) approach, and then selecting the topology with superior log likelihood value. The tree is drawn to scale, with branch lengths measured in the number of substitutions per site. The analysis involved 8 nucleotide sequences. Codon positions included were 1st+2nd+3rd+Noncoding. All positions containing gaps and missing data were eliminated. There were a total of 132 positions in the final dataset. Evolutionary analyses were conducted in MEGA7[2].

**Table S1.9. Estimates of Evolutionary Divergence between Sequences**
The number of base differences per sequence from between sequences are shown. The analysis involved 8 nucleotide sequences. Codon positions included were 1st+2nd+3rd+Noncoding. All positions containing gaps and missing data were eliminated. There were a total of 132 positions in the final dataset. Evolutionary analyses were conducted in MEGA7.

| **MAD-APMV6-MP-132nt** | | | | | | | |
|---|---|---|---|---|---|---|---|
| AB759118-APMV6-JP | 5 | | | | | | |
| GQ406232-APMV6-IT | 4 | 1 | | | | | |
| KP762799-APMV6-KZ | 29 | 30 | 30 | | | | |
| AY029299-APMV6-TW | 30 | 31 | 31 | 5 | | | |
| KT962980-APMV6-RU | 28 | 29 | 29 | 1 | 4 | | |
| JN571486-APMV6-BE | 29 | 30 | 30 | 4 | 5 | 3 | |
| KF267717-APMV6-CN | 30 | 31 | 31 | 1 | 6 | 2 | 5 |

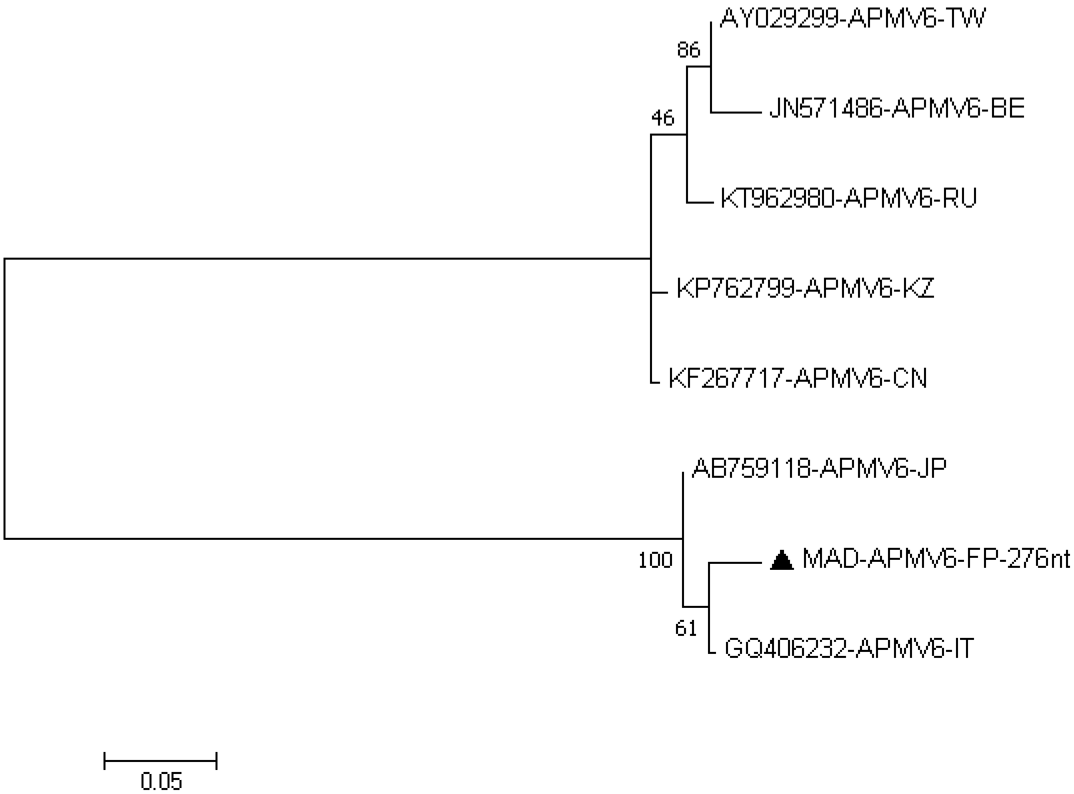


**Figure S1.10. Molecular Phylogenetic analysis by Maximum Likelihood method of APMV6 partial FP gene**
The evolutionary history was inferred by using the Maximum Likelihood method based on the Tamura 3-parameter model[4]. The tree with the highest log likelihood (-744.95) is shown. The percentage of trees in which the associated taxa clustered together is shown next to the branches. Initial tree(s) for the heuristic search were obtained automatically by applying Neighbor-Join and BioNJ algorithms to a matrix of pairwise distances estimated using the Maximum Composite Likelihood (MCL) approach, and then selecting the topology with superior log likelihood value. A discrete Gamma distribution was used to model evolutionary rate differences among sites (5 categories (+G, parameter = 0.4490)). The tree is drawn to scale, with branch lengths measured in the number of substitutions per site. The analysis involved 8 nucleotide sequences. Codon positions included were 1st+2nd+3rd+Noncoding. All positions containing gaps and missing data were eliminated. There were a total of 276 positions in the final dataset. Evolutionary analyses were conducted in MEGA7[2].

**Table S1.10. Estimates of Evolutionary Divergence between Sequences**
The number of base differences per sequence from between sequences are shown. The analysis involved 8 nucleotide sequences. Codon positions included were 1st+2nd+3rd+Noncoding. All positions containing gaps and missing data were eliminated. There were a total of 276 positions in the final dataset. Evolutionary analyses were conducted in MEGA7.

| | | | | | | | |
|---|---|---|---|---|---|---|---|
| **MAD-APMV6-FP-276nt** | | | | | | | |
| AB759118-APMV6-JP | 9 | | | | | | |
| GQ406232-APMV6-IT | 7 | 4 | | | | | |
| KP762799-APMV6-KZ | 63 | 61 | 63 | | | | |
| AY029299-APMV6-TW | 63 | 61 | 63 | 9 | | | |
| KT962980-APMV6-RU | 61 | 59 | 61 | 9 | 6 | | |
| JN571486-APMV6-BE | 66 | 62 | 66 | 15 | 6 | 12 | |
| KF267717-APMV6-CN | 63 | 61 | 63 | 3 | 8 | 8 | 14 |

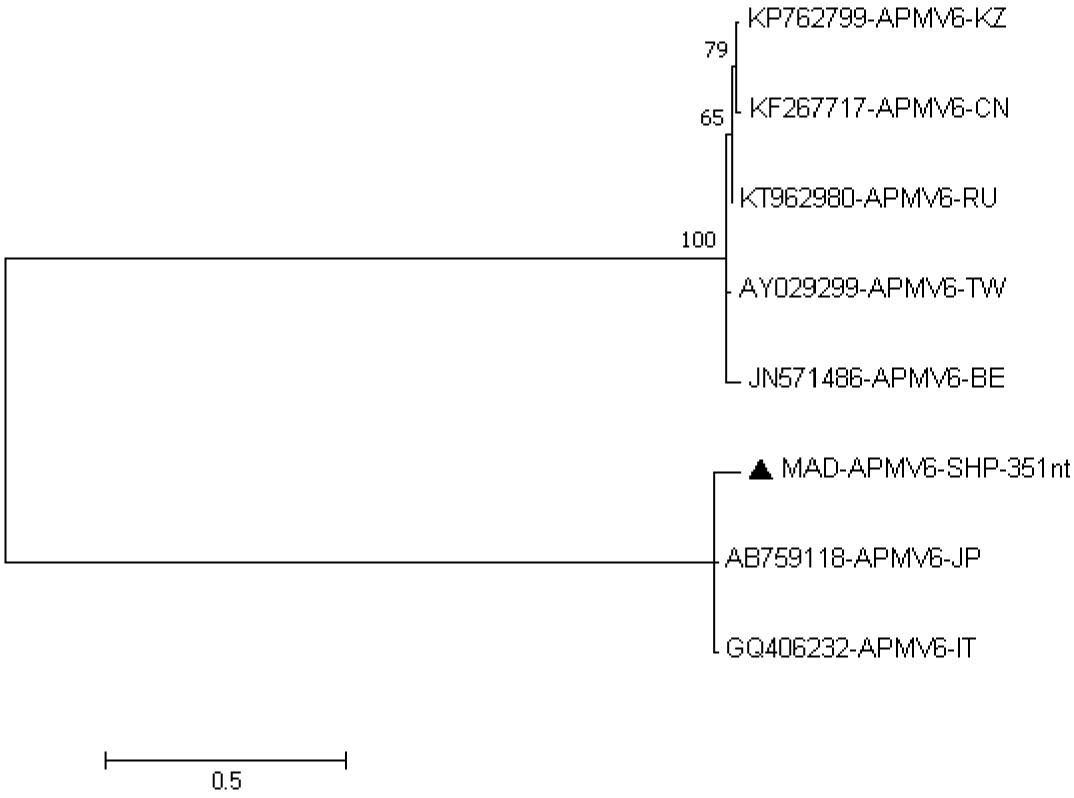


**FigureS 1.11. Molecular Phylogenetic analysis by Maximum Likelihood method of APMV6 partial SHP gene**
The evolutionary history was inferred by using the Maximum Likelihood method based on the Kimura 2-parameter model[1]. The tree with the highest log likelihood (-1186.85) is shown. The percentage of trees in which the associated taxa clustered together is shown next to the branches. Initial tree(s) for the heuristic search were obtained automatically by applying Neighbor-Join and BioNJ algorithms to a matrix of pairwise distances estimated using the Maximum Composite Likelihood (MCL) approach, and then selecting the topology with superior log likelihood value. The rate variation model allowed for some sites to be evolutionarily invariable ([+I], 33.12% sites). The tree is drawn to scale, with branch lengths measured in the number of substitutions per site. The analysis involved 8 nucleotide sequences. Codon positions included were 1st+2nd+3rd+Noncoding. All positions containing gaps and missing data were eliminated. There were a total of 344 positions in the final dataset. Evolutionary analyses were conducted in MEGA7[2].

**Table S1.11. Estimates of Evolutionary Divergence between Sequences**
The number of base differences per sequence from between sequences are shown. The analysis involved 8 nucleotide sequences. Codon positions included were 1st+2nd+3rd+Noncoding. All positions containing gaps and missing data were eliminated. There were a total of 344 positions in the final dataset. Evolutionary analyses were conducted in MEGA7.

| **MAD-APMV6-SHP-351nt** | | | | | | | |
|---|---|---|---|---|---|---|---|
| AB759118-APMV6-JP | 19 | | | | | | |
| GQ406232-APMV6-IT | 19 | 4 | | | | | |
| KP762799-APMV6-KZ | 166 | 155 | 155 | | | | |
| AY029299-APMV6-TW | 164 | 153 | 153 | 10 | | | |
| KT962980-APMV6-RU | 166 | 155 | 155 | 5 | 5 | | |
| JN571486-APMV6-BE | 167 | 156 | 156 | 15 | 11 | 12 | |
| KF267717-APMV6-CN | 165 | 154 | 154 | 5 | 11 | 6 | 15 |

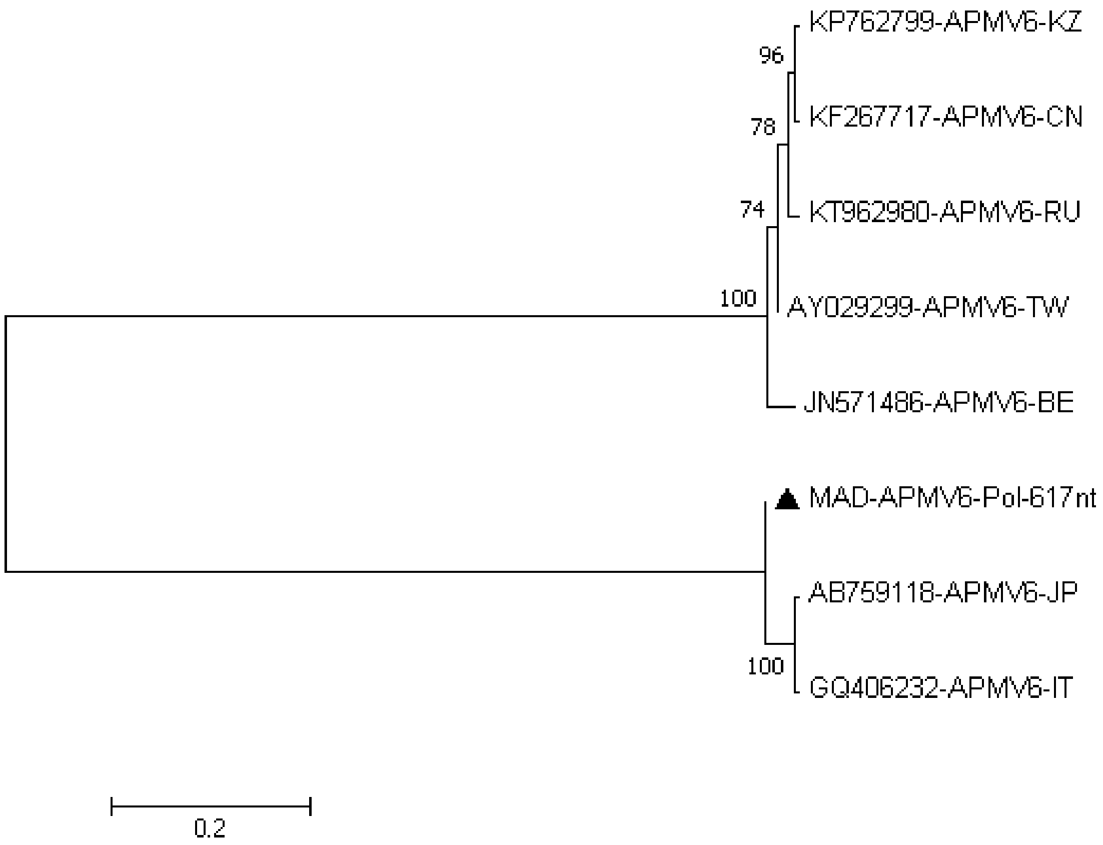


**Figure S1.12: Molecular Phylogenetic analysis by Maximum Likelihood method of APMV6 partial Pol gene**
The evolutionary history was inferred by using the Maximum Likelihood method based on the Kimura 2-parameter model[1]. The tree with the highest log likelihood (-1870.04) is shown. The percentage of trees in which the associated taxa clustered together is shown next to the branches. Initial tree(s) for the heuristic search were obtained automatically by applying Neighbor-Join and BioNJ algorithms to a matrix of pairwise distances estimated using the Maximum Composite Likelihood (MCL) approach, and then selecting the topology with superior log likelihood value. The rate variation model allowed for some sites to be evolutionarily invariable ([+I], 32.66% sites). The tree is drawn to scale, with branch lengths measured in the number of substitutions per site. The analysis involved 8 nucleotide sequences. All positions containing gaps and missing data were eliminated. There were a total of 617 positions in the final dataset. Evolutionary analyses were conducted in MEGA7[2].

**Table S1.12: Estimates of Evolutionary Divergence between Sequences**
The number of base differences per sequence from between sequences are shown. The analysis involved 8 nucleotide sequences. Codon positions included were 1st+2nd+3rd+Noncoding. All positions containing gaps and missing data were eliminated. There were a total of 617 positions in the final dataset. Evolutionary analyses were conducted in MEGA7.

| **MAD-APMV6-Pol-617nt** | | | | | | | |
|---|---|---|---|---|---|---|---|
| AB759118-APMV6-JP | 20 | | | | | | |
| GQ406232-APMV6-IT | 20 | 6 | | | | | |
| KP762799-APMV6-KZ | 188 | 188 | 190 | | | | |
| AY029299-APMV6-TW | 193 | 191 | 193 | 14 | | | |
| KT962980-APMV6-RU | 190 | 188 | 190 | 14 | 14 | | |
| JN571486-APMV6-BE | 193 | 193 | 193 | 33 | 23 | 33 | |
| KF267717-APMV6-CN | 188 | 188 | 190 | 6 | 14 | 14 | 33 |

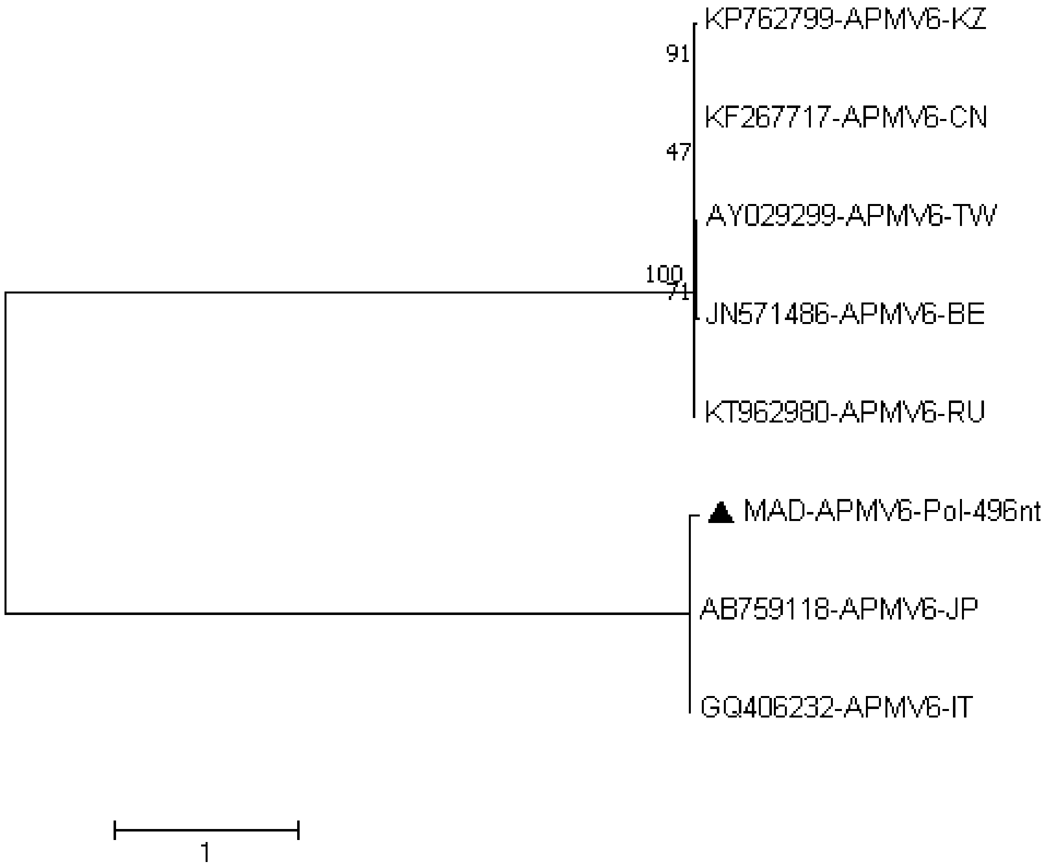


**Figure S1.13: Molecular Phylogenetic analysis by Maximum Likelihood method of APMV6 partial Pol gene**
The evolutionary history was inferred by using the Maximum Likelihood method based on the Kimura 2-parameter model[1]. The tree with the highest log likelihood (-1392.34) is shown. The percentage of trees in which the associated taxa clustered together is shown next to the branches. Initial tree(s) for the heuristic search were obtained automatically by applying Neighbor-Join and BioNJ algorithms to a matrix of pairwise distances estimated using the Maximum Composite Likelihood (MCL) approach, and then selecting the topology with superior log likelihood value. The rate variation model allowed for some sites to be evolutionarily invariable ([+I], 35.38% sites). The tree is drawn to scale, with branch lengths measured in the number of substitutions per site. The analysis involved 8 nucleotide sequences. All positions containing gaps and missing data were eliminated. There were a total of 496 positions in the final dataset. Evolutionary analyses were conducted in MEGA7[2].

**Table S1.13: Estimates of Evolutionary Divergence between Sequences**
The number of base differences per sequence from between sequences are shown. The analysis involved 8 nucleotide sequences. Codon positions included were 1st+2nd+3rd+Noncoding. All positions containing gaps and missing data were eliminated. There were a total of 496 positions in the final dataset. Evolutionary analyses were conducted in MEGA7.

| **MAD-APMV6-Pol-496nt** | | | | | | | |
|---|---|---|---|---|---|---|---|
| AB759118-APMV6-JP | 22 | | | | | | |
| GQ406232-APMV6-IT | 20 | 2 | | | | | |
| KP762799-APMV6-KZ | 131 | 129 | 127 | | | | |
| AY029299-APMV6-TW | 131 | 129 | 127 | 8 | | | |
| KT962980-APMV6-RU | 129 | 127 | 125 | 7 | 7 | | |
| JN571486-APMV6-BE | 134 | 132 | 130 | 13 | 7 | 10 | |
| KF267717-APMV6-CN | 131 | 129 | 127 | 2 | 6 | 5 | 11 |

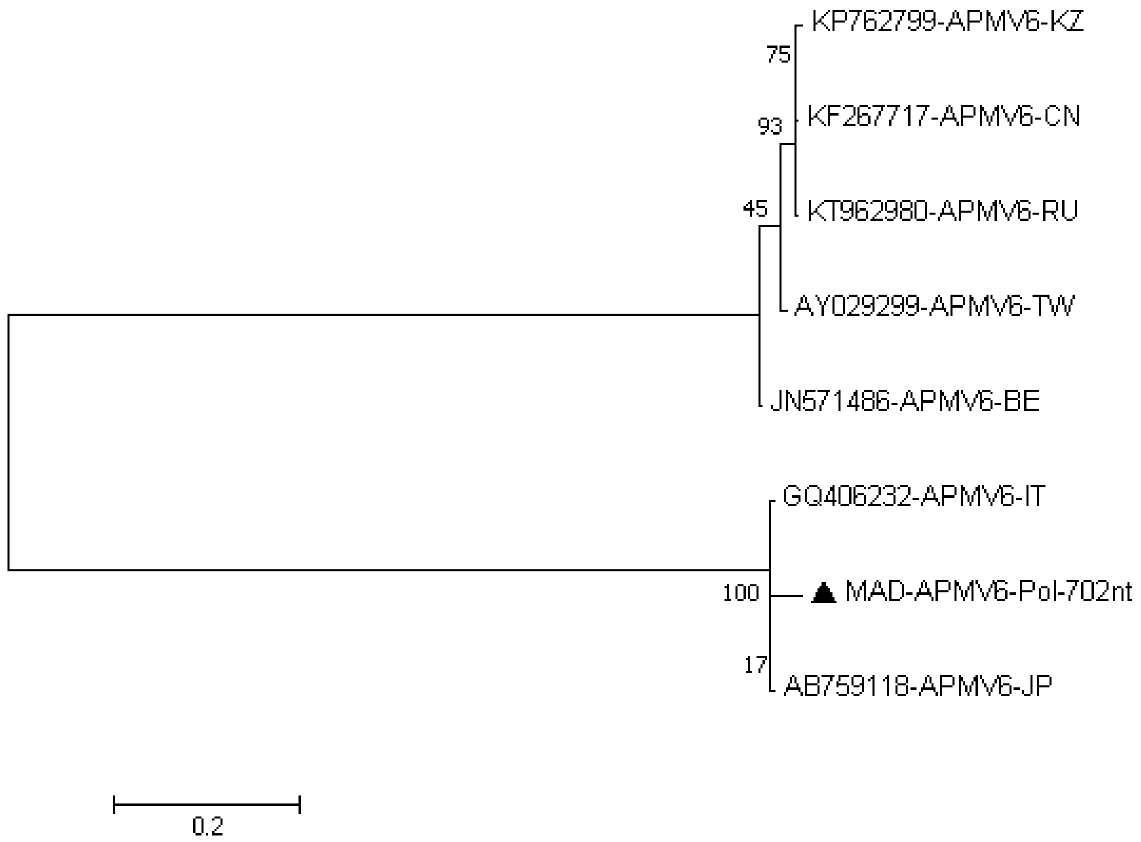


**Figure S1.14: Molecular Phylogenetic analysis by Maximum Likelihood method of APMV6 partial Pol gene**
The evolutionary history was inferred by using the Maximum Likelihood method based on the Kimura 2-parameter model[1]. The tree with the highest log likelihood (-1924.36) is shown. The percentage of trees in which the associated taxa clustered together is shown next to the branches. Initial tree(s) for the heuristic search were obtained automatically by applying Neighbor-Join and BioNJ algorithms to a matrix of pairwise distances estimated using the Maximum Composite Likelihood (MCL) approach, and then selecting the topology with superior log likelihood value. A discrete Gamma distribution was used to model evolutionary rate differences among sites (5 categories (+G, parameter = 0.4751)). The tree is drawn to scale, with branch lengths measured in the number of substitutions per site. The analysis involved 8 nucleotide sequences. All positions containing gaps and missing data were eliminated. There were a total of 702 positions in the final dataset. Evolutionary analyses were conducted in MEGA7[2].

**Table S1.14: Estimates of Evolutionary Divergence between Sequences**
The number of base differences per sequence from between sequences are shown. The analysis involved 8 nucleotide sequences. Codon positions included were 1st+2nd+3rd+Noncoding. All positions containing gaps and missing data were eliminated. There were a total of 702 positions in the final dataset. Evolutionary analyses were conducted in MEGA7.

| **MAD-APMV6-Pol-702nt** | | | | | | | |
|---|---|---|---|---|---|---|---|
| AB759118-APMV6-JP | 24 | | | | | | |
| GQ406232-APMV6-IT | 24 | 6 | | | | | |
| KP762799-APMV6-KZ | 176 | 181 | 178 | | | | |
| AY029299-APMV6-TW | 173 | 171 | 170 | 21 | | | |
| KT962980-APMV6-RU | 170 | 175 | 172 | 8 | 17 | | |
| JN571486-APMV6-BE | 177 | 175 | 176 | 29 | 20 | 27 | |
| KF267717-APMV6-CN | 171 | 176 | 173 | 7 | 18 | 5 | 28 |

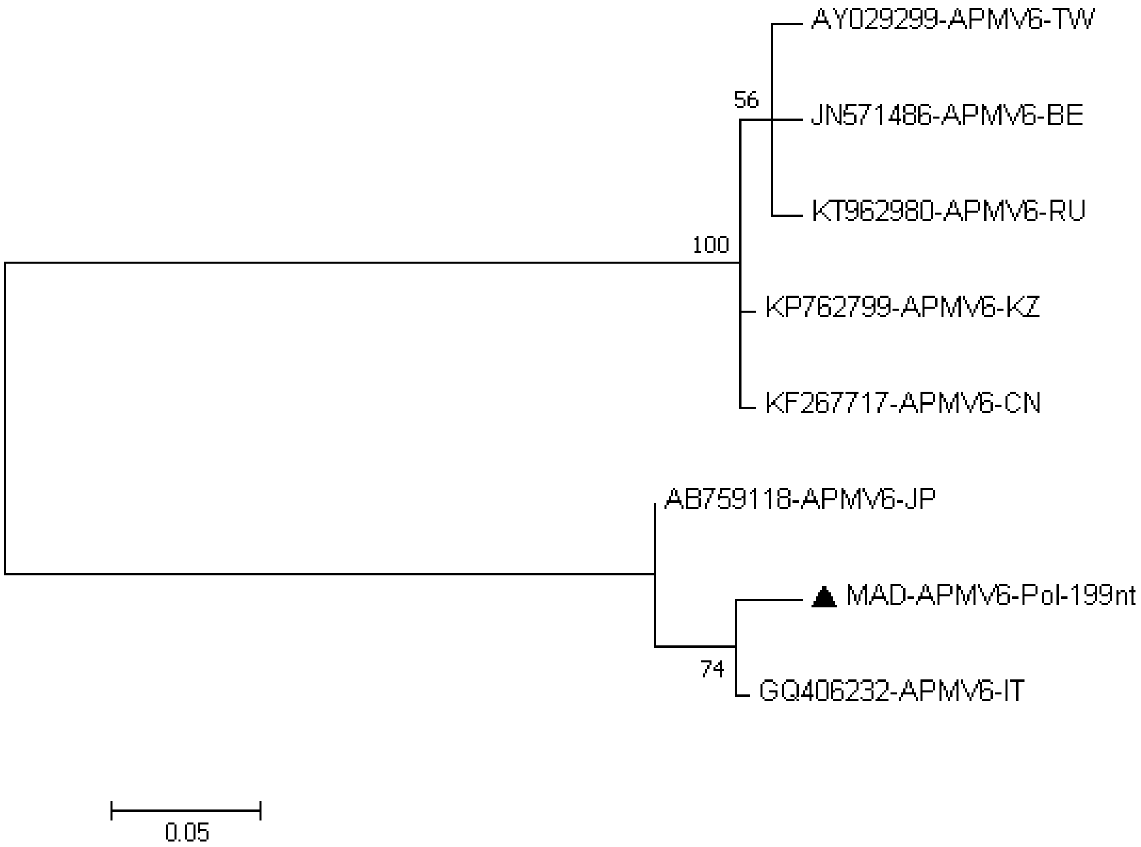


**Figure S1.15: Molecular Phylogenetic analysis by Maximum Likelihood method of APMV6 partial Pol gene**
The evolutionary history was inferred by using the Maximum Likelihood method based on the Kimura 2-parameter model[1]. The tree with the highest log likelihood (-572.97) is shown. The percentage of trees in which the associated taxa clustered together is shown next to the branches. Initial tree(s) for the heuristic search were obtained automatically by applying Neighbor-Join and BioNJ algorithms to a matrix of pairwise distances estimated using the Maximum Composite Likelihood (MCL) approach, and then selecting the topology with superior log likelihood value. The rate variation model allowed for some sites to be evolutionarily invariable ([+I], 36.68% sites). The tree is drawn to scale, with branch lengths measured in the number of substitutions per site. The analysis involved 8 nucleotide sequences. All positions containing gaps and missing data were eliminated. There were a total of 199 positions in the final dataset. Evolutionary analyses were conducted in MEGA7[2].

**Table S1.15: Estimates of Evolutionary Divergence between Sequences**
The number of base differences per sequence from between sequences are shown. The analysis involved 8 nucleotide sequences. Codon positions included were 1st+2nd+3rd+Noncoding. All positions containing gaps and missing data were eliminated. There were a total of 199 positions in the final dataset. Evolutionary analyses were conducted in MEGA7.

| **MAD-APMV6-Pol-199nt** | | | | | | | |
|---|---|---|---|---|---|---|---|
| AB759118-APMV6-JP | 9 | | | | | | |
| GQ406232-APMV6-IT | 5 | 6 | | | | | |
| KP762799-APMV6-KZ | 46 | 43 | 47 | | | | |
| AY029299-APMV6-TW | 48 | 47 | 47 | 5 | | | |
| KT962980-APMV6-RU | 46 | 45 | 47 | 5 | 4 | | |
| JN571486-APMV6-BE | 46 | 45 | 47 | 5 | 4 | 4 | |
| KF267717-APMV6-CN | 47 | 44 | 48 | 2 | 5 | 5 | 5 |

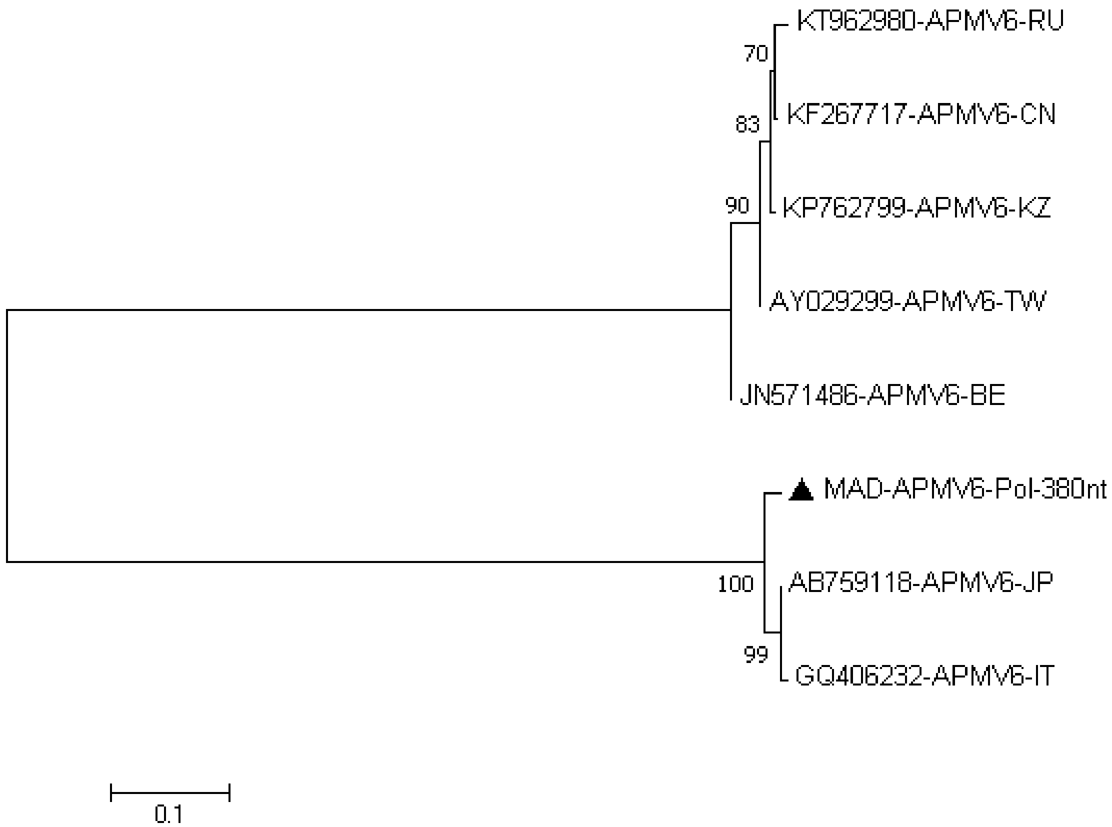


**Figure S1.16: Molecular Phylogenetic analysis by Maximum Likelihood method of APMV6 partial Pol gene**
The evolutionary history was inferred by using the Maximum Likelihood method based on the Kimura 2-parameter model[1]. The tree with the highest log likelihood (-1025.97) is shown. The percentage of trees in which the associated taxa clustered together is shown next to the branches. Initial tree(s) for the heuristic search were obtained automatically by applying Neighbor-Join and BioNJ algorithms to a matrix of pairwise distances estimated using the Maximum Composite Likelihood (MCL) approach, and then selecting the topology with superior log likelihood value. The rate variation model allowed for some sites to be evolutionarily invariable ([+I], 36.45% sites). The tree is drawn to scale, with branch lengths measured in the number of substitutions per site. The analysis involved 8 nucleotide sequences. All positions containing gaps and missing data were eliminated. There were a total of 380 positions in the final dataset. Evolutionary analyses were conducted in MEGA7[2].

**Table S1.16: Estimates of Evolutionary Divergence between Sequences**
The number of base differences per sequence from between sequences are shown. The analysis involved 8 nucleotide sequences. Codon positions included were 1st+2nd+3rd+Noncoding. All positions containing gaps and missing data were eliminated. There were a total of 380 positions in the final dataset. Evolutionary analyses were conducted in MEGA7.

| **MAD-APMV6-Pol-380nt** | | | | | | | |
|---|---|---|---|---|---|---|---|
| AB759118-APMV6-JP | 9 | | | | | | |
| GQ406232-APMV6-IT | 11 | 2 | | | | | |
| KP762799-APMV6-KZ | 89 | 91 | 92 | | | | |
| AY029299-APMV6-TW | 91 | 92 | 93 | 4 | | | |
| KT962980-APMV6-RU | 93 | 95 | 96 | 6 | 8 | | |
| JN571486-APMV6-BE | 88 | 89 | 90 | 13 | 9 | 15 | |
| KF267717-APMV6-CN | 92 | 94 | 95 | 3 | 5 | 5 | 14 |

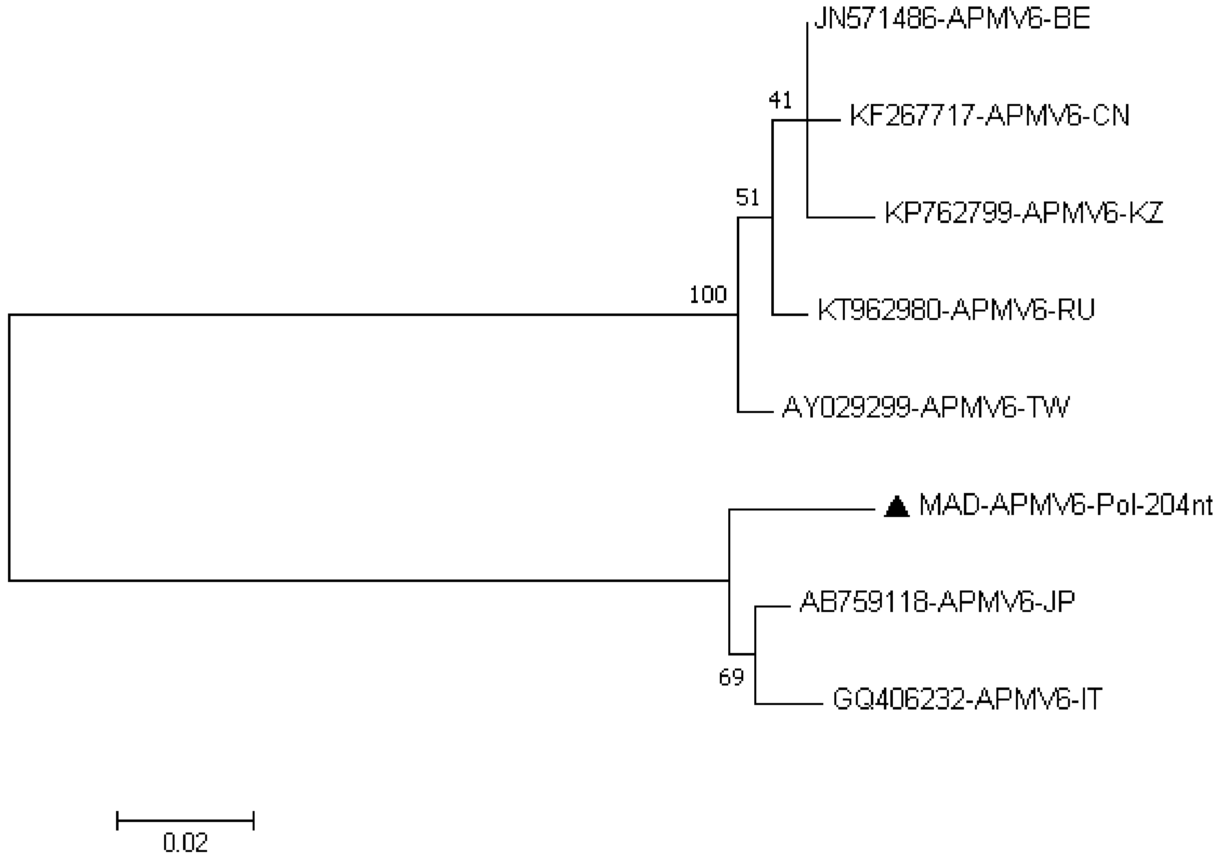


**Figure S1.17: Molecular Phylogenetic analysis by Maximum Likelihood method of APMV6 partial Pol gene**
The evolutionary history was inferred by using the Maximum Likelihood method based on the Kimura 2-parameter model[1]. The tree with the highest log likelihood (-509.35) is shown. The percentage of trees in which the associated taxa clustered together is shown next to the branches. Initial tree(s) for the heuristic search were obtained automatically by applying Neighbor-Join and BioNJ algorithms to a matrix of pairwise distances estimated using the Maximum Composite Likelihood (MCL) approach, and then selecting the topology with superior log likelihood value. The tree is drawn to scale, with branch lengths measured in the number of substitutions per site. The analysis involved 8 nucleotide sequences. All positions containing gaps and missing data were eliminated. There were a total of 204 positions in the final dataset. Evolutionary analyses were conducted in MEGA7[2].

**Table S1.17: Estimates of Evolutionary Divergence between Sequences**
The number of base differences per sequence from between sequences are shown. The analysis involved 8 nucleotide sequences. Codon positions included were 1st+2nd+3rd+Noncoding. All positions containing gaps and missing data were eliminated. There were a total of 204 positions in the final dataset. Evolutionary analyses were conducted in MEGA7.

| **MAD-APMV6-Pol-204nt** | | | | | | | |
|---|---|---|---|---|---|---|---|
| AB759118-APMV6-JP | 6 | | | | | | |
| GQ406232-APMV6-IT | 7 | 3 | | | | | |
| KP762799-APMV6-KZ | 40 | 39 | 39 | | | | |
| AY029299-APMV6-TW | 40 | 39 | 37 | 5 | | | |
| KT962980-APMV6-RU | 41 | 40 | 40 | 4 | 3 | | |
| JN571486-APMV6-BE | 40 | 39 | 39 | 2 | 3 | 2 | |
| KF267717-APMV6-CN | 41 | 40 | 40 | 3 | 4 | 3 | 1 |

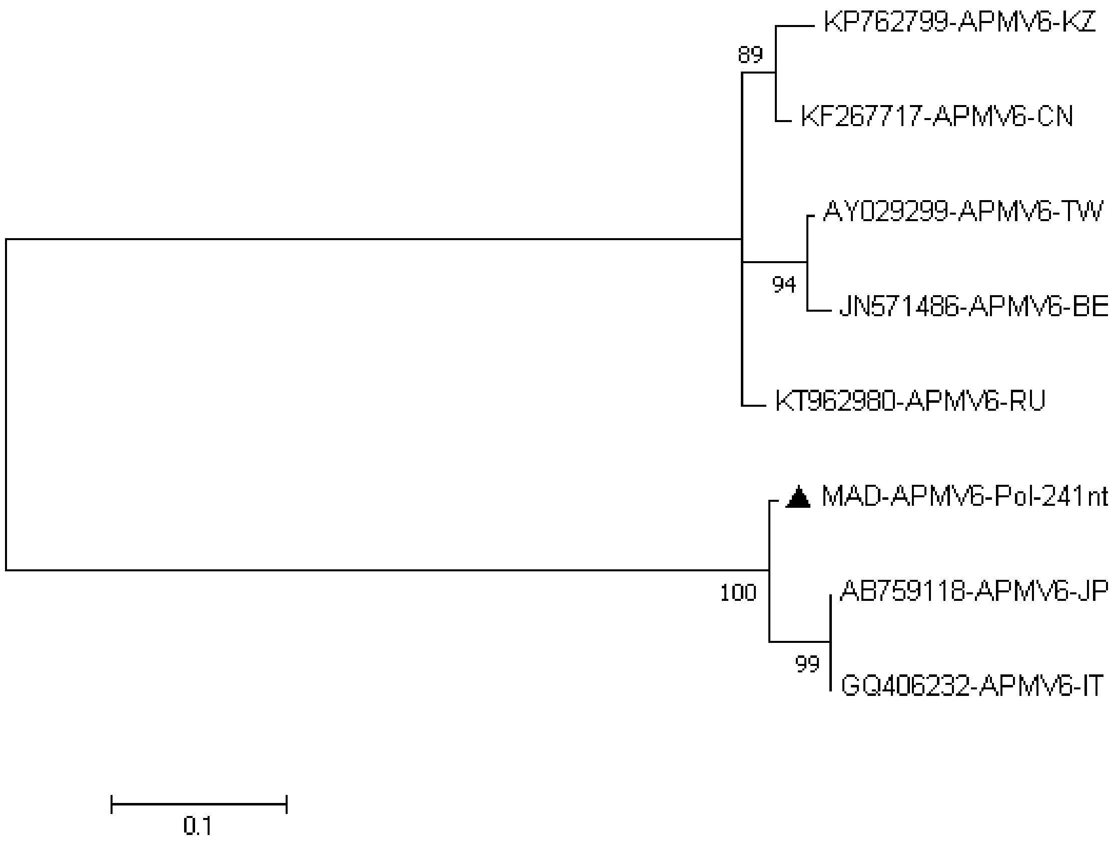


**Figure S1.18: Molecular Phylogenetic analysis by Maximum Likelihood method of APMV6 partial Pol gene**
The evolutionary history was inferred by using the Maximum Likelihood method based on the Kimura 2-parameter model[1]. The tree with the highest log likelihood (-720.37) is shown. The percentage of trees in which the associated taxa clustered together is shown next to the branches. Initial tree(s) for the heuristic search were obtained automatically by applying Neighbor-Join and BioNJ algorithms to a matrix of pairwise distances estimated using the Maximum Composite Likelihood (MCL) approach, and then selecting the topology with superior log likelihood value. A discrete Gamma distribution was used to model evolutionary rate differences among sites (5 categories (+G, parameter = 0.5353)). The tree is drawn to scale, with branch lengths measured in the number of substitutions per site. The analysis involved 8 nucleotide sequences. All positions containing gaps and missing data were eliminated. There were a total of 241 positions in the final dataset. Evolutionary analyses were conducted in MEGA7[2].

**Table S1.18: Estimates of Evolutionary Divergence between Sequences**
The number of base differences per sequence from between sequences are shown. The analysis involved 8 nucleotide sequences. Codon positions included were 1st+2nd+3rd+Noncoding. All positions containing gaps and missing data were eliminated. There were a total of 241 positions in the final dataset. Evolutionary analyses were conducted in MEGA7.

| **MAD-APMV6-Pol-241nt** | | | | | | | |
|---|---|---|---|---|---|---|---|
| AB759118-APMV6-JP | 9 | | | | | | |
| GQ406232-APMV6-IT | 9 | 0 | | | | | |
| KP762799-APMV6-KZ | 64 | 63 | 63 | | | | |
| AY029299-APMV6-TW | 66 | 66 | 66 | 15 | | | |
| KT962980-APMV6-RU | 61 | 61 | 61 | 12 | 12 | | |
| JN571486-APMV6-BE | 64 | 64 | 64 | 17 | 4 | 14 | |
| KF267717-APMV6-CN | 65 | 64 | 64 | 7 | 15 | 9 | 17 |

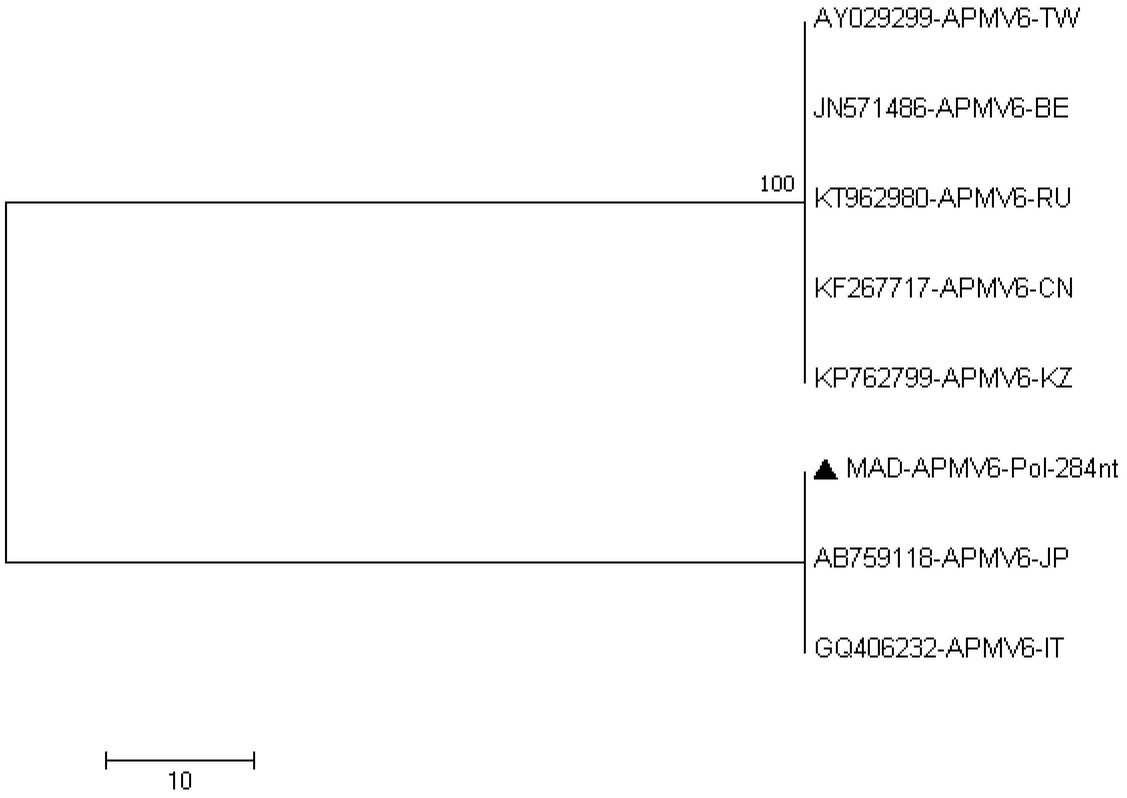


**Figure S1.19: Molecular Phylogenetic analysis by Maximum Likelihood method of APMV6 partial Pol gene**
The evolutionary history was inferred by using the Maximum Likelihood method based on the Kimura 2-parameter model[1]. The tree with the highest log likelihood (-816.02) is shown. The percentage of trees in which the associated taxa clustered together is shown next to the branches. Initial tree(s) for the heuristic search were obtained automatically by applying Neighbor-Join and BioNJ algorithms to a matrix of pairwise distances estimated using the Maximum Composite Likelihood (MCL) approach, and then selecting the topology with superior log likelihood value. A discrete Gamma distribution was used to model evolutionary rate differences among sites (5 categories (+G, parameter = 0.5244)). The tree is drawn to scale, with branch lengths measured in the number of substitutions per site. The analysis involved 8 nucleotide sequences. All positions containing gaps and missing data were eliminated. There were a total of 284 positions in the final dataset. Evolutionary analyses were conducted in MEGA7[2].

**Table S1.19: Estimates of Evolutionary Divergence between Sequences**
The number of base differences per sequence from between sequences are shown. The analysis involved 8 nucleotide sequences. Codon positions included were 1st+2nd+3rd+Noncoding. All positions containing gaps and missing data were eliminated. There were a total of 284 positions in the final dataset. Evolutionary analyses were conducted in MEGA7.

| **MAD-APMV6-Pol-284nt** | | | | | | | |
|---|---|---|---|---|---|---|---|
| AB759118-APMV6-JP | 3 | | | | | | |
| GQ406232-APMV6-IT | 4 | 1 | | | | | |
| KP762799-APMV6-KZ | 77 | 75 | 75 | | | | |
| AY029299-APMV6-TW | 81 | 79 | 79 | 6 | | | |
| KT962980-APMV6-RU | 80 | 78 | 78 | 5 | 5 | | |
| JN571486-APMV6-BE | 78 | 78 | 78 | 7 | 5 | 6 | |
| KF267717-APMV6-CN | 79 | 77 | 77 | 2 | 6 | 5 | 7 |

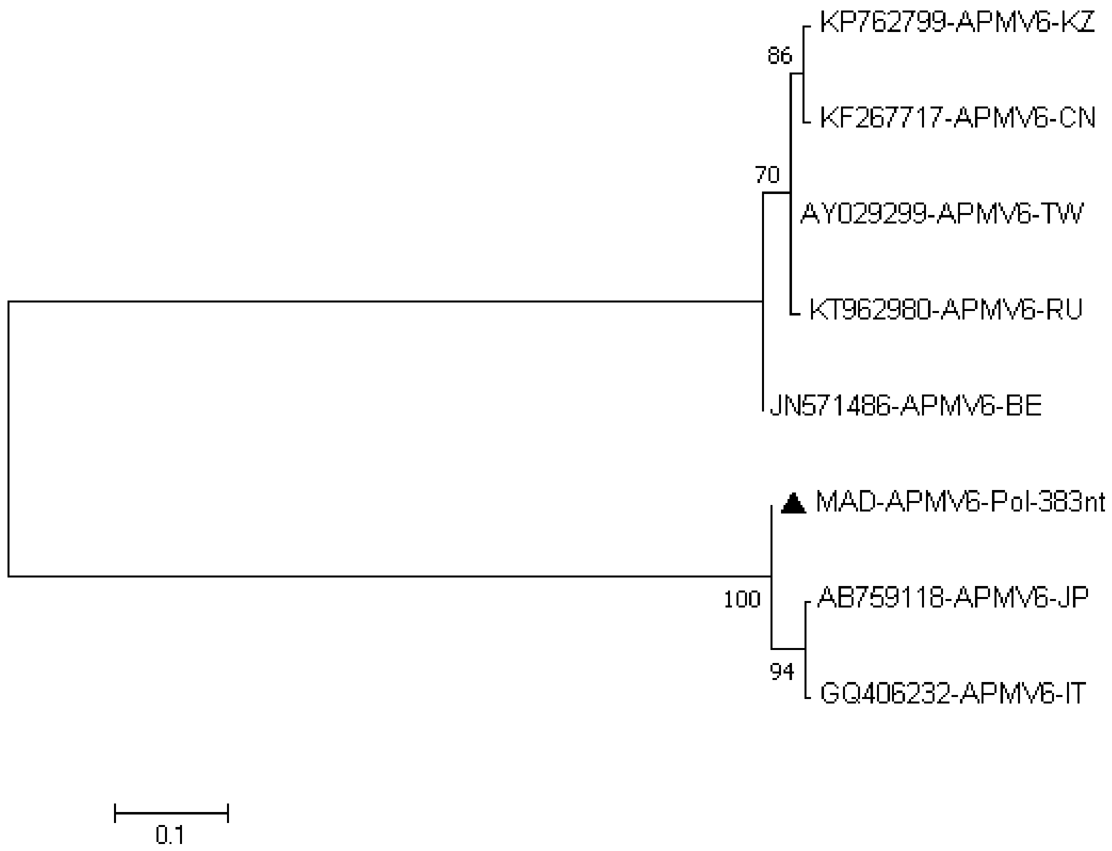


**Figure S1.20: Molecular Phylogenetic analysis by Maximum Likelihood method of APMV6 partial Pol gene**
The evolutionary history was inferred by using the Maximum Likelihood method based on the Tamura 3-parameter model[4]. The tree with the highest log likelihood (-1044.49) is shown. The percentage of trees in which the associated taxa clustered together is shown next to the branches. Initial tree(s) for the heuristic search were obtained automatically by applying Neighbor-Join and BioNJ algorithms to a matrix of pairwise distances estimated using the Maximum Composite Likelihood (MCL) approach, and then selecting the topology with superior log likelihood value. The rate variation model allowed for some sites to be evolutionarily invariable ([+I], 36.68% sites). The tree is drawn to scale, with branch lengths measured in the number of substitutions per site. The analysis involved 8 nucleotide sequences. All positions containing gaps and missing data were eliminated. There were a total of 383 positions in the final dataset. Evolutionary analyses were conducted in MEGA7[2].

**Table S1.20: Estimates of Evolutionary Divergence between Sequences**
The number of base differences per sequence from between sequences are shown. The analysis involved 8 nucleotide sequences. Codon positions included were 1st+2nd+3rd+Noncoding. All positions containing gaps and missing data were eliminated. There were a total of 383 positions in the final dataset. Evolutionary analyses were conducted in MEGA7.

| **MAD-APMV6-Pol-383nt** | | | | | | | |
|---|---|---|---|---|---|---|---|
| AB759118-APMV6-JP | 12 | | | | | | |
| GQ406232-APMV6-IT | 12 | 2 | | | | | |
| KP762799-APMV6-KZ | 90 | 88 | 88 | | | | |
| AY029299-APMV6-TW | 89 | 87 | 87 | 6 | | | |
| KT962980-APMV6-RU | 90 | 88 | 88 | 9 | 3 | | |
| JN571486-APMV6-BE | 91 | 89 | 89 | 13 | 9 | 12 | |
| KF267717-APMV6-CN | 91 | 89 | 89 | 4 | 6 | 9 | 15 |

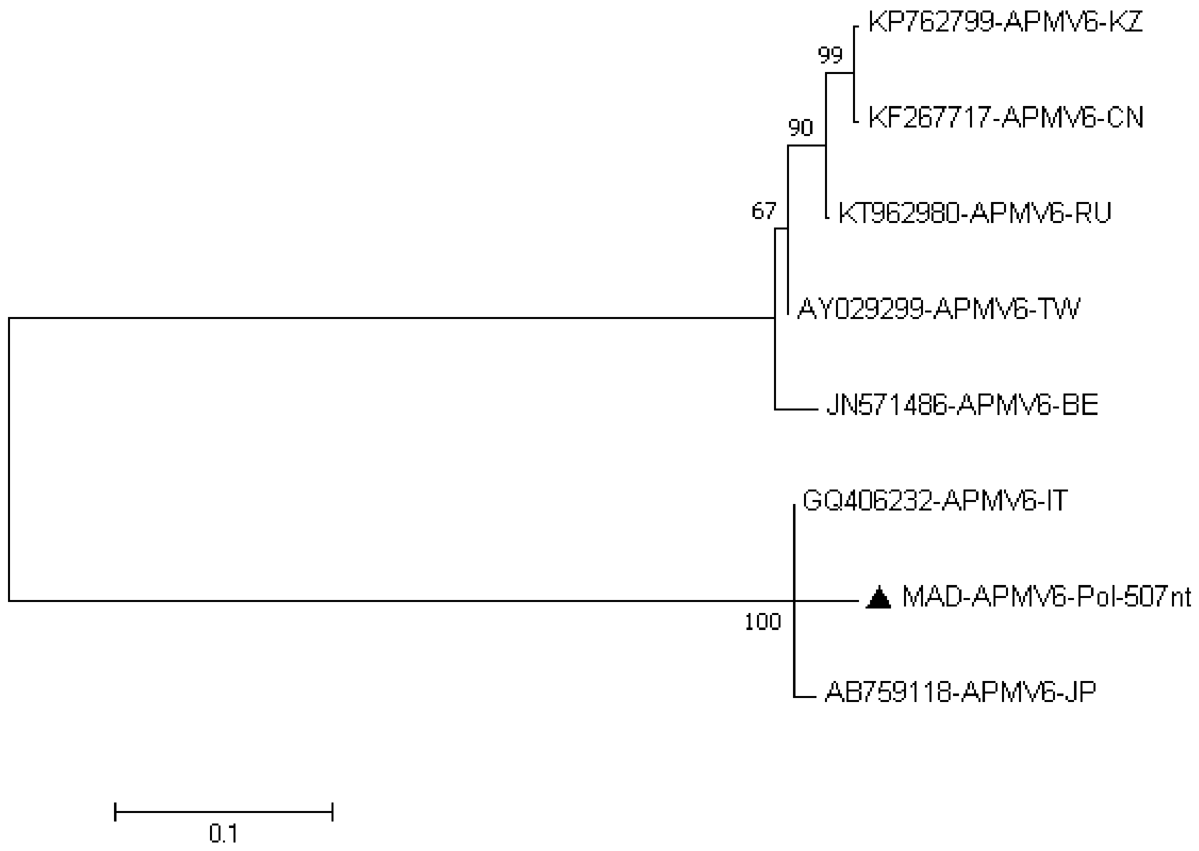


**Figure S1.21: Molecular Phylogenetic analysis by Maximum Likelihood method of APMV6 partial Pol gene**
The evolutionary history was inferred by using the Maximum Likelihood method based on the Kimura 2-parameter model[1]. The tree with the highest log likelihood (-1352.37) is shown. The percentage of trees in which the associated taxa clustered together is shown next to the branches. Initial tree(s) for the heuristic search were obtained automatically by applying Neighbor-Join and BioNJ algorithms to a matrix of pairwise distances estimated using the Maximum Composite Likelihood (MCL) approach, and then selecting the topology with superior log likelihood value. A discrete Gamma distribution was used to model evolutionary rate differences among sites (5 categories (+G, parameter = 0.4413)). The tree is drawn to scale, with branch lengths measured in the number of substitutions per site. The analysis involved 8 nucleotide sequences. All positions containing gaps and missing data were eliminated. There were a total of 507 positions in the final dataset. Evolutionary analyses were conducted in MEGA7[2].

**Table S1.21: Estimates of Evolutionary Divergence between Sequences**
The number of base differences per sequence from between sequences are shown. The analysis involved 8 nucleotide sequences. Codon positions included were 1st+2nd+3rd+Noncoding. All positions containing gaps and missing data were eliminated. There were a total of 507 positions in the final dataset. Evolutionary analyses were conducted in MEGA7.

| | | | | | | | |
|---|---|---|---|---|---|---|---|
| **MAD-APMV6-Pol-507nt** | | | | | | | |
| AB759118-APMV6-JP | 19 | | | | | | |
| GQ406232-APMV6-IT | 14 | 5 | | | | | |
| KP762799-APMV6-KZ | 127 | 119 | 119 | | | | |
| AY029299-APMV6-TW | 124 | 116 | 116 | 15 | | | |
| KT962980-APMV6-RU | 124 | 116 | 116 | 8 | 9 | | |
| JN571486-APMV6-BE | 122 | 116 | 116 | 25 | 12 | 21 | |
| KF267717-APMV6-CN | 128 | 120 | 120 | 2 | 15 | 8 | 25 |

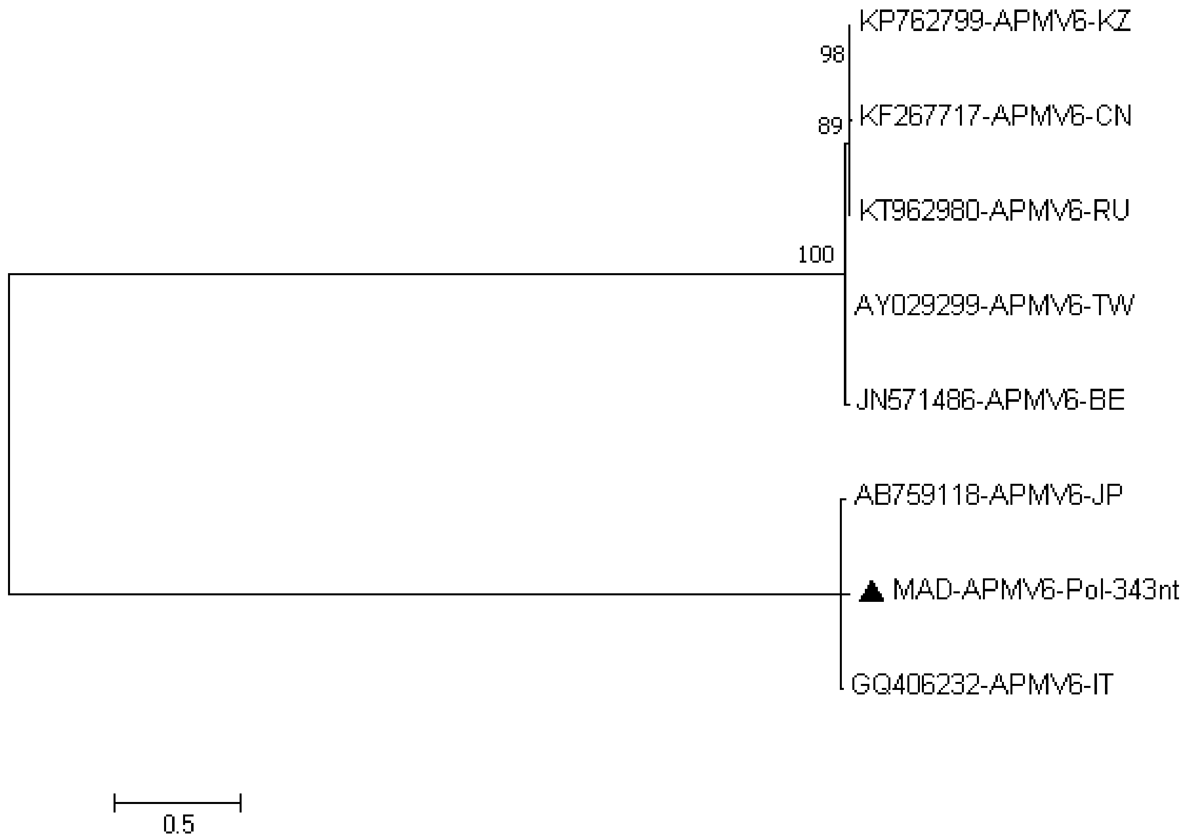


**Figure S1.22: Molecular Phylogenetic analysis by Maximum Likelihood method of APMV6 partial Pol gene**
The evolutionary history was inferred by using the Maximum Likelihood method based on the Kimura 2-parameter model[1]. The tree with the highest log likelihood (-947.92) is shown. The percentage of trees in which the associated taxa clustered together is shown next to the branches. Initial tree(s) for the heuristic search were obtained automatically by applying Neighbor-Join and BioNJ algorithms to a matrix of pairwise distances estimated using the Maximum Composite Likelihood (MCL) approach, and then selecting the topology with superior log likelihood value. A discrete Gamma distribution was used to model evolutionary rate differences among sites (5 categories (+G, parameter = 0.5111)). The tree is drawn to scale, with branch lengths measured in the number of substitutions per site. The analysis involved 8 nucleotide sequences. All positions containing gaps and missing data were eliminated. There were a total of 343 positions in the final dataset. Evolutionary analyses were conducted in MEGA7[2].

**Table S1.22: Estimates of Evolutionary Divergence between Sequences**
The number of base differences per sequence from between sequences are shown. The analysis involved 8 nucleotide sequences. Codon positions included were 1st+2nd+3rd+Noncoding. All positions containing gaps and missing data were eliminated. There were a total of 343 positions in the final dataset. Evolutionary analyses were conducted in MEGA7.

| **MAD-APMV6-Pol-343nt** | | | | | | | |
|---|---|---|---|---|---|---|---|
| AB759118-APMV6-JP | 14 | | | | | | |
| GQ406232-APMV6-IT | 11 | 5 | | | | | |
| KP762799-APMV6-KZ | 94 | 94 | 93 | | | | |
| AY029299-APMV6-TW | 90 | 90 | 89 | 6 | | | |
| KT962980-APMV6-RU | 92 | 92 | 91 | 4 | 6 | | |
| JN571486-APMV6-BE | 91 | 91 | 90 | 10 | 4 | 10 | |
| KF267717-APMV6-CN | 95 | 95 | 94 | 1 | 7 | 5 | 11 |

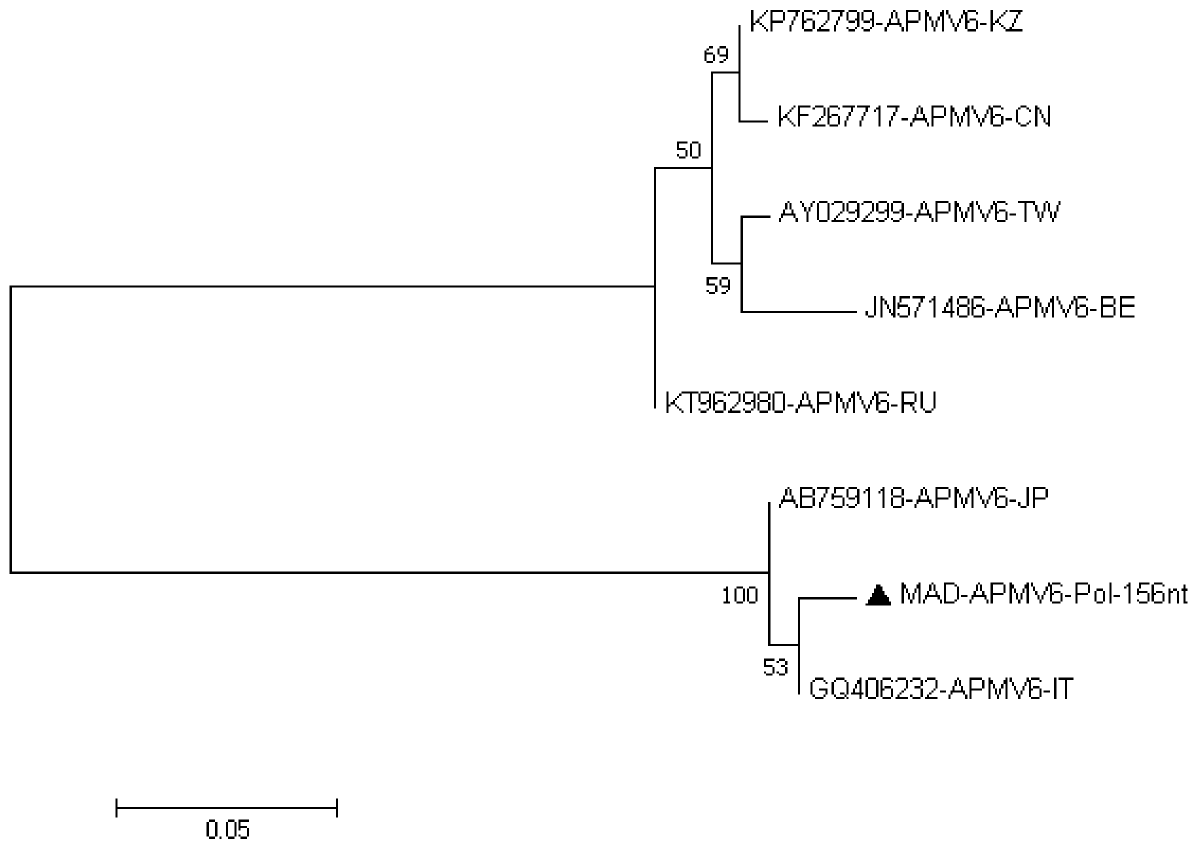


**Figure S1.23: Molecular Phylogenetic analysis by Maximum Likelihood method of APMV6 partial Pol gene**
The evolutionary history was inferred by using the Maximum Likelihood method based on the Tamura 3-parameter model[4]. The tree with the highest log likelihood (-402.62) is shown. The percentage of trees in which the associated taxa clustered together is shown next to the branches. Initial tree(s) for the heuristic search were obtained automatically by applying Neighbor-Join and BioNJ algorithms to a matrix of pairwise distances estimated using the Maximum Composite Likelihood (MCL) approach, and then selecting the topology with superior log likelihood value. The tree is drawn to scale, with branch lengths measured in the number of substitutions per site. The analysis involved 8 nucleotide sequences. All positions containing gaps and missing data were eliminated. There were a total of 156 positions in the final dataset. Evolutionary analyses were conducted in MEGA7[2].

**Table S1.23: Estimates of Evolutionary Divergence between Sequences**
The number of base differences per sequence from between sequences are shown. The analysis involved 8 nucleotide sequences. Codon positions included were 1st+2nd+3rd+Noncoding. All positions containing gaps and missing data were eliminated. There were a total of 156 positions in the final dataset. Evolutionary analyses were conducted in MEGA7.

| **MAD-APMV6-Pol-156nt** | | | | | | | |
|---|---|---|---|---|---|---|---|
| AB759118-APMV6-JP | 3 | | | | | | |
| GQ406232-APMV6-IT | 2 | 1 | | | | | |
| KP762799-APMV6-KZ | 41 | 40 | 41 | | | | |
| AY029299-APMV6-TW | 40 | 39 | 40 | 3 | | | |
| KT962980-APMV6-RU | 39 | 38 | 39 | 3 | 4 | | |
| JN571486-APMV6-BE | 44 | 43 | 44 | 6 | 5 | 7 | |
| KF267717-APMV6-CN | 40 | 39 | 40 | 1 | 4 | 4 | 7 |

## 2. Gammacoronavirus from MAD faecal sample

| Figure and Table | Long Name (Format: Sample-virus-protein-length-quality-coverage-year) | Short name (Format: Sample-virus-protein-length-year) | Coverage | No. of nucleotides | Mapping quality threshold | NCBI accession number |
|---|---|---|---|---|---|---|
| S2.1 | MAD-Gammacoronavirus-NSP12-1071nt-Q32-C-4-65-2016 | MAD-GCoV-NSP12-1071nt | 4-65 | 1071 | 32 | MG991677 |
| S2.2 | MAD-Gammacoronavirus-Spike-protein-524nt-Q32-C-3-19-2016 | MAD-GCoV-SP-524nt | 3-19 | 524 | 32 | MG991678 |
| S2.3 | MAD-Gammacoronavirus-Spike-protein-469nt-Q32-C-3-19-2016 | MAD-GCoV-SP-469nt | 3-13 | 469 | 32 | MG991679 |
| NCBI sequences taken for phylogenetic analysis | | | | | | |
| Long Name | | Short Name (Format: NCBI accession number-virus-country/state-year) | Country of collection | Collection date | | |
| KM454473-Duck-coronavirus-isolate-DK/GD/27/2014-complete-genome | | KM454473-DCoV-CN | China | 2014 | | |
| LN610099-Guinea-fowl-coronavirus-GfCoV/FR/2011-complete-genome | | LN610099-GfCoV-FR | France | - | | |
| KR822424-European-turkey-coronavirus-080385d-complete-genome | | KR822424-TCoV-FR | France | 2008 | | |
| EU022526-Turkey-coronavirus-isolate-TCoV-ATCC-complete-genome | | EU022526-TCoV-US | United States of America | - | | |
| KT254279-Pigeon-dominant-Coronavirus-isolate-PdCoV/PG/Guangdong/1418/2014-NSP12-(1ab)-gene-partial-cds | | KT254279-PdCoV-CN | China | 2014 | | |
| FN430415-Infectious-bronchitis-virus-NGA/A116E7/2006-complete-genome | | FN430415-IBV-NG | Nigeria | 2006 | | |
| KF652223-Turkey-coronavirus-isolate-NC/20/09-spike- | | KF652223-TCoV(P)-US | United States of America | 2009 | | |

| | | | |
|---|---|---|---|
| glycoprotein-(S)-gene-complete-cds | | | |

The table points towards the molecular phylogenetic analysis by Maximum Likelihood method of GCoV. The first half of the table provides details to identify the sample from which the virus was isolated, protein that encodes the consensus sequences that are being analysed, the length of the consensus sequences thus providing the depth of the gene being analysed, minimum mapping quality threshold used in IGV for the generation of the consensus sequences, the coverage of the consensus sequences from IGV, the year of collection of the sample and the NCBI accession number assigned to the particular consensus sequence. Corresponding short names have been used in the phylogenetic trees which contain the sample, virus, protein and the number of nucleotides. The second half of the table provides the details of the sequences that were used for the molecular phylogenetic analysis. They were found to be the most closely related sequences to the consensus sequences generated using BLASTN. Corresponding short names have been used in the phylogenetic trees which contain the NCBI accession number, virus and the country of collection. The collection date is given as retrieved from the corresponding NCBI nucleotide reference dataset which together with the country of collection can provide an insight into the possible evolution of the virus through the years.

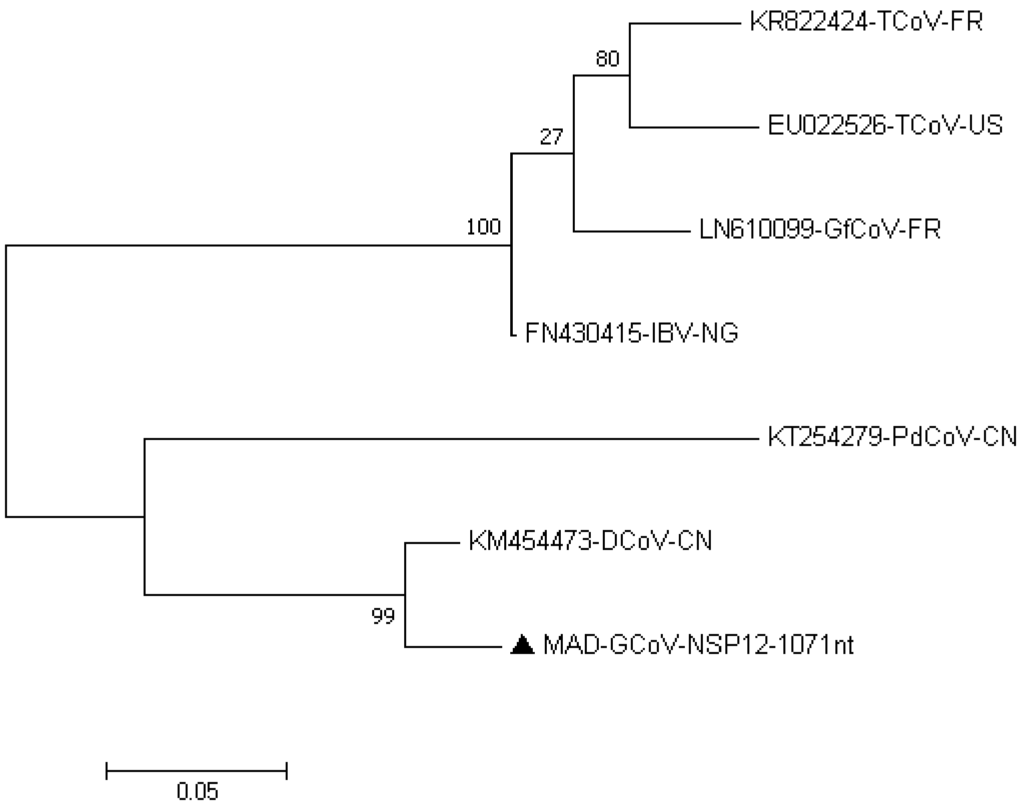


**Figure S2.1. Molecular Phylogenetic analysis by Maximum Likelihood method of GCoV partial OLPP gene**
The evolutionary history was inferred by using the Maximum Likelihood method based on the Tamura 3-parameter model[4]. The tree with the highest log likelihood (-3293.18) is shown. The percentage of trees in which the associated taxa clustered together is shown next to the branches. Initial tree(s) for the heuristic search were obtained automatically by applying Neighbor-Join and BioNJ algorithms to a matrix of pairwise distances estimated using the Maximum Composite Likelihood (MCL) approach, and then selecting the topology with superior log likelihood value. A discrete Gamma distribution was used to model evolutionary rate differences among sites (5 categories (+G, parameter = 0.2729)). The tree is drawn to scale, with branch lengths measured in the number of substitutions per site. The analysis involved 7 nucleotide sequences. Codon positions included were 1st+2nd+3rd+Noncoding. All positions containing gaps and missing data were eliminated. There were a total of 1071 positions in the final dataset. Evolutionary analyses were conducted in MEGA7[2].

**Table S2.1. Estimates of Evolutionary Divergence between Sequences**
The number of base differences per sequence from between sequences are shown. The analysis involved 7 nucleotide sequences. Codon positions included were 1st+2nd+3rd+Noncoding. All positions containing gaps and missing data were eliminated. There were a total of 1071 positions in the final dataset. Evolutionary analyses were conducted in MEGA7.

| | | | | | | |
|---|---|---|---|---|---|---|
| KM454473-DCoV-CN | | | | | | |
| LN610099-GfCoV-FR | 163 | | | | | |
| KR822424-TCoV-FR | 177 | 67 | | | | |
| EU022526-TCoV-US | 178 | 72 | 59 | | | |
| KT254279-PdCoV-CN | 152 | 186 | 170 | 187 | | |
| FN430415-IBV-NG | 158 | 47 | 58 | 62 | 181 | |
| **MAD-GCoV-NSP12-1071nt** | 41 | 167 | 185 | 186 | 154 | 166 |

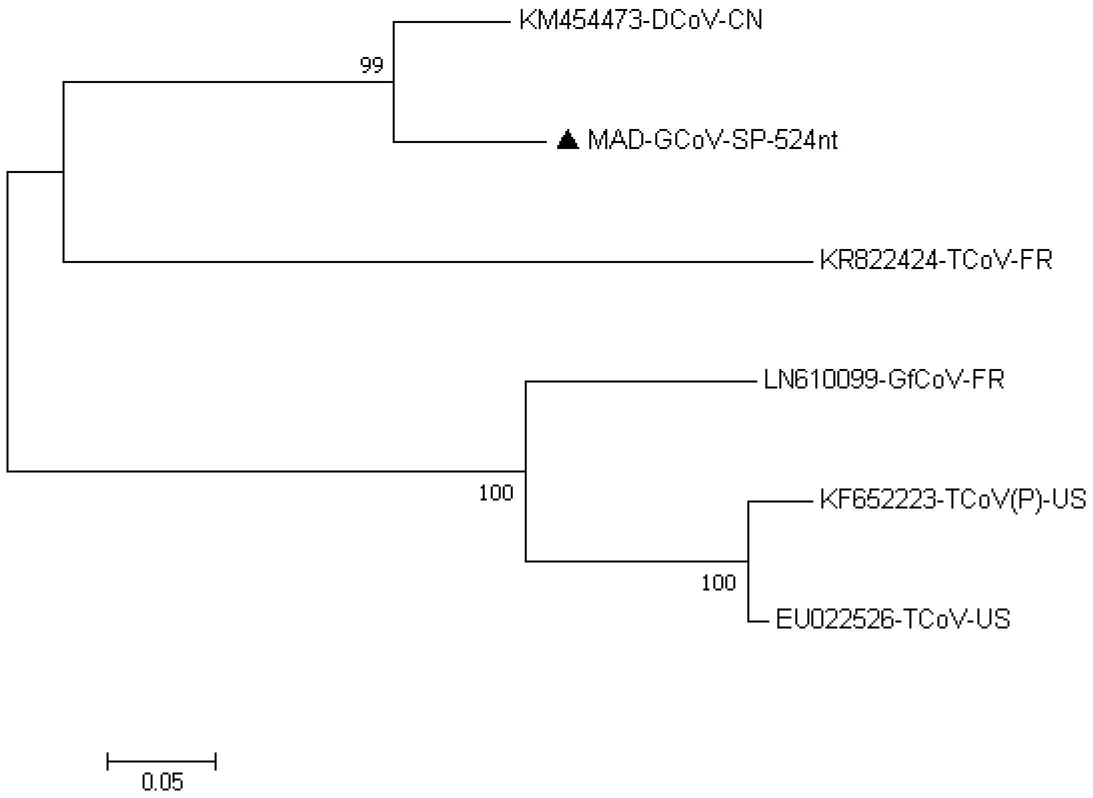


**Figure S2.2: Molecular Phylogenetic analysis by Maximum Likelihood method of GCoV partial SP gene**
The evolutionary history was inferred by using the Maximum Likelihood method based on the Hasegawa-Kishino-Yano model[5]. The tree with the highest log likelihood (-2146.14) is shown. The percentage of trees in which the associated taxa clustered together is shown next to the branches. Initial tree(s) for the heuristic search were obtained automatically by applying Neighbor-Join and BioNJ algorithms to a matrix of pairwise distances estimated using the Maximum Composite Likelihood (MCL) approach, and then selecting the topology with superior log likelihood value. A discrete Gamma distribution was used to model evolutionary rate differences among sites (5 categories (+G, parameter = 0.6568)). The tree is drawn to scale, with branch lengths measured in the number of substitutions per site. The analysis involved 6 nucleotide sequences. Codon positions included were 1st+2nd+3rd+Noncoding. All positions containing gaps and missing data were eliminated. There were a total of 524 positions in the final dataset. Evolutionary analyses were conducted in MEGA7[2].

**Table S2.2: Estimates of Evolutionary Divergence between Sequences**
The number of base differences per sequence from between sequences are shown. The analysis involved 6 nucleotide sequences. Codon positions included were 1st+2nd+3rd+Noncoding. All positions containing gaps and missing data were eliminated. There were a total of 524 positions in the final dataset. Evolutionary analyses were conducted in MEGA7.

| | | | | | |
|---|---|---|---|---|---|
| KM454473-DCoV-CN | | | | | |
| LN610099-GfCoV-FR | 143 | | | | |
| KR822424-TCoV-FR | 149 | 172 | | | |
| KF652223-TCoV(P)-US | 160 | 88 | 167 | | |
| EU022526-TCoV-US | 153 | 83 | 160 | 19 | |
| **MAD-GCoV-SP-524nt** | 54 | 143 | 151 | 159 | 156 |

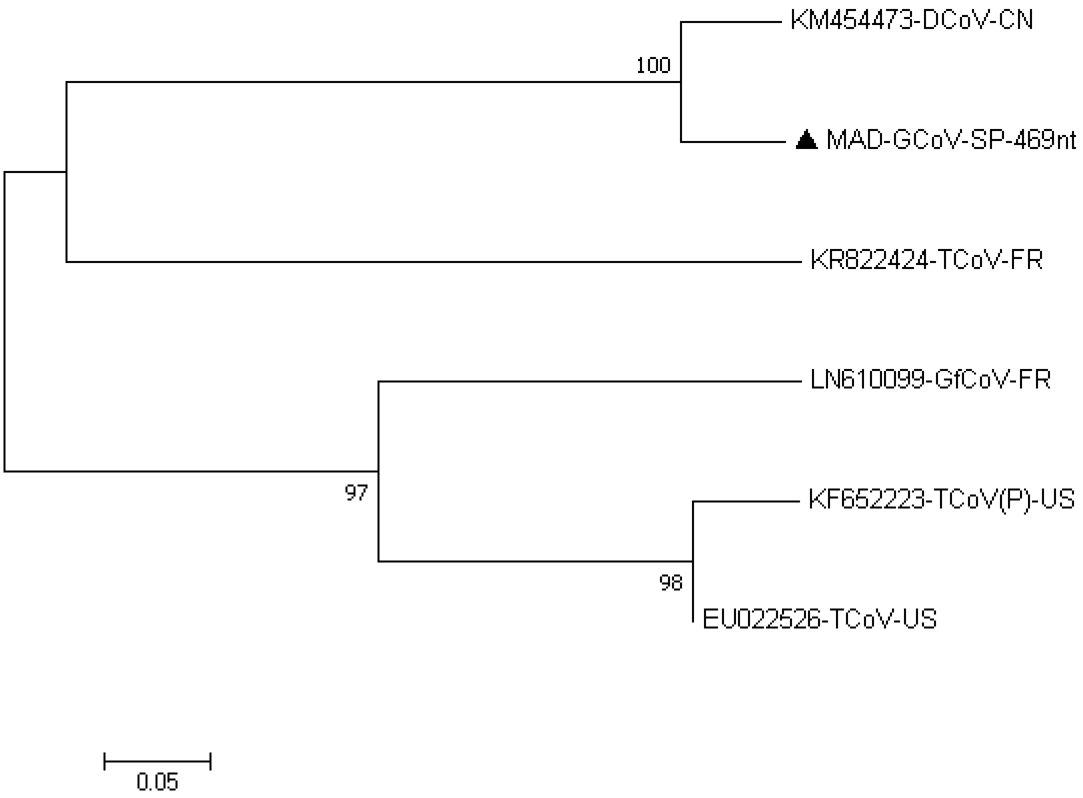


**Figure S2.3: Molecular Phylogenetic analysis by Maximum Likelihood method of GCoV partial SP gene**
The evolutionary history was inferred by using the Maximum Likelihood method based on the Tamura 3-parameter model[4]. The tree with the highest log likelihood (-1893.27) is shown. The percentage of trees in which the associated taxa clustered together is shown next to the branches. Initial tree(s) for the heuristic search were obtained automatically by applying Neighbor-Join and BioNJ algorithms to a matrix of pairwise distances estimated using the Maximum Composite Likelihood (MCL) approach, and then selecting the topology with superior log likelihood value. A discrete Gamma distribution was used to model evolutionary rate differences among sites (5 categories (+G, parameter = 0.5370)). The tree is drawn to scale, with branch lengths measured in the number of substitutions per site. The analysis involved 6 nucleotide sequences. Codon positions included were 1st+2nd+3rd+Noncoding. All positions containing gaps and missing data were eliminated. There were a total of 469 positions in the final dataset. Evolutionary analyses were conducted in MEGA7[2].

**Table S2.3: Estimates of Evolutionary Divergence between Sequences**
The number of base differences per sequence from between sequences are shown. Standard error estimate(s) are shown above the diagonal. The analysis involved 6 nucleotide sequences. Codon positions included were 1st+2nd+3rd+Noncoding. All positions containing gaps and missing data were eliminated. There were a total of 469 positions in the final dataset. Evolutionary analyses were conducted in MEGA7.

| | | | | | |
|---|---|---|---|---|---|
| KM454473-DCoV-CN | | | | | |
| LN610099-GfCoV-FR | 126 | | | | |
| KR822424-TCoV-FR | 127 | 129 | | | |
| KF652223-TCoV(P)-US | 132 | 101 | 126 | | |
| EU022526-TCoV-US | 127 | 93 | 125 | 21 | |
| **MAD-GCoV-SP-469nt** | 38 | 130 | 131 | 133 | 126 |

## 3. Deltacoronavirus from MAD faecal sample

| Figure and Table | Long Name (Format: Sample-virus-protein-length-quality-coverage-year) | Short name (Format: Sample-virus-protein-length-year) | Coverage | No. of nucleotides | Mapping quality threshold | NCBI accession number |
|---|---|---|---|---|---|---|
| S3.1 | MAD-Deltacoronavirus-spike-glycoprotein-201nt-Q20-C-3-8-2016 | MAD-DCoV-SP-201nt | 3-8 | 201 | 20 | Part of MH013337 |
| S3.2 | MAD-Deltacoronavirus-spike-glycoprotein-201nt-Q20-C-3-8-2016 | MAD-DCoV-SP-aa-201nt | 3-8 | 201 | 20 | Part of MH013337 |
| S3.3 | MAD-Deltacoronavirus-replicase-polyprotein-205nt-Q20-C-3-30-2016 | MAD-DCoV-RPP-205nt | 3-30 | 205 | 20 | Part of MH013331 |
| S3.4 | MAD-Deltacoronavirus-replicase-polyprotein-117nt-Q20-C-4-21-2016 | MAD-DCoV-RPP-117nt | 4-21 | 117 | 20 | Part of MH013335 |
| S3.5 | MAD-Deltacoronavirus-spike-glycoprotein-1131nt-Q20-C-290-1700-2016 | MAD-DCoV-SP-1131nt | 290-1700 | 1131 | 20 | Part of MH013337 |
| S3.6 | MAD-Deltacoronavirus-spike-glycoprotein-1131nt-Q20-C-290-1700-2016 | MAD-DCoV-SP-aa-1131nt | 290-1700 | 1131 | 20 | Part of MH013337 |
| S3.7 | MAD-Deltacoronavirus-replicase-polyprotein-2121nt-Q20-C-39-1266-2016 | MAD-DCoV-RPP-2121nt | 39-1266 | 2121 | 20 | Part of MH013332 |
| S3.8 | MAD-Deltacoronavirus-replicase-polyprotein-2121nt-Q20-C-39-1266-2016 | MAD-DCoV-RPP-aa-2121nt | 39-1266 | 2121 | 20 | Part of MH013332 |
| S3.9 | MAD-Deltacoronavirus-envelope-protein-261nt-Q20-C-36-6274-2016 | MAD-DCoV-EP-261nt | 36-6274 | 261 | 20 | MH013336 |

| | | | | | | |
|---|---|---|---|---|---|---|
| S3.10 | MAD-Deltacoronavirus-envelope-protein-261nt-Q20-C-36-6274-2016 | MAD-DCoV-EP-aa-261nt | 36-6274 | 261 | 20 | MH013336 |
| S3.11 | MAD-Deltacoronavirus-membrane-protein-654nt-Q20-C-36-6274-2016 | MAD-DCoV-MP-654nt | 36-6274 | 654 | 20 | MH013338 |
| S3.12 | MAD-Deltacoronavirus-membrane-protein-654nt-Q20-C-36-6274-2016 | MAD-DCoV-MP-aa-654nt | 36-6274 | 654 | 20 | MH013338 |
| S3.13 | MAD-Deltacoronavirus-orf1bstart-1095nt-Q20-C-5-336-2016 | MAD-DCoV-RPP-1095nt | 5-336 | 1095 | 20 | MH013333 |
| S3.14 | MAD-Deltacoronavirus-orf1bstart-1095nt-Q20-C-5-336-2016 | MAD-DCoV-RPP-aa-1095nt | 5-336 | 1095 | 20 | MH013333 |
| S3.15 | MAD-Deltacoronavirus-NS6-protein-276nt-Q20-C-36-6274-2016 | MAD-DCoV-NS6-276nt | 36-6274 | 276 | 20 | MH013339 |
| S3.16 | MAD-Deltacoronavirus-NS6-protein-276nt-Q20-C-36-6274-2016 | MAD-DCoV-NS6-aa-276nt | 36-6274 | 276 | 20 | MH013339 |
| S3.17 | MAD-Deltacoronavirus-Orf1bNSP11-13-2619nt-Q20-C-7-1923-2016 | MAD-DCoV-RPP-2619nt | 7-1923 | 2619 | 20 | MH013334 |
| S3.18 | MAD-Deltacoronavirus-Orf1bNSP11-13-2619nt-Q20-C-7-1923-2016 | MAD-DCoV-RPP-aa-2619nt | 7-1923 | 2619 | 20 | MH013334 |
| S3.19 | MAD-Deltacoronavirus-Orf1bNSP11-1914nt-Q20-C-14-290-2016 | MAD-DCoV-RPP-1914nt | 14-290 | 1914 | 20 | MH013335 |
| S3.20 | MAD-Deltacoronavirus- | MAD-DCoV-RPP-aa-1914nt | 14-290 | 1914 | 20 | MH013335 |

| | | | | | | |
|---|---|---|---|---|---|---|
| | Orf1bNSP11-1914nt-Q20-C-14-290-2016 | | | | | |
| S3.21 | MAD-Deltacoronavirus-Orf1a-10650nt-Q20-C-6-2326-2016 | MAD-DCoV-Orf1a-aa-10650nt | 6-2326 | 10650 | 20 | MH013332 |
| S3.22 | MAD-Deltacoronavirus-Orf1b-polymerase-2076nt-Q20-C-5-475-2016 | MAD-DCoV-RPP-aa-2076nt | 5-475 | 2076 | 20 | MH013331 |
| S3.23 | MAD-Deltacoronavirus-spike-glycoprotein-3702nt-Q20-C-36-6274-2016 | MAD-DCoV-SP-aa-3702nt | 36-6274 | 3702 | 20 | MH013337 |
| NCBI sequences taken for phylogenetic analysis | | | | | | |
| Long Name | | Short Name (Format: NCBI accession number-virus-country/state-year) | Country of collection | Collection date | | |
| JQ065049-Common-moorhen coronavirus-HKU21-strain-HKU21-8295-complete-genome | | JQ065049-MCoV-CN | China | 2007 | | |
| JQ065046-Magpie-robin-coronavirus HKU18-strain-HKU18-chu3-complete-genome | | JQ065046-MrCoV-CN | China | 2007 | | |
| FJ376622-Munia-coronavirus-HKU13-3514-complete-genome | | FJ376622-MuCoV-CN | China | 2007 | | |
| FJ376621-Thrush-coronavirus-HKU12-600-complete-genome | | FJ376621-TCoV-CN | China | 2007 | | |
| MF431743-Porcine-deltacoronavirus-strain-SD-complete-genome | | MF431743-PDCoV-CN | China | 2014 | | |
| FJ376620-Bulbul-coronavirus-HKU11-796-complete-genome | | FJ376620-BCoV-CN | China | 2007 | | |
| JQ065047-Night-heron-coronavirus-HKU19-strain-HKU19-6918-complete genome | | JQ065047-NhCoV-CN | China | 2007 | | |
| JQ065048-Wigeon-coronavirus-HKU20-strain-HKU20-9243-complete-genome | | JQ065048-WCoV-CN | China | 2008 | | |

The table points towards the molecular phylogenetic analysis by Maximum Likelihood method of DCoV. The first half of the table provides details to identify the sample from which the virus was isolated, protein that encodes the consensus sequences that are being analysed, the length of the consensus sequences thus providing the depth of the gene being analysed, minimum mapping

quality threshold used in IGV for the generation of the consensus sequences, the coverage of the consensus sequences from IGV, the year of collection of the sample and the NCBI accession number assigned to the particular consensus sequence. Corresponding short names have been used in the phylogenetic trees which contain the sample, virus, protein and the number of nucleotides. In case of the amino acid sequence analysis, the short names contain the “aa” after the protein. The second half of the table provides the details of the sequences that were used for the molecular phylogenetic analysis. They were found to be the most closely related sequences to the consensus sequences generated using BLASTN. Corresponding short names have been used in the phylogenetic trees which contain the NCBI accession number, virus and the country of collection. The collection date is given as retrieved from the corresponding NCBI nucleotide reference dataset which together with the country of collection can provide an insight into the possible evolution of the virus through the years.

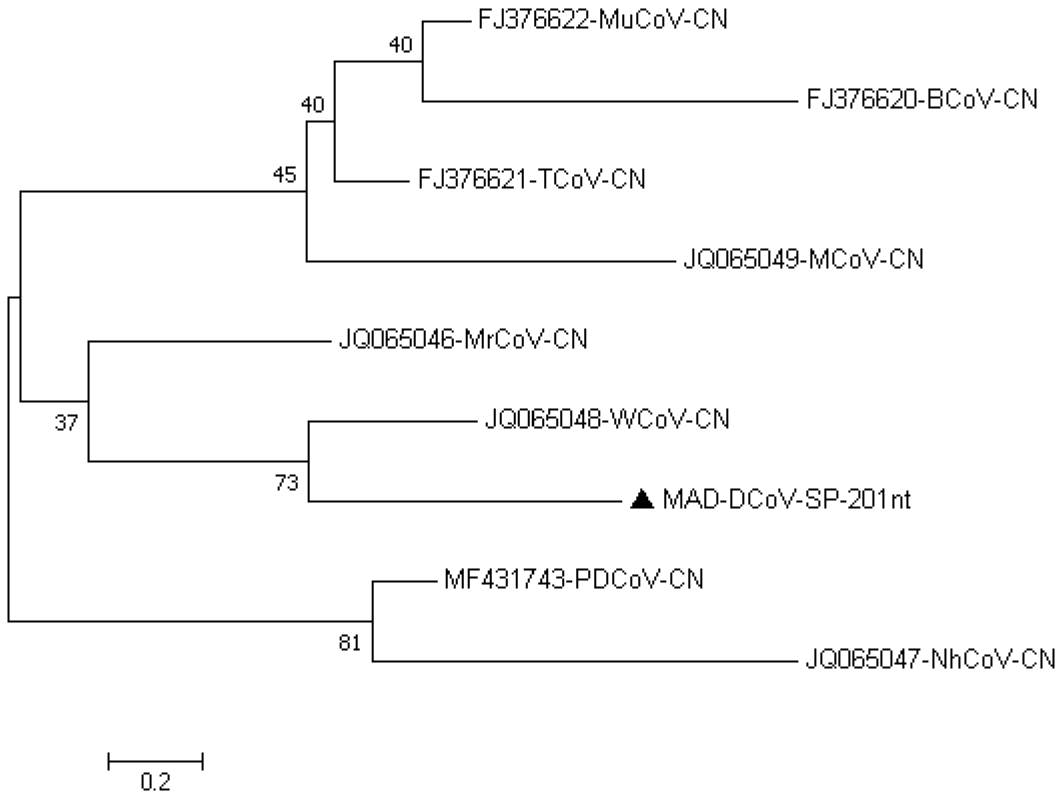


**Figure S3.1: Molecular Phylogenetic analysis by Maximum Likelihood method of DCoV partial SP gene**
The evolutionary history was inferred by using the Maximum Likelihood method based on the Tamura 3-parameter model[4]. The tree with the highest log likelihood (-1292.92) is shown. The percentage of trees in which the associated taxa clustered together is shown next to the branches. Initial tree(s) for the heuristic search were obtained automatically by applying Neighbor-Join and BioNJ algorithms to a matrix of pairwise distances estimated using the Maximum Composite Likelihood (MCL) approach, and then selecting the topology with superior log likelihood value. A discrete Gamma distribution was used to model evolutionary rate differences among sites (5 categories (+G, parameter = 0.6091)). The tree is drawn to scale, with branch lengths measured in the number of substitutions per site. The analysis involved 9 nucleotide sequences. Codon positions included were 1st+2nd+3rd+Noncoding. All positions containing gaps and missing data were eliminated. There were a total of 201 positions in the final dataset. Evolutionary analyses were conducted in MEGA7[2].

**Table S3.1: Estimates of Evolutionary Divergence between Sequences**
The number of base differences per sequence from between sequences are shown. The analysis involved 9 nucleotide sequences. Codon positions included were 1st+2nd+3rd+Noncoding. All positions containing gaps and missing data were eliminated. There were a total of 201 positions in the final dataset. Evolutionary analyses were conducted in MEGA7.

| | | | | | | | | |
|---|---|---|---|---|---|---|---|---|
| JQ065048-WCoV-CN | | | | | | | | |
| JQ065049-MCoV-CN | 49 | | | | | | | |
| JQ065046-MrCoV-CN | 52 | 59 | | | | | | |
| FJ376622-MuCoV-CN | 48 | 48 | 54 | | | | | |
| FJ376621-TCoV-CN | 50 | 41 | 57 | 32 | | | | |
| MF431743-PDCoV-CN | 57 | 58 | 55 | 57 | 56 | | | |
| FJ376620-BCoV-CN | 55 | 53 | 60 | 40 | 45 | 56 | | |
| JQ065047-NhCoV-CN | 63 | 59 | 53 | 54 | 49 | 45 | 56 | |
| **MAD-DCoV-SP-201nt** | 37 | 52 | 53 | 58 | 58 | 59 | 58 | 61 |

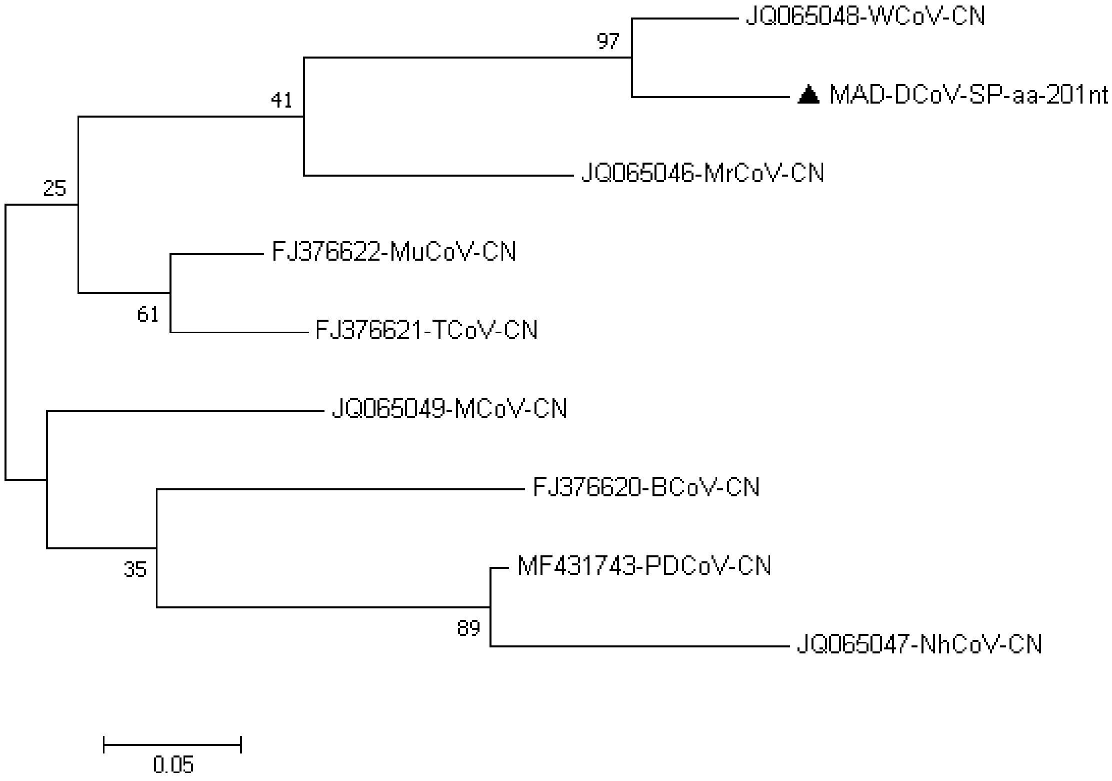


**Figure S3.2. Molecular Phylogenetic analysis by Maximum Likelihood method for amino acid sequences of DCoV partial SP gene**

The evolutionary history was inferred by using the Maximum Likelihood method based on the Le_Gascuel_2008 model[6]. The tree with the highest log likelihood (-501.04) is shown. The percentage of trees in which the associated taxa clustered together is shown next to the branches. Initial tree(s) for the heuristic search were obtained automatically by applying Neighbor-Join and BioNJ algorithms to a matrix of pairwise distances estimated using a JTT model, and then selecting the topology with superior log likelihood value. The rate variation model allowed for some sites to be evolutionarily invariable ([+*I*], 60.17% sites). The tree is drawn to scale, with branch lengths measured in the number of substitutions per site. The analysis involved 9 amino acid sequences. All positions containing gaps and missing data were eliminated. There were a total of 67 positions in the final dataset. Evolutionary analyses were conducted in MEGA7[2].

**Table S3.2. Estimates of Evolutionary Divergence between Sequences**

The number of amino acid differences per sequence from between sequences are shown. The analysis involved 9 amino acid sequences. All positions containing gaps and missing data were eliminated. There were a total of 67 positions in the final dataset. Evolutionary analyses were conducted in MEGA7.

| | | | | | | | | |
|---|---|---|---|---|---|---|---|---|
| JQ065048-WCoV-CN | | | | | | | | |
| JQ065049-MCoV-CN | 11 | | | | | | | |
| JQ065046-MrCoV-CN | 12 | 15 | | | | | | |
| FJ376622-MuCoV-CN | 13 | 11 | 9 | | | | | |
| FJ376621-TCoV-CN | 12 | 12 | 11 | 5 | | | | |
| MF431743-PDCoV-CN | 17 | 11 | 13 | 11 | 12 | | | |
| FJ376620-BCoV-CN | 17 | 14 | 15 | 10 | 13 | 12 | | |
| JQ065047-NhCoV-CN | 17 | 15 | 14 | 13 | 11 | 6 | 15 | |
| **MAD-DCoV-SP-aa-201nt** | 5 | 13 | 12 | 13 | 13 | 16 | 16 | 17 |

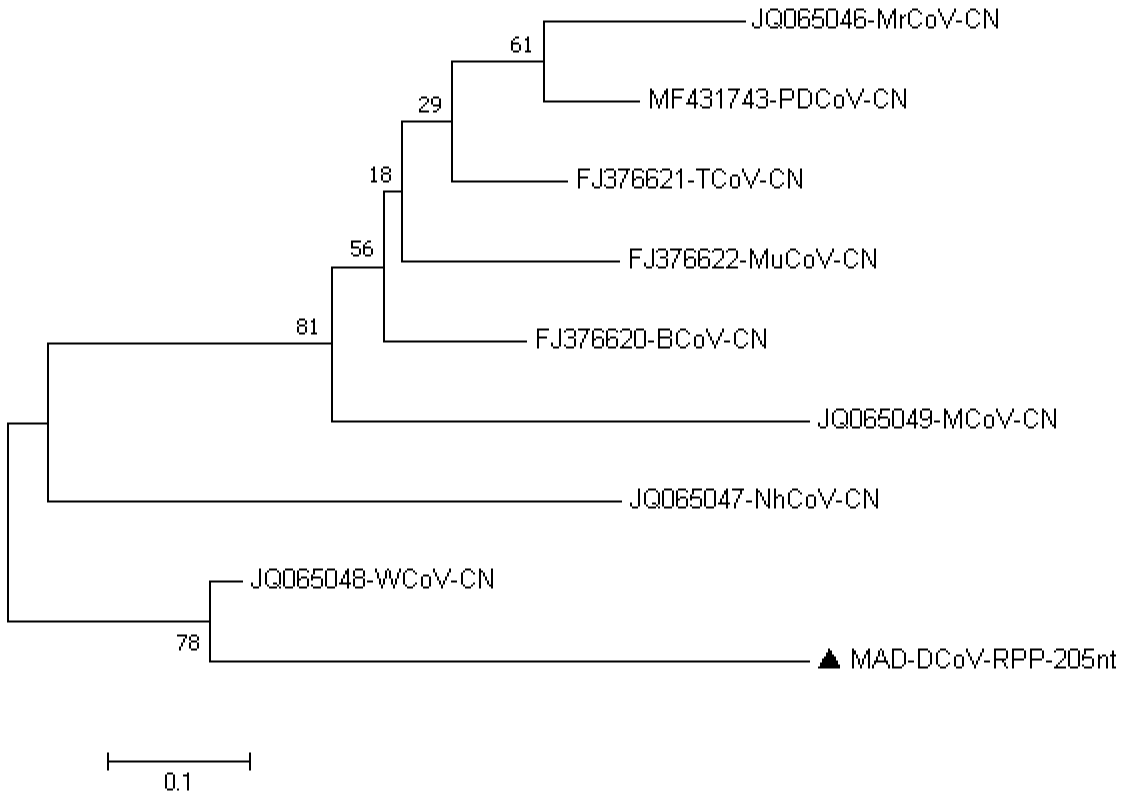


**Figure S3.3: Molecular Phylogenetic analysis by Maximum Likelihood method of DCoV partial RPP gene**
The evolutionary history was inferred by using the Maximum Likelihood method based on the Tamura 3-parameter model[4]. The tree with the highest log likelihood (-1144.72) is shown. The percentage of trees in which the associated taxa clustered together is shown next to the branches. Initial tree(s) for the heuristic search were obtained automatically by applying Neighbor-Join and BioNJ algorithms to a matrix of pairwise distances estimated using the Maximum Composite Likelihood (MCL) approach, and then selecting the topology with superior log likelihood value. A discrete Gamma distribution was used to model evolutionary rate differences among sites (5 categories (+G, parameter = 0.4466)). The tree is drawn to scale, with branch lengths measured in the number of substitutions per site. The analysis involved 9 nucleotide sequences. Codon positions included were 1st+2nd+3rd+Noncoding. All positions containing gaps and missing data were eliminated. There were a total of 205 positions in the final dataset. Evolutionary analyses were conducted in MEGA7[2].

**Table S3.3: Estimates of Evolutionary Divergence between Sequences**
The number of base differences per sequence from between sequences are shown. The analysis involved 9 nucleotide sequences. Codon positions included were 1st+2nd+3rd+Noncoding. All positions containing gaps and missing data were eliminated. There were a total of 205 positions in the final dataset. Evolutionary analyses were conducted in MEGA7.

| | | | | | | | | |
|---|---|---|---|---|---|---|---|---|
| JQ065049-MCoV-CN | | | | | | | | |
| JQ065046-MrCoV-CN | 48 | | | | | | | |
| FJ376622-MuCoV-CN | 43 | 35 | | | | | | |
| FJ376621-TCoV-CN | 37 | 35 | 34 | | | | | |
| MF431743-PDCoV-CN | 47 | 29 | 33 | 29 | | | | |
| FJ376620-BCoV-CN | 43 | 36 | 36 | 28 | 32 | | | |
| JQ065047-NhCoV-CN | 53 | 60 | 55 | 50 | 52 | 48 | | |
| JQ065048-WCoV-CN | 48 | 58 | 46 | 46 | 53 | 43 | 45 | |
| **MAD-DCoV-RPP-205nt** | 46 | 59 | 50 | 51 | 58 | 53 | 52 | 39 |

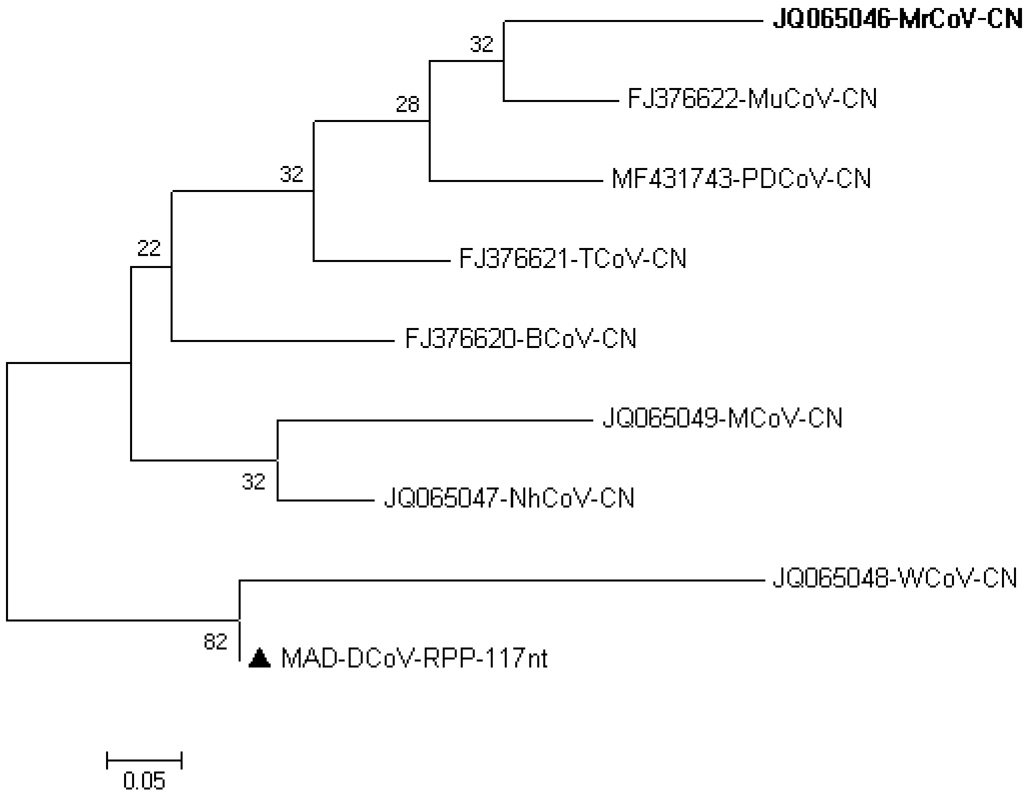


**Figure S3.4: Molecular Phylogenetic analysis by Maximum Likelihood method of DCoV partial RPP gene**
The evolutionary history was inferred by using the Maximum Likelihood method based on the Tamura 3-parameter model[4]. The tree with the highest log likelihood (-653.38) is shown. The percentage of trees in which the associated taxa clustered together is shown next to the branches. Initial tree(s) for the heuristic search were obtained automatically by applying Neighbor-Join and BioNJ algorithms to a matrix of pairwise distances estimated using the Maximum Composite Likelihood (MCL) approach, and then selecting the topology with superior log likelihood value. A discrete Gamma distribution was used to model evolutionary rate differences among sites (5 categories (+G, parameter = 0.4247)). The tree is drawn to scale, with branch lengths measured in the number of substitutions per site. The analysis involved 9 nucleotide sequences. Codon positions included were 1st+2nd+3rd+Noncoding. All positions containing gaps and missing data were eliminated. There were a total of 117 positions in the final dataset. Evolutionary analyses were conducted in MEGA7[2].

**Table S3.4: Estimates of Evolutionary Divergence between Sequences**
The number of base differences per sequence from between sequences are shown. The analysis involved 9 nucleotide sequences. Codon positions included were 1st+2nd+3rd+Noncoding. All positions containing gaps and missing data were eliminated. There were a total of 117 positions in the final dataset. Evolutionary analyses were conducted in MEGA7.

| | | | | | | | | |
|---|---|---|---|---|---|---|---|---|
| JQ065049-MCoV-CN | | | | | | | | |
| JQ065046-MrCoV-CN | 28 | | | | | | | |
| FJ376622-MuCoV-CN | 27 | 20 | | | | | | |
| FJ376621-TCoV-CN | 21 | 22 | 21 | | | | | |
| MF431743-PDCoV-CN | 22 | 23 | 18 | 20 | | | | |
| FJ376620-BCoV-CN | 27 | 23 | 22 | 22 | 27 | | | |
| JQ065047-NhCoV-CN | 20 | 28 | 24 | 26 | 21 | 22 | | |
| JQ065048-WCoV-CN | 30 | 32 | 33 | 32 | 32 | 30 | 26 | |
| **MAD-DCoV-RPP-117nt** | 25 | 28 | 26 | 25 | 28 | 24 | 23 | 21 |

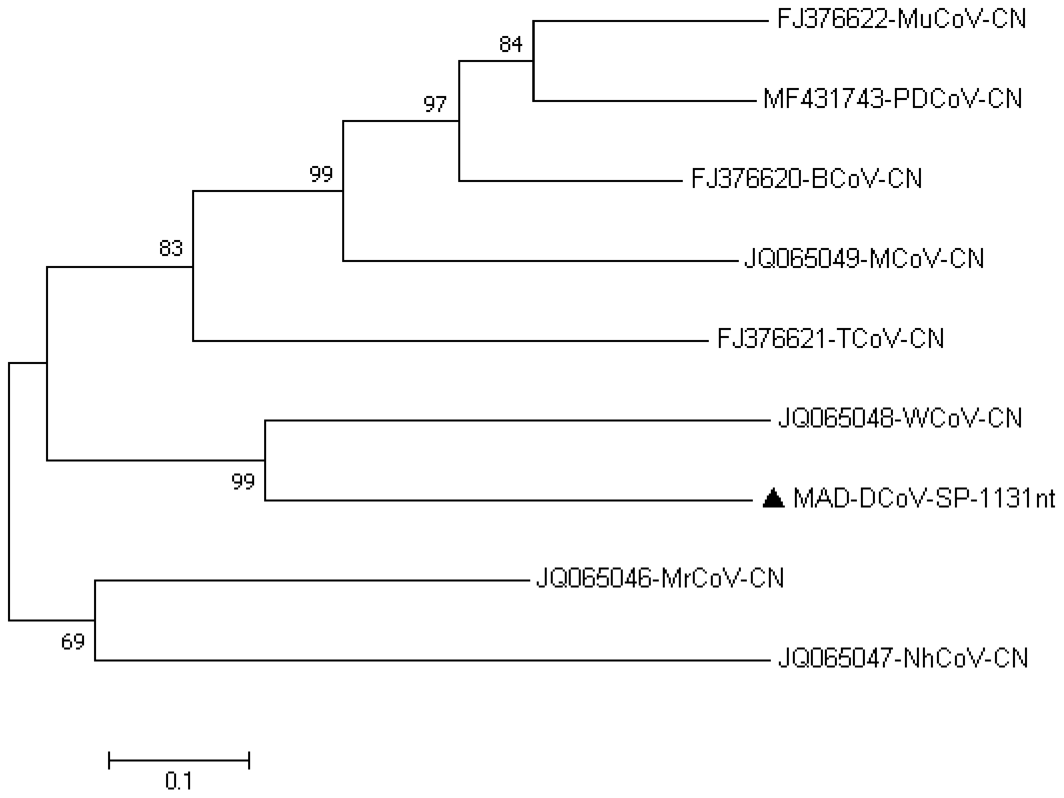


**Figure S3.5: Molecular Phylogenetic analysis by Maximum Likelihood method of DCoV partial SP gene**
The evolutionary history was inferred by using the Maximum Likelihood method based on the Tamura 3-parameter model[4]. The tree with the highest log likelihood (-8892.42) is shown. The percentage of trees in which the associated taxa clustered together is shown next to the branches. Initial tree(s) for the heuristic search were obtained automatically by applying Neighbor-Join and BioNJ algorithms to a matrix of pairwise distances estimated using the Maximum Composite Likelihood (MCL) approach, and then selecting the topology with superior log likelihood value. A discrete Gamma distribution was used to model evolutionary rate differences among sites (5 categories (+G, parameter = 1.0627)). The rate variation model allowed for some sites to be evolutionarily invariable ([+I], 26.08% sites). The tree is drawn to scale, with branch lengths measured in the number of substitutions per site. The analysis involved 9 nucleotide sequences. Codon positions included were 1st+2nd+3rd+Noncoding. All positions containing gaps and missing data were eliminated. There were a total of 1125 positions in the final dataset. Evolutionary analyses were conducted in MEGA7[2].

**Table S3.5: Estimates of Evolutionary Divergence between Sequences**
The number of base differences per sequence from between sequences are shown. The analysis involved 9 nucleotide sequences. Codon positions included were 1st+2nd+3rd+Noncoding. All positions containing gaps and missing data were eliminated. There were a total of 1125 positions in the final dataset. Evolutionary analyses were conducted in MEGA7.

| | | | | | | | | |
|---|---|---|---|---|---|---|---|---|
| JQ065049-MCoV-CN | | | | | | | | |
| JQ065046-MrCoV-CN | 421 | | | | | | | |
| FJ376622-MuCoV-CN | 347 | 405 | | | | | | |
| FJ376621-TCoV-CN | 389 | 413 | 388 | | | | | |
| MF431743-PDCoV-CN | 354 | 417 | 252 | 377 | | | | |
| FJ376620-BCoV-CN | 308 | 423 | 274 | 390 | 261 | | | |
| JQ065047-NhCoV-CN | 436 | 408 | 443 | 416 | 421 | 423 | | |
| JQ065048-WCoV-CN | 421 | 424 | 420 | 439 | 413 | 436 | 456 | |
| **MAD-DCoV-SP-1131nt** | 425 | 421 | 428 | 432 | 428 | 426 | 436 | 393 |

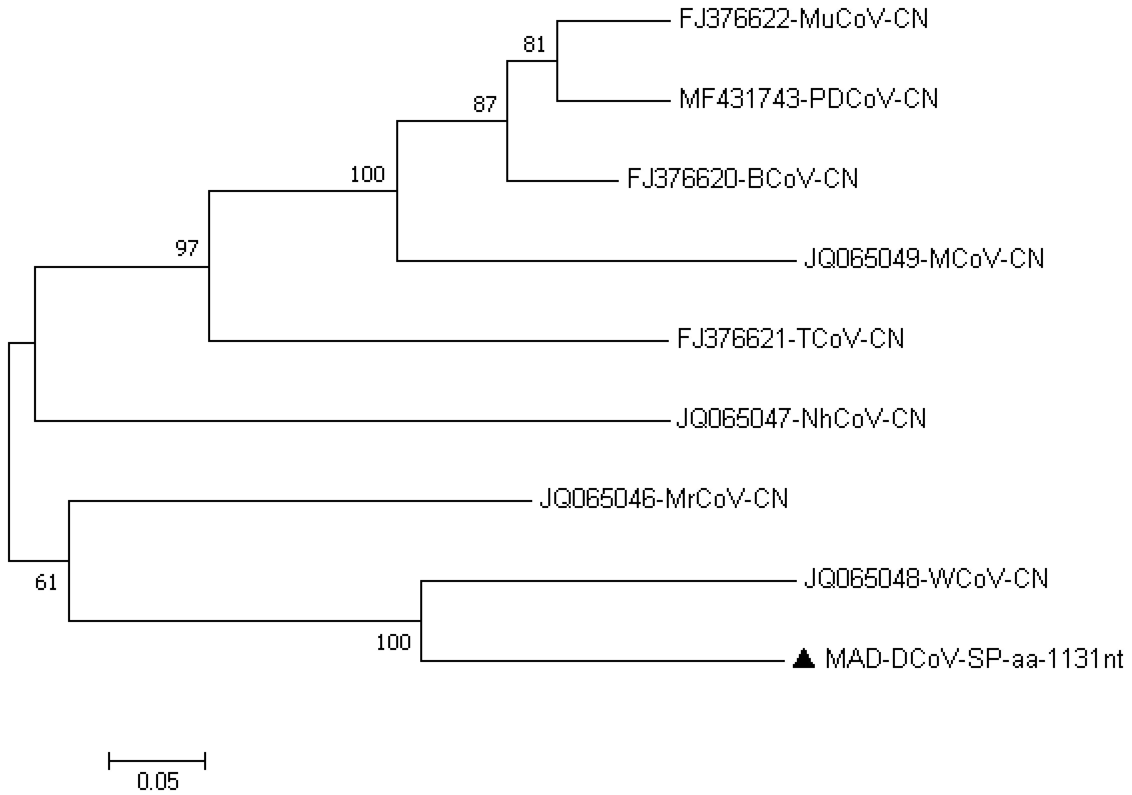


**Figure S3.6: Molecular Phylogenetic analysis by Maximum Likelihood method for amino acid sequences of DCoV partial SP gene**

The evolutionary history was inferred by using the Maximum Likelihood method based on the Le_Gascuel_2008 model[6]. The tree with the highest log likelihood (-3983.73) is shown. The percentage of trees in which the associated taxa clustered together is shown next to the branches. Initial tree(s) for the heuristic search were obtained automatically by applying Neighbor-Join and BioNJ algorithms to a matrix of pairwise distances estimated using a JTT model, and then selecting the topology with superior log likelihood value. A discrete Gamma distribution was used to model evolutionary rate differences among sites (5 categories (+G, parameter = 0.7265)). The tree is drawn to scale, with branch lengths measured in the number of substitutions per site. The analysis involved 9 amino acid sequences. All positions containing gaps and missing data were eliminated. There were a total of 375 positions in the final dataset. Evolutionary analyses were conducted in MEGA7[2].

**Table S3.6: Estimates of Evolutionary Divergence between Sequences**

The number of amino acid differences per sequence from between sequences are shown. Standard error estimate(s) are shown above the diagonal. The analysis involved 9 amino acid sequences. All positions containing gaps and missing data were eliminated. There were a total of 375 positions in the final dataset. Evolutionary analyses were conducted in MEGA7.

| | | | | | | | | |
|---|---|---|---|---|---|---|---|---|
| JQ065049-MCoV-CN | | | | | | | | |
| JQ065046-MrCoV-CN | 133 | | | | | | | |
| FJ376622-MuCoV-CN | 89 | 129 | | | | | | |
| FJ376621-TCoV-CN | 128 | 132 | 111 | | | | | |
| MF431743-PDCoV-CN | 96 | 132 | 43 | 114 | | | | |
| FJ376620-BCoV-CN | 80 | 133 | 49 | 115 | 46 | | | |
| JQ065047-NhCoV-CN | 131 | 123 | 137 | 134 | 143 | 136 | | |
| JQ065048-WCoV-CN | 149 | 130 | 141 | 147 | 135 | 140 | 144 | |
| **MAD-DCoV-SP-aa-1131nt** | 145 | 129 | 147 | 142 | 140 | 140 | 149 | 99 |

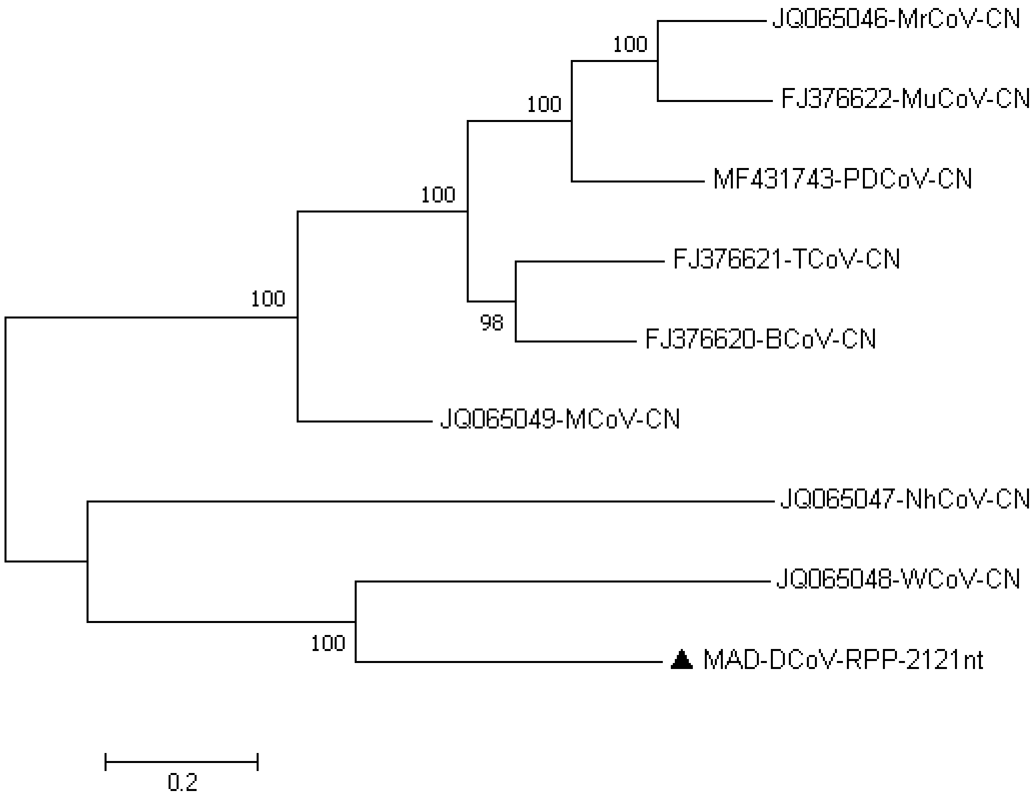


**Figure S3.7: Molecular Phylogenetic analysis by Maximum Likelihood method of DCoV partial RPP gene**
The evolutionary history was inferred by using the Maximum Likelihood method based on the General Time Reversible model[7]. The tree with the highest log likelihood (-15847.72) is shown. The percentage of trees in which the associated taxa clustered together is shown next to the branches. Initial tree(s) for the heuristic search were obtained automatically by applying Neighbor-Join and BioNJ algorithms to a matrix of pairwise distances estimated using the Maximum Composite Likelihood (MCL) approach, and then selecting the topology with superior log likelihood value. A discrete Gamma distribution was used to model evolutionary rate differences among sites (5 categories (+G, parameter = 1.0085)). The tree is drawn to scale, with branch lengths measured in the number of substitutions per site. The analysis involved 9 nucleotide sequences. All positions containing gaps and missing data were eliminated. There were a total of 2091 positions in the final dataset. Evolutionary analyses were conducted in MEGA7[2].

**Table S3.7: Estimates of Evolutionary Divergence between Sequences**
The number of base differences per sequence from between sequences are shown. The analysis involved 9 nucleotide sequences. Codon positions included were 1st+2nd+3rd+Noncoding. All positions containing gaps and missing data were eliminated. There were a total of 2091 positions in the final dataset. Evolutionary analyses were conducted in MEGA7.

| | | | | | | | | |
|---|---|---|---|---|---|---|---|---|
| JQ065048-WCoV-CN | | | | | | | | |
| JQ065049-MCoV-CN | 830 | | | | | | | |
| JQ065046-MrCoV-CN | 907 | 664 | | | | | | |
| FJ376622-MuCoV-CN | 887 | 617 | 403 | | | | | |
| FJ376621-TCoV-CN | 872 | 569 | 614 | 557 | | | | |
| MF431743-PDCoV-CN | 908 | 633 | 495 | 501 | 559 | | | |
| FJ376620-BCoV-CN | 866 | 577 | 576 | 566 | 433 | 534 | | |
| JQ065047-NhCoV-CN | 878 | 835 | 954 | 912 | 891 | 928 | 887 | |
| **MAD-DCoV-RPP-2121nt** | 707 | 804 | 908 | 895 | 843 | 918 | 859 | 872 |

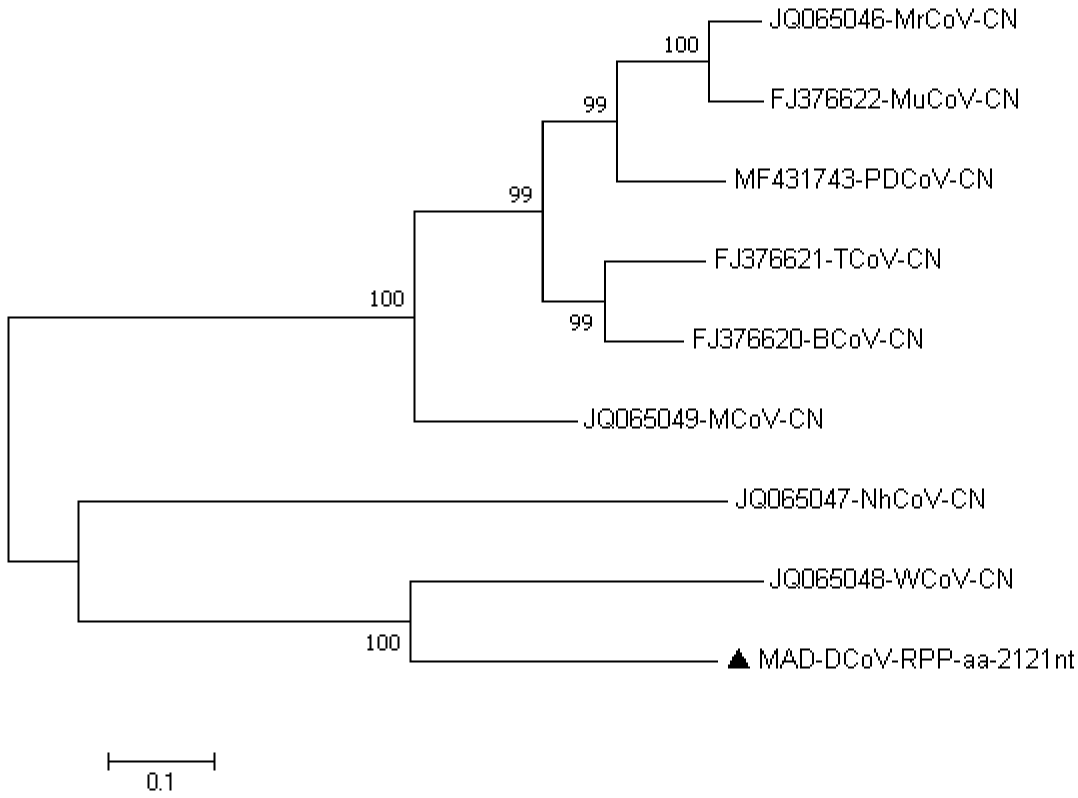


**Figure S3.8: Molecular Phylogenetic analysis by Maximum Likelihood method for amino acid sequences of DCoV partial RPP gene**

The evolutionary history was inferred by using the Maximum Likelihood method based on the Le_Gascuel_2008 model[6]. The tree with the highest log likelihood (-7469.23) is shown. The percentage of trees in which the associated taxa clustered together is shown next to the branches. Initial tree(s) for the heuristic search were obtained automatically by applying Neighbor-Join and BioNJ algorithms to a matrix of pairwise distances estimated using a JTT model, and then selecting the topology with superior log likelihood value. A discrete Gamma distribution was used to model evolutionary rate differences among sites (5 categories (+G, parameter = 0.6196)). The tree is drawn to scale, with branch lengths measured in the number of substitutions per site. The analysis involved 9 amino acid sequences. All positions containing gaps and missing data were eliminated. There were a total of 697 positions in the final dataset. Evolutionary analyses were conducted in MEGA7[2].

**Table S3.8: Estimates of Evolutionary Divergence between Sequences**

The number of amino acid differences per sequence from between sequences are shown. The analysis involved 9 amino acid sequences. All positions containing gaps and missing data were eliminated. There were a total of 697 positions in the final dataset. Evolutionary analyses were conducted in MEGA7.

| | | | | | | | | |
|---|---|---|---|---|---|---|---|---|
| JQ065048-WCoV-CN | | | | | | | | |
| JQ065049-MCoV-CN | 316 | | | | | | | |
| JQ065046-MrCoV-CN | 328 | 181 | | | | | | |
| FJ376622-MuCoV-CN | 323 | 189 | 60 | | | | | |
| FJ376621-TCoV-CN | 333 | 183 | 147 | 152 | | | | |
| MF431743-PDCoV-CN | 327 | 183 | 117 | 116 | 141 | | | |
| FJ376620-BCoV-CN | 317 | 166 | 145 | 146 | 94 | 131 | | |
| JQ065047-NhCoV-CN | 332 | 320 | 325 | 325 | 328 | 325 | 329 | |
| **MAD-DCoV-RPP-aa-2121nt** | 239 | 316 | 323 | 320 | 324 | 327 | 322 | 321 |

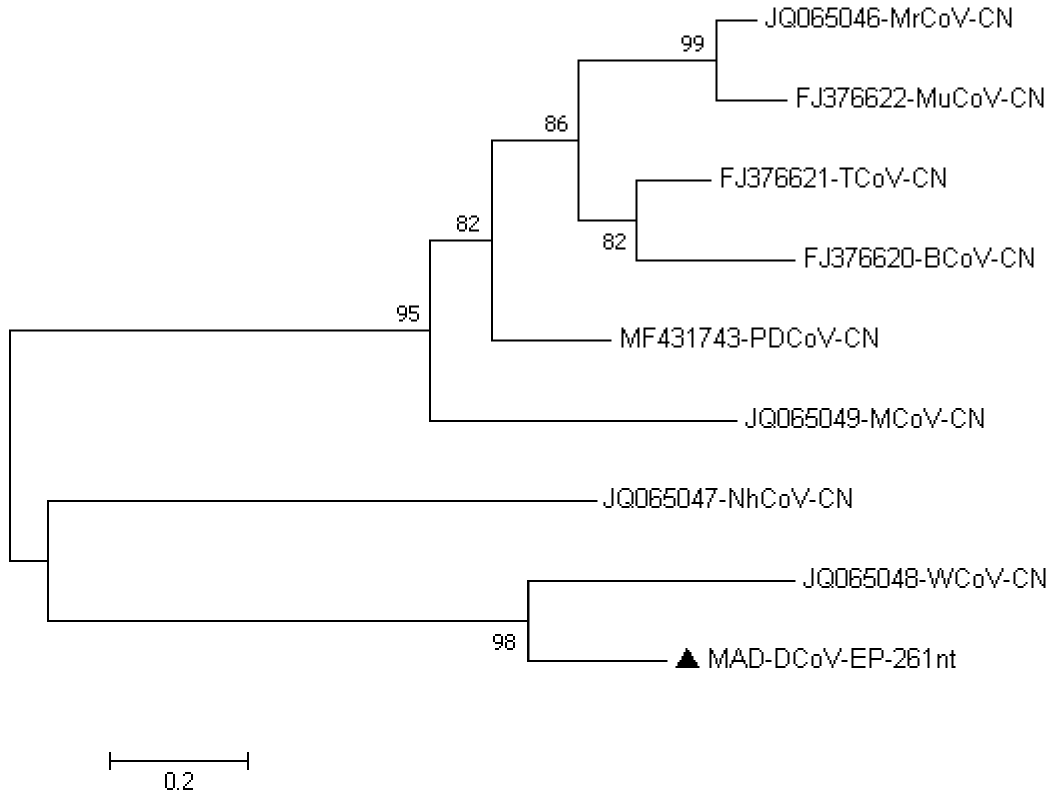


**Figure S3.9: Molecular Phylogenetic analysis by Maximum Likelihood method of DCoV partial EP gene**
The evolutionary history was inferred by using the Maximum Likelihood method based on the Tamura 3-parameter model[4]. The tree with the highest log likelihood (-1858.26) is shown. The percentage of trees in which the associated taxa clustered together is shown next to the branches. Initial tree(s) for the heuristic search were obtained automatically by applying Neighbor-Join and BioNJ algorithms to a matrix of pairwise distances estimated using the Maximum Composite Likelihood (MCL) approach, and then selecting the topology with superior log likelihood value. A discrete Gamma distribution was used to model evolutionary rate differences among sites (5 categories (+G, parameter = 1.3268)). The tree is drawn to scale, with branch lengths measured in the number of substitutions per site. The analysis involved 9 nucleotide sequences. All positions containing gaps and missing data were eliminated. There were a total of 231 positions in the final dataset. Evolutionary analyses were conducted in MEGA7[2].

**Table S3.9: Estimates of Evolutionary Divergence between Sequences**
The number of base differences per sequence from between sequences are shown. The analysis involved 9 nucleotide sequences. Codon positions included were 1st+2nd+3rd+Noncoding. All positions containing gaps and missing data were eliminated. There were a total of 231 positions in the final dataset. Evolutionary analyses were conducted in MEGA7.

| | | | | | | | | |
|---|---|---|---|---|---|---|---|---|
| JQ065049-MCoV-CN | | | | | | | | |
| JQ065046-MrCoV-CN | 86 | | | | | | | |
| FJ376622-MuCoV-CN | 80 | 30 | | | | | | |
| FJ376621-TCoV-CN | 84 | 58 | 60 | | | | | |
| MF431743-PDCoV-CN | 79 | 65 | 70 | 65 | | | | |
| FJ376620-BCoV-CN | 85 | 69 | 67 | 50 | 68 | | | |
| JQ065047-NhCoV-CN | 114 | 117 | 114 | 115 | 107 | 117 | | |
| JQ065048-WCoV-CN | 115 | 118 | 125 | 116 | 120 | 120 | 115 | |
| **MAD-DCoV-EP-261nt** | 111 | 112 | 111 | 121 | 110 | 117 | 111 | 74 |

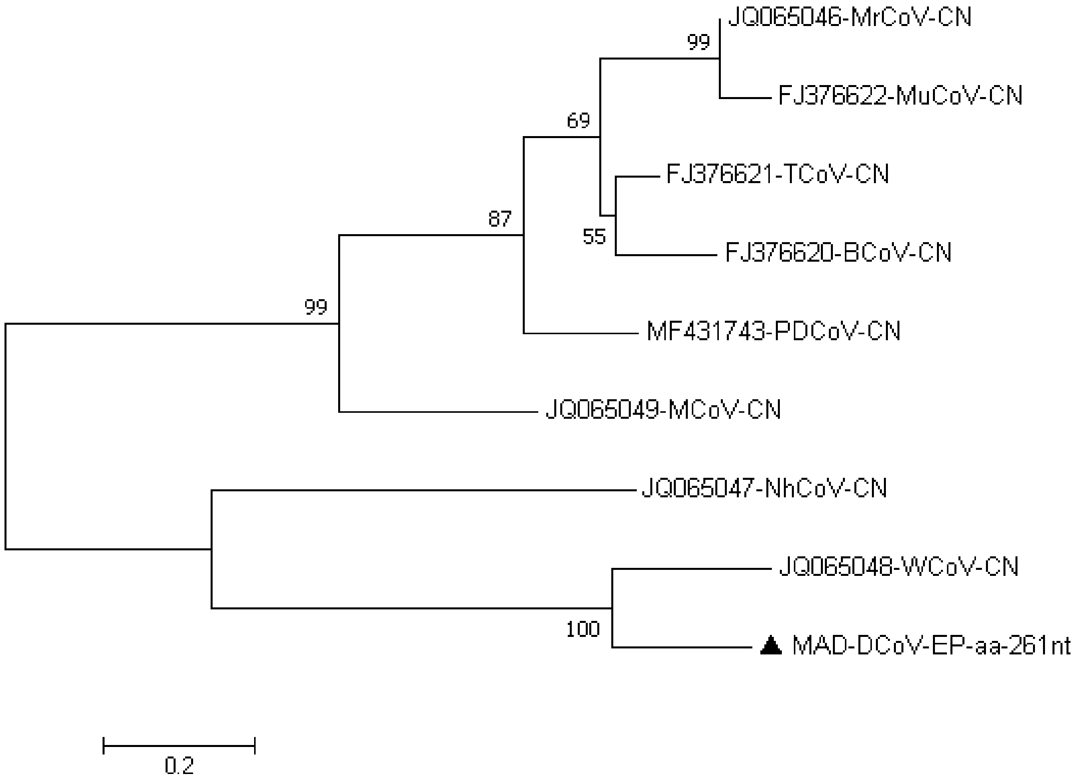


**Figure S3.10: Molecular Phylogenetic analysis by Maximum Likelihood method for amino acid sequences of DCoV partial EP gene**

The evolutionary history was inferred by using the Maximum Likelihood method based on the Le_Gascuel_2008 model[6]. The tree with the highest log likelihood (-962.16) is shown. The percentage of trees in which the associated taxa clustered together is shown next to the branches. Initial tree(s) for the heuristic search were obtained automatically by applying Neighbor-Join and BioNJ algorithms to a matrix of pairwise distances estimated using a JTT model, and then selecting the topology with superior log likelihood value. A discrete Gamma distribution was used to model evolutionary rate differences among sites (5 categories (+G, parameter = 1.9470)). The tree is drawn to scale, with branch lengths measured in the number of substitutions per site. The analysis involved 9 amino acid sequences. All positions containing gaps and missing data were eliminated. There were a total of 77 positions in the final dataset. Evolutionary analyses were conducted in MEGA7[2].

**Table S3.10: Estimates of Evolutionary Divergence between Sequences**

The number of amino acid differences per sequence from between sequences are shown. The analysis involved 9 amino acid sequences. All positions containing gaps and missing data were eliminated. There were a total of 77 positions in the final dataset. Evolutionary analyses were conducted in MEGA7.

| | | | | | | | | |
|---|---|---|---|---|---|---|---|---|
| JQ065049-MCoV-CN | | | | | | | | |
| JQ065046-MrCoV-CN | 29 | | | | | | | |
| FJ376622-MuCoV-CN | 32 | 5 | | | | | | |
| FJ376621-TCoV-CN | 27 | 14 | 17 | | | | | |
| MF431743-PDCoV-CN | 29 | 22 | 24 | 19 | | | | |
| FJ376620-BCoV-CN | 31 | 18 | 19 | 12 | 21 | | | |
| JQ065047-NhCoV-CN | 49 | 47 | 47 | 46 | 47 | 49 | | |
| JQ065048-WCoV-CN | 47 | 50 | 51 | 51 | 48 | 50 | 45 | |
| **MAD-DCoV-EP-aa-261nt** | 47 | 50 | 51 | 50 | 48 | 50 | 46 | 22 |

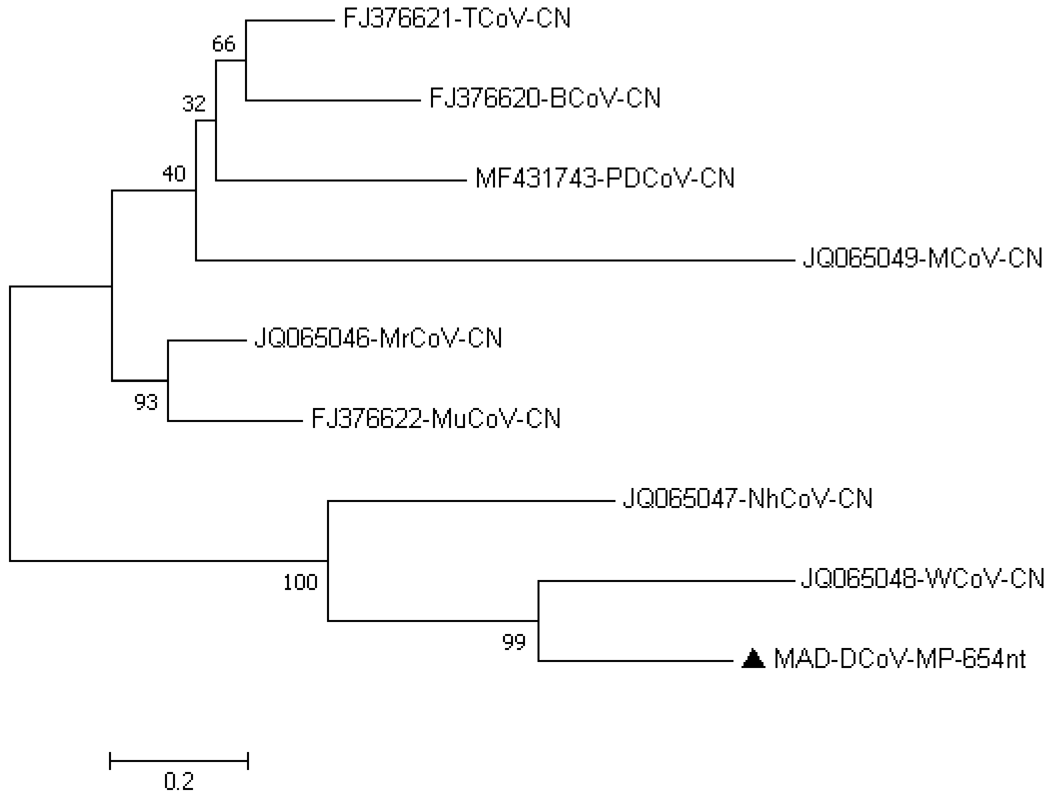


**Figure S3.11. Molecular Phylogenetic analysis by Maximum Likelihood method of DCoV partial MP gene**
The evolutionary history was inferred by using the Maximum Likelihood method based on the Tamura 3-parameter model[4]. The tree with the highest log likelihood (-5074.04) is shown. The percentage of trees in which the associated taxa clustered together is shown next to the branches. Initial tree(s) for the heuristic search were obtained automatically by applying Neighbor-Join and BioNJ algorithms to a matrix of pairwise distances estimated using the Maximum Composite Likelihood (MCL) approach, and then selecting the topology with superior log likelihood value. A discrete Gamma distribution was used to model evolutionary rate differences among sites (5 categories (+G, parameter = 1.0453)). The tree is drawn to scale, with branch lengths measured in the number of substitutions per site. The analysis involved 9 nucleotide sequences. All positions containing gaps and missing data were eliminated. There were a total of 642 positions in the final dataset. Evolutionary analyses were conducted in MEGA7[2].

**Table S3.11: Estimates of Evolutionary Divergence between Sequences**
The number of base differences per sequence from between sequences are shown. The analysis involved 9 nucleotide sequences. Codon positions included were 1st+2nd+3rd+Noncoding. All positions containing gaps and missing data were eliminated. There were a total of 642 positions in the final dataset. Evolutionary analyses were conducted in MEGA7.

| | | | | | | | | |
|---|---|---|---|---|---|---|---|---|
| JQ065049-MCoV-CN | | | | | | | | |
| JQ065046-MrCoV-CN | 251 | | | | | | | |
| FJ376622-MuCoV-CN | 261 | 128 | | | | | | |
| FJ376621-TCoV-CN | 223 | 173 | 184 | | | | | |
| MF431743-PDCoV-CN | 244 | 194 | 210 | 174 | | | | |
| FJ376620-BCoV-CN | 232 | 192 | 193 | 145 | 193 | | | |
| JQ065047-NhCoV-CN | 290 | 262 | 246 | 258 | 268 | 252 | | |
| JQ065048-WCoV-CN | 295 | 256 | 265 | 257 | 266 | 276 | 245 | |
| MAD-DCoV-MP-654nt | 285 | 273 | 252 | 265 | 281 | 271 | 231 | 195 |

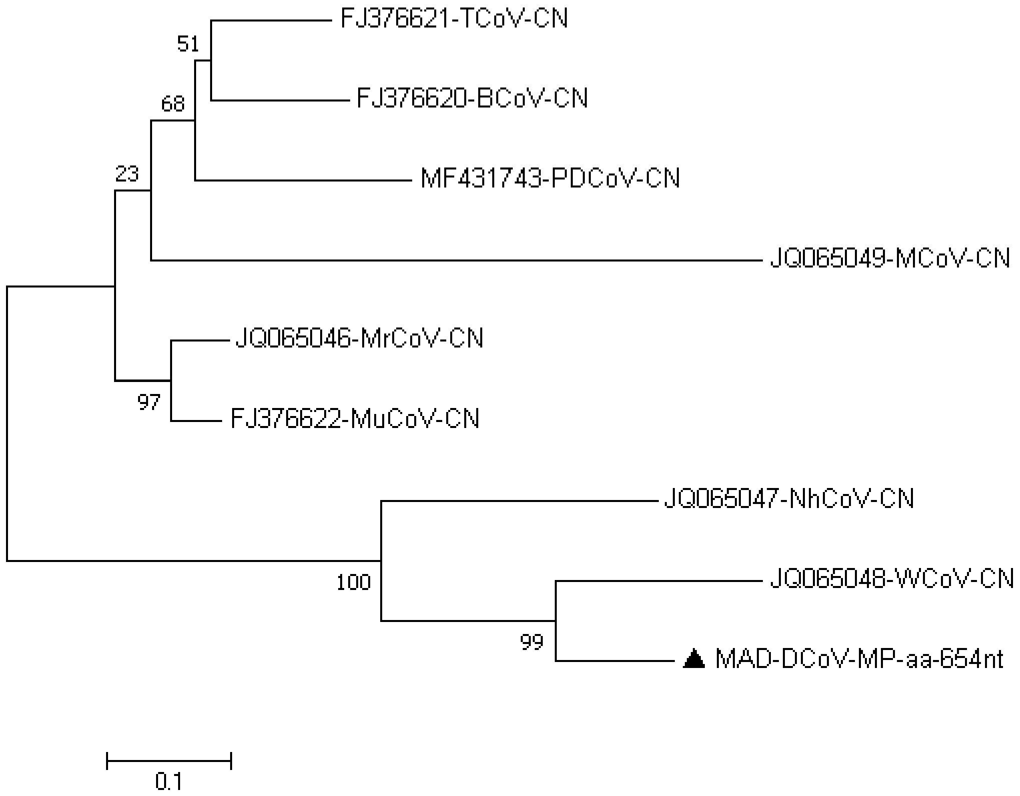


**Figure S3.12: Molecular Phylogenetic analysis by Maximum Likelihood method for amino acid sequences of DCoV partial MP gene**

The evolutionary history was inferred by using the Maximum Likelihood method based on the Le_Gascuel_2008 model[6]. The tree with the highest log likelihood (-2219.53) is shown. The percentage of trees in which the associated taxa clustered together is shown next to the branches. Initial tree(s) for the heuristic search were obtained automatically by applying Neighbor-Join and BioNJ algorithms to a matrix of pairwise distances estimated using a JTT model, and then selecting the topology with superior log likelihood value. A discrete Gamma distribution was used to model evolutionary rate differences among sites (5 categories (+G, parameter = 0.8155)). The tree is drawn to scale, with branch lengths measured in the number of substitutions per site. The analysis involved 9 amino acid sequences. All positions containing gaps and missing data were eliminated. There were a total of 214 positions in the final dataset. Evolutionary analyses were conducted in MEGA7[2].

**Table 3.12: Estimates of Evolutionary Divergence between Sequences**

The number of amino acid differences per sequence from between sequences are shown. The analysis involved 9 amino acid sequences. All positions containing gaps and missing data were eliminated. There were a total of 214 positions in the final dataset. Evolutionary analyses were conducted in MEGA7.

| | | | | | | | | |
|---|---|---|---|---|---|---|---|---|
| JQ065049-MCoV-CN | | | | | | | | |
| JQ065046-MrCoV-CN | 77 | | | | | | | |
| FJ376622-MuCoV-CN | 75 | 18 | | | | | | |
| FJ376621-TCoV-CN | 73 | 43 | 40 | | | | | |
| MF431743-PDCoV-CN | 78 | 55 | 54 | 48 | | | | |
| FJ376620-BCoV-CN | 76 | 49 | 47 | 36 | 48 | | | |
| JQ065047-NhCoV-CN | 107 | 85 | 83 | 82 | 95 | 90 | | |
| JQ065048-WCoV-CN | 104 | 92 | 95 | 89 | 96 | 97 | 72 | |
| **MAD-DCoV-MP-aa-654nt** | 102 | 86 | 82 | 88 | 99 | 89 | 70 | 46 |

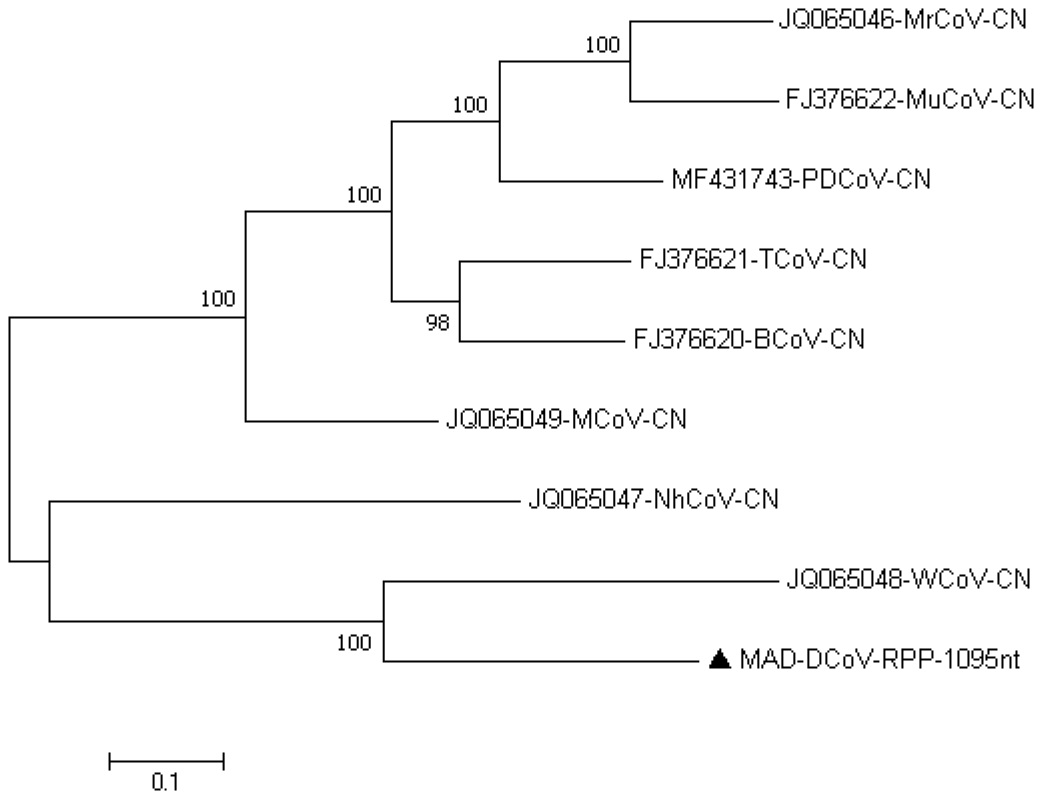


**Figure S3.13: Molecular Phylogenetic analysis by Maximum Likelihood method of DCoV partial Orf1bStart gene**

The evolutionary history was inferred by using the Maximum Likelihood method based on the Tamura 3-parameter model[4]. The tree with the highest log likelihood (-7597.55) is shown. The percentage of trees in which the associated taxa clustered together is shown next to the branches. Initial tree(s) for the heuristic search were obtained automatically by applying Neighbor-Join and BioNJ algorithms to a matrix of pairwise distances estimated using the Maximum Composite Likelihood (MCL) approach, and then selecting the topology with superior log likelihood value. A discrete Gamma distribution was used to model evolutionary rate differences among sites (5 categories (+G, parameter = 0.7931)). The rate variation model allowed for some sites to be evolutionarily invariable ([+I], 19.86% sites). The tree is drawn to scale, with branch lengths measured in the number of substitutions per site. The analysis involved 9 nucleotide sequences. All positions containing gaps and missing data were eliminated. There were a total of 1095 positions in the final dataset. Evolutionary analyses were conducted in MEGA7[2].

**Table S3.13: Estimates of Evolutionary Divergence between Sequences**

The number of base differences per sequence from between sequences are shown. The analysis involved 9 nucleotide sequences. Codon positions included were 1st+2nd+3rd+Noncoding. All positions containing gaps and missing data were eliminated. There were a total of 1095 positions in the final dataset. Evolutionary analyses were conducted in MEGA7.

| | | | | | | | | |
|---|---|---|---|---|---|---|---|---|
| JQ065049-MCoV-CN | | | | | | | | |
| JQ065046-MrCoV-CN | 312 | | | | | | | |
| FJ376622-MuCoV-CN | 297 | 195 | | | | | | |
| FJ376621-TCoV-CN | 267 | 280 | 279 | | | | | |
| MF431743-PDCoV-CN | 288 | 247 | 247 | 259 | | | | |
| FJ376620-BCoV-CN | 270 | 281 | 260 | 203 | 264 | | | |
| JQ065047-NhCoV-CN | 350 | 392 | 392 | 376 | 392 | 375 | | |
| JQ065048-WCoV-CN | 379 | 429 | 407 | 389 | 404 | 399 | 397 | |
| **MAD-DCoV-RPP-1095nt** | 373 | 433 | 419 | 395 | 423 | 402 | 378 | 317 |

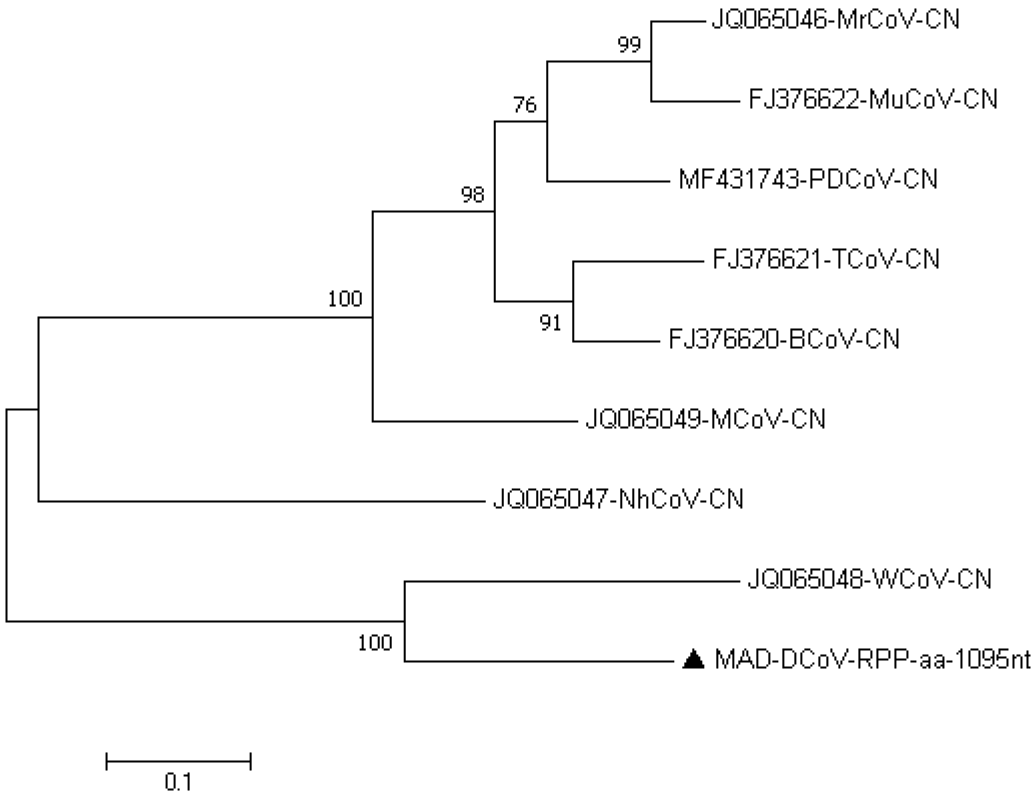


**Figure S3.14: Molecular Phylogenetic analysis by Maximum Likelihood method for amino acid sequences of DCoV partial Orf1bStart gene**

The evolutionary history was inferred by using the Maximum Likelihood method based on the Le_Gascuel_2008 model[6]. The tree with the highest log likelihood (-3466.48) is shown. The percentage of trees in which the associated taxa clustered together is shown next to the branches. Initial tree(s) for the heuristic search were obtained automatically by applying Neighbor-Join and BioNJ algorithms to a matrix of pairwise distances estimated using a JTT model, and then selecting the topology with superior log likelihood value. A discrete Gamma distribution was used to model evolutionary rate differences among sites (5 categories (+G, parameter = 0.5360)). The tree is drawn to scale, with branch lengths measured in the number of substitutions per site. The analysis involved 9 amino acid sequences. All positions containing gaps and missing data were eliminated. There were a total of 365 positions in the final dataset. Evolutionary analyses were conducted in MEGA7[2].

**Table S3.14: Estimates of Evolutionary Divergence between Sequences**

The number of amino acid differences per sequence from between sequences are shown. The analysis involved 9 amino acid sequences. All positions containing gaps and missing data were eliminated. There were a total of 365 positions in the final dataset. Evolutionary analyses were conducted in MEGA7.

| | | | | | | | | |
|---|---|---|---|---|---|---|---|---|
| JQ065049-MCoV-CN | | | | | | | | |
| JQ065046-MrCoV-CN | 72 | | | | | | | |
| FJ376622-MuCoV-CN | 75 | 28 | | | | | | |
| FJ376621-TCoV-CN | 75 | 68 | 66 | | | | | |
| MF431743-PDCoV-CN | 70 | 48 | 50 | 56 | | | | |
| FJ376620-BCoV-CN | 74 | 54 | 52 | 42 | 56 | | | |
| JQ065047-NhCoV-CN | 121 | 127 | 126 | 124 | 124 | 118 | | |
| JQ065048-WCoV-CN | 140 | 141 | 139 | 138 | 137 | 138 | 136 | |
| **MAD-DCoV-RPP-aa-1095nt** | 134 | 140 | 140 | 137 | 141 | 135 | 132 | 89 |

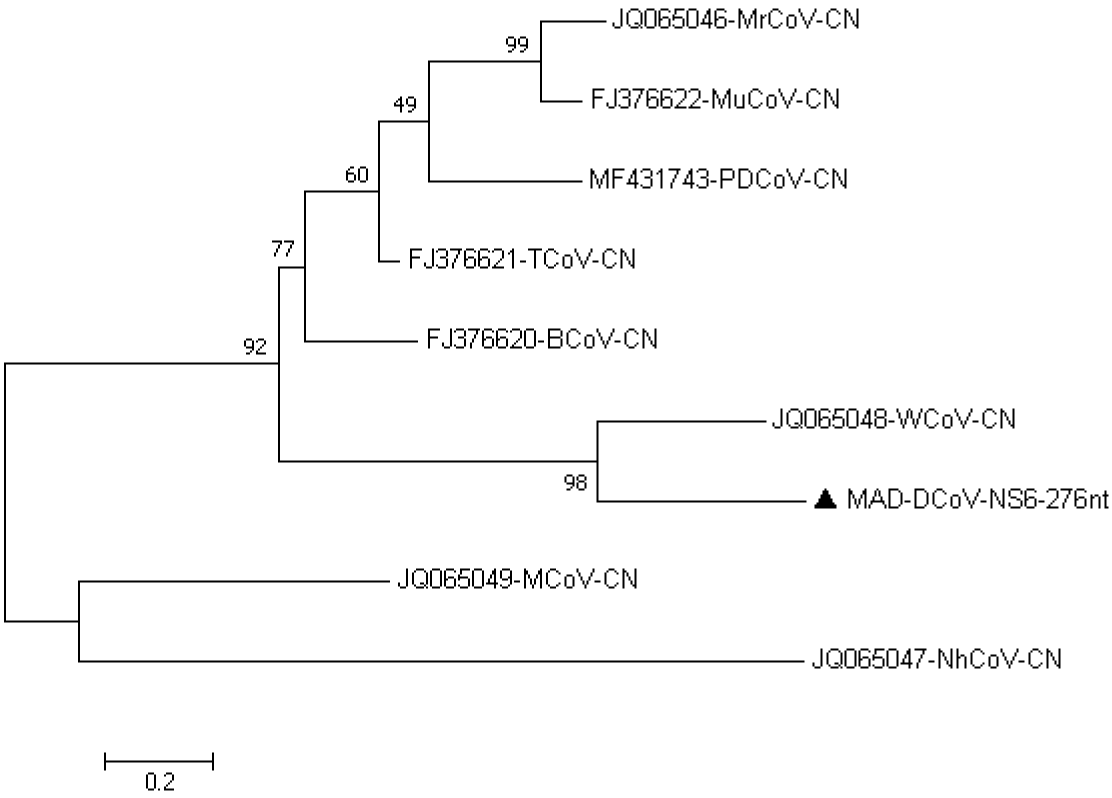


**Figure S3.15: Molecular Phylogenetic analysis by Maximum Likelihood method of DCoV partial NS6 gene**
The evolutionary history was inferred by using the Maximum Likelihood method based on the Tamura 3-parameter model[4]. The tree with the highest log likelihood (-2090.07) is shown. The percentage of trees in which the associated taxa clustered together is shown next to the branches. Initial tree(s) for the heuristic search were obtained automatically by applying Neighbor-Join and BioNJ algorithms to a matrix of pairwise distances estimated using the Maximum Composite Likelihood (MCL) approach, and then selecting the topology with superior log likelihood value. A discrete Gamma distribution was used to model evolutionary rate differences among sites (5 categories (+G, parameter = 1.9020)). The tree is drawn to scale, with branch lengths measured in the number of substitutions per site. The analysis involved 9 nucleotide sequences. All positions containing gaps and missing data were eliminated. There were a total of 240 positions in the final dataset. Evolutionary analyses were conducted in MEGA7[2].

**Table S3.15: Estimates of Evolutionary Divergence between Sequences**
The number of base differences per sequence from between sequences are shown. The analysis involved 9 nucleotide sequences. Codon positions included were 1st+2nd+3rd+Noncoding. All positions containing gaps and missing data were eliminated. There were a total of 240 positions in the final dataset. Evolutionary analyses were conducted in MEGA7.

| | | | | | | | | |
|---|---|---|---|---|---|---|---|---|
| JQ065049-MCoV-CN | | | | | | | | |
| JQ065046-MrCoV-CN | 125 | | | | | | | |
| FJ376622-MuCoV-CN | 121 | 38 | | | | | | |
| FJ376621-TCoV-CN | 119 | 69 | 62 | | | | | |
| MF431743-PDCoV-CN | 118 | 79 | 78 | 64 | | | | |
| FJ376620-BCoV-CN | 120 | 93 | 88 | 60 | 81 | | | |
| JQ065047-NhCoV-CN | 127 | 146 | 144 | 137 | 137 | 134 | | |
| JQ065048-WCoV-CN | 131 | 119 | 123 | 99 | 113 | 109 | 136 | |
| **MAD-DCoV-NS6-276nt** | 123 | 122 | 126 | 112 | 120 | 113 | 140 | 84 |

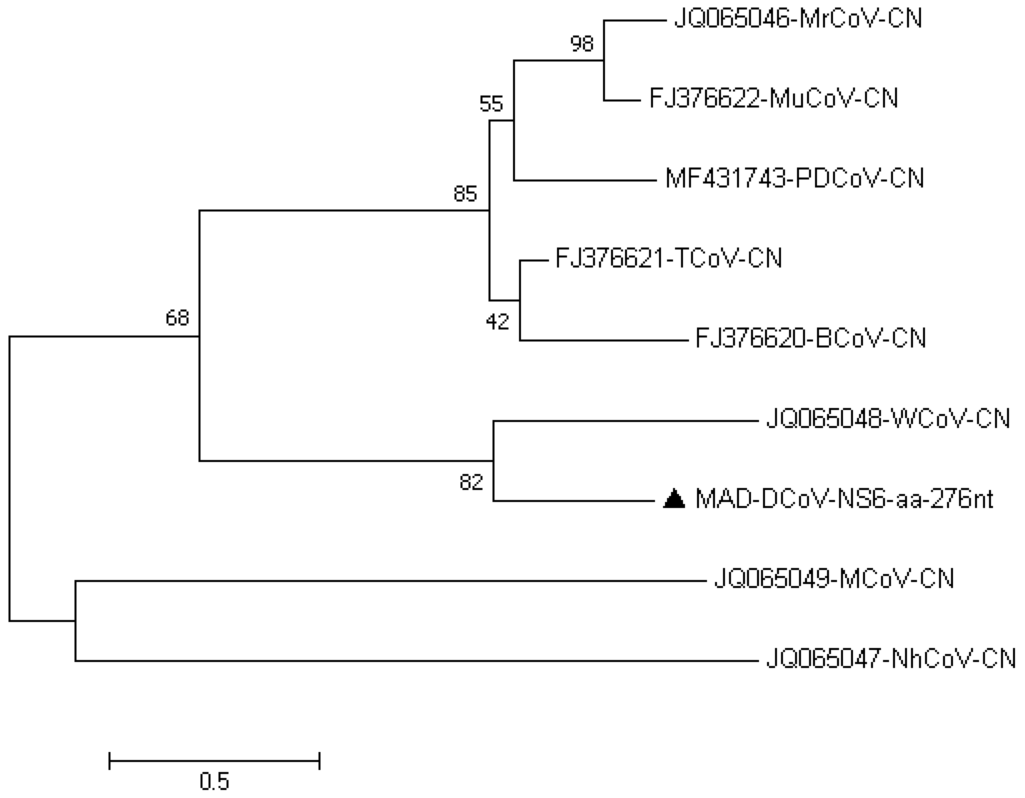


**Figure S3.16: Molecular Phylogenetic analysis by Maximum Likelihood method for amino acid sequences of DCoV partial NS6 gene**

The evolutionary history was inferred by using the Maximum Likelihood method based on the Le_Gascuel_2008 model[6]. The tree with the highest log likelihood (-1316.26) is shown. The percentage of trees in which the associated taxa clustered together is shown next to the branches. Initial tree(s) for the heuristic search were obtained automatically by applying Neighbor-Join and BioNJ algorithms to a matrix of pairwise distances estimated using a JTT model, and then selecting the topology with superior log likelihood value. A discrete Gamma distribution was used to model evolutionary rate differences among sites (5 categories (+G, parameter = 2.1381)). The tree is drawn to scale, with branch lengths measured in the number of substitutions per site. The analysis involved 9 amino acid sequences. All positions containing gaps and missing data were eliminated. There were a total of 80 positions in the final dataset. Evolutionary analyses were conducted in MEGA7[2].

**Table S3.16: Estimates of Evolutionary Divergence between Sequences**

The number of amino acid differences per sequence from between sequences are shown. The analysis involved 9 amino acid sequences. All positions containing gaps and missing data were eliminated. There were a total of 80 positions in the final dataset. Evolutionary analyses were conducted in MEGA7.

| | | | | | | | | |
|---|---|---|---|---|---|---|---|---|
| JQ065049-MCoV-CN | | | | | | | | |
| JQ065046-MrCoV-CN | 57 | | | | | | | |
| FJ376622-MuCoV-CN | 59 | 15 | | | | | | |
| FJ376621-TCoV-CN | 58 | 27 | 24 | | | | | |
| MF431743-PDCoV-CN | 57 | 27 | 28 | 25 | | | | |
| FJ376620-BCoV-CN | 60 | 38 | 35 | 24 | 32 | | | |
| JQ065047-NhCoV-CN | 60 | 58 | 61 | 58 | 57 | 57 | | |
| JQ065048-WCoV-CN | 59 | 55 | 54 | 48 | 50 | 54 | 60 | |
| **MAD-DCoV-NS6-aa-276nt** | 54 | 55 | 56 | 48 | 50 | 51 | 57 | 36 |

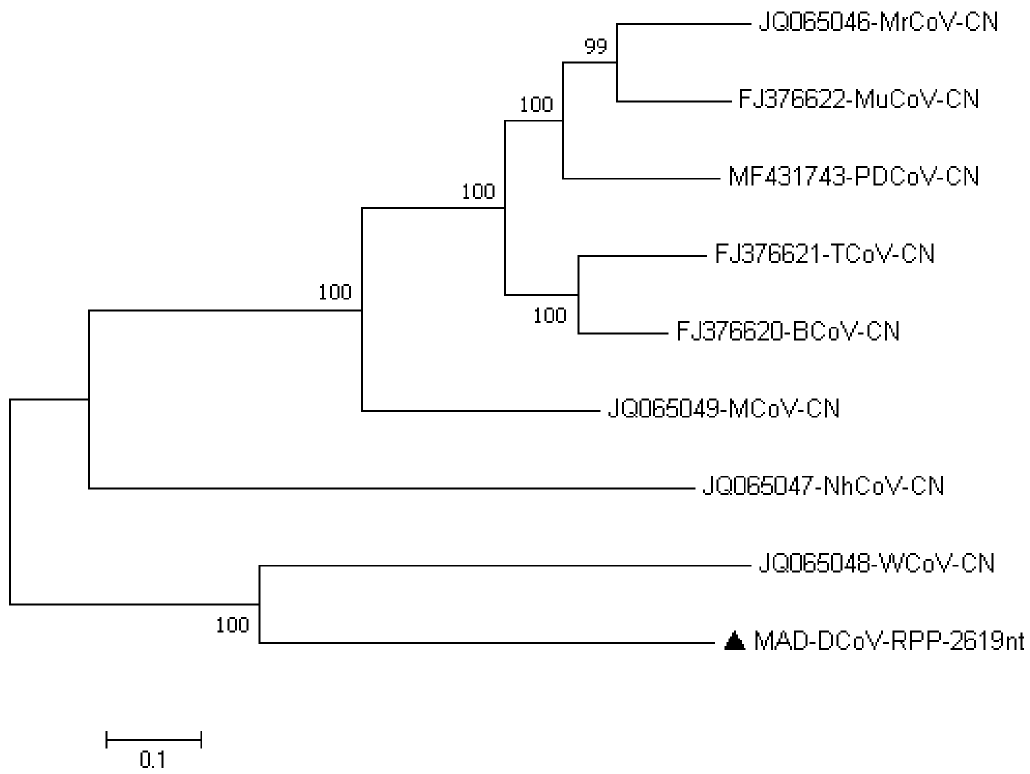


**Figure 3.17: Molecular Phylogenetic analysis by Maximum Likelihood method of DCoV partial Orf1bNSP11-13 gene**

The evolutionary history was inferred by using the Maximum Likelihood method based on the General Time Reversible model[7]. The tree with the highest log likelihood (-18412.00) is shown. The percentage of trees in which the associated taxa clustered together is shown next to the branches. Initial tree(s) for the heuristic search were obtained automatically by applying Neighbor-Join and BioNJ algorithms to a matrix of pairwise distances estimated using the Maximum Composite Likelihood (MCL) approach, and then selecting the topology with superior log likelihood value. A discrete Gamma distribution was used to model evolutionary rate differences among sites (5 categories (+G, parameter = 0.8614)). The rate variation model allowed for some sites to be evolutionarily invariable ([+I], 18.41% sites). The tree is drawn to scale, with branch lengths measured in the number of substitutions per site. The analysis involved 9 nucleotide sequences. All positions containing gaps and missing data were eliminated. There were a total of 2607 positions in the final dataset. Evolutionary analyses were conducted in MEGA7[2].

**Table S3.17: Estimates of Evolutionary Divergence between Sequences**

The number of base differences per sequence from between sequences are shown. The analysis involved 9 nucleotide sequences. Codon positions included were 1st+2nd+3rd+Noncoding. All positions containing gaps and missing data were eliminated. There were a total of 2607 positions in the final dataset. Evolutionary analyses were conducted in MEGA7.

| | | | | | | | | |
|---|---|---|---|---|---|---|---|---|
| JQ065049-MCoV-CN | | | | | | | | |
| JQ065046-MrCoV-CN | 781 | | | | | | | |
| FJ376622-MuCoV-CN | 704 | 467 | | | | | | |
| FJ376621-TCoV-CN | 698 | 604 | 609 | | | | | |
| MF431743-PDCoV-CN | 736 | 555 | 537 | 588 | | | | |
| FJ376620-BCoV-CN | 681 | 596 | 552 | 406 | 585 | | | |
| JQ065047-NhCoV-CN | 956 | 971 | 949 | 970 | 969 | 958 | | |
| JQ065048-WCoV-CN | 993 | 1061 | 1020 | 974 | 1051 | 982 | 1044 | |
| **MAD-DCoV-RPP-2619nt** | 946 | 1083 | 1040 | 1009 | 1051 | 985 | 1032 | 905 |

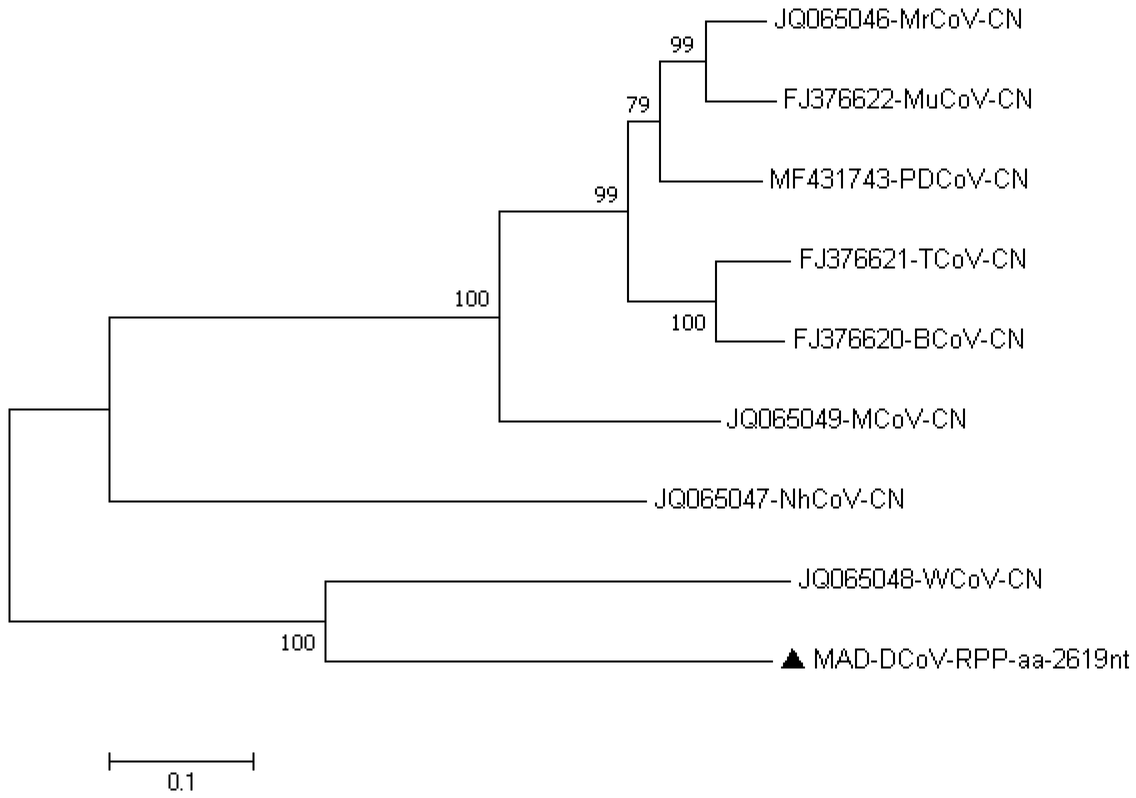


**Figure S3.18: Molecular Phylogenetic analysis by Maximum Likelihood method for amino acid sequences of DCoV partial Orf1bNSP11-13 gene**

The evolutionary history was inferred by using the Maximum Likelihood method based on the Le_Gascuel_2008 model[6]. The tree with the highest log likelihood (-8195.31) is shown. The percentage of trees in which the associated taxa clustered together is shown next to the branches. Initial tree(s) for the heuristic search were obtained automatically by applying Neighbor-Join and BioNJ algorithms to a matrix of pairwise distances estimated using a JTT model, and then selecting the topology with superior log likelihood value. A discrete Gamma distribution was used to model evolutionary rate differences among sites (5 categories (+G, parameter = 0.6597)). The tree is drawn to scale, with branch lengths measured in the number of substitutions per site. The analysis involved 9 amino acid sequences. All positions containing gaps and missing data were eliminated. There were a total of 869 positions in the final dataset. Evolutionary analyses were conducted in MEGA7[2].

**Table S3.18: Estimates of Evolutionary Divergence between Sequences**

The number of amino acid differences per sequence from between sequences are shown. The analysis involved 9 amino acid sequences. All positions containing gaps and missing data were eliminated. There were a total of 869 positions in the final dataset. Evolutionary analyses were conducted in MEGA7.

| | | | | | | | | |
|---|---|---|---|---|---|---|---|---|
| JQ065049-MCoV-CN | | | | | | | | |
| JQ065046-MrCoV-CN | 174 | | | | | | | |
| FJ376622-MuCoV-CN | 181 | 67 | | | | | | |
| FJ376621-TCoV-CN | 196 | 123 | 131 | | | | | |
| MF431743-PDCoV-CN | 183 | 99 | 101 | 121 | | | | |
| FJ376620-BCoV-CN | 194 | 116 | 131 | 73 | 120 | | | |
| JQ065047-NhCoV-CN | 317 | 315 | 312 | 313 | 306 | 315 | | |
| JQ065048-WCoV-CN | 351 | 346 | 349 | 340 | 346 | 335 | 347 | |
| **MAD-DCoV-RPP-aa-2619nt** | 351 | 356 | 365 | 358 | 353 | 351 | 336 | 283 |

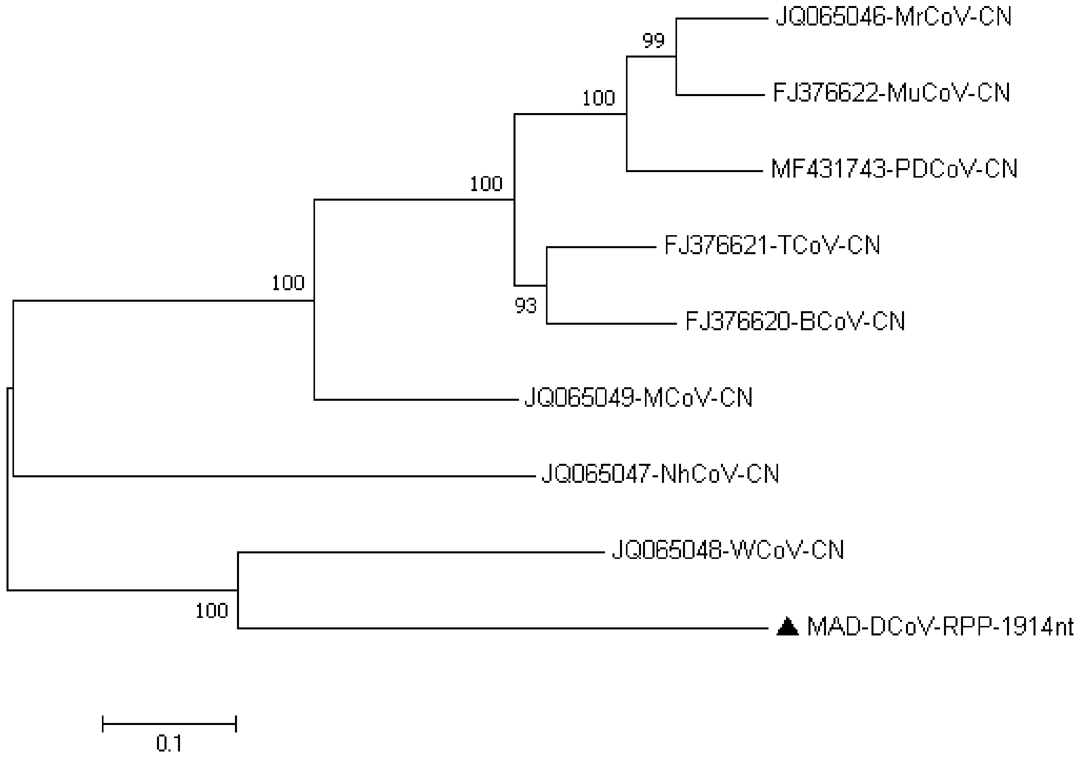


**Figure S3.19: Molecular Phylogenetic analysis by Maximum Likelihood method of DCoV partial Orf1bNSP11 gene**

The evolutionary history was inferred by using the Maximum Likelihood method based on the General Time Reversible model[7]. The tree with the highest log likelihood (-11913.38) is shown. The percentage of trees in which the associated taxa clustered together is shown next to the branches. Initial tree(s) for the heuristic search were obtained automatically by applying Neighbor-Join and BioNJ algorithms to a matrix of pairwise distances estimated using the Maximum Composite Likelihood (MCL) approach, and then selecting the topology with superior log likelihood value. A discrete Gamma distribution was used to model evolutionary rate differences among sites (5 categories (+G, parameter = 0.7350)). The rate variation model allowed for some sites to be evolutionarily invariable ([+I], 20.83% sites). The tree is drawn to scale, with branch lengths measured in the number of substitutions per site. The analysis involved 9 nucleotide sequences. All positions containing gaps and missing data were eliminated. There were a total of 1851 positions in the final dataset. Evolutionary analyses were conducted in MEGA7[2].

**Table S3.19: Estimates of Evolutionary Divergence between Sequences**

The number of base differences per sequence from between sequences are shown. The analysis involved 9 nucleotide sequences. Codon positions included were 1st+2nd+3rd+Noncoding. All positions containing gaps and missing data were eliminated. There were a total of 1851 positions in the final dataset. Evolutionary analyses were conducted in MEGA7.

| | | | | | | | | |
|---|---|---|---|---|---|---|---|---|
| JQ065049-MCoV-CN | | | | | | | | |
| JQ065046-MrCoV-CN | 481 | | | | | | | |
| FJ376622-MuCoV-CN | 468 | 206 | | | | | | |
| FJ376621-TCoV-CN | 392 | 338 | 328 | | | | | |
| MF431743-PDCoV-CN | 493 | 270 | 274 | 346 | | | | |
| FJ376620-BCoV-CN | 426 | 353 | 344 | 253 | 351 | | | |
| JQ065047-NhCoV-CN | 603 | 661 | 625 | 640 | 623 | 617 | | |
| JQ065048-WCoV-CN | 613 | 659 | 659 | 653 | 676 | 659 | 635 | |
| **MAD-DCoV-RPP-1914nt** | 634 | 680 | 675 | 665 | 701 | 658 | 659 | 559 |

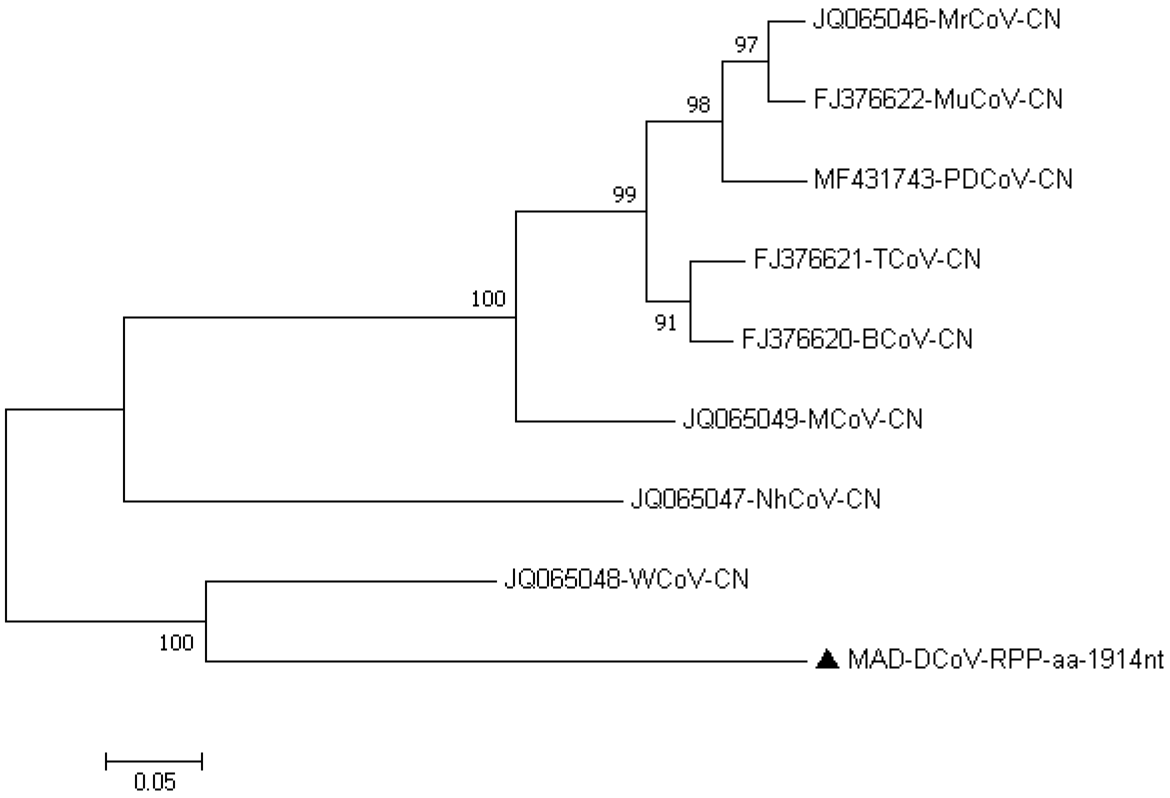


**Figure S3.20. Molecular Phylogenetic analysis by Maximum Likelihood method for amino acid sequences of DCoV partial Orf1bNSP11 gene**

The evolutionary history was inferred by using the Maximum Likelihood method based on the Le_Gascuel_2008 model[6]. The tree with the highest log likelihood (-5002.27) is shown. The percentage of trees in which the associated taxa clustered together is shown next to the branches. Initial tree(s) for the heuristic search were obtained automatically by applying Neighbor-Join and BioNJ algorithms to a matrix of pairwise distances estimated using a JTT model, and then selecting the topology with superior log likelihood value. A discrete Gamma distribution was used to model evolutionary rate differences among sites (5 categories (+G, parameter = 0.5922)). The tree is drawn to scale, with branch lengths measured in the number of substitutions per site. The analysis involved 9 amino acid sequences. All positions containing gaps and missing data were eliminated. There were a total of 617 positions in the final dataset. Evolutionary analyses were conducted in MEGA7[2].

**Table S3.20: Estimates of Evolutionary Divergence between Sequences**

The number of amino acid differences per sequence from between sequences are shown. The analysis involved 9 amino acid sequences. All positions containing gaps and missing data were eliminated. There were a total of 617 positions in the final dataset. Evolutionary analyses were conducted in MEGA7.

| | | | | | | | | |
|---|---|---|---|---|---|---|---|---|
| JQ065049-MCoV-CN | | | | | | | | |
| JQ065046-MrCoV-CN | 100 | | | | | | | |
| FJ376622-MuCoV-CN | 99 | 22 | | | | | | |
| FJ376621-TCoV-CN | 91 | 65 | 61 | | | | | |
| MF431743-PDCoV-CN | 103 | 46 | 48 | 57 | | | | |
| FJ376620-BCoV-CN | 87 | 62 | 58 | 29 | 66 | | | |
| JQ065047-NhCoV-CN | 174 | 177 | 176 | 179 | 176 | 178 | | |
| JQ065048-WCoV-CN | 181 | 191 | 186 | 187 | 193 | 178 | 188 | |
| **MAD-DCoV-RPP-aa-1914nt** | 211 | 216 | 210 | 216 | 219 | 210 | 203 | 162 |

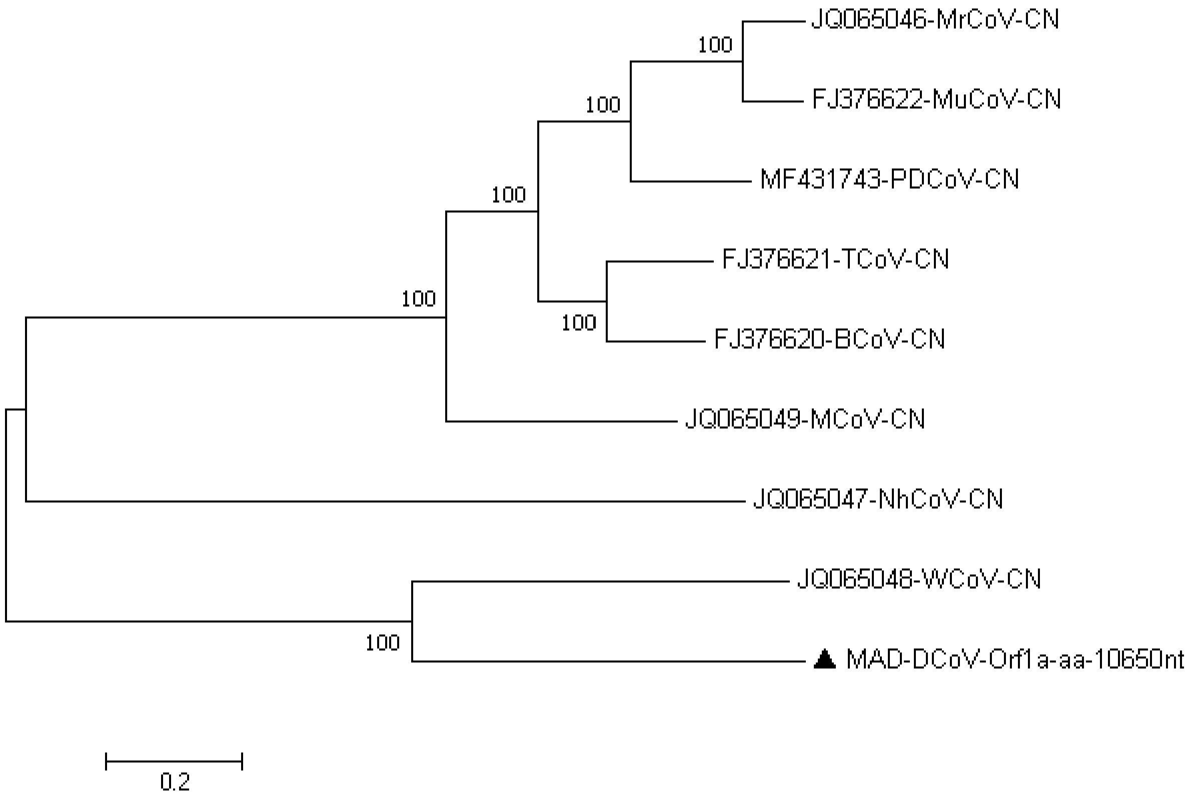


**Figure S3.21: Molecular Phylogenetic analysis by Maximum Likelihood method for amino acid sequences of DCoV Orf1a gene**

The evolutionary history was inferred by using the Maximum Likelihood method based on the Le_Gascuel_2008 model[6]. The tree with the highest log likelihood (-46030.60) is shown. The percentage of trees in which the associated taxa clustered together is shown next to the branches. Initial tree(s) for the heuristic search were obtained automatically by applying Neighbor-Join and BioNJ algorithms to a matrix of pairwise distances estimated using a JTT model, and then selecting the topology with superior log likelihood value. A discrete Gamma distribution was used to model evolutionary rate differences among sites (5 categories (+G, parameter = 1.2319)). The tree is drawn to scale, with branch lengths measured in the number of substitutions per site. The analysis involved 9 amino acid sequences. All positions containing gaps and missing data were eliminated. There were a total of 3379 positions in the final dataset. Evolutionary analyses were conducted in MEGA7[2].

**Table S3.21: Estimates of Evolutionary Divergence between Sequences**

The number of amino acid differences per sequence from between sequences are shown. The analysis involved 9 amino acid sequences. All positions containing gaps and missing data were eliminated. There were a total of 3379 positions in the final dataset. Evolutionary analyses were conducted in MEGA7.

| | | | | | | | | |
|---|---|---|---|---|---|---|---|---|
| JQ065049-MCoV-CN | | | | | | | | |
| JQ065046-MrCoV-CN | 1311 | | | | | | | |
| FJ376622-MuCoV-CN | 1312 | 479 | | | | | | |
| FJ376621-TCoV-CN | 1207 | 1120 | 1125 | | | | | |
| MF431743-PDCoV-CN | 1293 | 885 | 890 | 1063 | | | | |
| FJ376620-BCoV-CN | 1193 | 1096 | 1084 | 733 | 1034 | | | |
| JQ065047-NhCoV-CN | 2000 | 1996 | 2004 | 1997 | 1989 | 1971 | | |
| JQ065048-WCoV-CN | 2012 | 2037 | 2024 | 2043 | 2029 | 1993 | 2078 | |
| **MAD-DCoV-Orf1a-aa-10650nt** | 2012 | 2033 | 2031 | 2048 | 2045 | 2033 | 2086 | 1591 |

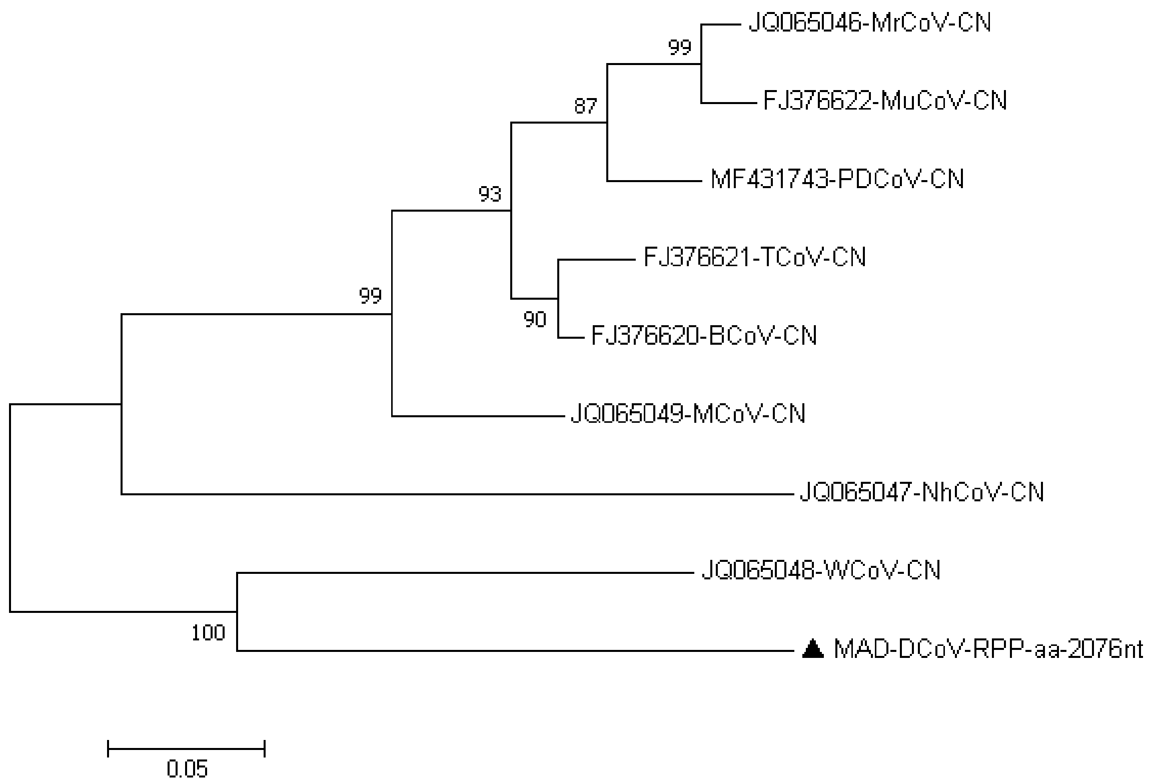


**Figure S3.22: Molecular Phylogenetic analysis by Maximum Likelihood method for amino acid sequences of DCoV partial RPP gene**

The evolutionary history was inferred by using the Maximum Likelihood method based on the Le_Gascuel_2008 model[6]. The tree with the highest log likelihood (-4534.10) is shown. The percentage of trees in which the associated taxa clustered together is shown next to the branches. Initial tree(s) for the heuristic search were obtained automatically by applying Neighbor-Join and BioNJ algorithms to a matrix of pairwise distances estimated using a JTT model, and then selecting the topology with superior log likelihood value. A discrete Gamma distribution was used to model evolutionary rate differences among sites (5 categories (+G, parameter = 0.3177)). The tree is drawn to scale, with branch lengths measured in the number of substitutions per site. The analysis involved 9 amino acid sequences. All positions containing gaps and missing data were eliminated. There were a total of 692 positions in the final dataset. Evolutionary analyses were conducted in MEGA7[2].

**Table S3.22: Estimates of Evolutionary Divergence between Sequences**

The number of amino acid differences per sequence from between sequences are shown. The analysis involved 9 amino acid sequences. All positions containing gaps and missing data were eliminated. There were a total of 692 positions in the final dataset. Evolutionary analyses were conducted in MEGA7.

| | | | | | | | | |
|---|---|---|---|---|---|---|---|---|
| JQ065049-MCoV-CN | | | | | | | | |
| JQ065046-MrCoV-CN | 72 | | | | | | | |
| FJ376622-MuCoV-CN | 71 | 18 | | | | | | |
| FJ376621-TCoV-CN | 69 | 52 | 54 | | | | | |
| MF431743-PDCoV-CN | 79 | 39 | 41 | 45 | | | | |
| FJ376620-BCoV-CN | 61 | 50 | 55 | 21 | 46 | | | |
| JQ065047-NhCoV-CN | 142 | 134 | 131 | 139 | 132 | 136 | | |
| JQ065048-WCoV-CN | 137 | 147 | 144 | 144 | 150 | 146 | 159 | |
| **MAD-DCoV-RPP-aa-2076nt** | 141 | 146 | 143 | 146 | 152 | 149 | 162 | 125 |

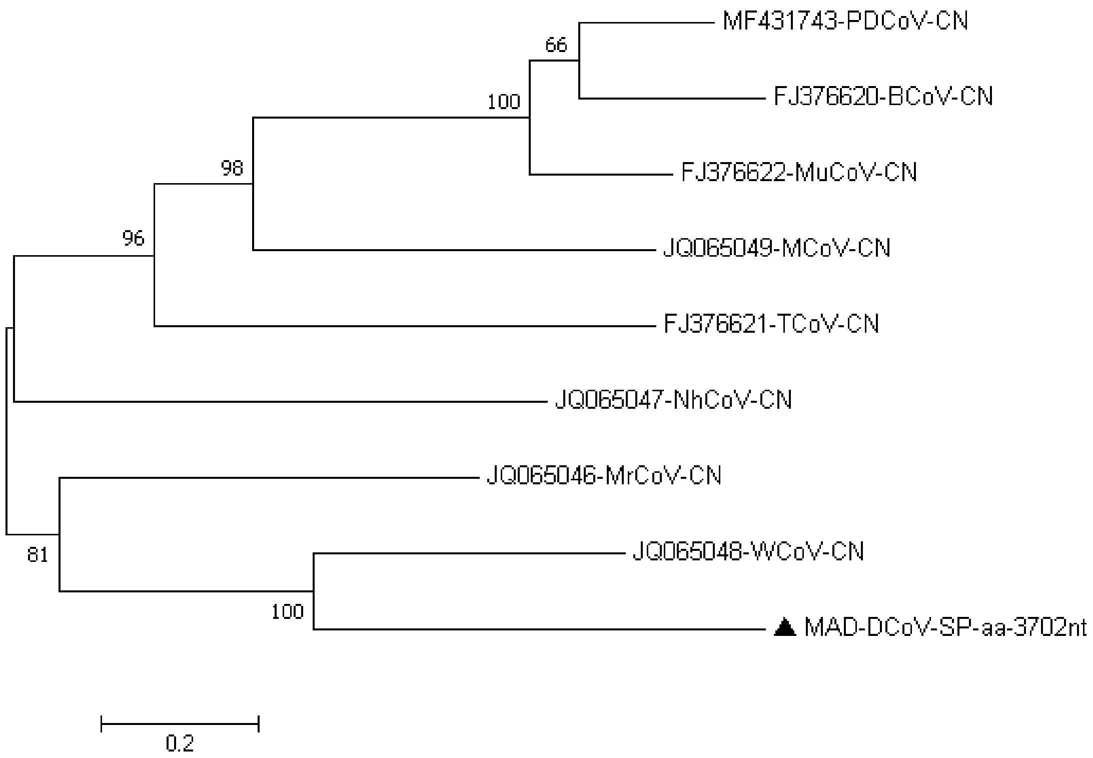


**Figure S3.23: Molecular Phylogenetic analysis by Maximum Likelihood method for amino acid sequences of DCoV partial SP gene**

The evolutionary history was inferred by using the Maximum Likelihood method based on the Whelan And Goldman + Freq. model[8]. The tree with the highest log likelihood (-16834.34) is shown. The percentage of trees in which the associated taxa clustered together is shown next to the branches. Initial tree(s) for the heuristic search were obtained automatically by applying Neighbor-Join and BioNJ algorithms to a matrix of pairwise distances estimated using a JTT model, and then selecting the topology with superior log likelihood value. A discrete Gamma distribution was used to model evolutionary rate differences among sites (5 categories (+G, parameter = 1.2876)). The rate variation model allowed for some sites to be evolutionarily invariable ([+I], 12.14% sites). The tree is drawn to scale, with branch lengths measured in the number of substitutions per site. The analysis involved 9 amino acid sequences. All positions containing gaps and missing data were eliminated. There were a total of 1104 positions in the final dataset. Evolutionary analyses were conducted in MEGA7[2].

**Table S3.23: Estimates of Evolutionary Divergence between Sequences**

The number of amino acid differences per sequence from between sequences are shown. The analysis involved 9 amino acid sequences. All positions containing gaps and missing data were eliminated. There were a total of 1104 positions in the final dataset. Evolutionary analyses were conducted in MEGA7.

| | | | | | | | | |
|---|---|---|---|---|---|---|---|---|
| JQ065049-MCoV-CN | | | | | | | | |
| JQ065046-MrCoV-CN | 568 | | | | | | | |
| FJ376622-MuCoV-CN | 509 | 576 | | | | | | |
| FJ376621-TCoV-CN | 566 | 581 | 553 | | | | | |
| MF431743-PDCoV-CN | 526 | 583 | 302 | 554 | | | | |
| FJ376620-BCoV-CN | 496 | 585 | 333 | 559 | 303 | | | |
| JQ065047-NhCoV-CN | 582 | 559 | 609 | 586 | 609 | 620 | | |
| JQ065048-WCoV-CN | 590 | 555 | 585 | 618 | 589 | 599 | 605 | |
| **MAD-DCoV-SP-aa-3072nt** | 618 | 577 | 626 | 622 | 612 | 632 | 624 | 504 |

4. **Virus related to goose adenovirus 4 (GoA4) and/or duck adenovirus 2 (DuA2) from MAD faecal sample**

| Figure and Table | Long Name (Format: Sample-virus-protein-length-quality-coverage-year) | Short name (Format: Sample-virus-protein-length-year) | Coverage | No. of nucleotides | Mapping quality threshold | NCBI accession number |
|---|---|---|---|---|---|---|
| DuA2 | | | | | | |
| S4.1 | MAD-Adenovirus-52K-153nt-Q20-C-3-10-2016 | MAD-AV-52K-153nt | 3-10 | 153 | 20 | MH028882 |
| S4.2 | MAD-Adenovirus-Hexon-179nt-Q20-C-4-25-2016 | MAD-AV-Hexon-179nt | 4-25 | 179 | 20 | MH028883 |
| S4.3 | MAD-Adenovirus-Hexon-200nt-Q20-C-4-23-2016 | MAD-AV-Hexon-200nt | 4-23 | 200 | 20 | MH028884 |
| GoA4 | | | | | | |
| S4.4 | MAD-Adenovirus-DNA-Polymerase-176nt-Q20-C-3-11-2016 | MAD-AV-Pol-176nt | 3-11 | 176 | 20 | MH028875 |
| S4.5 | MAD-Adenovirus-DNA-Polymerase-144nt-Q20-C-8-180-2016 | MAD-AV-Pol-144nt | 8-180 | 144 | 20 | MH028876 |
| S4.6 | MAD-Adenovirus-III-171nt-Q20-C-3-27-2016 | MAD-AV-III-171nt | 3-27 | 171 | 20 | MH028877 |
| S4.7 | MAD-Adenovirus-Hexon-157nt-Q20-C-7-78-2016 | MAD-AV-Hexon-157nt | 7-78 | 157 | 20 | MH028878 |
| S4.8 | MAD-Adenovirus-Hexon-164nt-Q20-C-3-15-2016 | MAD-AV-Hexon-164nt | 3-15 | 164 | 20 | MH028879 |
| S4.9 | MAD-Adenovirus-Hexon-191nt-Q20-C-7-16-2016 | MAD-AV-Hexon-191nt | 7-16 | 191 | 20 | MH028880 |
| S4.10 | MAD-Adenovirus-Hexon-228nt-Q20-C-4-39-2016 | MAD-AV-Hexon-228nt | 4-39 | 228 | ≤20 | MH028881 |
| NCBI sequences taken for phylogenetic analysis | | | | | | |
| Long Name | | Short Name (Format: NCBI accession number-virus-country/state) | Country of collection | Collection date | | |

| | | | |
|---|---|---|---|
| KR135164-Duck-adenovirus-2-strain-CH-GD-12-2014-complete-genome | KR135164-DAd2-CN | China | 2014 |
| JF510462-Goose-adenovirus-4-strain-P29-complete-genome | JF510462-GAd4-HU | Hungary | - |
| FN824512-Pigeon-adenovirus-1-complete-genome-strain-IDA4 | FN824512-PAd1-NL | Netherlands | 1995 |
| KC493646-Fowl-adenovirus-5-strain-340-complete-genome | KC493646-FAd5-IE | Ireland | 1970 |

The table points towards the molecular phylogenetic analysis by Maximum Likelihood method of AV. The first half of the table provides details to identify the sample from which the virus was isolated, protein that encodes the consensus sequences that are being analysed, the length of the consensus sequences thus providing the depth of the gene being analysed, minimum mapping quality threshold used in IGV for the generation of the consensus sequences, the coverage of the consensus sequences from IGV, the year of collection of the sample and the NCBI accession number assigned to the particular consensus sequence. Corresponding short names have been used in the phylogenetic trees which contain the sample, virus, protein and the number of nucleotides. The second half of the table provides the details of the sequences that were used for the molecular phylogenetic analysis. They were found to be the most closely related sequences to the consensus sequences generated using BLASTN. Corresponding short names have been used in the phylogenetic trees which contain the NCBI accession number, virus and the country of collection. The collection date is given as retrieved from the corresponding NCBI nucleotide reference dataset which together with the country of collection can provide an insight into the possible evolution of the virus through the years.

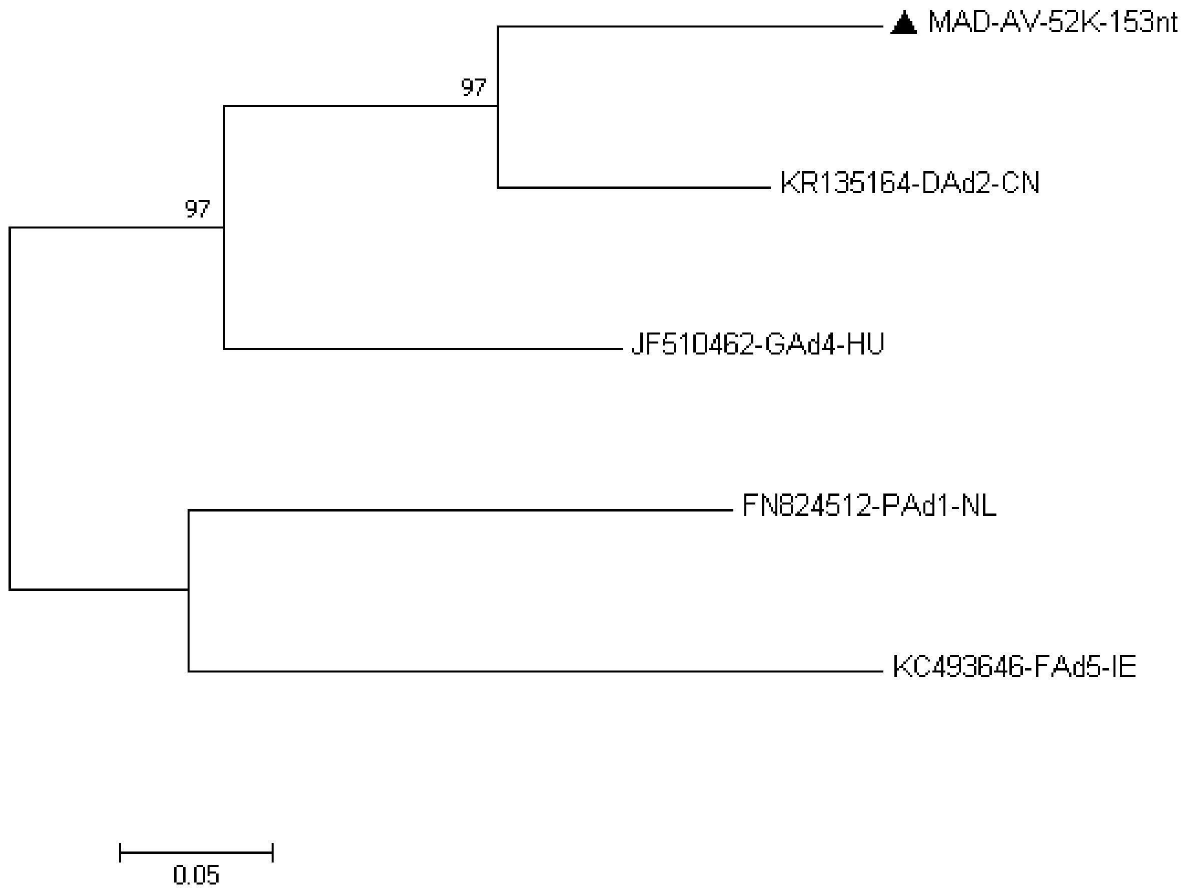


**Figure S4.1: Molecular Phylogenetic analysis by Maximum Likelihood method of AV partial 52K gene**
The evolutionary history was inferred by using the Maximum Likelihood method based on the Jukes-Cantor model[9]. The tree with the highest log likelihood (-674.58) is shown. The percentage of trees in which the associated taxa clustered together is shown next to the branches. Initial tree(s) for the heuristic search were obtained automatically by applying Neighbor-Join and BioNJ algorithms to a matrix of pairwise distances estimated using the Maximum Composite Likelihood (MCL) approach, and then selecting the topology with superior log likelihood value. The rate variation model allowed for some sites to be evolutionarily invariable ([+I], 24.51% sites). The tree is drawn to scale, with branch lengths measured in the number of substitutions per site. The analysis involved 5 nucleotide sequences. All positions containing gaps and missing data were eliminated. There were a total of 153 positions in the final dataset. Evolutionary analyses were conducted in MEGA7[2].

**Table S4.1: Estimates of Evolutionary Divergence between Sequences**
The number of base differences per sequence from between sequences are shown. The analysis involved 5 nucleotide sequences. Codon positions included were 1st+2nd+3rd+Noncoding. All positions containing gaps and missing data were eliminated. There were a total of 153 positions in the final dataset. Evolutionary analyses were conducted in MEGA7.

| **MAD-AV-52K-153nt** | | | | |
|---|---|---|---|---|
| KR135164-DAd2-CN | 26 | | | |
| JF510462-GAd4-HU | 37 | 35 | | |
| FN824512-PAd1-NL | 50 | 48 | 43 | |
| KC493646-FAd5-IE | 51 | 50 | 51 | 43 |

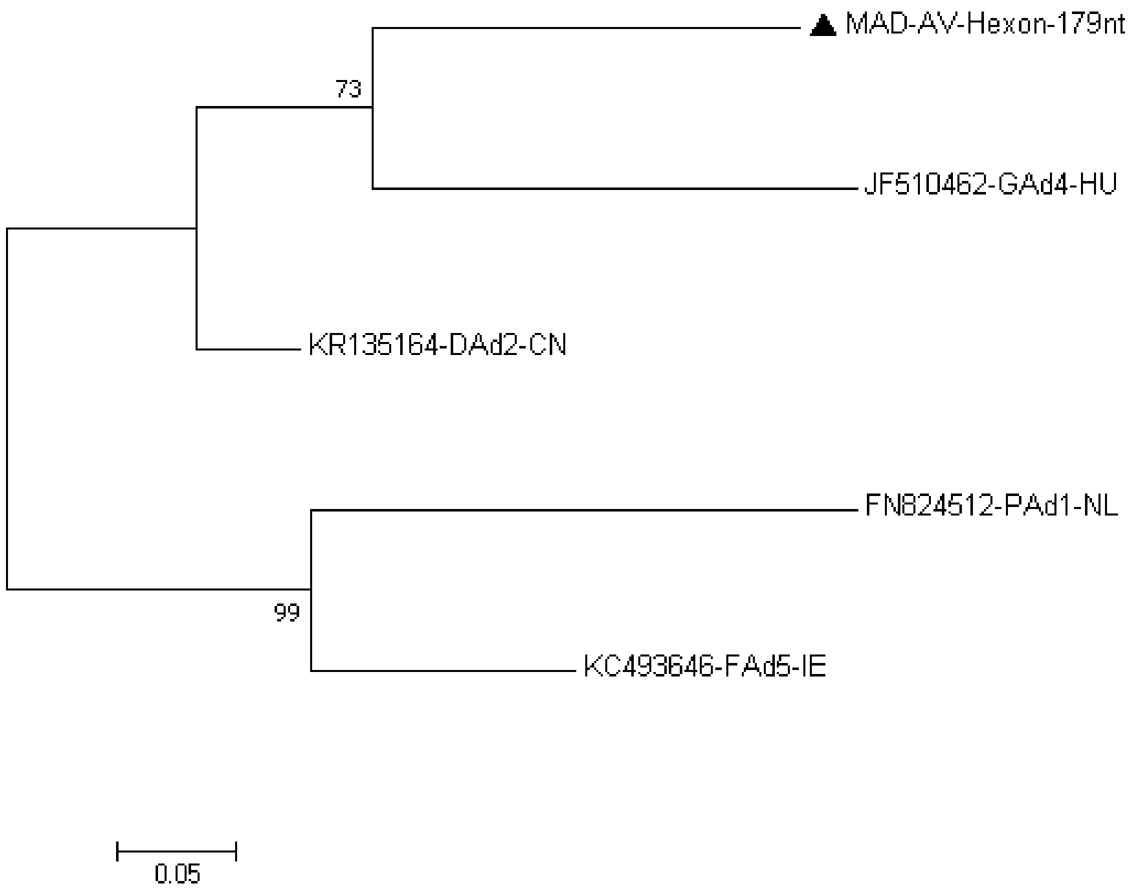


**Figure S4.2: Molecular Phylogenetic analysis by Maximum Likelihood method of AV partial Hexon gene**
The evolutionary history was inferred by using the Maximum Likelihood method based on the Kimura 2-parameter model[1]. The tree with the highest log likelihood (-693.56) is shown. The percentage of trees in which the associated taxa clustered together is shown next to the branches. Initial tree(s) for the heuristic search were obtained automatically by applying Neighbor-Join and BioNJ algorithms to a matrix of pairwise distances estimated using the Maximum Composite Likelihood (MCL) approach, and then selecting the topology with superior log likelihood value. The rate variation model allowed for some sites to be evolutionarily invariable ([+I], 30.17% sites). The tree is drawn to scale, with branch lengths measured in the number of substitutions per site. The analysis involved 5 nucleotide sequences. All positions containing gaps and missing data were eliminated. There were a total of 179 positions in the final dataset. Evolutionary analyses were conducted in MEGA7[2].

**Table S4.2: Estimates of Evolutionary Divergence between Sequences**
The number of base differences per sequence from between sequences are shown. The analysis involved 5 nucleotide sequences. Codon positions included were 1st+2nd+3rd+Noncoding. All positions containing gaps and missing data were eliminated. There were a total of 179 positions in the final dataset. Evolutionary analyses were conducted in MEGA7.

| **MAD-AV-Hexon-179nt** | | | | |
|---|---|---|---|---|
| KR135164-DAd2-CN | 34 | | | |
| JF510462-GAd4-HU | 36 | 38 | | |
| FN824512-PAd1-NL | 57 | 43 | 50 | |
| KC493646-FAd5-IE | 49 | 37 | 44 | 34 |

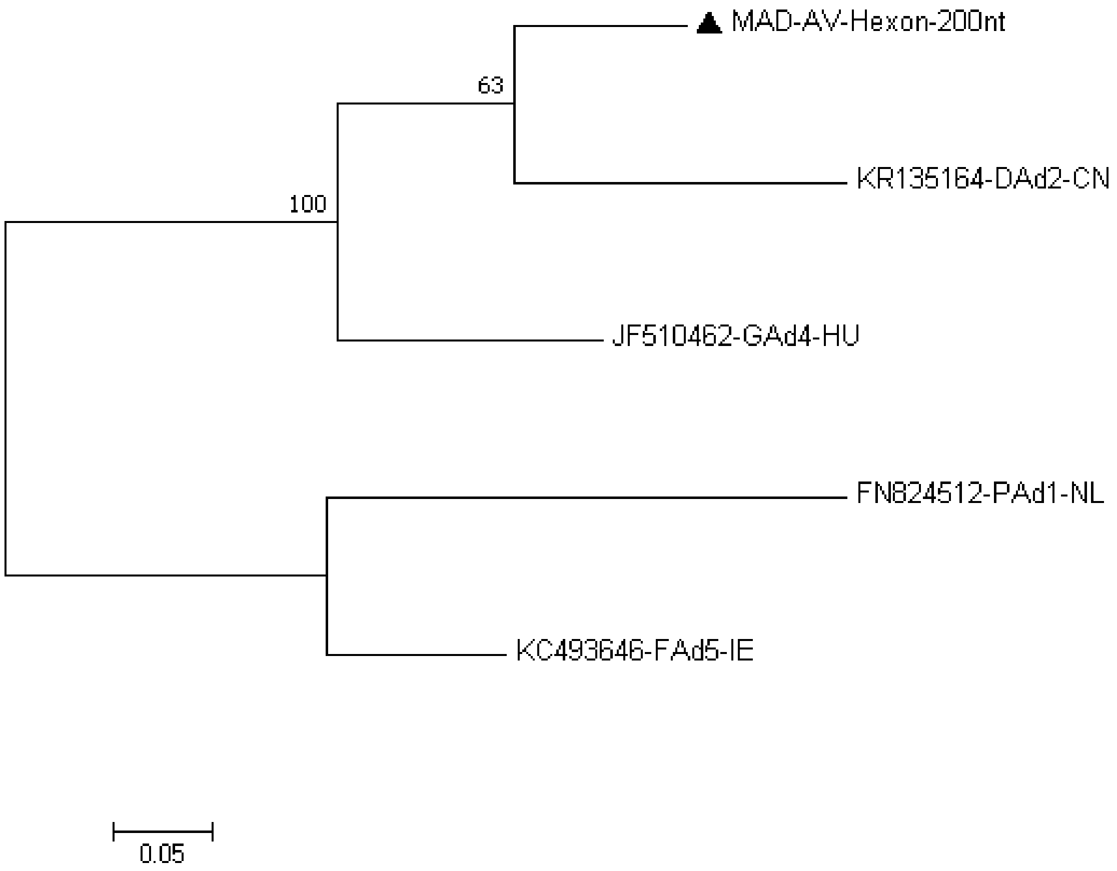


**Figure S4.3: Molecular Phylogenetic analysis by Maximum Likelihood method of AV partial Hexon gene**
The evolutionary history was inferred by using the Maximum Likelihood method based on the Kimura 2-parameter model[1]. The tree with the highest log likelihood (-845.82) is shown. The percentage of trees in which the associated taxa clustered together is shown next to the branches. Initial tree(s) for the heuristic search were obtained automatically by applying Neighbor-Join and BioNJ algorithms to a matrix of pairwise distances estimated using the Maximum Composite Likelihood (MCL) approach, and then selecting the topology with superior log likelihood value. The rate variation model allowed for some sites to be evolutionarily invariable ([+I], 27.25% sites). The tree is drawn to scale, with branch lengths measured in the number of substitutions per site. The analysis involved 5 nucleotide sequences. All positions containing gaps and missing data were eliminated. There were a total of 200 positions in the final dataset. Evolutionary analyses were conducted in MEGA7[2].

**Table S4.3: Estimates of Evolutionary Divergence between Sequences**
The number of base differences per sequence from between sequences are shown. The analysis involved 5 nucleotide sequences. Codon positions included were 1st+2nd+3rd+Noncoding. All positions containing gaps and missing data were eliminated. There were a total of 200 positions in the final dataset. Evolutionary analyses were conducted in MEGA7.

| **MAD-AV-Hexon-200nt** | | | | |
|---|---|---|---|---|
| KR135164-DAd2-CN | 37 | | | |
| JF510462-GAd4-HU | 41 | 49 | | |
| FN824512-PAd1-NL | 68 | 64 | 71 | |
| KC493646-FAd5-IE | 61 | 63 | 58 | 44 |

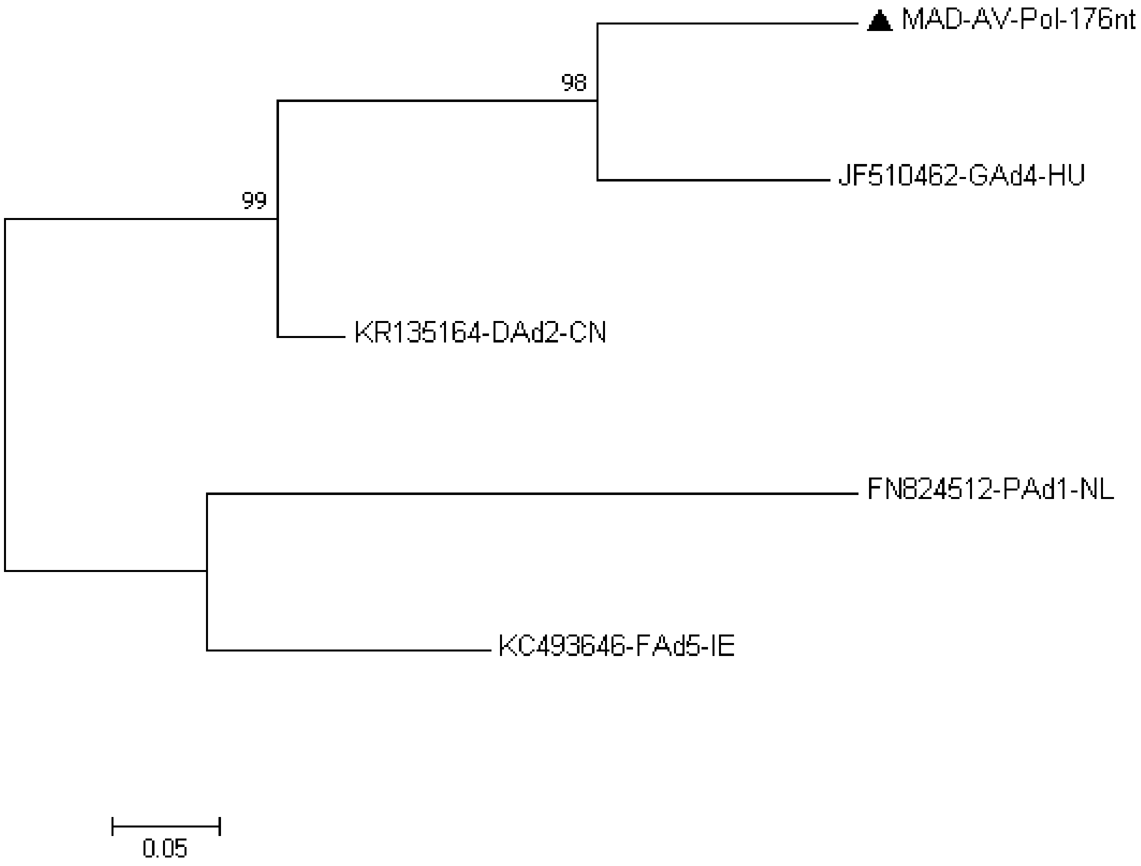


**Figure S4.4: Molecular Phylogenetic analysis by Maximum Likelihood method of AV partial Pol gene**
The evolutionary history was inferred by using the Maximum Likelihood method based on the Kimura 2-parameter model[1]. The tree with the highest log likelihood (-747.36) is shown. The percentage of trees in which the associated taxa clustered together is shown next to the branches. Initial tree(s) for the heuristic search were obtained automatically by applying Neighbor-Join and BioNJ algorithms to a matrix of pairwise distances estimated using the Maximum Composite Likelihood (MCL) approach, and then selecting the topology with superior log likelihood value. The rate variation model allowed for some sites to be evolutionarily invariable ([+I], 25.85% sites). The tree is drawn to scale, with branch lengths measured in the number of substitutions per site. The analysis involved 5 nucleotide sequences. All positions containing gaps and missing data were eliminated. There were a total of 176 positions in the final dataset. Evolutionary analyses were conducted in MEGA7[2].

**Table S4.4: Estimates of Evolutionary Divergence between Sequences**
The number of base differences per sequence from between sequences are shown. The analysis involved 5 nucleotide sequences. Codon positions included were 1st+2nd+3rd+Noncoding. All positions containing gaps and missing data were eliminated. There were a total of 176 positions in the final dataset. Evolutionary analyses were conducted in MEGA7.

| **MAD-AV-Pol-176nt** | | | | |
|---|---|---|---|---|
| KR135164-DAd2-CN | 38 | | | |
| JF510462-GAd4-HU | 31 | 37 | | |
| FN824512-PAd1-NL | 65 | 56 | 59 | |
| KC493646-FAd5-IE | 57 | 44 | 56 | 47 |

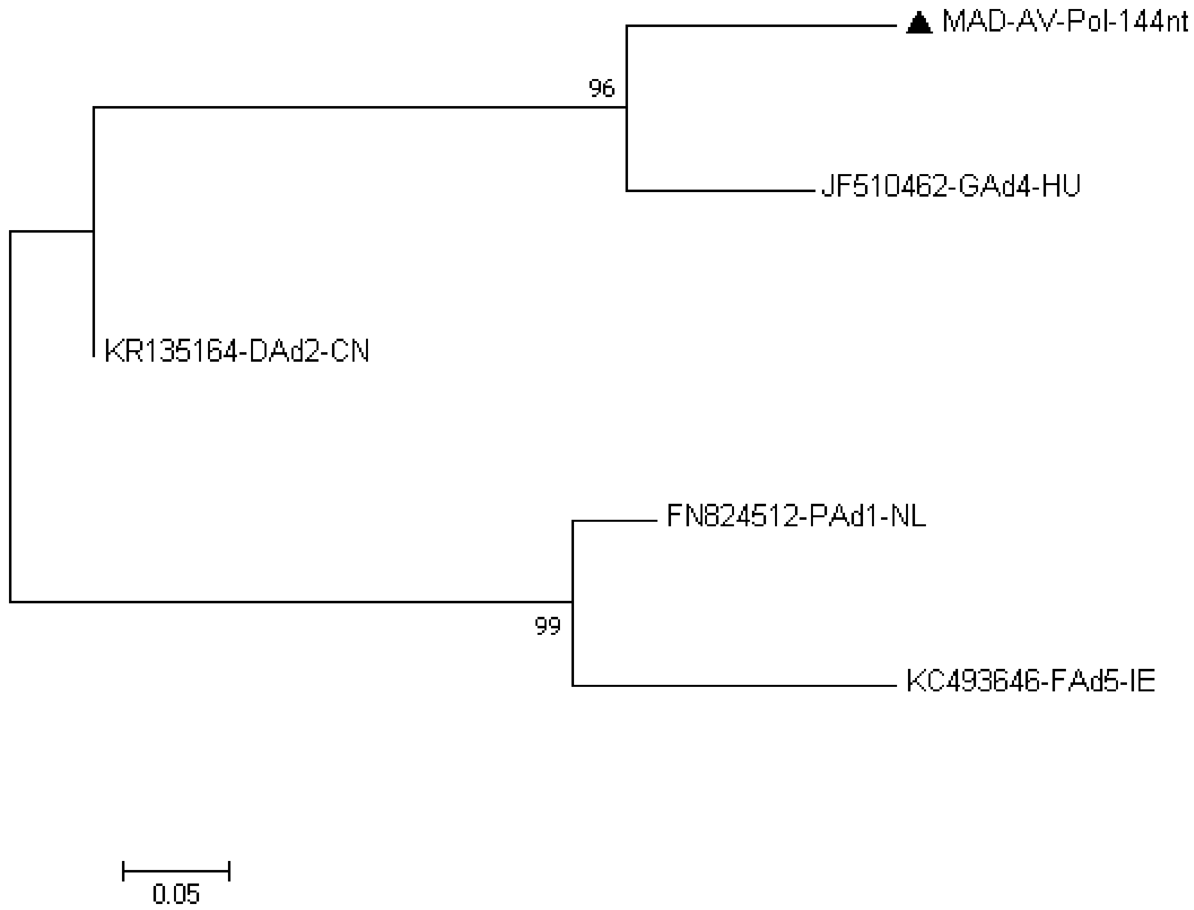


**Figure S4.5: Molecular Phylogenetic analysis by Maximum Likelihood method of AV partial Pol gene**
The evolutionary history was inferred by using the Maximum Likelihood method based on the Jukes-Cantor model[9]. The tree with the highest log likelihood (-566.00) is shown. The percentage of trees in which the associated taxa clustered together is shown next to the branches. Initial tree(s) for the heuristic search were obtained automatically by applying Neighbor-Join and BioNJ algorithms to a matrix of pairwise distances estimated using the Maximum Composite Likelihood (MCL) approach, and then selecting the topology with superior log likelihood value. The rate variation model allowed for some sites to be evolutionarily invariable ([+I], 30.90% sites). The tree is drawn to scale, with branch lengths measured in the number of substitutions per site. The analysis involved 5 nucleotide sequences. All positions containing gaps and missing data were eliminated. There were a total of 144 positions in the final dataset. Evolutionary analyses were conducted in MEGA7[2].

**Table S4.5: Estimates of Evolutionary Divergence between Sequences**
The number of base differences per sequence from between sequences are shown. The analysis involved 5 nucleotide sequences. Codon positions included were 1st+2nd+3rd+Noncoding. All positions containing gaps and missing data were eliminated. There were a total of 144 positions in the final dataset. Evolutionary analyses were conducted in MEGA7.

| **MAD-AV-Pol-144nt** | | | | |
|---|---|---|---|---|
| KR135164-DAd2-CN | 33 | | | |
| JF510462-GAd4-HU | 23 | 31 | | |
| FN824512-PAd1-NL | 45 | 31 | 39 | |
| KC493646-FAd5-IE | 45 | 36 | 38 | 21 |

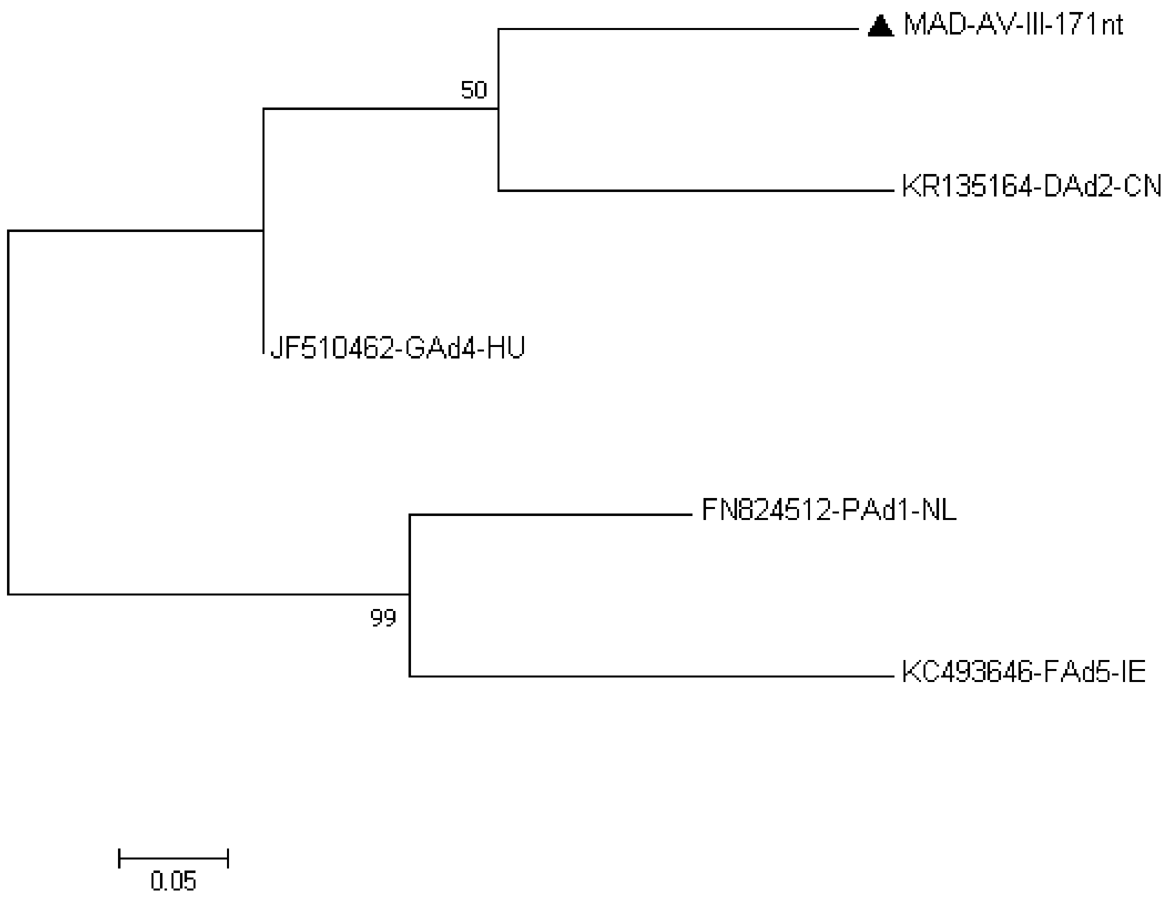


**Figure S4.6: Molecular Phylogenetic analysis by Maximum Likelihood method of AV partial III gene**
The evolutionary history was inferred by using the Maximum Likelihood method based on the Kimura 2-parameter model[1]. The tree with the highest log likelihood (-721.85) is shown. The percentage of trees in which the associated taxa clustered together is shown next to the branches. Initial tree(s) for the heuristic search were obtained automatically by applying Neighbor-Join and BioNJ algorithms to a matrix of pairwise distances estimated using the Maximum Composite Likelihood (MCL) approach, and then selecting the topology with superior log likelihood value. The rate variation model allowed for some sites to be evolutionarily invariable ([+I], 26.61% sites). The tree is drawn to scale, with branch lengths measured in the number of substitutions per site. The analysis involved 5 nucleotide sequences. All positions containing gaps and missing data were eliminated. There were a total of 171 positions in the final dataset. Evolutionary analyses were conducted in MEGA7[2].

**Table S4.6: Estimates of Evolutionary Divergence between Sequences**
The number of base differences per sequence from between sequences are shown. The analysis involved 5 nucleotide sequences. Codon positions included were 1st+2nd+3rd+Noncoding. All positions containing gaps and missing data were eliminated. There were a total of 171 positions in the final dataset. Evolutionary analyses were conducted in MEGA7.

| **MAD-AV-III-171nt** | | | | |
|---|---|---|---|---|
| KR135164-DAd2-CN | 40 | | | |
| JF510462-GAd4-HU | 33 | 37 | | |
| FN824512-PAd1-NL | 61 | 48 | 45 | |
| KC493646-FAd5-IE | 60 | 61 | 48 | 39 |

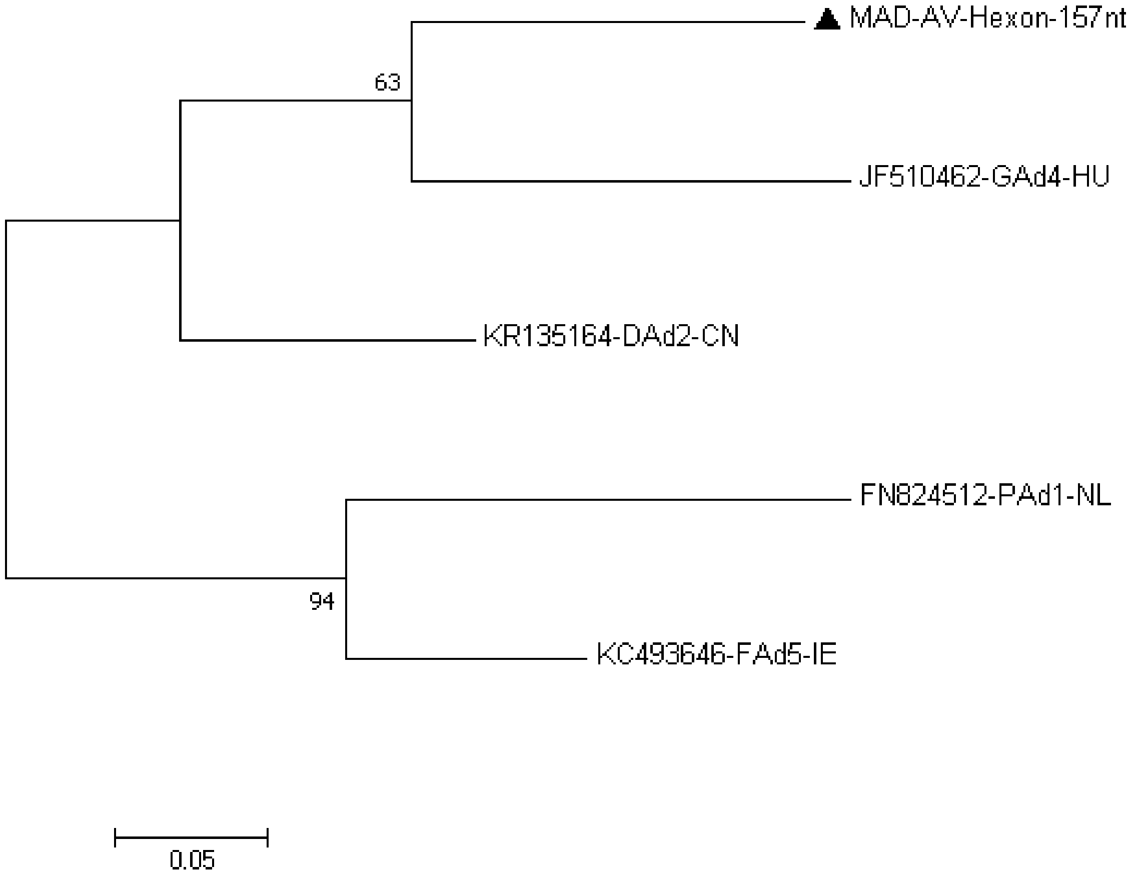


**Figure S4.7: Molecular Phylogenetic analysis by Maximum Likelihood method of AV partial Hexon gene**
The evolutionary history was inferred by using the Maximum Likelihood method based on the Kimura 2-parameter model[1]. The tree with the highest log likelihood (-605.36) is shown. The percentage of trees in which the associated taxa clustered together is shown next to the branches. Initial tree(s) for the heuristic search were obtained automatically by applying Neighbor-Join and BioNJ algorithms to a matrix of pairwise distances estimated using the Maximum Composite Likelihood (MCL) approach, and then selecting the topology with superior log likelihood value. The rate variation model allowed for some sites to be evolutionarily invariable ([+I], 31.21% sites). The tree is drawn to scale, with branch lengths measured in the number of substitutions per site. The analysis involved 5 nucleotide sequences. All positions containing gaps and missing data were eliminated. There were a total of 157 positions in the final dataset. Evolutionary analyses were conducted in MEGA7[2].

**Table S4.7: Estimates of Evolutionary Divergence between Sequences**
The number of base differences per sequence from between sequences are shown. The analysis involved 5 nucleotide sequences. Codon positions included were 1st+2nd+3rd+Noncoding. All positions containing gaps and missing data were eliminated. There were a total of 157 positions in the final dataset. Evolutionary analyses were conducted in MEGA7.

| **MAD-AV-Hexon-157nt** | | | | |
|---|---|---|---|---|
| KR135164-DAd2-CN | 30 | | | |
| JF510462-GAd4-HU | 30 | 33 | | |
| FN824512-PAd1-NL | 42 | 38 | 41 | |
| KC493646-FAd5-IE | 42 | 31 | 33 | 27 |

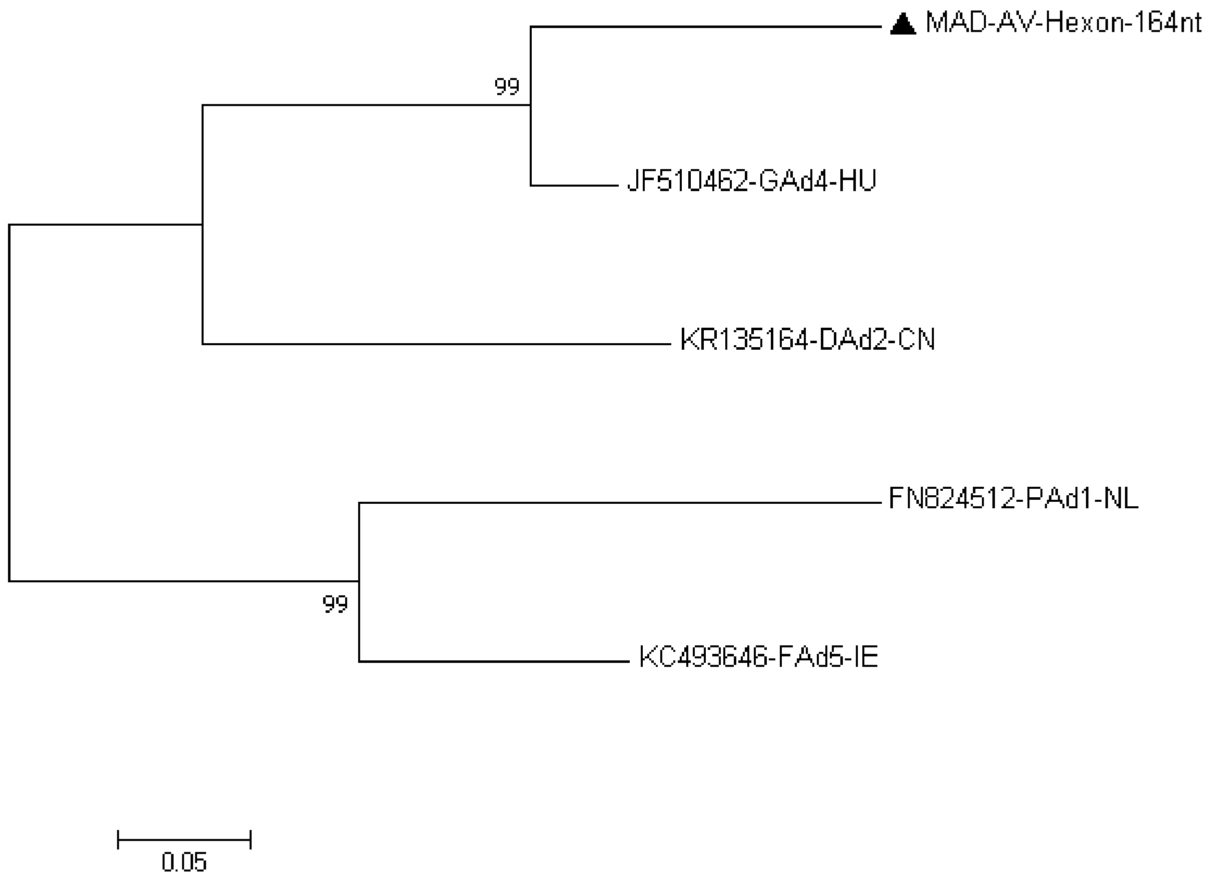


**Figure S4.8: Molecular Phylogenetic analysis by Maximum Likelihood method of AV partial Hexon gene**
The evolutionary history was inferred by using the Maximum Likelihood method based on the Kimura 2-parameter model[1]. The tree with the highest log likelihood (-618.97) is shown. The percentage of trees in which the associated taxa clustered together is shown next to the branches. Initial tree(s) for the heuristic search were obtained automatically by applying Neighbor-Join and BioNJ algorithms to a matrix of pairwise distances estimated using the Maximum Composite Likelihood (MCL) approach, and then selecting the topology with superior log likelihood value. The rate variation model allowed for some sites to be evolutionarily invariable ([+I], 30.79% sites). The tree is drawn to scale, with branch lengths measured in the number of substitutions per site. The analysis involved 5 nucleotide sequences. All positions containing gaps and missing data were eliminated. There were a total of 164 positions in the final dataset. Evolutionary analyses were conducted in MEGA7[2].

**Table S4.8: Estimates of Evolutionary Divergence between Sequences**
The number of base differences per sequence from between sequences are shown. The analysis involved 5 nucleotide sequences. Codon positions included were 1st+2nd+3rd+Noncoding. All positions containing gaps and missing data were eliminated. There were a total of 164 positions in the final dataset. Evolutionary analyses were conducted in MEGA7.

| **MAD-AV-Hexon-164nt** | | | | |
|---|---|---|---|---|
| KR135164-DAd2-CN | 35 | | | |
| JF510462-GAd4-HU | 21 | 31 | | |
| FN824512-PAd1-NL | 49 | 42 | 45 | |
| KC493646-FAd5-IE | 46 | 39 | 39 | 30 |

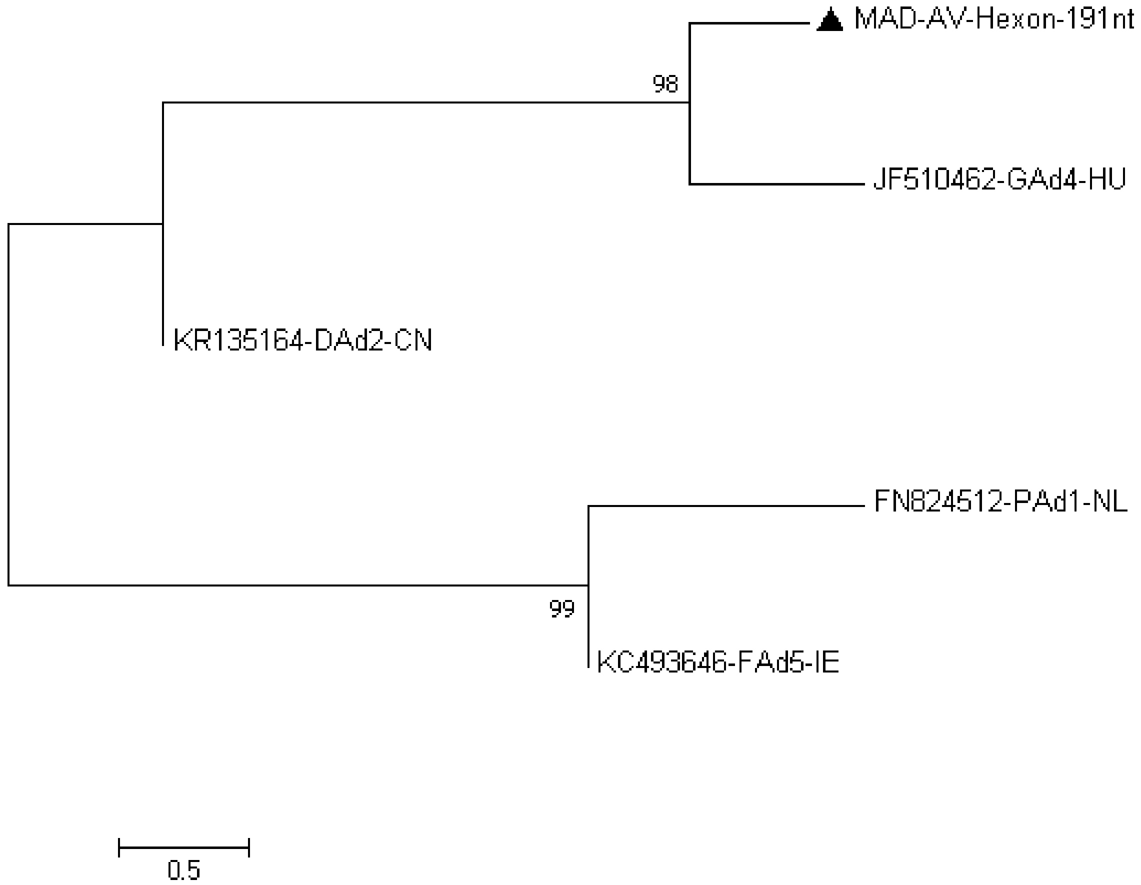


**Figure S4.9: Molecular Phylogenetic analysis by Maximum Likelihood method of AV partial Hexon gene**
The evolutionary history was inferred by using the Maximum Likelihood method based on the Kimura 2-parameter model[1]. The tree with the highest log likelihood (-778.30) is shown. The percentage of trees in which the associated taxa clustered together is shown next to the branches. Initial tree(s) for the heuristic search were obtained automatically by applying Neighbor-Join and BioNJ algorithms to a matrix of pairwise distances estimated using the Maximum Composite Likelihood (MCL) approach, and then selecting the topology with superior log likelihood value. A discrete Gamma distribution was used to model evolutionary rate differences among sites (5 categories (+G, parameter = 0.6781)). The rate variation model allowed for some sites to be evolutionarily invariable ([+I], 29.84% sites). The tree is drawn to scale, with branch lengths measured in the number of substitutions per site. The analysis involved 5 nucleotide sequences. All positions containing gaps and missing data were eliminated. There were a total of 191 positions in the final dataset. Evolutionary analyses were conducted in MEGA7[2].

**Table S4.9: Estimates of Evolutionary Divergence between Sequences**
The number of base differences per sequence from between sequences are shown. The analysis involved 5 nucleotide sequences. Codon positions included were 1st+2nd+3rd+Noncoding. All positions containing gaps and missing data were eliminated. There were a total of 191 positions in the final dataset. Evolutionary analyses were conducted in MEGA7.

| **MAD-AV-Hexon-191nt** | | | | |
|---|---|---|---|---|
| KR135164-DAd2-CN | 49 | | | |
| JF510462-GAd4-HU | 33 | 41 | | |
| FN824512-PAd1-NL | 65 | 52 | 62 | |
| KC493646-FAd5-IE | 59 | 49 | 61 | 33 |

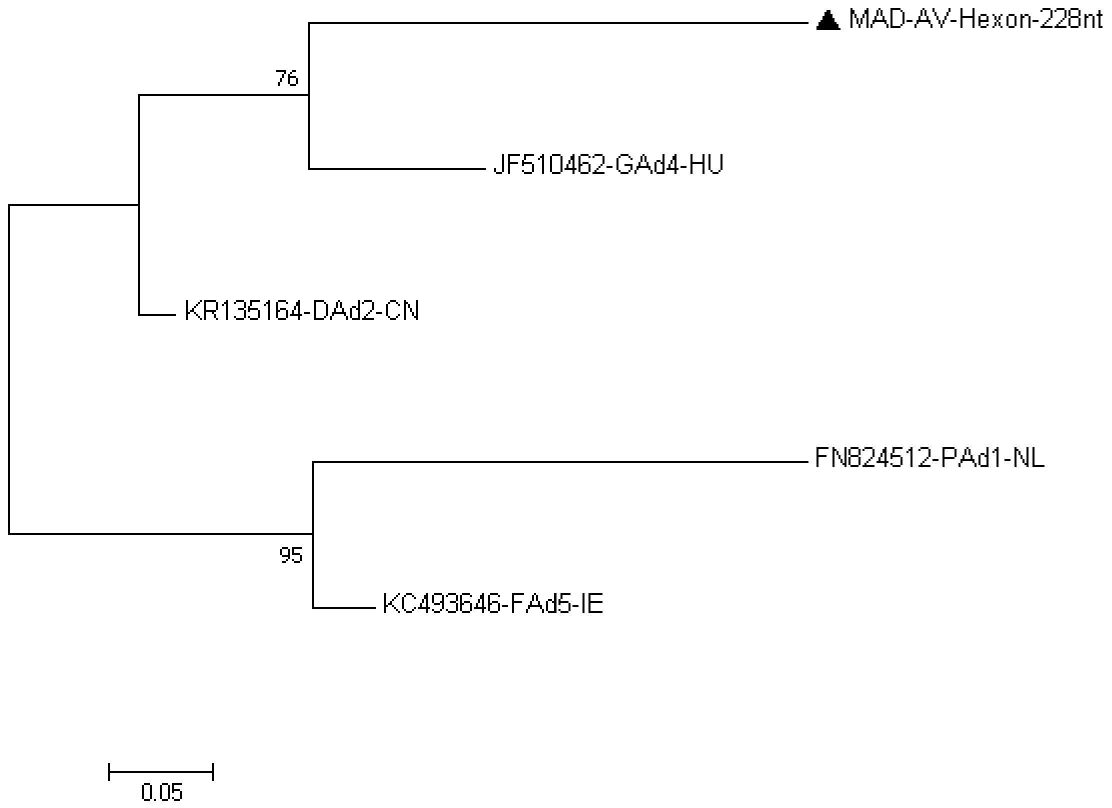


**Figure S4.10: Molecular Phylogenetic analysis by Maximum Likelihood method of AV partial Hexon gene**
The evolutionary history was inferred by using the Maximum Likelihood method based on the Kimura 2-parameter model[1]. The tree with the highest log likelihood (-781.58) is shown. The percentage of trees in which the associated taxa clustered together is shown next to the branches. Initial tree(s) for the heuristic search were obtained automatically by applying Neighbor-Join and BioNJ algorithms to a matrix of pairwise distances estimated using the Maximum Composite Likelihood (MCL) approach, and then selecting the topology with superior log likelihood value. The rate variation model allowed for some sites to be evolutionarily invariable ([+I], 33.99% sites). The tree is drawn to scale, with branch lengths measured in the number of substitutions per site. The analysis involved 5 nucleotide sequences. All positions containing gaps and missing data were eliminated. There were a total of 228 positions in the final dataset. Evolutionary analyses were conducted in MEGA7[2].

**Table S4.10: Estimates of Evolutionary Divergence between Sequences**
The number of base differences per sequence from between sequences are shown. The analysis involved 5 nucleotide sequences. Codon positions included were 1st+2nd+3rd+Noncoding. All positions containing gaps and missing data were eliminated. There were a total of 228 positions in the final dataset. Evolutionary analyses were conducted in MEGA7.

| **MAD-AV-Hexon-228nt** | | | | |
|---|---|---|---|---|
| KR135164-DAd2-CN | 43 | | | |
| JF510462-GAd4-HU | 40 | 28 | | |
| FN824512-PAd1-NL | 61 | 41 | 42 | |
| KC493646-FAd5-IE | 54 | 36 | 41 | 36 |

5. **Adeno-associated virus from MAD faecal sample**

| Figure and Table | Long Name (Format: Sample-virus-protein-length-quality-coverage-year) | Short name (Format: Sample-virus-protein-length) | Coverage | No. of nucleotides | Mapping quality threshold | NCBI accession number |
|---|---|---|---|---|---|---|
| S5.1 | MAD-Adenoassociated-virus-rep-protein-94nt-Q20-C-4-5-2016 | MAD-AAV-RP-94nt | 4-5 | 94 | 20 | MH107865 |
| S5.2 | MAD-Adenoassociated-virus-capsid-protein-279nt-Q32-C-7-27-2016 | MAD-AAV-CP-279nt | 7-27 | 279 | 32 | MH107866 |
| S5.3 | MAD-Adenoassociated-virus-capsid-protein-142nt-Q32-C-3-4-2016 | MAD-AAV-CP-142nt | 3-4 | 142 | 32 | MH107867 |
| NCBI sequences taken for phylogenetic analysis | | | | | | |
| Long Name | | Short Name (Format: NCBI accession number-virus-country/state) | Country of collection | Collection date | | |
| KX583629-Adenoassociated-virus-isolate-MHH-05-2015-complete-genome | | KX583629-AAV-CN | China | 2015 | | |
| KY475562-Goose-parvovirus-strain-RC16-complete-genome | | KY475562-GPV-CN | China | 2016 | | |
| KY679174-Duck-parvovirus-isolate-SC16-complete-genome | | KY679174-DPV-CN | China | 2016 | | |
| KY069274-Muscovy-duck-parvovirus-strain-LH-complete-genome | | KY069274-MDPV-CN | China | 2008 | | |
| KR265069-Goose-parvovirus-strain-WX2-capsid-protein-(VP)-gene-complete-cds | | KR265069-GPV-CN | China | 2014 | | |

The table points towards the molecular phylogenetic analysis by Maximum Likelihood method of AAV. The first half of the table provides details to identify the sample from which the virus was isolated, protein that encodes the consensus sequences that are being analysed, the length of the consensus sequences thus providing the depth of the gene being analysed, minimum mapping quality threshold used in IGV for the generation of the consensus sequences, the coverage of the consensus sequences from IGV, the year of collection of the sample and the NCBI accession number assigned to the particular consensus sequence. Corresponding short names have been used in the phylogenetic trees which contain the sample, virus, protein and the number of nucleotides. The second half of the table provides the details of the sequences that were used for the molecular phylogenetic analysis. They were found to be the most closely related sequences to the consensus

sequences generated using BLASTN. Corresponding short names have been used in the phylogenetic trees which contain the NCBI accession number, virus and the country of collection. The collection date is given as retrieved from the corresponding NCBI nucleotide reference dataset which together with the country of collection can provide an insight into the possible evolution of the virus through the years.

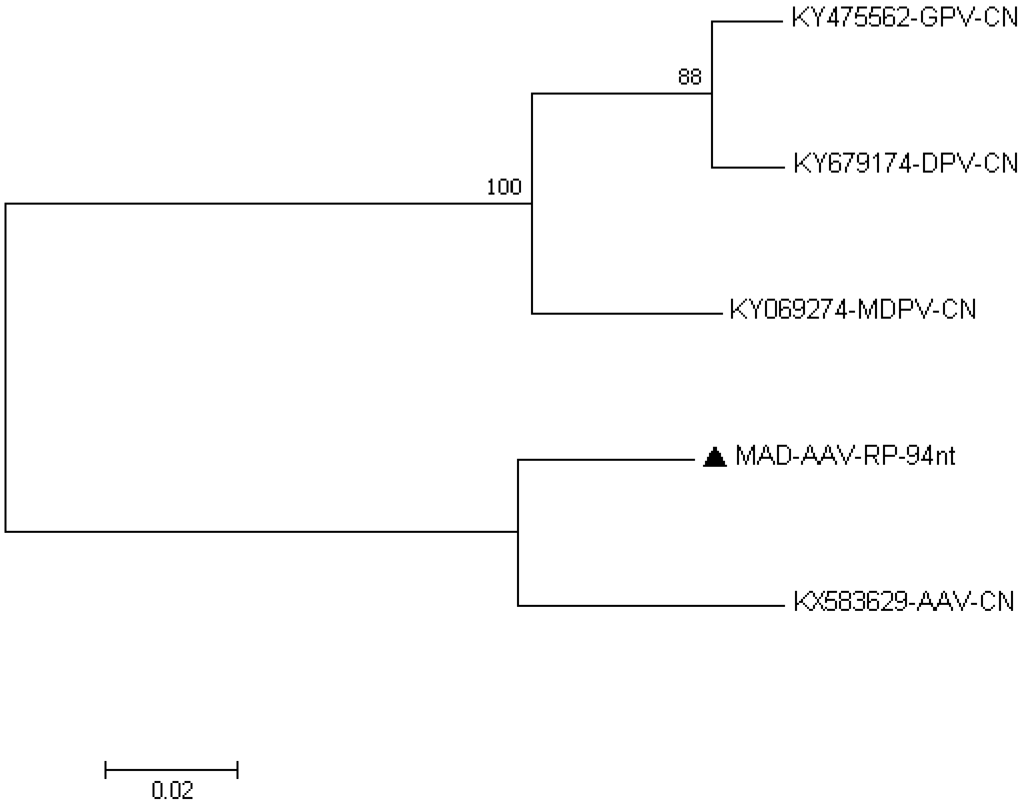


**Figure S5.1: Molecular Phylogenetic analysis by Maximum Likelihood method of AAV partial RP gene**
The evolutionary history was inferred by using the Maximum Likelihood method based on the Jukes-Cantor model[9]. The tree with the highest log likelihood (-255.32) is shown. The percentage of trees in which the associated taxa clustered together is shown next to the branches. Initial tree(s) for the heuristic search were obtained automatically by applying Neighbor-Join and BioNJ algorithms to a matrix of pairwise distances estimated using the Maximum Composite Likelihood (MCL) approach, and then selecting the topology with superior log likelihood value. The tree is drawn to scale, with branch lengths measured in the number of substitutions per site. The analysis involved 5 nucleotide sequences. Codon positions included were 1st+2nd+3rd+Noncoding. All positions containing gaps and missing data were eliminated. There were a total of 94 positions in the final dataset. Evolutionary analyses were conducted in MEGA7[2].

**Table S5.1: Estimates of Evolutionary Divergence between Sequences**
The number of base differences per sequence from between sequences are shown. The analysis involved 5 nucleotide sequences. Codon positions included were 1st+2nd+3rd+Noncoding. All positions containing gaps and missing data were eliminated. There were a total of 94 positions in the final dataset. Evolutionary analyses were conducted in MEGA7.

| | | | | |
|---|---|---|---|---|
| **MAD-AAV-RP-94nt** | | | | |
| KX583629-AAV-CN | 6 | | | |
| KY475562-GPV-CN | 16 | 18 | | |
| KY069274-MDPV-CN | 17 | 18 | 6 | |
| KY679174-DPV-CN | 18 | 18 | 2 | 6 |

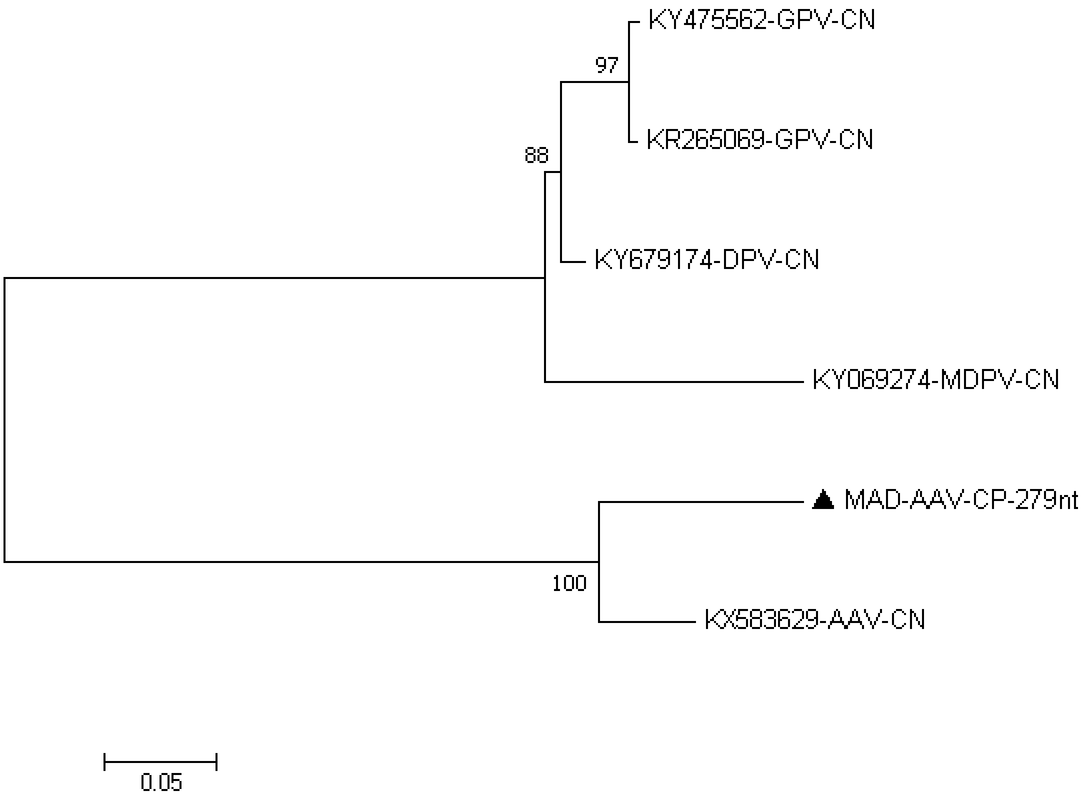


**Figure S5.2: Molecular Phylogenetic analysis by Maximum Likelihood method of AAV partial CP gene**
The evolutionary history was inferred by using the Maximum Likelihood method based on the Kimura 2-parameter model[1]. The tree with the highest log likelihood (-889.81) is shown. The percentage of trees in which the associated taxa clustered together is shown next to the branches. Initial tree(s) for the heuristic search were obtained automatically by applying Neighbor-Join and BioNJ algorithms to a matrix of pairwise distances estimated using the Maximum Composite Likelihood (MCL) approach, and then selecting the topology with superior log likelihood value. A discrete Gamma distribution was used to model evolutionary rate differences among sites (5 categories (+G, parameter = 0.5762)). The tree is drawn to scale, with branch lengths measured in the number of substitutions per site. The analysis involved 6 nucleotide sequences. Codon positions included were 1st+2nd+3rd+Noncoding. All positions containing gaps and missing data were eliminated. There were a total of 255 positions in the final dataset. Evolutionary analyses were conducted in MEGA7 [2].

**Table S5.2: Estimates of Evolutionary Divergence between Sequences**
The number of base differences per sequence from between sequences are shown. The analysis involved 6 nucleotide sequences. Codon positions included were 1st+2nd+3rd+Noncoding. All positions containing gaps and missing data were eliminated. There were a total of 255 positions in the final dataset. Evolutionary analyses were conducted in MEGA7.

| **MAD-AAV-CP-279nt** | | | | | |
|---|---|---|---|---|---|
| KX583629-AAV-CN | 29 | | | | |
| KY475562-GPV-CN | 87 | 82 | | | |
| KY679174-DPV-CN | 85 | 81 | 11 | | |
| KY069274-MDPV-CN | 89 | 88 | 34 | 29 | |
| KR265069-GPV-CN | 85 | 81 | 2 | 11 | 32 |

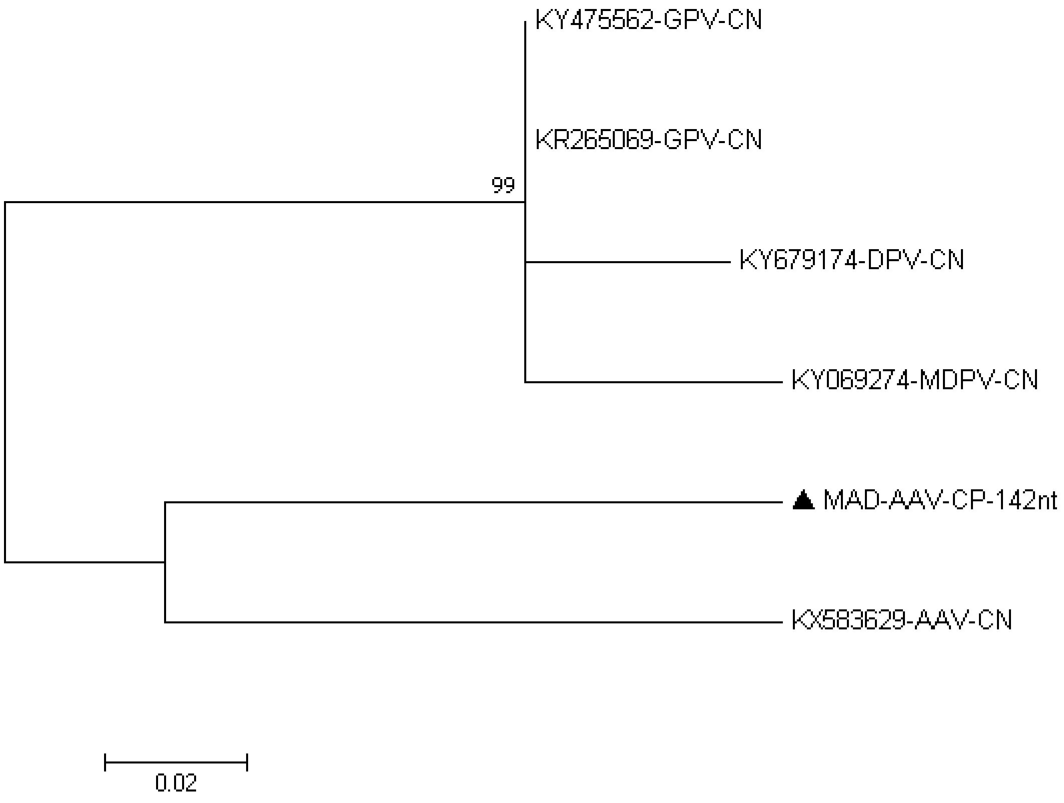


**Figure S5.3: Molecular Phylogenetic analysis by Maximum Likelihood method of AAV partial CP gene**
The evolutionary history was inferred by using the Maximum Likelihood method based on the Jukes-Cantor model[9]. The tree with the highest log likelihood (-403.33) is shown. The percentage of trees in which the associated taxa clustered together is shown next to the branches. Initial tree(s) for the heuristic search were obtained automatically by applying Neighbor-Join and BioNJ algorithms to a matrix of pairwise distances estimated using the Maximum Composite Likelihood (MCL) approach, and then selecting the topology with superior log likelihood value. The tree is drawn to scale, with branch lengths measured in the number of substitutions per site. The analysis involved 6 nucleotide sequences. Codon positions included were 1st+2nd+3rd+Noncoding. All positions containing gaps and missing data were eliminated. There were a total of 142 positions in the final dataset. Evolutionary analyses were conducted in MEGA7[2].

**Table S5.3: Estimates of Evolutionary Divergence between Sequences**
The number of base differences per sequence from between sequences are shown. The analysis involved 6 nucleotide sequences. Codon positions included were 1st+2nd+3rd+Noncoding. All positions containing gaps and missing data were eliminated. There were a total of 142 positions in the final dataset. Evolutionary analyses were conducted in MEGA7.

| **MAD-AAV-CP-142nt** | | | | | |
|---|---|---|---|---|---|
| KX583629-AAV-CN | 22 | | | | |
| KY475562-GPV-CN | 23 | 23 | | | |
| KY679174-DPV-CN | 27 | 27 | 4 | | |
| KY069274-MDPV-CN | 23 | 25 | 5 | 7 | |
| KR265069-GPV-CN | 23 | 23 | 0 | 4 | 5 |

## 6. Rotavirus G from MAD faecal sample

| Figure and Table | Long Name (Format: Sample-virus-segment-protein-length-quality-coverage-year) | Short name (Format: Sample-virus-segment-protein-length) | Coverage | No. of nucleotides | Mapping quality threshold | NCBI accession number |
|---|---|---|---|---|---|---|
| S6.1 | MAD-RotavirusG-seg1-VP1-230nt-Q10-C-3-14-2016 | MAD-RG-s1-VP1-230nt | 3-14 | 230 | 10 | MH085089 |
| S6.2 | MAD-RotavirusG-seg3VP3-162nt-Q10-C-3-7-2016 | MAD-RG-s3-VP3-162nt | 3-7 | 162 | 10 | MH085090 |
| S6.3 | MAD-RotavirusG-seg8-NSP2-149nt-Q10-C-3-6-2016 | MAD-RG-s8-NSP2-149nt | 3-6 | 149 | 10 | MH085891 |
| NCBI sequences taken for phylogenetic analysis | | | | | | |
| Long Name | | Short Name (Format: NCBI accession number-virus-segment-country/state) | Country of collection | Collection date | | |
| Segment 1 | | | | | | |
| KC876010-RotavirusG-pigeon/HK18-strain-HK18-VP1-gene-complete-cds | | KC876010-PRG-s1-CN | China | 2011 | | |
| JN596592-RotavirusG-chicken/03V0567/DEU/2003-segment-1-complete-sequence | | JN596592-CRG-s1-DE | Germany | 2003 | | |
| KX362367-RotavirusB-strain-RVB/Pig-wt/VNM/12089-7/VP1-RNA-dependent-RNA-polymerase-(VP1)-gene-partial-cds | | KX362367-PiRB-VN | Vietnam | 2012 | | |
| Segment 3/4 (as per matched NCBI submitted reference sequence) | | | | | | |
| JQ920005-RotavirusG-chicken/03V0567/DEU/2003-segment4-complete-sequence | | JQ920005-CRG-s4-DE | Germany | 2003 | | |
| KJ752086-RotavirusG-strain-RVG/chicken/ZAF/MRC-DPRU1679/2011/GXPX-segment3-RNA-capping-protein-VP3-(VP3)-gene-complete-cds | | KJ752086-CRG-s3-ZA | South Africa | 2011 | | |
| KC876012-RotavirusG-pigeon/HK18-strain-HK18-VP3-gene-complete-cds | | KC876012-PRG-s4-CN | China | 2011 | | |
| Segment 8 | | | | | | |

| | | | |
|---|---|---|---|
| KC876006-RotavirusG-pigeon/HK18-strain-HK18-NSP2-gene-complete-cds | KC876006-PRG-s8-CN | China | 2011 |
| JQ920009-RotavirusG-chicken/03V0567/DEU/2003-segment8-complete-sequence | JQ920009-CRG-s8-DE | Germany | 2003 |
| KJ752080-RotavirusG-strain-RVG/chicken/ZAF/MRC-DPRU1679/2011/GXPX-segment8-non-structural-protein-2-(NSP2)-gene-complete-cds | KJ752080-CRG-s8-ZA | South Africa | 2011 |

The table points towards the molecular phylogenetic analysis by Maximum Likelihood method of RG. The first half of the table provides details to identify the sample from which the virus was isolated, protein that encodes the consensus sequences that are being analysed, the length of the consensus sequences thus providing the depth of the gene being analysed, minimum mapping quality threshold used in IGV for the generation of the consensus sequences, the coverage of the consensus sequences from IGV, the year of collection of the sample and the NCBI accession number assigned to the particular consensus sequence. Corresponding short names have been used in the phylogenetic trees which contain the sample, virus, protein and the number of nucleotides. The second half of the table provides the details of the sequences that were used for the molecular phylogenetic analysis. They were found to be the most closely related sequences to the consensus sequences generated using BLASTN. Corresponding short names have been used in the phylogenetic trees which contain the NCBI accession number, virus and the country of collection. The collection date is given as retrieved from the corresponding NCBI nucleotide reference dataset which together with the country of collection can provide an insight into the possible evolution of the virus through the years.

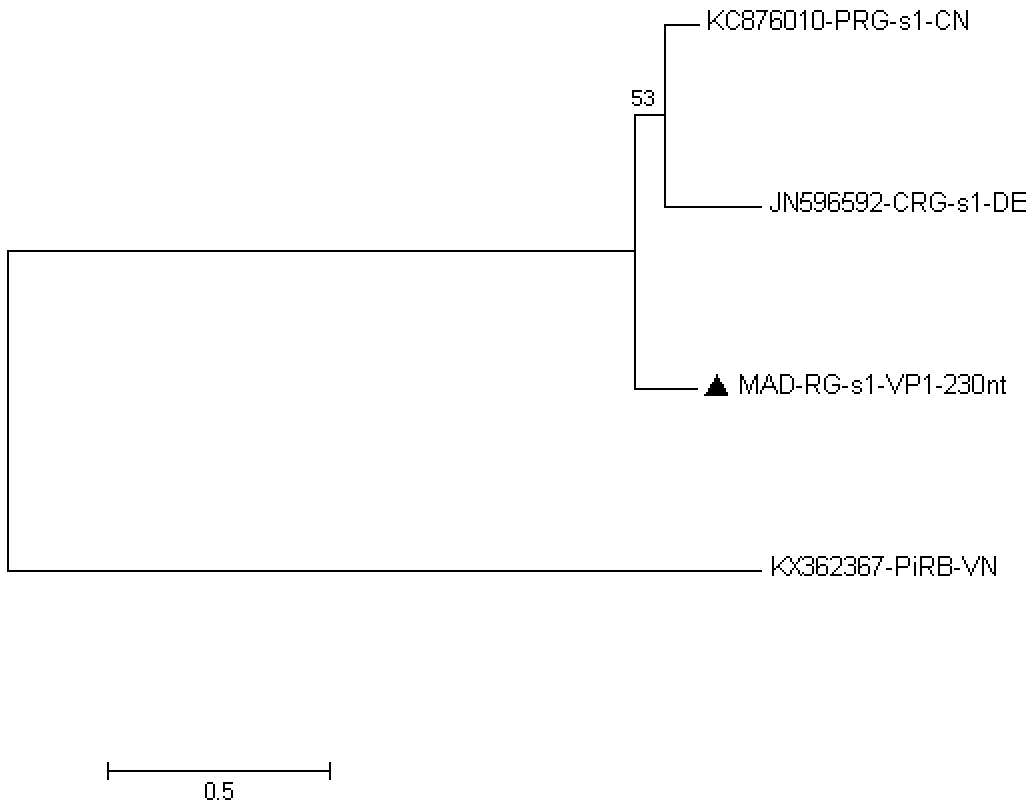


**Figure S6.1: Molecular Phylogenetic analysis by Maximum Likelihood method of RG partial VP1 gene**
The evolutionary history was inferred by using the Maximum Likelihood method based on the Tamura 3-parameter model[4]. The tree with the highest log likelihood (-805.99) is shown. The percentage of trees in which the associated taxa clustered together is shown next to the branches. Initial tree(s) for the heuristic search were obtained automatically by applying Neighbor-Join and BioNJ algorithms to a matrix of pairwise distances estimated using the Maximum Composite Likelihood (MCL) approach, and then selecting the topology with superior log likelihood value. The rate variation model allowed for some sites to be evolutionarily invariable ([+I], 36.76% sites). The tree is drawn to scale, with branch lengths measured in the number of substitutions per site. The analysis involved 4 nucleotide sequences. Codon positions included were 1st+2nd+3rd+Noncoding. All positions containing gaps and missing data were eliminated. There were a total of 227 positions in the final dataset. Evolutionary analyses were conducted in MEGA7[2].

**Table S6.1: Estimates of Evolutionary Divergence between Sequences**
The number of base differences per sequence from between sequences are shown. The analysis involved 4 nucleotide sequences. Codon positions included were 1st+2nd+3rd+Noncoding. All positions containing gaps and missing data were eliminated. There were a total of 227 positions in the final dataset. Evolutionary analyses were conducted in MEGA7.

| **MAD-RG-s1-VP1-230nt** | | | |
|---|---|---|---|
| KC876010-PRG-s1-CN | 44 | | |
| JN596592-CRG-s1-DE | 57 | 38 | |
| KX362367-PiRB-VN | 92 | 88 | 87 |

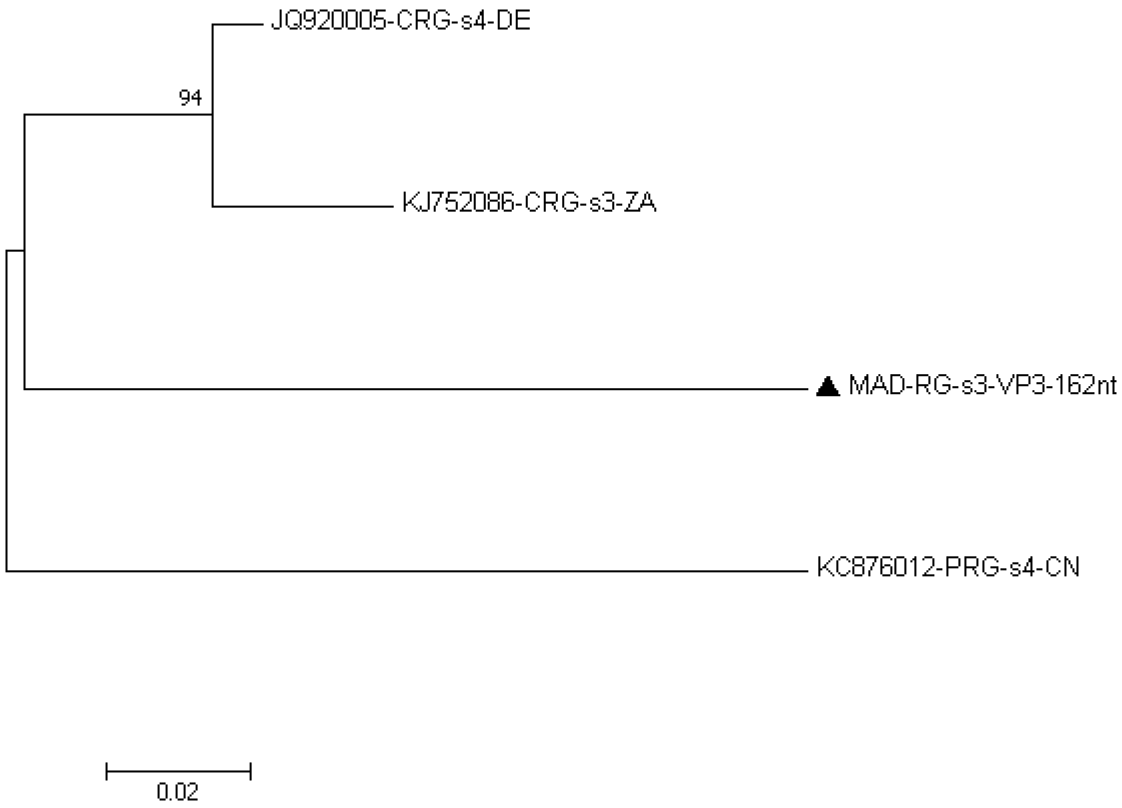


**Figure S6.2: Molecular Phylogenetic analysis by Maximum Likelihood method of RG partial VP3 gene**
The evolutionary history was inferred by using the Maximum Likelihood method based on the Tamura 3-parameter model[4]. The tree with the highest log likelihood (-388.80) is shown. The percentage of trees in which the associated taxa clustered together is shown next to the branches. Initial tree(s) for the heuristic search were obtained automatically by applying Neighbor-Join and BioNJ algorithms to a matrix of pairwise distances estimated using the Maximum Composite Likelihood (MCL) approach, and then selecting the topology with superior log likelihood value. The tree is drawn to scale, with branch lengths measured in the number of substitutions per site. The analysis involved 4 nucleotide sequences. Codon positions included were 1st+2nd+3rd+Noncoding. All positions containing gaps and missing data were eliminated. There were a total of 162 positions in the final dataset. Evolutionary analyses were conducted in MEGA7[2].

**Table S6.2: Estimates of Evolutionary Divergence between Sequences**
The number of base differences per sequence from between sequences are shown. The analysis involved 4 nucleotide sequences. Codon positions included were 1st+2nd+3rd+Noncoding. All positions containing gaps and missing data were eliminated. There were a total of 162 positions in the final dataset. Evolutionary analyses were conducted in MEGA7.

| **MAD-RG-s3-VP3-162nt** | | | |
|---|---|---|---|
| JQ920005-CRG-s4-DE | 20 | | |
| KJ752086-CRG-s3-ZA | 23 | 5 | |
| KC876012-PRG-s4-CN | 29 | 21 | 22 |

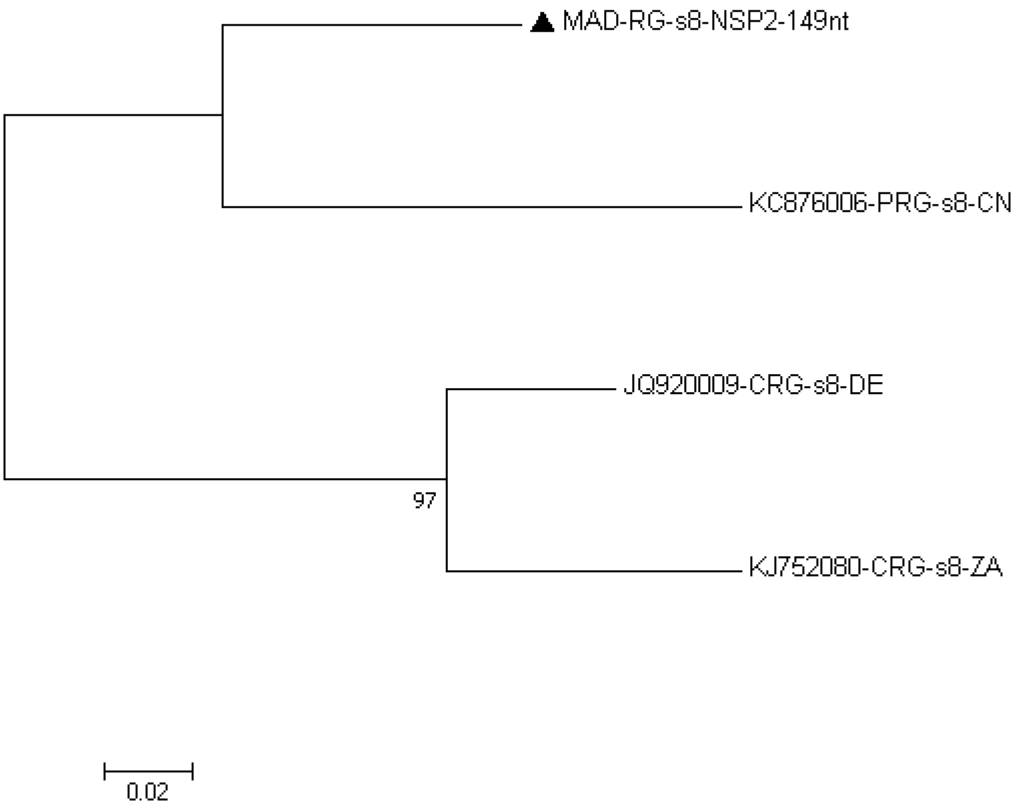


**Figure S6.3: Molecular Phylogenetic analysis by Maximum Likelihood method of RG partial NSP2 gene**
The evolutionary history was inferred by using the Maximum Likelihood method based on the Tamura 3-parameter model[4]. The tree with the highest log likelihood (-383.45) is shown. The percentage of trees in which the associated taxa clustered together is shown next to the branches. Initial tree(s) for the heuristic search were obtained automatically by applying Neighbor-Join and BioNJ algorithms to a matrix of pairwise distances estimated using the Maximum Composite Likelihood (MCL) approach, and then selecting the topology with superior log likelihood value. A discrete Gamma distribution was used to model evolutionary rate differences among sites (5 categories (+G, parameter = 0.4597)). The tree is drawn to scale, with branch lengths measured in the number of substitutions per site. The analysis involved 4 nucleotide sequences. Codon positions included were 1st+2nd+3rd+Noncoding. All positions containing gaps and missing data were eliminated. There were a total of 149 positions in the final dataset. Evolutionary analyses were conducted in MEGA7[2].

**Table S6.3: Estimates of Evolutionary Divergence between Sequences**
The number of base differences per sequence from between sequences are shown. The analysis involved 4 nucleotide sequences. Codon positions included were 1st+2nd+3rd+Noncoding. All positions containing gaps and missing data were eliminated. There were a total of 149 positions in the final dataset. Evolutionary analyses were conducted in MEGA7.

| **MAD-RG-s8-NSP2-149nt** | | | |
|---|---|---|---|
| KC876006-PRG-s8-CN | 19 | | |
| JQ920009-CRG-s8-DE | 22 | 26 | |
| KJ752080-CRG-s8-ZA | 26 | 23 | 13 |

## 7. Hubei chryso-like virus 1 from MUD faecal sample

| Figure and Table | Long Name (Format: Sample-virus-segment-protein-length-quality-coverage-year) | Short name (Format: Sample-virus-segment-protein-length) | Coverage | No. of nucleotides | Mapping quality threshold | NCBI accession number |
|---|---|---|---|---|---|---|
| S7.1 | MUD-Hubei-chryso-like-virus-1-seg2-putative-protease-2175nt-Q32-C-4-71-2016 | MUD-HCLV1-s2-PP-2175nt | 4-71 | 2175 | 32 | MH085093 |
| S7.2 | MUD-Hubei-chryso-like-virus-1-seg3-hypothetical-protein-536nt-Q32-C-9-42-2016 | MUD-HCLV1-s3-HP-536nt | 9-42 | 536 | 32 | MH085094 |
| S7.3 | MUD-Hubei-chryso-like-virus-1-seg4-hypothetical-protein-1938nt-Q32-C-3-121-2016 | MUD-HCLV1-s4-HP-1938nt | 3-121 | 1938 | 32 | MH085095 |
| NCBI sequences taken for phylogenetic analysis | | | | | | |
| Long Name | | Short Name (Format: NCBI accession number-virus-segment-country/state) | Country or State of collection | Collection date | | |
| Segment 2 | | | | | | |
| MF176389-Hubei-chryso-like-virus-1-strain-mosWSX51080-segment2-complete-sequence | | MF176389-HCLV1-s2-WA | Western Australia | 2015 | | |
| MF176262-Hubei-chryso-like-virus-1-strain-mos172gb42656-segment2-complete-sequence | | MF176262-HCLV1-s2-WA | Western Australia | 2015 | | |
| MF176310-Hubei-chryso-like-virus-1-strain-mos191gb77171-segment2-complete-sequence | | MF176310-HCLV1-s2-WA | Western Australia | 2015 | | |
| MF176281-Hubei-chryso-like-virus-1-strain-mos172X13576-segment2-complete-sequence | | MF176281-HCLV1-s2-WA | Western Australia | 2015 | | |
| MF176369-Hubei-chryso-like-virus-1-strain-mosWSgb49785-segment2-complete-sequence | | MF176369-HCLV1-s2-WA | Western Australia | 2015 | | |
| Segment 3 | | | | | | |

| | | | |
|---|---|---|---|
| MF176263-Hubei-chryso-like-virus-1-strain-mos172gb42656-segment3-complete-sequence | MF176263-HCLV1-s3-WA | Western Australia | 2015 |
| MF176370-Hubei-chryso-like-virus-1-strain-mosWSgb49785-segment3-complete-sequence | MF176370-HCLV1-s3-WA | Western Australia | 2015 |
| MF176390-Hubei-chryso-like-virus-1-strain-mosWSX51080-segment3-complete-sequence | MF176390-HCLV1-s3-WA | Western Australia | 2015 |
| MF176282-Hubei-chryso-like-virus-1-strain-mos172X13576-segment3-complete-sequence | MF176282-HCLV1-s3-WA | Western Australia | 2015 |
| Segment 4 | | | |
| MF176391-Hubei-chryso-like-virus-1-strain-mosWSX51080-segment4-complete-sequence | MF176391-HCLV1-s4-WA | Western Australia | 2015 |
| MF176371-Hubei-chryso-like-virus-1-strain-mosWSgb49785-segment4-complete-sequence | MF176371-HCLV1-s4-WA | Western Australia | 2015 |
| MF176312-Hubei-chryso-like-virus-1-strain-mos191gb77171-segment4-complete-sequence | MF176312-HCLV1-s4-WA | Western Australia | 2015 |
| MF176283-Hubei-chryso-like-virus-1-strain-mos172X13576-segment4-complete-sequence | MF176283-HCLV1-s4-WA | Western Australia | 2015 |
| MF176264-Hubei-chryso-like-virus-1-strain-mos172gb42656-segment-4-complete-sequence | MF176264-HCLV1-s4-WA | Western Australia | 2015 |

The table points towards the molecular phylogenetic analysis by Maximum Likelihood method of HCLV1. The first half of the table provides details to identify the sample from which the virus was isolated, protein that encodes the consensus sequences that are being analysed, the length of the consensus sequences thus providing the depth of the gene being analysed, minimum mapping quality threshold used in IGV for the generation of the consensus sequences, the coverage of the consensus sequences from IGV, the year of collection of the sample and the NCBI accession number assigned to the particular consensus sequence. Corresponding short names have been used in the phylogenetic trees which contain the sample, virus, protein and the number of nucleotides. The second half of the table provides the details of the sequences that were used for the molecular phylogenetic analysis. They were found to be the most closely related sequences to the consensus sequences generated using BLASTN. Corresponding short names have been used in the phylogenetic trees which contain the NCBI accession number, virus and the country of collection. The collection date is given as retrieved from the corresponding NCBI nucleotide reference dataset which together with the country of collection can provide an insight into the possible evolution of the virus through the years.

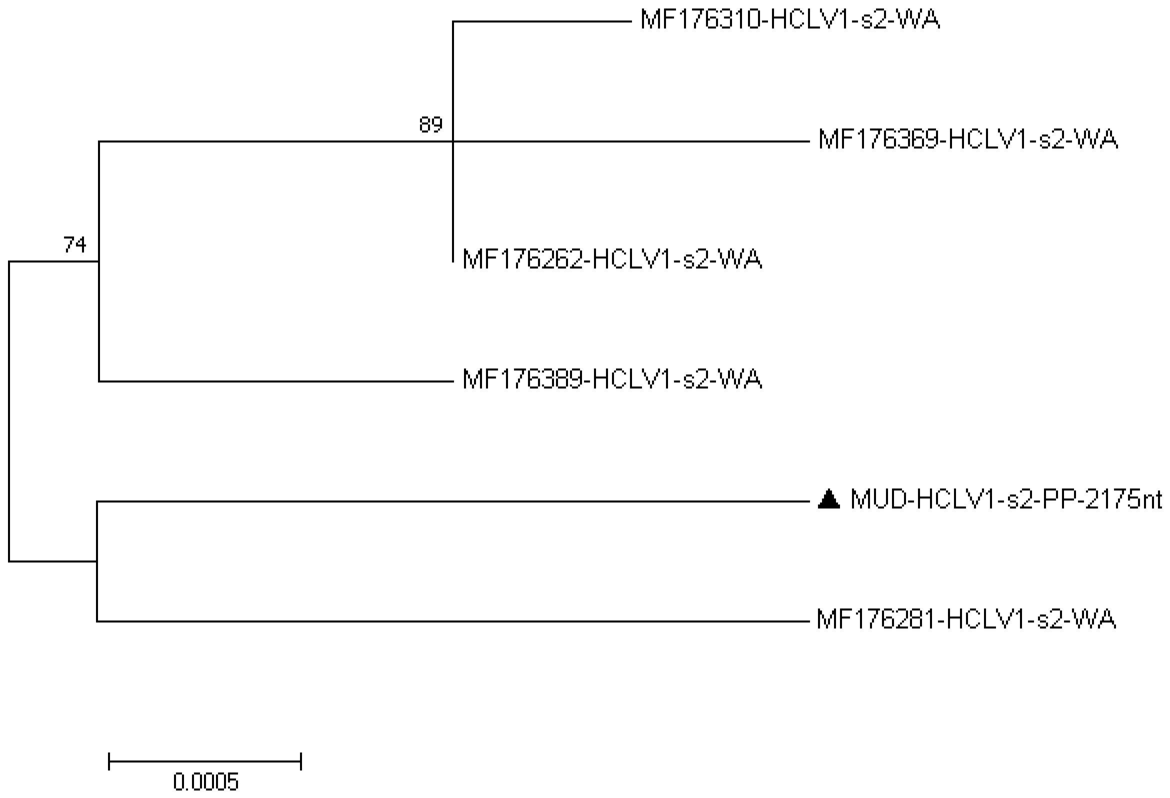


**Figure S7.1: Molecular Phylogenetic analysis by Maximum Likelihood method of HCLV1 partial PP gene**
The evolutionary history was inferred by using the Maximum Likelihood method based on the Hasegawa-Kishino-Yano model[5]. The tree with the highest log likelihood (-3129.64) is shown. The percentage of trees in which the associated taxa clustered together is shown next to the branches. Initial tree(s) for the heuristic search were obtained automatically by applying Neighbor-Join and BioNJ algorithms to a matrix of pairwise distances estimated using the Maximum Composite Likelihood (MCL) approach, and then selecting the topology with superior log likelihood value. The tree is drawn to scale, with branch lengths measured in the number of substitutions per site. The analysis involved 6 nucleotide sequences. Codon positions included were 1st+2nd+3rd+Noncoding. All positions containing gaps and missing data were eliminated. There were a total of 2175 positions in the final dataset. Evolutionary analyses were conducted in MEGA7[2].

**Table S7.1: Estimates of Evolutionary Divergence between Sequences**
The number of base differences per sequence from between sequences are shown. The analysis involved 6 nucleotide sequences. Codon positions included were 1st+2nd+3rd+Noncoding. All positions containing gaps and missing data were eliminated. There were a total of 2175 positions in the final dataset. Evolutionary analyses were conducted in MEGA7.

| **MUD-HCLV1-s2-PP-2175nt** | | | | | |
|---|---|---|---|---|---|
| MF176389-HCLV1-s2-WA | 7 | | | | |
| MF176262-HCLV1-s2-WA | 7 | 4 | | | |
| MF176310-HCLV1-s2-WA | 8 | 5 | 1 | | |
| MF176281-HCLV1-s2-WA | 8 | 7 | 7 | 8 | |
| MF176369-HCLV1-s2-WA | 9 | 6 | 2 | 3 | 9 |

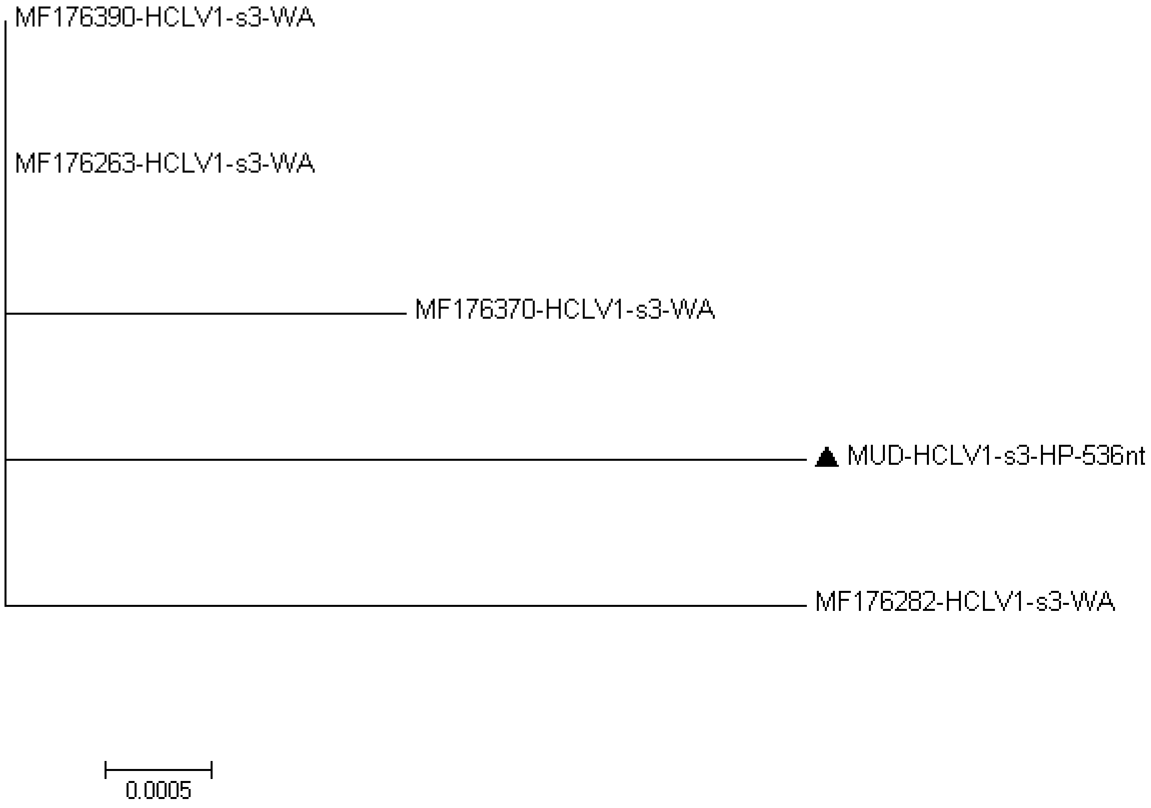


**Figure S7.2: Molecular Phylogenetic analysis by Maximum Likelihood method of HCLV1 partial HP gene**
The evolutionary history was inferred by using the Maximum Likelihood method based on the Jukes-Cantor model[9]. The tree with the highest log likelihood (-782.19) is shown. The percentage of trees in which the associated taxa clustered together is shown next to the branches. Initial tree(s) for the heuristic search were obtained automatically by applying Neighbor-Join and BioNJ algorithms to a matrix of pairwise distances estimated using the Maximum Composite Likelihood (MCL) approach, and then selecting the topology with superior log likelihood value. The tree is drawn to scale, with branch lengths measured in the number of substitutions per site. The analysis involved 5 nucleotide sequences. Codon positions included were 1st+2nd+3rd+Noncoding. All positions containing gaps and missing data were eliminated. There were a total of 536 positions in the final dataset. Evolutionary analyses were conducted in MEGA7[2].

**Table S7.2: Estimates of Evolutionary Divergence between Sequences**
The number of base differences per sequence from between sequences are shown. The analysis involved 5 nucleotide sequences. Codon positions included were 1st+2nd+3rd+Noncoding. All positions containing gaps and missing data were eliminated. There were a total of 536 positions in the final dataset. Evolutionary analyses were conducted in MEGA7.

| **MUD-HCLV1-s3-HP-536nt** | | | | |
|---|---|---|---|---|
| MF176390-HCLV1-s3-WA | 2 | | | |
| MF176263-HCLV1-s3-WA | 2 | 0 | | |
| MF176370-HCLV1-s3-WA | 3 | 1 | 1 | |
| MF176282-HCLV1-s3-WA | 4 | 2 | 2 | 3 |

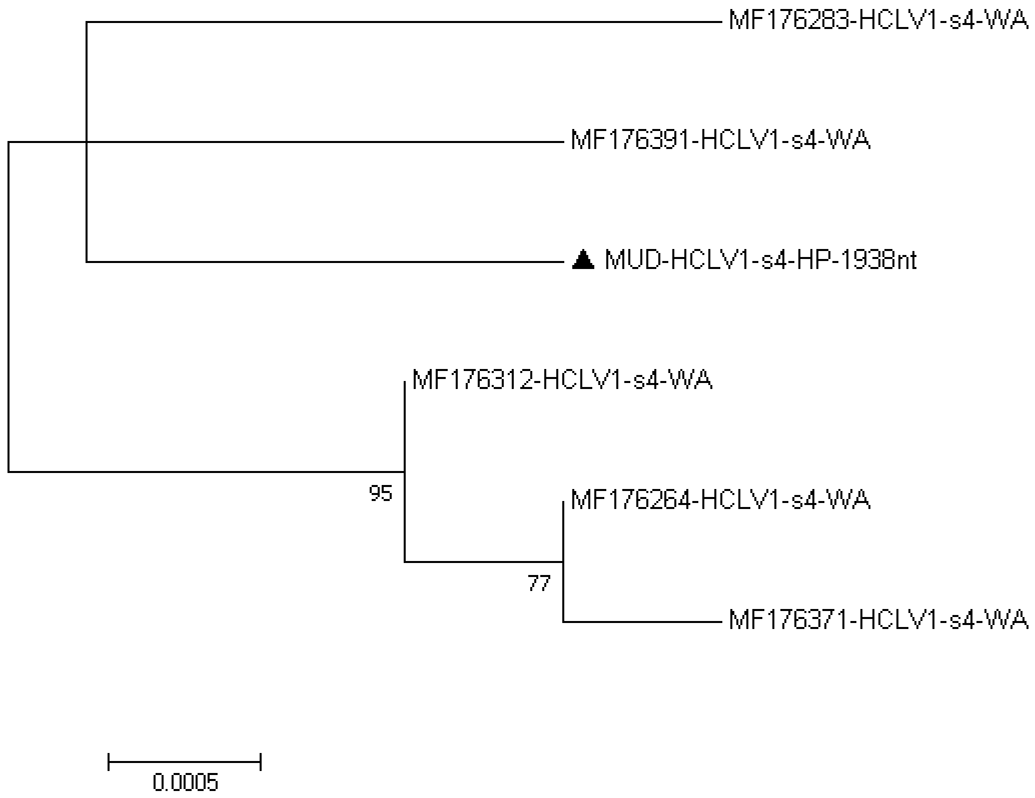


**Figure S7.3: Molecular Phylogenetic analysis by Maximum Likelihood method of HCLV1 partial HP gene**
The evolutionary history was inferred by using the Maximum Likelihood method based on the Hasegawa-Kishino-Yano model[5]. The tree with the highest log likelihood (-2775.63) is shown. The percentage of trees in which the associated taxa clustered together is shown next to the branches. Initial tree(s) for the heuristic search were obtained automatically by applying Neighbor-Join and BioNJ algorithms to a matrix of pairwise distances estimated using the Maximum Composite Likelihood (MCL) approach, and then selecting the topology with superior log likelihood value. The tree is drawn to scale, with branch lengths measured in the number of substitutions per site. The analysis involved 6 nucleotide sequences. Codon positions included were 1st+2nd+3rd+Noncoding. All positions containing gaps and missing data were eliminated. There were a total of 1938 positions in the final dataset. Evolutionary analyses were conducted in MEGA7[2].

**Table S7.3: Estimates of Evolutionary Divergence between Sequences**
The number of base differences per sequence from between sequences are shown. The analysis involved 6 nucleotide sequences. Codon positions included were 1st+2nd+3rd+Noncoding. All positions containing gaps and missing data were eliminated. There were a total of 1938 positions in the final dataset. Evolutionary analyses were conducted in MEGA7.

| **MUD-HCLV1-s4-HP-1938nt** | | | | | |
|---|---|---|---|---|---|
| MF176283-HCLV1-s4-WA | 7 | | | | |
| MF176391-HCLV1-s4-WA | 6 | 7 | | | |
| MF176312-HCLV1-s4-WA | 6 | 7 | 6 | | |
| MF176264-HCLV1-s4-WA | 7 | 8 | 7 | 1 | |
| MF176371-HCLV1-s4-WA | 8 | 9 | 8 | 2 | 1 |

## 8. Culex Negev like virus 3 of MUD faecal sample

<table>
<tr><th>Figure and Table</th><th>Long Name<br>(Format: Sample-virus-protein-length-quality-coverage-year)</th><th>Short name<br>(Format: Sample-virus-protein-length)</th><th>Coverage</th><th>No. of nucleotides</th><th>Mapping quality threshold</th><th>NCBI accession number</th></tr>
<tr><td>8.1</td><td>MUD-Culex-negev-like-virus3-Rp-531nt-Q32-C-3-22-2016</td><td>MUD-CNLV3-Rp-531nt</td><td>3-22</td><td>531</td><td>32</td><td>MH085096</td></tr>
<tr><td>8.2</td><td>MUD-Culex-negev-like-virus3-Rp-275nt-Q32-C-5-30-2016</td><td>MUD-CNLV3-Rp-275nt</td><td>5-30</td><td>275</td><td>32</td><td>MH085097</td></tr>
<tr><td>8.3</td><td>MUD-Culex-negev-like-virus3-putative-membrane-protein-456nt-Q32-C-3-19-2016</td><td>MUD-CNLV3-PMP-456nt</td><td>3-19</td><td>456</td><td>32</td><td>MH085098</td></tr>
<tr><td colspan="7">NCBI sequences taken for phylogenetic analysis</td></tr>
<tr><td colspan="2">Long Name</td><td>Short Name<br>(Format: NCBI accession number-virus-country/state)</td><td>Country or State of collection</td><td colspan="3">Collection date</td></tr>
<tr><td colspan="2">MF176277-Culex-negev-like-virus3-strain-mos172X44875-complete-genome</td><td>MF176277-CNLV3-WA</td><td>Western Australia</td><td colspan="3">2015</td></tr>
<tr><td colspan="2">MF281708-Biggievirus-Mos11-strain-258719/2008-ORF1-gene-partial-cds-and-ORF2-and-ORF3-genes-complete-cds</td><td>MF281708-BV-M11-IT</td><td>Italy</td><td colspan="3">2008</td></tr>
<tr><td colspan="2">MF281709-Biggievirus-Mos11-strain-pool-11/2008-ORF1-gene-partial-cds-and-ORF2-and-ORF3-genes-complete-cds</td><td>MF281709-BV-M11-IT</td><td>Italy</td><td colspan="3">2008</td></tr>
<tr><td colspan="2">KX924639-Biggievirus-Mos11-replicase-large-subunit-gene-partial-cds-and-hypothetical-protein-genes-complete-cds</td><td>KX924639-BV-M11-US</td><td>USA</td><td colspan="3">2016</td></tr>
</table>

The table points towards the molecular phylogenetic analysis by Maximum Likelihood method of CNLV3. The first half of the table provides details to identify the sample from which the virus was isolated, protein that encodes the consensus sequences that are being analysed, the length of the consensus sequences thus providing the depth of the gene being analysed, minimum mapping quality threshold used in IGV for the generation of the consensus sequences, the coverage of the consensus sequences from IGV, the year of collection of the sample and the NCBI accession number assigned to the particular consensus sequence. Corresponding short names have been used in the phylogenetic trees which contain the sample, virus, protein and the number of nucleotides. The second half of the table provides the details of the sequences that were used for the molecular

phylogenetic analysis. They were found to be the most closely related sequences to the consensus sequences generated using BLASTN. Corresponding short names have been used in the phylogenetic trees which contain the NCBI accession number, virus and the country of collection. The collection date is given as retrieved from the corresponding NCBI nucleotide reference dataset which together with the country of collection can provide an insight into the possible evolution of the virus through the years.

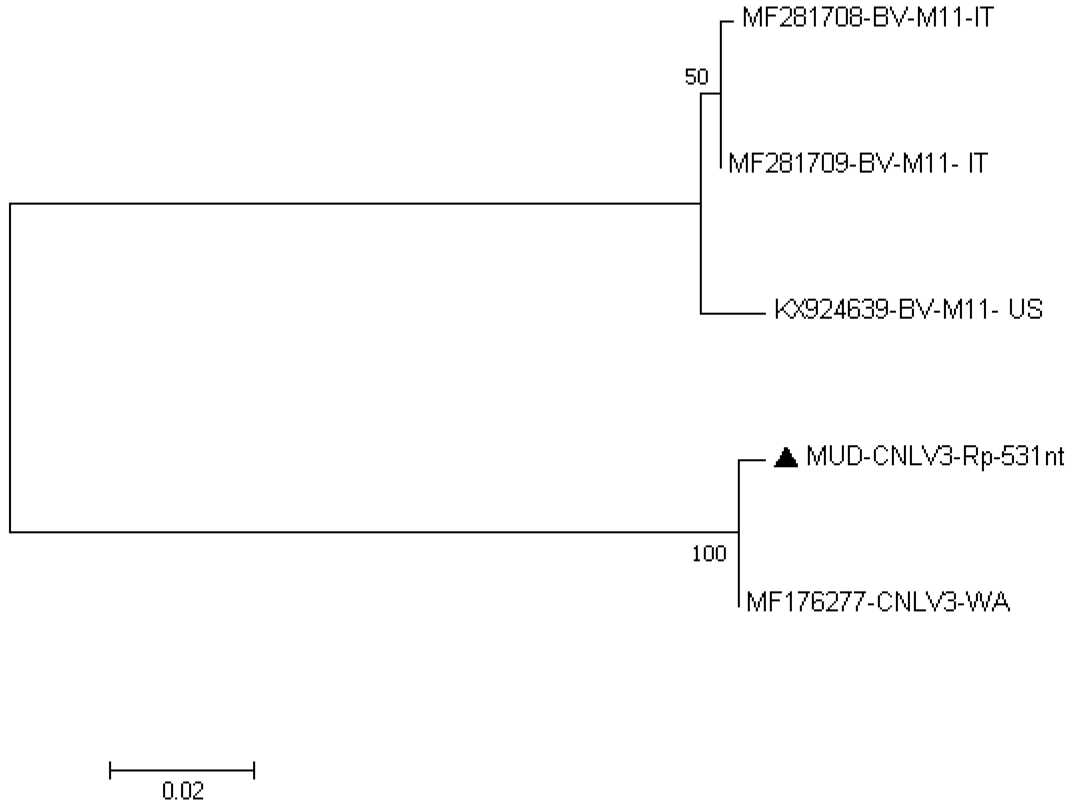


**Figure S8.1: Molecular Phylogenetic analysis by Maximum Likelihood method of CNLV3 partial Rp gene**
The evolutionary history was inferred by using the Maximum Likelihood method based on the Tamura 3-parameter model[4]. The tree with the highest log likelihood (-1086.99) is shown. The percentage of trees in which the associated taxa clustered together is shown next to the branches. Initial tree(s) for the heuristic search were obtained automatically by applying Neighbor-Join and BioNJ algorithms to a matrix of pairwise distances estimated using the Maximum Composite Likelihood (MCL) approach, and then selecting the topology with superior log likelihood value. The tree is drawn to scale, with branch lengths measured in the number of substitutions per site. The analysis involved 5 nucleotide sequences. Codon positions included were 1st+2nd+3rd+Noncoding. All positions containing gaps and missing data were eliminated. There were a total of 531 positions in the final dataset. Evolutionary analyses were conducted in MEGA7[2].

**Table S8.1: Estimates of Evolutionary Divergence between Sequences**
The number of base differences per sequence from between sequences are shown. The analysis involved 5 nucleotide sequences. Codon positions included were 1st+2nd+3rd+Noncoding. All positions containing gaps and missing data were eliminated. There were a total of 531 positions in the final dataset. Evolutionary analyses were conducted in MEGA7.

| | | | | |
|---|---|---|---|---|
| **MUD-CNLV3-Rp-531nt** | | | | |
| MF176277-CNLV3-WA | 2 | | | |
| MF281708-BV-M11-IT | 90 | 89 | | |
| MF281709-BV-M11-_IT | 91 | 90 | 1 | |
| KX924639-BV-M11-_US | 93 | 92 | 7 | 6 |

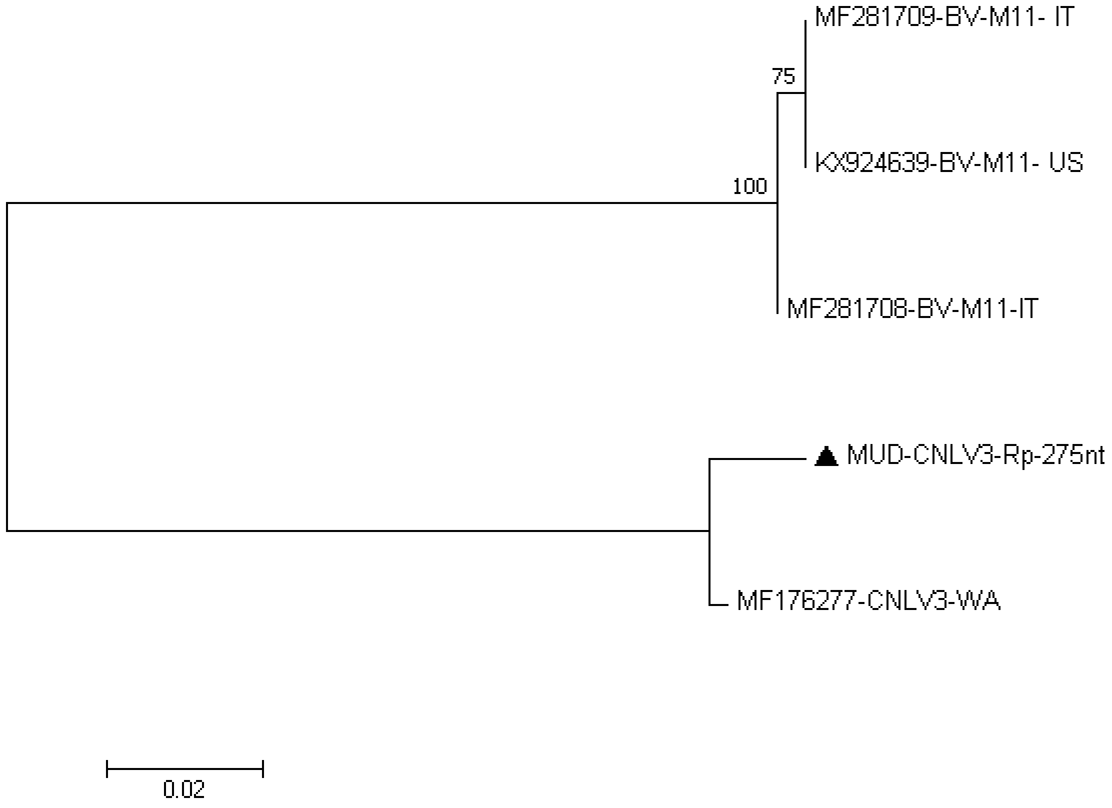


**Figure S8.2: Molecular Phylogenetic analysis by Maximum Likelihood method of CNLV3 partial Rp gene**
The evolutionary history was inferred by using the Maximum Likelihood method based on the Tamura 3-parameter model[4]. The tree with the highest log likelihood (-569.79) is shown. The percentage of trees in which the associated taxa clustered together is shown next to the branches. Initial tree(s) for the heuristic search were obtained automatically by applying Neighbor-Join and BioNJ algorithms to a matrix of pairwise distances estimated using the Maximum Composite Likelihood (MCL) approach, and then selecting the topology with superior log likelihood value. The tree is drawn to scale, with branch lengths measured in the number of substitutions per site. The analysis involved 5 nucleotide sequences. Codon positions included were 1st+2nd+3rd+Noncoding. All positions containing gaps and missing data were eliminated. There were a total of 275 positions in the final dataset. Evolutionary analyses were conducted in MEGA7[2].

**Table S8.2: Estimates of Evolutionary Divergence between Sequences**
The number of base differences per sequence from between sequences are shown. The analysis involved 5 nucleotide sequences. Codon positions included were 1st+2nd+3rd+Noncoding. All positions containing gaps and missing data were eliminated. There were a total of 275 positions in the final dataset. Evolutionary analyses were conducted in MEGA7.

| **MUD-CNLV3-Rp-275nt** | | | | |
|---|---|---|---|---|
| MF176277-CNLV3-WA | 4 | | | |
| MF281708-BV-M11-IT | 48 | 46 | | |
| MF281709-BV-M11-_IT | 49 | 47 | 1 | |
| KX924639-BV-M11-_US | 49 | 47 | 1 | 0 |

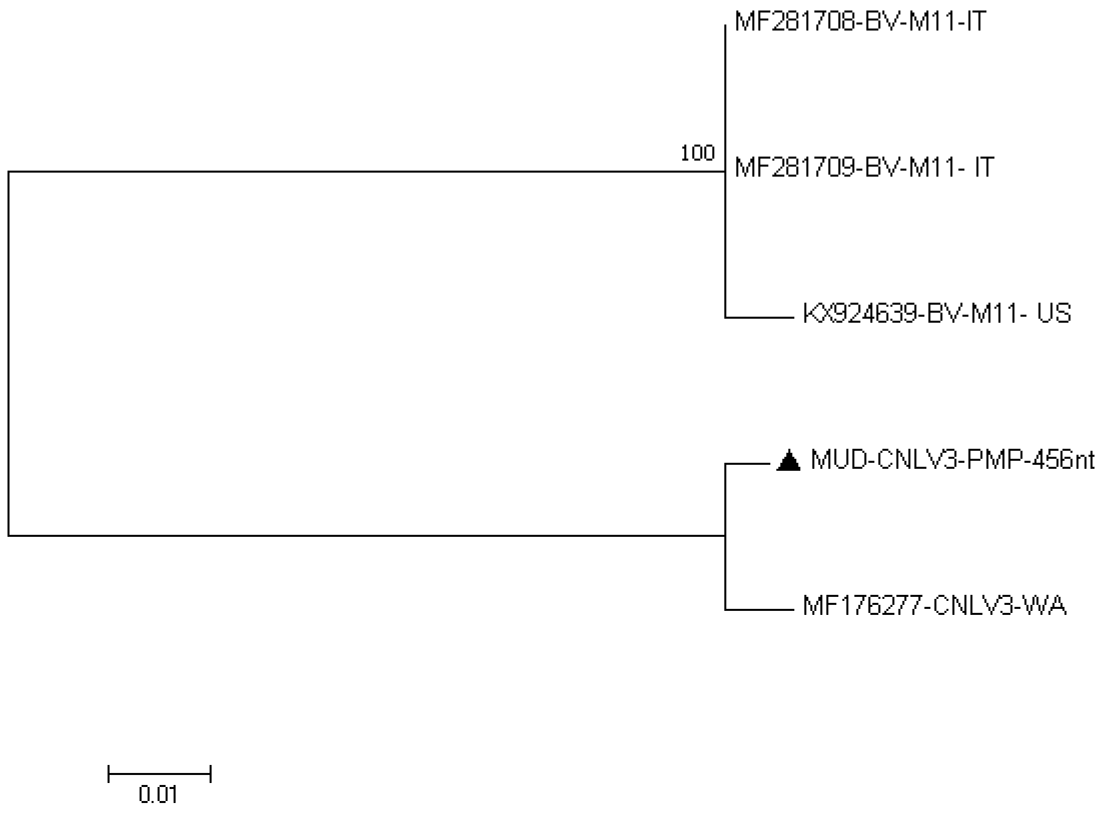


**Figure S8.3: Molecular Phylogenetic analysis by Maximum Likelihood method of CNLV3 partial PMP gene**
The evolutionary history was inferred by using the Maximum Likelihood method based on the Hasegawa-Kishino-Yano model[5]. The tree with the highest log likelihood (-893.63) is shown. The percentage of trees in which the associated taxa clustered together is shown next to the branches. Initial tree(s) for the heuristic search were obtained automatically by applying Neighbor-Join and BioNJ algorithms to a matrix of pairwise distances estimated using the Maximum Composite Likelihood (MCL) approach, and then selecting the topology with superior log likelihood value. The tree is drawn to scale, with branch lengths measured in the number of substitutions per site. The analysis involved 5 nucleotide sequences. Codon positions included were 1st+2nd+3rd+Noncoding. All positions containing gaps and missing data were eliminated. There were a total of 456 positions in the final dataset. Evolutionary analyses were conducted in MEGA7[2].

**Table S8.3: Estimates of Evolutionary Divergence between Sequences**
The number of base differences per sequence from between sequences are shown. The analysis involved 5 nucleotide sequences. Codon positions included were 1st+2nd+3rd+Noncoding. All positions containing gaps and missing data were eliminated. There were a total of 456 positions in the final dataset. Evolutionary analyses were conducted in MEGA7.

| **MUD-CNLV3-PMP-456nt** | | | | |
|---|---|---|---|---|
| MF176277-CNLV3-WA | 5 | | | |
| MF281708-BV-M11-IT | 59 | 60 | | |
| MF281709-BV-M11-_IT | 59 | 60 | 0 | |
| KX924639-BV-M11-_US | 62 | 61 | 3 | 3 |

9. **Hubei reo-like virus 7 like virus from MUD faecal sample**

| Figure and Table | Long Name (Format: Sample-virus-protein-length-quality-coverage-year) | Short name (Format: Sample-virus-protein-length) | Coverage | No. of nucleotides | Mapping quality threshold | NCBI accession number |
|---|---|---|---|---|---|---|
| S9.1 | MUD-Hubei-reo-like-virus7-Rp-240nt-Q20-C-3-11-2016 | MUD-HRLV7L-Rp-240nt | 3-11 | 240 | 20 | MH085099 |
| S9.2 | MUD-Hubei-reo-like-virus7-Rp-184nt-Q32-C-3-6-2016 | MUD-HRLV7L-Rp-184nt | 3-6 | 184 | 32 | MH085100 |
| S9.3 | MUD-Hubei-reo-like-virus7-Rp-117nt-Q32-C-4-5-2016 | MUD-HRLV7L-Rp-117nt | 4-5 | 117 | 32 | MH085101 |
| NCBI sequences taken for phylogenetic analysis | | | | | | |
| Long Name | | Short Name (Format: NCBI accession number-virus-country/state) | Country of collection | Collection date | | |
| KX884635-Hubei-reo-like-virus7-strain-mosHB235771-Rp-gene-complete-cds | | KX884635-HRLV7-CN | China | 2013 | | |
| DQ087277-Aedes-pseudoscutellaris-reovirus-segment2-complete-sequence | | DQ087277-APRV-FR | France | - | | |
| KM978429-Fako-virus-strain-CSW87-segment2-RNA-dependent-RNA-polymerase-gene-complete-cds | | KM978429-FV-CM | Cameroon | 2010 | | |
| KX884633-Hubei-mosquito-virus5-strain-mosHB233040-RdRp-gene-partial-cds | | KX884633-HMV5-CN | China | 2013 | | |

The table points towards the molecular phylogenetic analysis by Maximum Likelihood method of HRLV7L. The first half of the table provides details to identify the sample from which the virus was isolated, protein that encodes the consensus sequences that are being analysed, the length of the consensus sequences thus providing the depth of the gene being analysed, minimum mapping quality threshold used in IGV for the generation of the consensus sequences, the coverage of the consensus sequences from IGV, the year of collection of the sample and the NCBI accession number assigned to the particular consensus sequence. Corresponding short names have been used in the phylogenetic trees which contain the sample, virus, protein and the number of nucleotides. The second half of the table provides the details of the sequences that were used for the molecular phylogenetic analysis. They were found to be the most closely related sequences to the consensus sequences generated using BLASTN. Corresponding short names have been used in the phylogenetic trees which contain the NCBI accession number, virus and the country of collection.

The collection date is given as retrieved from the corresponding NCBI nucleotide reference dataset which together with the country of collection can provide an insight into the possible evolution of the virus through the years.

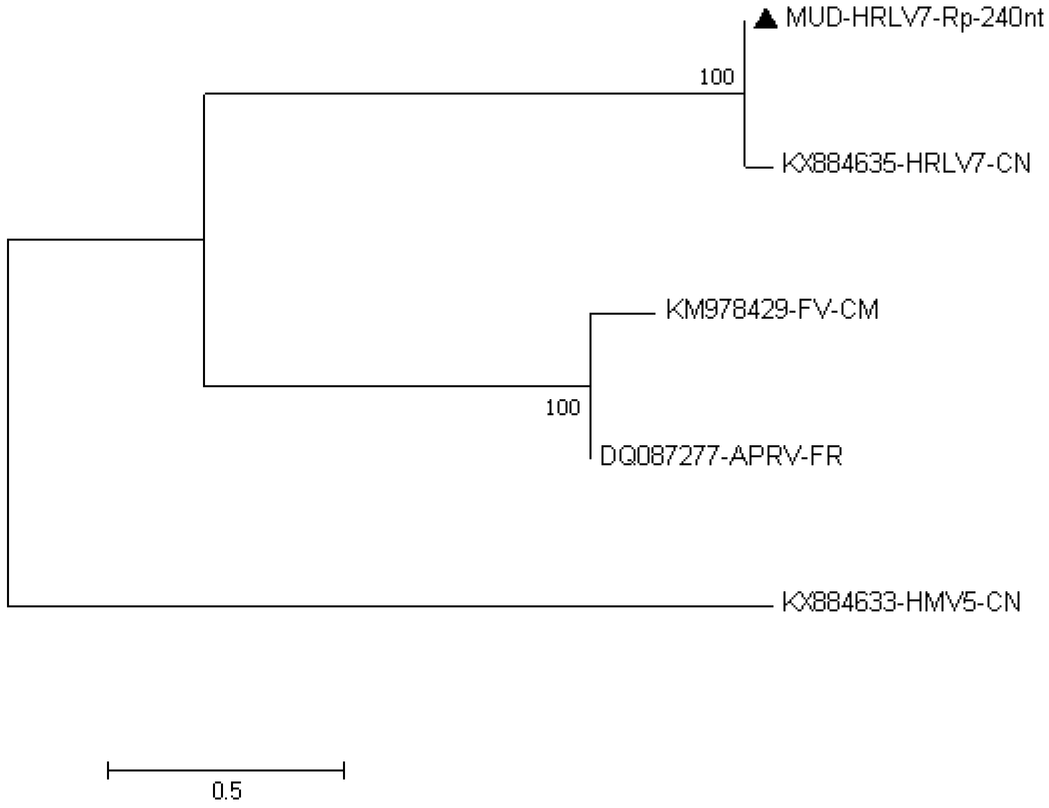


**Figure S9.1: Molecular Phylogenetic analysis by Maximum Likelihood method of HRLV7L partial Rp gene**
The evolutionary history was inferred by using the Maximum Likelihood method based on the Tamura 3-parameter model[4]. The tree with the highest log likelihood (-989.22) is shown. The percentage of trees in which the associated taxa clustered together is shown next to the branches. Initial tree(s) for the heuristic search were obtained automatically by applying Neighbor-Join and BioNJ algorithms to a matrix of pairwise distances estimated using the Maximum Composite Likelihood (MCL) approach, and then selecting the topology with superior log likelihood value. The rate variation model allowed for some sites to be evolutionarily invariable ([+I], 31.77% sites). The tree is drawn to scale, with branch lengths measured in the number of substitutions per site. The analysis involved 5 nucleotide sequences. Codon positions included were 1st+2nd+3rd+Noncoding. All positions containing gaps and missing data were eliminated. There were a total of 239 positions in the final dataset. Evolutionary analyses were conducted in MEGA7[2].

**Table S9.1: Estimates of Evolutionary Divergence between Sequences**
The number of base differences per sequence from between sequences are shown. The analysis involved 5 nucleotide sequences. Codon positions included were 1st+2nd+3rd+Noncoding. All positions containing gaps and missing data were eliminated. There were a total of 239 positions in the final dataset. Evolutionary analyses were conducted in MEGA7.

| **MUD-HRLV7L-Rp-240nt** | | | | |
|---|---|---|---|---|
| KX884635-HRLV7-CN | 13 | | | |
| KX884633-HMV5-CN | 112 | 111 | | |
| KM978429-FV-CM | 97 | 97 | 97 | |
| DQ087277-APRV-FR | 90 | 91 | 93 | 27 |

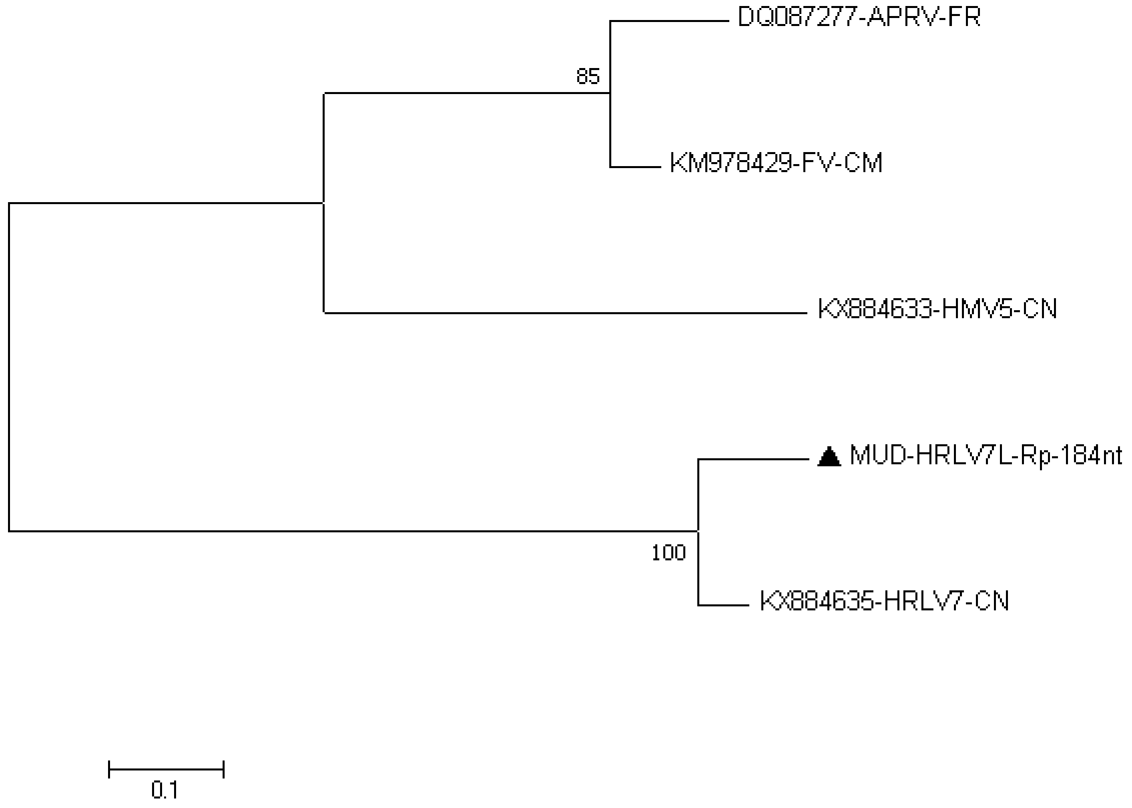


**Figure S9.2: Molecular Phylogenetic analysis by Maximum Likelihood method of HRLV7L partial Rp gene**
The evolutionary history was inferred by using the Maximum Likelihood method based on the Tamura 3-parameter model[4]. The tree with the highest log likelihood (-822.59) is shown. The percentage of trees in which the associated taxa clustered together is shown next to the branches. Initial tree(s) for the heuristic search were obtained automatically by applying Neighbor-Join and BioNJ algorithms to a matrix of pairwise distances estimated using the Maximum Composite Likelihood (MCL) approach, and then selecting the topology with superior log likelihood value. A discrete Gamma distribution was used to model evolutionary rate differences among sites (5 categories (+G, parameter = 2.1378)). The tree is drawn to scale, with branch lengths measured in the number of substitutions per site. The analysis involved 5 nucleotide sequences. Codon positions included were 1st+2nd+3rd+Noncoding. All positions containing gaps and missing data were eliminated. There were a total of 184 positions in the final dataset. Evolutionary analyses were conducted in MEGA7[2].

**Table S9.2. Estimates of Evolutionary Divergence between Sequences**
The number of base differences per sequence from between sequences are shown. The analysis involved 5 nucleotide sequences. Codon positions included were 1st+2nd+3rd+Noncoding. All positions containing gaps and missing data were eliminated. There were a total of 184 positions in the final dataset. Evolutionary analyses were conducted in MEGA7.

| **MUD-HRLV7L-Rp-184nt** | | | | |
|---|---|---|---|---|
| KX884635-HRLV7-CN | 23 | | | |
| DQ087277-APRV-FR | 95 | 85 | | |
| KM978429-FV-CM | 87 | 87 | 23 | |
| KX884633-HMV5-CN | 98 | 95 | 72 | 70 |

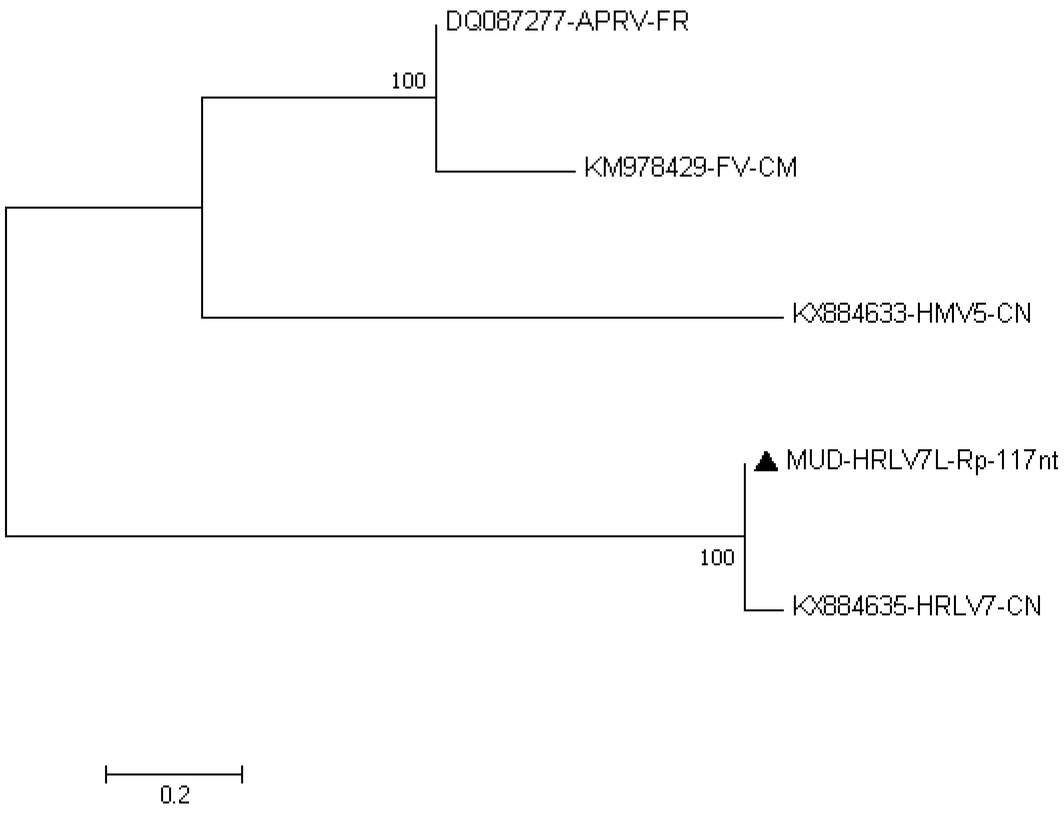


**Figure S9.3: Molecular Phylogenetic analysis by Maximum Likelihood method of HRLV7L partial Rp gene**
The evolutionary history was inferred by using the Maximum Likelihood method based on the Tamura 3-parameter model[4]. The tree with the highest log likelihood (-516.09) is shown. The percentage of trees in which the associated taxa clustered together is shown next to the branches. Initial tree(s) for the heuristic search were obtained automatically by applying Neighbor-Join and BioNJ algorithms to a matrix of pairwise distances estimated using the Maximum Composite Likelihood (MCL) approach, and then selecting the topology with superior log likelihood value. The rate variation model allowed for some sites to be evolutionarily invariable ([+I], 20.42% sites). The tree is drawn to scale, with branch lengths measured in the number of substitutions per site. The analysis involved 5 nucleotide sequences. Codon positions included were 1st+2nd+3rd+Noncoding. All positions containing gaps and missing data were eliminated. There were a total of 117 positions in the final dataset. Evolutionary analyses were conducted in MEGA7[2].

**Table S9.3: Estimates of Evolutionary Divergence between Sequences**
The number of base differences per sequence from between sequences are shown. The analysis involved 5 nucleotide sequences. Codon positions included were 1st+2nd+3rd+Noncoding. All positions containing gaps and missing data were eliminated. There were a total of 117 positions in the final dataset. Evolutionary analyses were conducted in MEGA7.

| **MUD-HRLV7L-Rp-117nt** | | | | |
|---|---|---|---|---|
| KX884635-HRLV7-CN | 6 | | | |
| DQ087277-APRV-FR | 56 | 58 | | |
| KM978429-FV-CM | 53 | 56 | 19 | |
| KX884633-HMV5-CN | 63 | 63 | 53 | 58 |

10. **Enterobacteria phage phi92 from MUD faecal sample**

| Figure and Table | Long Name (Format: Sample-virus-protein-length-quality-coverage-year) | Short name (Format: Sample-virus-protein-length) | Coverage | No. of nucleotides | Mapping quality threshold | NCBI accession number |
|---|---|---|---|---|---|---|
| S10.1 | MUD-Enterobacteria-phage-phi92-Phi92-gp141-277nt-Q32-C-2-4-2016 | MUD-EPP92-g141-277nt | 2-4 | 277 | 32 | MH188081 |
| S10.2 | MUD-Enterobacteria-phage-phi92-Phi92-gp141-aa-276nt-Q32-C-2-4-2016 | MUD-EPP92-g141-aa-276nt | 2-4 | 276 | 32 | MH188081 |
| NCBI sequences taken for phylogenetic analysis | | | | | | |
| Long Name | | Short Name (Format: NCBI accession number-virus-country/state) | Country of collection | Collection date | | |
| FR775895-Enterobacteria-phage-phi92-complete-genome | | FR775895-EPP92 | - | - | | |
| KU522583-Enterobacteria-phage-ECGD1-complete-genome | | KU522583-EP | - | 2015 | | |
| KX552041-Escherichia-phage-ESCO13-complete-genome | | KX552041-EsP-FR | France | 2014 | | |
| KX664695-Escherichia-phage-ESCO5-complete-genome | | KX664695-EsP-FR | France | 2014 | | |
| JX561091-Escherichia-phage-phAPEC8-complete-genome | | JX561091-EsP-BE | Belgium | 2010 | | |
| KR296694-Salmonella-phage-40-complete-genome | | KR296694-SP40-IN | India | 2002 | | |

The table points towards the molecular phylogenetic analysis by Maximum Likelihood method of EPP92. The first half of the table provides details to identify the sample from which the virus was isolated, protein that encodes the consensus sequences that are being analysed, the length of the consensus sequences thus providing the depth of the gene being analysed, minimum mapping quality threshold used in IGV for the generation of the consensus sequences, the coverage of the consensus sequences from IGV, the year of collection of the sample and the NCBI accession number assigned to the particular consensus sequence. Corresponding short names have been used in the phylogenetic trees which contain the sample, virus, protein and the number of nucleotides. In case of the amino acid sequence analysis, the short names contain the “aa” after the protein. The second half of the table provides the details of the sequences that were used for the molecular phylogenetic analysis. They were found to be the most closely related sequences to the consensus

sequences generated using BLASTN. Corresponding short names have been used in the phylogenetic trees which contain the NCBI accession number, virus and the country of collection. The collection date is given as retrieved from the corresponding NCBI nucleotide reference dataset which together with the country of collection can provide an insight into the possible evolution of the virus through the years.

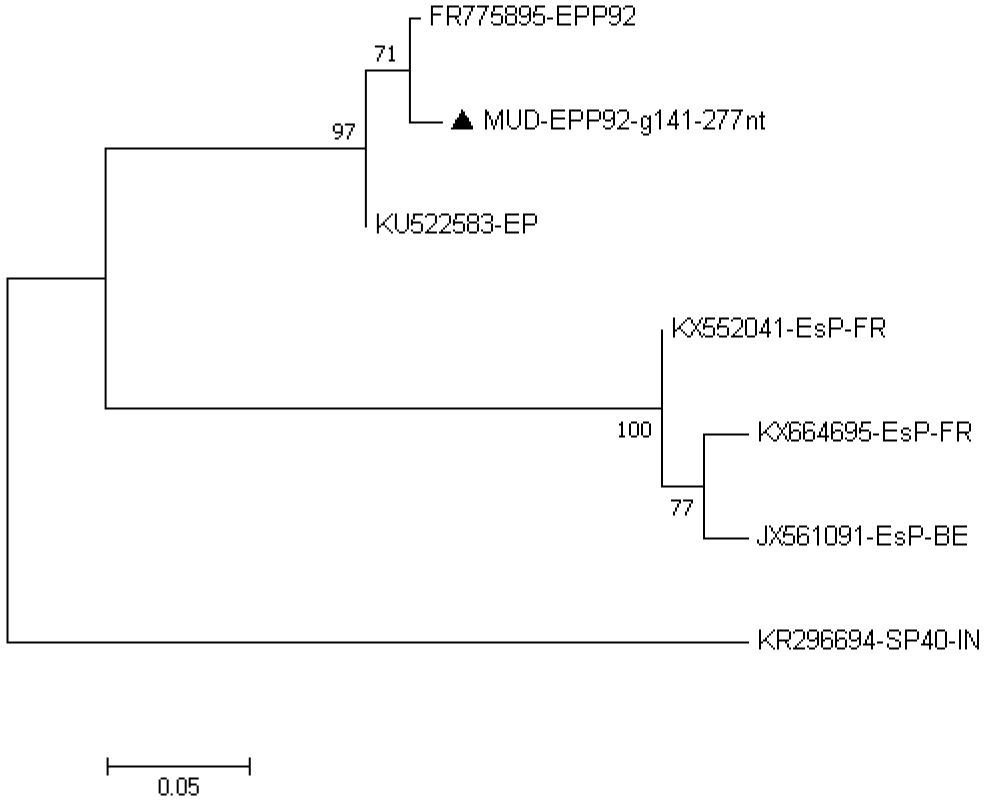


**Figure S10.1: Molecular Phylogenetic analysis by Maximum Likelihood method of EPP92 partial g141 gene**
The evolutionary history was inferred by using the Maximum Likelihood method based on the Tamura 3-parameter model[4]. The tree with the highest log likelihood (-899.41) is shown. The percentage of trees in which the associated taxa clustered together is shown next to the branches. Initial tree(s) for the heuristic search were obtained automatically by applying Neighbor-Join and BioNJ algorithms to a matrix of pairwise distances estimated using the Maximum Composite Likelihood (MCL) approach, and then selecting the topology with superior log likelihood value. A discrete Gamma distribution was used to model evolutionary rate differences among sites (5 categories (+G, parameter = 0.4004)). The tree is drawn to scale, with branch lengths measured in the number of substitutions per site. The analysis involved 7 nucleotide sequences. Codon positions included were 1st+2nd+3rd+Noncoding. All positions containing gaps and missing data were eliminated. There were a total of 277 positions in the final dataset. Evolutionary analyses were conducted in MEGA7[2].

**Table S10.1: Estimates of Evolutionary Divergence between Sequences**
The number of base differences per sequence from between sequences are shown. The analysis involved 7 nucleotide sequences. Codon positions included were 1st+2nd+3rd+Noncoding. All positions containing gaps and missing data were eliminated. There were a total of 277 positions in the final dataset. Evolutionary analyses were conducted in MEGA7.

| | | | | | | |
|---|---|---|---|---|---|---|
| FR775895-EPP92 | | | | | | |
| **MUD-EPP92-g141-277nt** | 4 | | | | | |
| KU522583-EP | 5 | 7 | | | | |
| KX552041-EsP-FR | 54 | 54 | 52 | | | |
| KX664695-EsP-FR | 57 | 57 | 55 | 8 | | |
| JX561091-EsP-BE | 61 | 61 | 59 | 8 | 8 | |
| KR296694-SP40-IN | 69 | 67 | 66 | 78 | 77 | 75 |

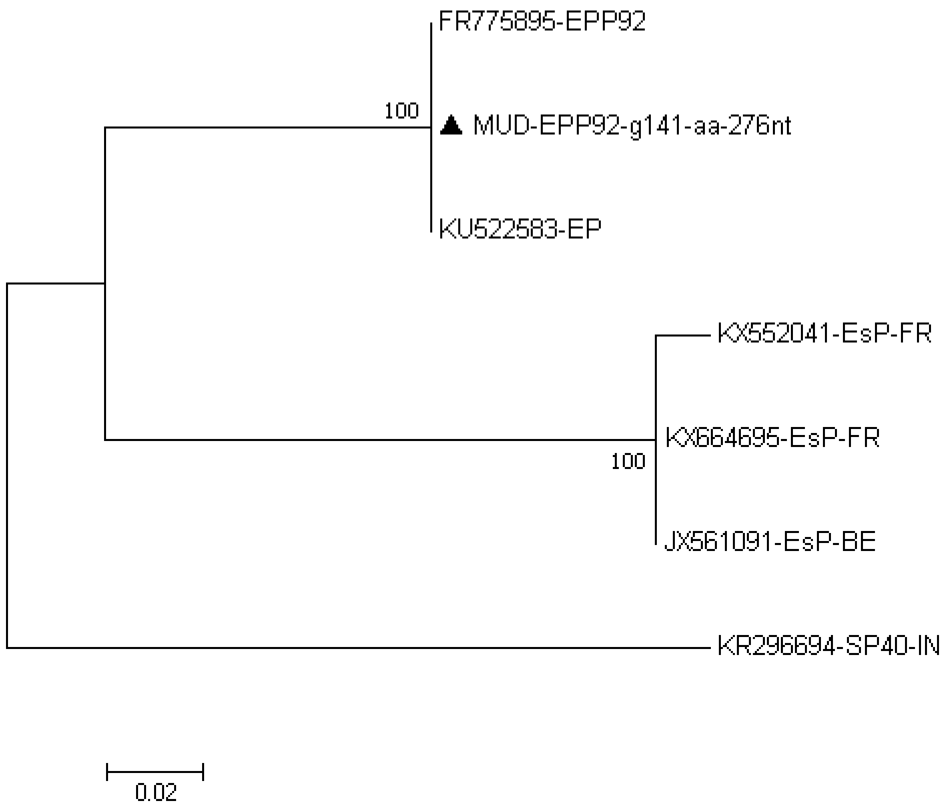


**Figure S10.2: Molecular Phylogenetic analysis by Maximum Likelihood method for amino acid sequences of EPP92 partial g141 gene**

The evolutionary history was inferred by using the Maximum Likelihood method based on the Le_Gascuel_2008 model[6]. The tree with the highest log likelihood (-431.10) is shown. The percentage of trees in which the associated taxa clustered together is shown next to the branches. Initial tree(s) for the heuristic search were obtained automatically by applying Neighbor-Join and BioNJ algorithms to a matrix of pairwise distances estimated using a JTT model, and then selecting the topology with superior log likelihood value. The tree is drawn to scale, with branch lengths measured in the number of substitutions per site. The analysis involved 7 amino acid sequences. All positions containing gaps and missing data were eliminated. There were a total of 92 positions in the final dataset. Evolutionary analyses were conducted in MEGA7[2].

**Table S10.2: Estimates of Evolutionary Divergence between Sequences**

The number of amino acid differences per sequence from between sequences are shown. The analysis involved 7 amino acid sequences. All positions containing gaps and missing data were eliminated. There were a total of 92 positions in the final dataset. Evolutionary analyses were conducted in MEGA7.

| | | | | | | |
|---|---|---|---|---|---|---|
| FR775895-EPP92 | | | | | | |
| **MUD-EPP92-g141-aa-276nt** | 0 | | | | | |
| KU522583-EP | 0 | 0 | | | | |
| KX552041-EsP-FR | 15 | 15 | 15 | | | |
| KX664695-EsP-FR | 15 | 15 | 15 | 1 | | |
| JX561091-EsP-BE | 15 | 15 | 15 | 1 | 0 | |
| KR296694-SP40-IN | 19 | 19 | 19 | 21 | 21 | 21 |

### 11. Human viruses from ST4, ST5 and ST6 faecal samples

| Figure and Table | Long Name (Format: Sample-virus-protein-length-quality-coverage-year) | Short name (Format: Sample-virus-protein-length) | Coverage | No. of nucleotides | Mapping quality threshold | NCBI accession number |
|---|---|---|---|---|---|---|
| ssDNA viruses | | | | | | |
| S11.1 | ST6- Human-fecal-virus-Jorvi2-hypothetical-protein-208nt-Q46-C-1-2017 | ST6-HFVJ2-HP-208nt | 1 | 208 | 46 | MH188072 |
| S11.2 | ST4-Torque-teno-mini-virus-5prime-untranslated-116nt-Q10-C-11-15-2017 | ST4-TTMV-5UTR-116nt | 11-15 | 116 | 10 | MH188073 |
| S11.2 | ST5-Torque-teno-mini-virus-5prime-untranslated-116nt-Q10-C-10-13-2017 | ST5-TTMV-5UTR-116nt | 10-13 | 116 | 10 | MH188073 |
| Picornaviruses | | | | | | |
| S11.3 | ST4-Human-rhinovirus-C- genome-capsid-protein-VP2-163nt-Q8-C-5-6-2017 | ST4-HRVC-VP2-163nt | 5-6 | 163 | 8 | MH188075 |
| S11.4 | ST6-Human-TMEV-like-cardiovirus-polyprotein-261nt-Q10-C-3-15-2017 | ST6-HTMEVLCV-VP1-261nt | 3-15 | 261 | 10 | MH188076 |
| S11.5 | ST6-H Human-TMEV-like-cardiovirus-capsid protein-VP3-422nt-Q10-C-3-18-2017 | ST6-HTMEVLCV-VP3-422nt | 3-18 | 422 | 10 | MH188077 |
| S11.6 | ST6-Saffold-virus-protein-2C-212nt-Q10-C-2-4-2017 | ST6-SV-2C-212nt | 2-4 | 212 | 10 | MH188078 |
| Contigs assembled | | | | | | |
| S11.7 | ST4-RotavirusA-VP4-909nt-Q20-C-3-52-2017 | ST4-RVA-VP4-909nt | 3-52 | 909 | 20 | MH188079 |
| S11.8 | ST5-Norovirus-contig-7044nt-Q20-C-2-189-2017 | ST5-NV-7044nt | 2-189 | 7044 | 20 | MH188080 |
| NCBI sequences taken for phylogenetic analysis | | | | | | |
| Long Name | | Short Name (Format: NCBI accession number-virus-country/state) | Country of collection | Collection date | | |

| | | | |
|---|---|---|---|
| MF118166-Human-fecal-virus-Tarto-complete-genome | MF118166-HFVT-EE | Estonia | - |
| MF118167-Human-fecal-virus-Jorvi2-complete-genome | MF118167-HFVJ2-FI | Finland | - |
| KT163899-Torque-teno-virus-isolate-P13-4-ORF1-gene-partial-cds | KT163899-TTV | - | - |
| KM259873-Torque-teno-mini-virus-ALA22-complete-genome | KM259873-TTMV-ES | Spain | 2014 |
| EF538883-TTV-like-mini-virus-isolate-LIL-y4-ORF2-and-ORF1-genes-complete-cds | EF538883-TTVLMV-FR | France | - |
| KX810063-TTV-like-mini-virus-isolate-Emory1-complete-genome | KX810063-TTVLMV-US | USA | 1993 |
| AB041962-Torque-teno-mini-virus5-DNA-complete-genome-isolate:TGP96 | AB041962-TTMV5 | - | - |
| KY983589-Human-rhinovirus-C11-strain-SC3107-complete-sequence | KY983589-HRVC-US | USA | 2015 |
| KM486097-Rhinovirus-C-strain-Mex14-Consensus-polyprotein-gene-complete-cds | KM486097-RVC-MX | Mexico | 2014 |
| JN815249-Human-rhinovirus-C-strain-HRV-C43-p1281-s6410-1999-polyprotein-gene-partial-cds | JN815249-HRVC-US | USA | 1999 |
| KY369878-Human-rhinovirus-C43-strain-SC174-complete-genome | KY369878-HRVC43-US | USA | 2016 |
| JX074056-Human-rhinovirus-C-strain-HRV-C43-p1154-sR1124-2009-polyprotein-gene-complete-cds | JX074056-HRVC43-US | USA | 2009 |
| LC004883-Rhinovirus-C-gene-for-polyprotein-VP2-VP3-VP1-region-partial-cds-strain:HRV-C/JPN/Yokohama63/2012 | LC004883-RVC-JP | Japan | 2012 |
| AB747252-Saffold-virus-gene-for-polyprotein-partial-cds-isolate:Pak-3290 | AB747252-SV-PK | Pakistan | 2009 |
| AB747248-Saffold-virus-gene-for-polyprotein-partial-cds-isolate:Pak-3097 | AB747248-SV-PK | Pakistan | 2009 |
| FJ463616-Saffold-virus-isolate-Pak5152-polyprotein-(gp1)-gene-partial-cds-and-L*-protein-(L*)-gene-complete-cds | FJ463616-SV-PK | Pakistan | 2009 |

| | | | |
|---|---|---|---|
| AB747255-Saffold-virus-gene-for-polyprotein-partial-cds-isolate:Pak-3486 | AB747255-SV-PK | Pakistan | 2009 |
| AB747250-Saffold-virus-gene-for-polyprotein-partial-cds-isolate:Pak-3641 | AB747250-SV-PK | Pakistan | 2009 |
| EF165067-Saffold-virus-complete-genome | EF165067-SV-US | USA | - |
| GU595289-Human-TMEV-like-cardiovirus-isolate-HTCV-UC6-complete-genome | GU595289-HTMEVLCV-US | USA | 2001 |
| EU681179-Cardiovirus-D/VI2223/2004-polyprotein-gene-complete-cds | EU681179-CV-DE | Germany | - |
| EU376394-Human-TMEV-like-cardiovirus-complete-genome | EU376394-HTMEVLCV | - | - |
| EU681176-Cardiovirus-D/VI2229/2004-polyprotein-gene-complete-cds | EU681176-CV-DE | Germany | - |
| JN652233-Saffold-virus-strain-S19-polyprotein-gene-complete-cds | JN652233-SA-US | USA | 2005 |
| JF813004-Saffold-virus-strain-Can112051-06-adapted-complete-genome | JF813004-SV-CA | Canada | 2007 |
| GU565088-RotavirusA-strain-RVA/Vaccine/USA/RotaTeq-BrB-9/1996/G4P75-VP4-gene-complete-cds | GU565088-RVA-US | USA | 1996 |
| GU565077-RotavirusA-strain-RVA/Vaccine/USA/RotaTeq-WI78-8/1992/G3P75-VP4-gene-complete-cds | GU565077-RVA-US | USA | 1992 |
| D16351-Rotavirus-sp.-VP4-mRNA-encoding-VP5-and-VP8-complete-cds | D16351-RV | - | - |
| KC815661-Equine-rotavirusA-genotype-G6-P5-I2-R2-C2-M2-A13-N2-T6-E2-H3-outer-capsid-spike-protein-(VP4)-gene-complete-cds | KC815661-ERVA-JP | Japan | 1982 |
| L42980-Rotavirus-sp.-mRNA-fragment | L42980-RV | - | - |
| U53923-Bovine-rotavirus-spike-protein-VP4-(VP4)-mRNA-complete-cds | U53923-BRV-US | USA | - |
| MG002634-Norovirus-GII-strain-Hu/GII.P4-New-Orleans2009- | MG002634-NV-AU | Australia | 2017 |

| | | | |
|---|---|---|---|
| GII.4-Sydney2012/BNE5/2017/AU-polyprotein-major-capsid-protein-and-minor-structural-protein-genes-complete-cds | | | |
| MG002632-Norovirus-GII-strain-Hu/GII.P4-New-Orleans2009-GII.4-Sydney2012/BNE3/2017/AU-polyprotein-major-capsid-protein-and-minor-structural-protein-genes-complete-cds | MG002632-NV-AU | Australia | 2017 |
| KJ685411-Norovirus-Hu/GII/BG1C0398/2012/BGD-partial-genome | KJ685411-NV-BD | Bangladesh | 2012 |
| MF140641-Norovirus-GII-strain_Norovirus/GII/Hu/NL/2013/GII.P4-GII.4/Rotterdam/E7800009-p11-d0-ORF1-gene-partial-cds-and-ORF2-and-ORF3-genes-complete-cds | MF140641-NV-NL | Netherlands | 2013 |
| MF140642.1-Norovirus-GII-strain-Norovirus/GII/Hu/NL/2013/GII.P4-GII.4/Rotterdam/E1300306-p11-d83-ORF1-gene-partial-cds-ORF2-gene-complete-cds-and-ORF3-gene-partial-cds | MF140642-NV-NL | Netherlands | 2013 |
| JX459901-Norovirus-Hu/GII.4/Caringbah/NSW409G/2011/AU-complete-genome | JX459901-NV-AU | Australia | 2011 |
| JX459904-Norovirus-Hu/GII.4/Doonside/NSW536I/2011/AU-complete-genome | JX459904-NV-AU | Australia | 2011 |

The table points towards the molecular phylogenetic analysis by Maximum Likelihood method of viruses isolated from human samples. The first half of the table provides details to identify the sample from which the virus was isolated, protein that encodes the consensus sequences that are being analysed, the length of the consensus sequences thus providing the depth of the gene being analysed, minimum mapping quality threshold used in IGV for the generation of the consensus sequences, the coverage of the consensus sequences from IGV, the year of collection of the sample and the NCBI accession number assigned to the particular consensus sequence. Corresponding short names have been used in the phylogenetic trees which contain the sample, virus, protein and the number of nucleotides. The second half of the table provides the details of the sequences that were used for the molecular phylogenetic analysis. They were found to be the most closely related sequences to the consensus sequences generated using BLASTN. Corresponding short names have been used in the phylogenetic trees which contain the NCBI accession number, virus and the

country of collection. The collection date is given as retrieved from the corresponding NCBI nucleotide reference dataset which together with the country of collection can provide an insight into the possible evolution of the virus through the years.

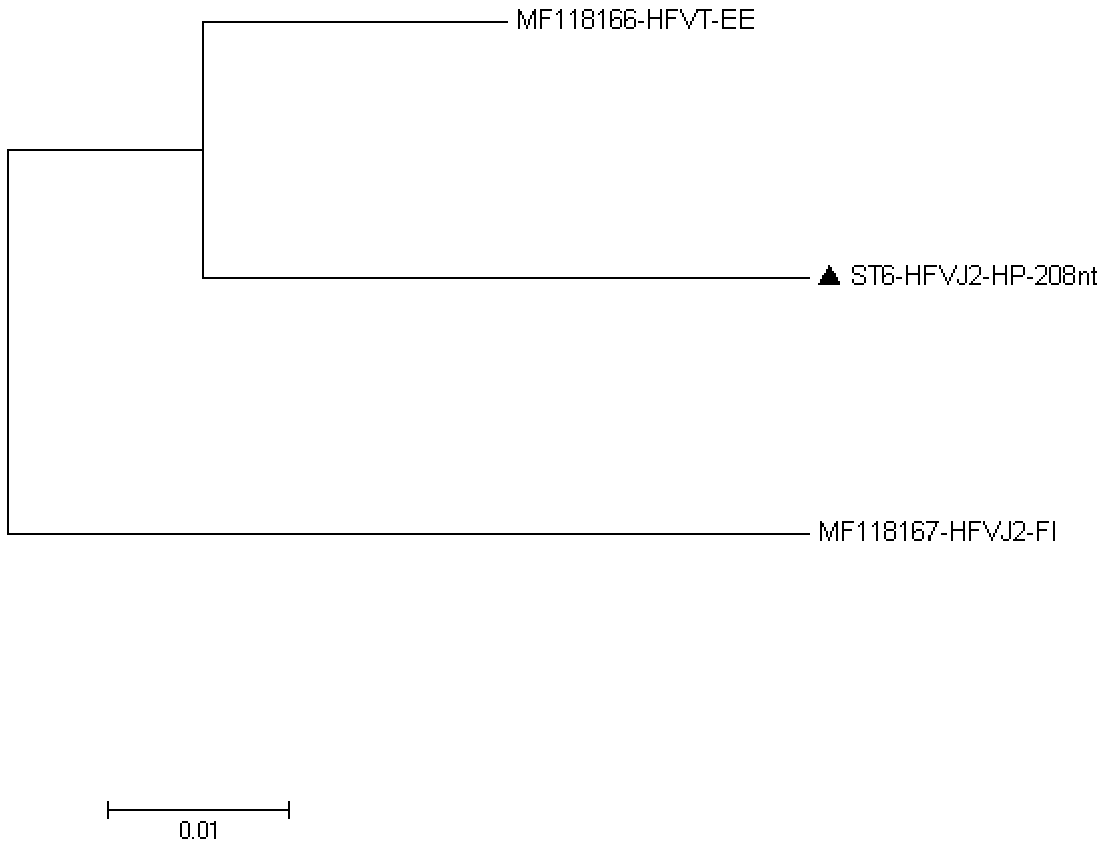


**Figure S11.1: Molecular Phylogenetic analysis by Maximum Likelihood method of HFVJ2 partial HP gene**
The evolutionary history was inferred by using the Maximum Likelihood method based on the Tamura 3-parameter model[4]. The tree with the highest log likelihood (-385.14) is shown. Initial tree(s) for the heuristic search were obtained automatically by applying Neighbor-Join and BioNJ algorithms to a matrix of pairwise distances estimated using the Maximum Composite Likelihood (MCL) approach, and then selecting the topology with superior log likelihood value. The tree is drawn to scale, with branch lengths measured in the number of substitutions per site. The analysis involved 3 nucleotide sequences. Codon positions included were 1st+2nd+3rd+Noncoding. All positions containing gaps and missing data were eliminated. There were a total of 208 positions in the final dataset. Evolutionary analyses were conducted in MEGA7[2].

**Table S11.1: Estimates of Evolutionary Divergence between Sequences**
The number of base differences per sequence from between sequences are shown. The analysis involved 3 nucleotide sequences. Codon positions included were 1st+2nd+3rd+Noncoding. All positions containing gaps and missing data were eliminated. There were a total of 208 positions in the final dataset. Evolutionary analyses were conducted in MEGA7.

| | | |
|---|---|---|
| MF118166-HFVT-EE | | |
| MF118167-HFVJ2-FI | 14 | |
| **ST6-HFVJ2-HP-208nt** | 10 | 17 |

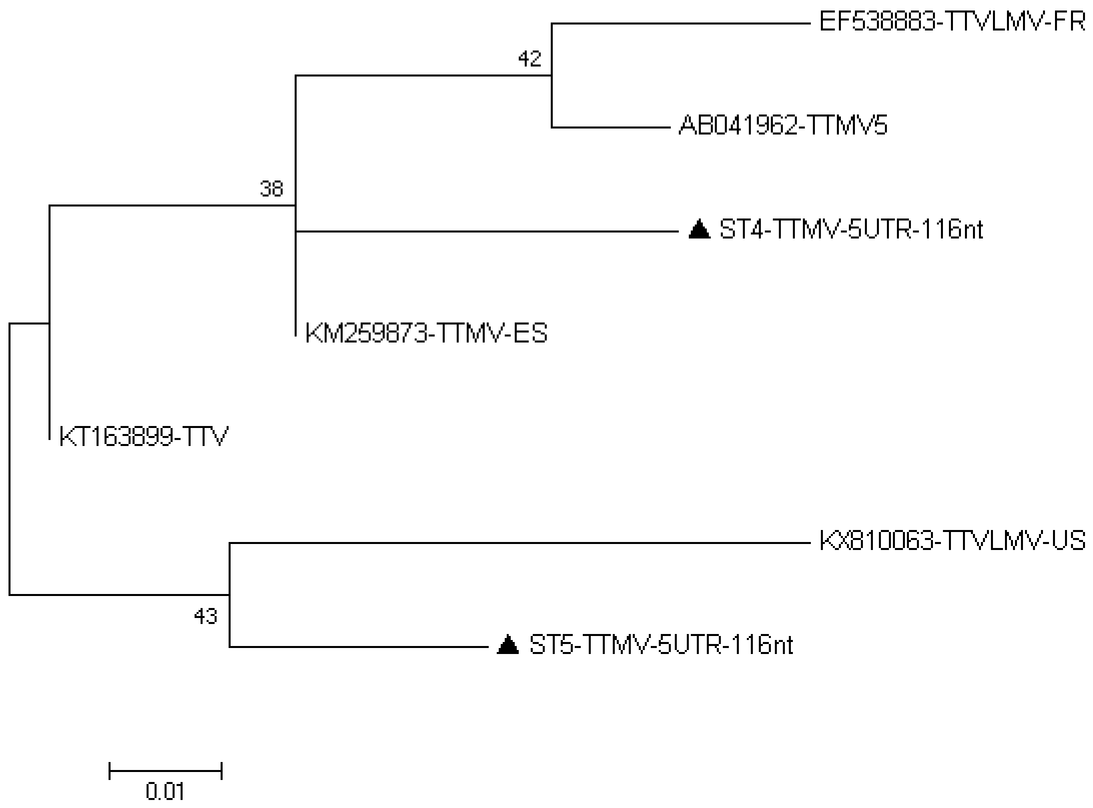


**Figure S11.2: Molecular Phylogenetic analysis by Maximum Likelihood method of TTMV partial 5UTR gene**
The evolutionary history was inferred by using the Maximum Likelihood method based on the Jukes-Cantor model[9]. The tree with the highest log likelihood (-260.34) is shown. The percentage of trees in which the associated taxa clustered together is shown next to the branches. Initial tree(s) for the heuristic search were obtained automatically by applying Neighbor-Join and BioNJ algorithms to a matrix of pairwise distances estimated using the Maximum Composite Likelihood (MCL) approach, and then selecting the topology with superior log likelihood value. A discrete Gamma distribution was used to model evolutionary rate differences among sites (5 categories (+G, parameter = 0.1000)). The tree is drawn to scale, with branch lengths measured in the number of substitutions per site. The analysis involved 7 nucleotide sequences. All positions containing gaps and missing data were eliminated. There were a total of 116 positions in the final dataset. Evolutionary analyses were conducted in MEGA7[2].

**Table S11.2: Estimates of Evolutionary Divergence between Sequences**
The number of base differences per sequence from between sequences are shown. The analysis involved 7 nucleotide sequences. Codon positions included were 1st+2nd+3rd+Noncoding. All positions containing gaps and missing data were eliminated. There were a total of 116 positions in the final dataset. Evolutionary analyses were conducted in MEGA7.

| | | | | | | |
|---|---|---|---|---|---|---|
| KT163899-TTV | | | | | | |
| KM259873-TTMV-ES | 2 | | | | | |
| EF538883-TTVLMV-FR | 6 | 4 | | | | |
| KX810063-TTVLMV-US | 6 | 8 | 8 | | | |
| AB041962-TTMV5 | 5 | 3 | 3 | 5 | | |
| **ST5-TTMV-5UTR-116nt** | 4 | 6 | 6 | 6 | 7 | |
| **ST4-TTMV-5UTR-116nt** | 5 | 3 | 7 | 8 | 4 | 7 |

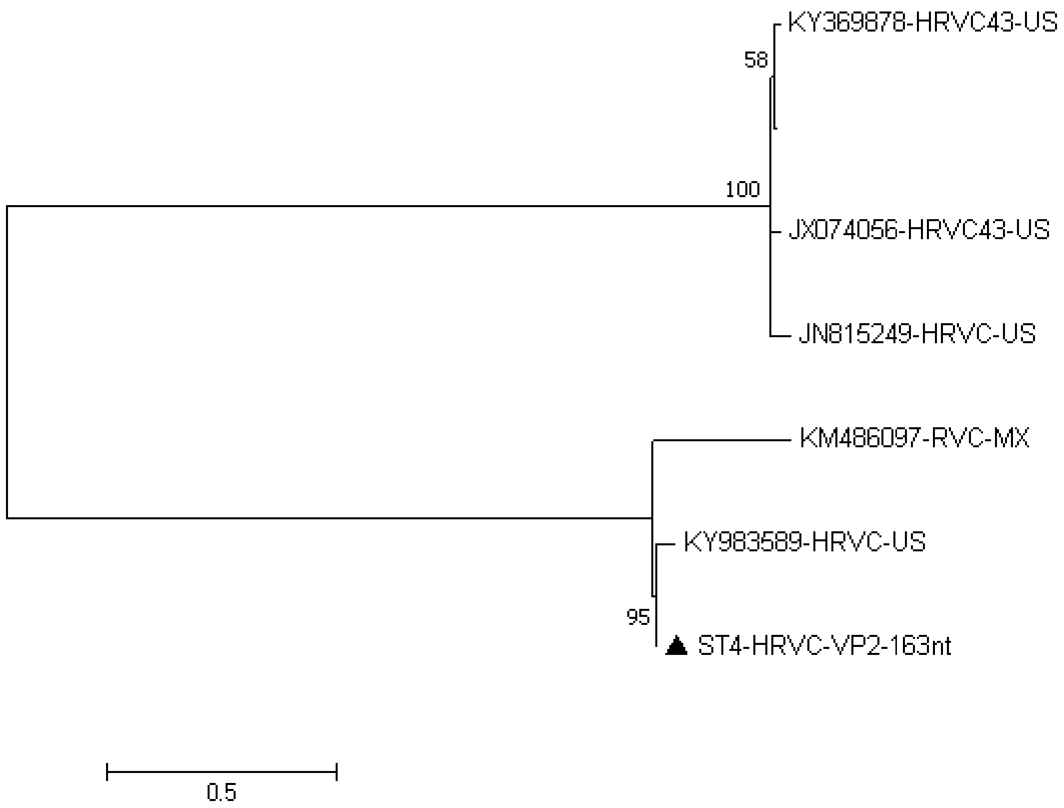


**Figure S11.3: Molecular Phylogenetic analysis by Maximum Likelihood method of HRVC partial VP2 gene**
The evolutionary history was inferred by using the Maximum Likelihood method based on the Kimura 2-parameter model[1]. The tree with the highest log likelihood (-537.01) is shown. The percentage of trees in which the associated taxa clustered together is shown next to the branches. Initial tree(s) for the heuristic search were obtained automatically by applying Neighbor-Join and BioNJ algorithms to a matrix of pairwise distances estimated using the Maximum Composite Likelihood (MCL) approach, and then selecting the topology with superior log likelihood value. The rate variation model allowed for some sites to be evolutionarily invariable ([+I], 66.13% sites). The tree is drawn to scale, with branch lengths measured in the number of substitutions per site. The analysis involved 7 nucleotide sequences. Codon positions included were 1st+2nd+3rd+Noncoding. All positions containing gaps and missing data were eliminated. There were a total of 163 positions in the final dataset. Evolutionary analyses were conducted in MEGA7[2].

**Table S11.3: Estimates of Evolutionary Divergence between Sequences**
The number of base differences per sequence from between sequences are shown. The analysis involved 7 nucleotide sequences. Codon positions included were 1st+2nd+3rd+Noncoding. All positions containing gaps and missing data were eliminated. There were a total of 163 positions in the final dataset. Evolutionary analyses were conducted in MEGA7.

| | | | | | | |
|---|---|---|---|---|---|---|
| KY983589-HRVC-US | | | | | | |
| KM486097-RVC-MX | 25 | | | | | |
| JN815249-HRVC-US | 34 | 39 | | | | |
| KY369878-HRVC43-US | 29 | 40 | 9 | | | |
| JX074056-HRVC43-US | 33 | 43 | 9 | 6 | | |
| LC004883-RVC-JP | 31 | 40 | 8 | 3 | 5 | |
| **ST4-HRVC-VP2-163nt** | 6 | 24 | 32 | 33 | 33 | 33 |

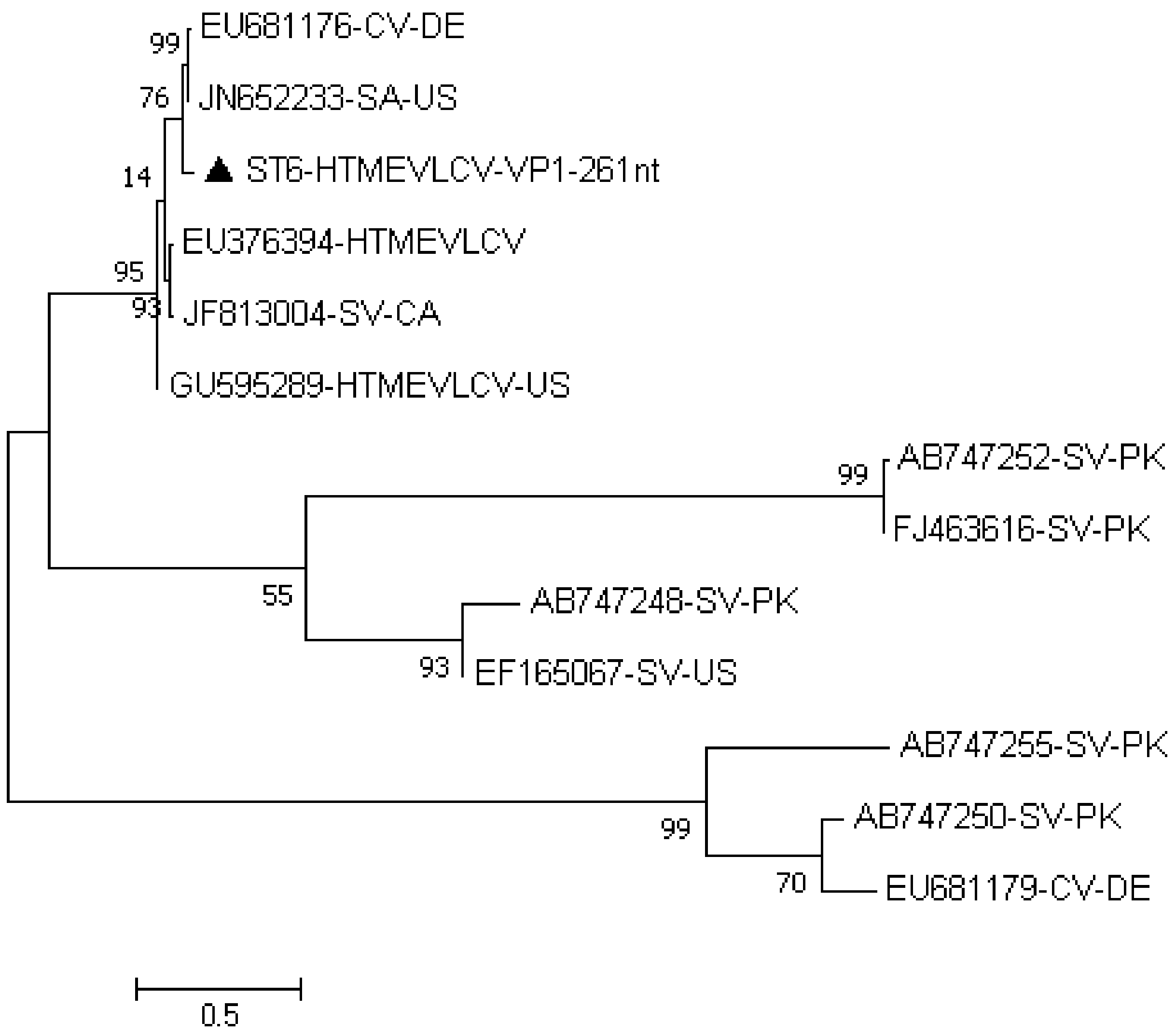


**Figure S11.4: Molecular Phylogenetic analysis by Maximum Likelihood method of HTMEVLCV partial VP1 gene**

The evolutionary history was inferred by using the Maximum Likelihood method based on the Tamura 3-parameter model[4]. The tree with the highest log likelihood (-1847.23) is shown. The percentage of trees in which the associated taxa clustered together is shown next to the branches. Initial tree(s) for the heuristic search were obtained automatically by applying Neighbor-Join and BioNJ algorithms to a matrix of pairwise distances estimated using the Maximum Composite Likelihood (MCL) approach, and then selecting the topology with superior log likelihood value. A discrete Gamma distribution was used to model evolutionary rate differences among sites (5 categories (+G, parameter = 1.1715)). The tree is drawn to scale, with branch lengths measured in the number of substitutions per site. The analysis involved 13 nucleotide sequences. Codon positions included were 1st+2nd+3rd+Noncoding. All positions containing gaps and missing data were eliminated. There were a total of 255 positions in the final dataset. Evolutionary analyses were conducted in MEGA7[2].

**Table S11.4: Estimates of Evolutionary Divergence between Sequences**

The number of base differences per sequence from between sequences are shown. The analysis involved 13 nucleotide sequences. Codon positions included were 1st+2nd+3rd+Noncoding. All positions containing gaps and missing data were eliminated. There were a total of 255 positions in the final dataset. Evolutionary analyses were conducted in MEGA7.

| | | | | | | | | | | | | |
|---|---|---|---|---|---|---|---|---|---|---|---|---|
| AB747252-SV-PK | | | | | | | | | | | | |
| AB747248-SV-PK | 107 | | | | | | | | | | | |
| FJ463616-SV-PK | 4 | 105 | | | | | | | | | | |
| AB747255-SV-PK | 115 | 125 | 115 | | | | | | | | | |
| AB747250-SV-PK | 112 | 121 | 115 | 77 | | | | | | | | |
| EF165067-SV-US | 103 | 34 | 101 | 127 | 121 | | | | | | | |
| GU595289-HTMEVLCV-US | 119 | 96 | 118 | 116 | 111 | 90 | | | | | | |
| EU681179-CV-DE | 107 | 115 | 108 | 80 | 42 | 113 | 113 | | | | | |
| EU376394-HTMEVLCV | 116 | 95 | 115 | 115 | 112 | 95 | 11 | 111 | | | | |
| EU681176-CV-DE | 114 | 99 | 113 | 115 | 114 | 93 | 22 | 114 | 21 | | | |
| JN652233-SA-US | 112 | 100 | 111 | 114 | 114 | 94 | 21 | 114 | 20 | 3 | | |
| JF813004-SV-CA | 116 | 95 | 115 | 115 | 113 | 95 | 12 | 112 | 3 | 22 | 21 | |
| **ST6-HTMEVLCV-VP1-261nt** | 111 | 97 | 110 | 111 | 111 | 91 | 23 | 112 | 20 | 11 | 10 | 23 |

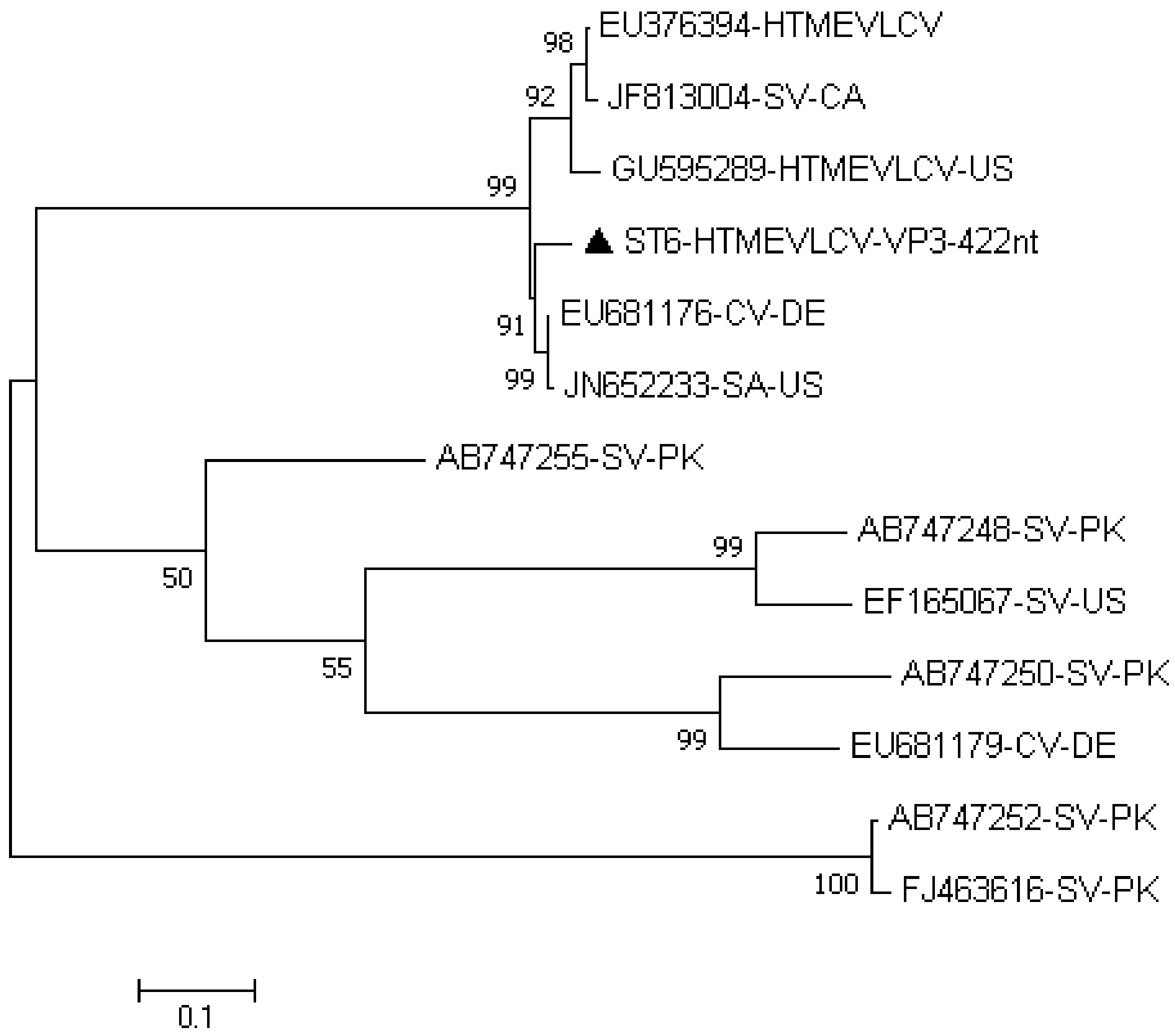


**Figure S11.5: Molecular Phylogenetic analysis by Maximum Likelihood method of HTMEVLCV partial VP3 gene**

The evolutionary history was inferred by using the Maximum Likelihood method based on the Hasegawa-Kishino-Yano model[5]. The tree with the highest log likelihood (-2386.84) is shown. The percentage of trees in which the associated taxa clustered together is shown next to the branches. Initial tree(s) for the heuristic search were obtained automatically by applying Neighbor-Join and BioNJ algorithms to a matrix of pairwise distances estimated using the Maximum Composite Likelihood (MCL) approach, and then selecting the topology with superior log likelihood value. A discrete Gamma distribution was used to model evolutionary rate differences among sites (5 categories (+G, parameter = 0.5836)). The tree is drawn to scale, with branch lengths measured in the number of substitutions per site. The analysis involved 13 nucleotide sequences. Codon positions included were 1st+2nd+3rd+Noncoding. All positions containing gaps and missing data were eliminated. There were a total of 422 positions in the final dataset. Evolutionary analyses were conducted in MEGA7[2].

**Table S11.5: Estimates of Evolutionary Divergence between Sequences**

The number of base differences per sequence from between sequences are shown. The analysis involved 13 nucleotide sequences. Codon positions included were 1st+2nd+3rd+Noncoding. All positions containing gaps and missing data were eliminated. There were a total of 422 positions in the final dataset. Evolutionary analyses were conducted in MEGA7.

| | | | | | | | | | | | | |
|---|---|---|---|---|---|---|---|---|---|---|---|---|
| AB747252-SV-PK | | | | | | | | | | | | |
| AB747248-SV-PK | 123 | | | | | | | | | | | |
| FJ463616-SV-PK | 9 | 124 | | | | | | | | | | |
| AB747255-SV-PK | 108 | 95 | 111 | | | | | | | | | |
| AB747250-SV-PK | 124 | 97 | 127 | 93 | | | | | | | | |
| EF165067-SV-US | 117 | 50 | 120 | 98 | 96 | | | | | | | |
| GU595289-HTMEVLCV-US | 123 | 110 | 127 | 100 | 114 | 110 | | | | | | |
| EU681179-CV-DE | 128 | 102 | 128 | 97 | 67 | 100 | 110 | | | | | |
| EU376394-HTMEVLCV | 119 | 106 | 123 | 101 | 106 | 108 | 17 | 111 | | | | |
| EU681176-CV-DE | 117 | 102 | 120 | 98 | 109 | 106 | 28 | 116 | 25 | | | |
| JN652233-SA-US | 118 | 101 | 121 | 97 | 111 | 104 | 30 | 118 | 27 | 2 | | |
| JF813004-SV-CA | 120 | 104 | 124 | 103 | 106 | 109 | 20 | 115 | 5 | 28 | 30 | |
| **ST6-HTMEVLCV-VP3-422nt** | 126 | 106 | 127 | 102 | 106 | 108 | 35 | 115 | 32 | 17 | 19 | 35 |

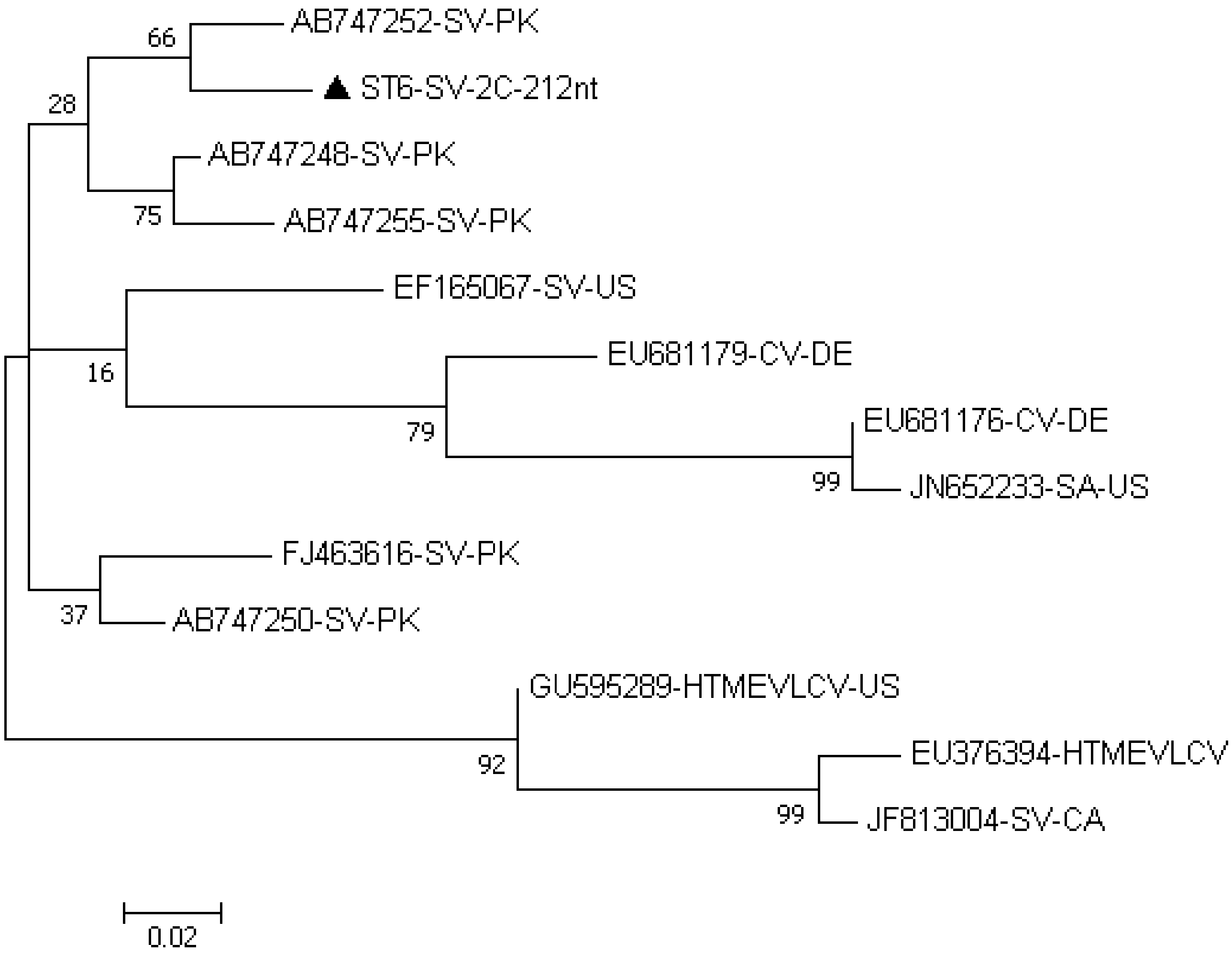


**Figure S11.6: Molecular Phylogenetic analysis by Maximum Likelihood method of SV partial 2C gene**

The evolutionary history was inferred by using the Maximum Likelihood method based on the Tamura 3-parameter model[4]. The tree with the highest log likelihood (-752.38) is shown. The percentage of trees in which the associated taxa clustered together is shown next to the branches. Initial tree(s) for the heuristic search were obtained automatically by applying Neighbor-Join and BioNJ algorithms to a matrix of pairwise distances estimated using the Maximum Composite Likelihood (MCL) approach, and then selecting the topology with superior log likelihood value. A discrete Gamma distribution was used to model evolutionary rate differences among sites (5 categories (+G, parameter = 0.2798)). The tree is drawn to scale, with branch lengths measured in the number of substitutions per site. The analysis involved 13 nucleotide sequences. Codon positions included were 1st+2nd+3rd+Noncoding. All positions containing gaps and missing data were eliminated. There were a total of 212 positions in the final dataset. Evolutionary analyses were conducted in MEGA7[2].

**Table S11.6: Estimates of Evolutionary Divergence between Sequences**

The number of base differences per sequence from between sequences are shown. The analysis involved 13 nucleotide sequences. Codon positions included were 1st+2nd+3rd+Noncoding. All positions containing gaps and missing data were eliminated. There were a total of 212 positions in the final dataset. Evolutionary analyses were conducted in MEGA7.

| | | | | | | | | | | | | |
|---|---|---|---|---|---|---|---|---|---|---|---|---|
| AB747252-SV-PK | | | | | | | | | | | | |
| AB747248-SV-PK | 11 | | | | | | | | | | | |
| FJ463616-SV-PK | 13 | 14 | | | | | | | | | | |
| AB747255-SV-PK | 14 | 5 | 17 | | | | | | | | | |
| AB747250-SV-PK | 14 | 9 | 9 | 10 | | | | | | | | |
| EF165067-SV-US | 14 | 16 | 17 | 16 | 16 | | | | | | | |
| GU595289-HTMEVLCV-US | 19 | 20 | 22 | 22 | 21 | 19 | | | | | | |
| EU681179-CV-DE | 24 | 20 | 21 | 17 | 14 | 21 | 25 | | | | | |
| EU376394-HTMEVLCV | 26 | 27 | 29 | 27 | 28 | 26 | 13 | 29 | | | | |
| EU681176-CV-DE | 27 | 25 | 26 | 22 | 21 | 24 | 23 | 17 | 24 | | | |
| JN652233-SA-US | 28 | 26 | 27 | 23 | 22 | 24 | 24 | 19 | 25 | 2 | | |
| JF813004-SV-CA | 25 | 26 | 26 | 26 | 27 | 25 | 12 | 31 | 5 | 25 | 26 | |
| **ST6-SV-2C-212nt** | 8 | 11 | 13 | 14 | 16 | 18 | 21 | 26 | 30 | 27 | 28 | 29 |

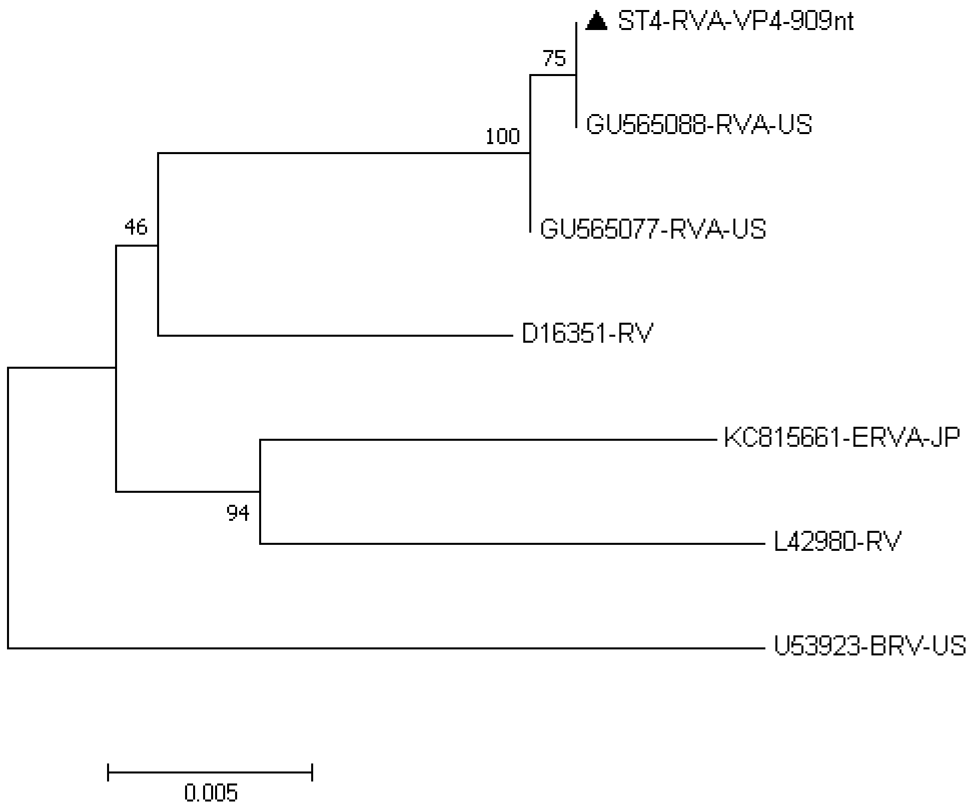


**Figure S11.7: Molecular Phylogenetic analysis by Maximum Likelihood method of RVA partial VP4 gene**
The evolutionary history was inferred by using the Maximum Likelihood method based on the Hasegawa-Kishino-Yano model[5]. The tree with the highest log likelihood (-1615.82) is shown. The percentage of trees in which the associated taxa clustered together is shown next to the branches. Initial tree(s) for the heuristic search were obtained automatically by applying Neighbor-Join and BioNJ algorithms to a matrix of pairwise distances estimated using the Maximum Composite Likelihood (MCL) approach, and then selecting the topology with superior log likelihood value. The tree is drawn to scale, with branch lengths measured in the number of substitutions per site. The analysis involved 7 nucleotide sequences. Codon positions included were 1st+2nd+3rd+Noncoding. All positions containing gaps and missing data were eliminated. There were a total of 909 positions in the final dataset. Evolutionary analyses were conducted in MEGA7[2].

**Table S11.7: Estimates of Evolutionary Divergence between Sequences**
The number of base differences per sequence from between sequences are shown. The analysis involved 7 nucleotide sequences. Codon positions included were 1st+2nd+3rd+Noncoding. All positions containing gaps and missing data were eliminated. There were a total of 909 positions in the final dataset. Evolutionary analyses were conducted in MEGA7.

| **ST4-RVA-VP4-909nt** | | | | | | |
|---|---|---|---|---|---|---|
| GU565088-RVA-US | 0 | | | | | |
| GU565077-RVA-US | 1 | 1 | | | | |
| D16351-RV | 17 | 17 | 16 | | | |
| KC815661-ERVA-JP | 22 | 22 | 21 | 22 | | |
| L42980-RV | 23 | 23 | 22 | 23 | 21 | |
| U53923-BRV-US | 29 | 29 | 28 | 26 | 32 | 33 |

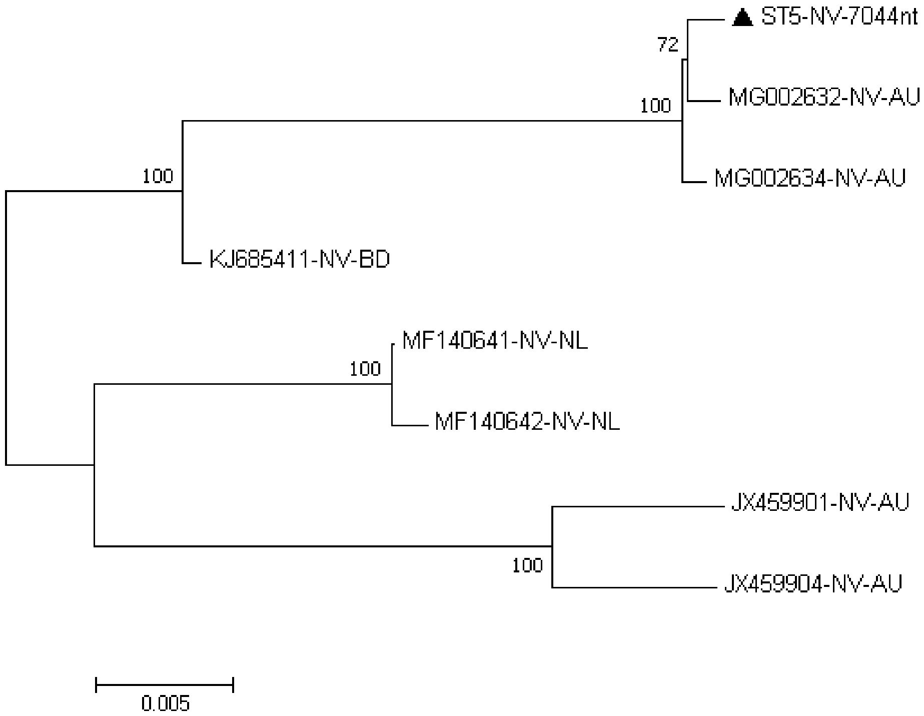


**Figure S11.8: Molecular Phylogenetic analysis by Maximum Likelihood method of NV**

The evolutionary history was inferred by using the Maximum Likelihood method based on the Tamura-Nei model[3]. The tree with the highest log likelihood (-12582.19) is shown. The percentage of trees in which the associated taxa clustered together is shown next to the branches. Initial tree(s) for the heuristic search were obtained automatically by applying Neighbor-Join and BioNJ algorithms to a matrix of pairwise distances estimated using the Maximum Composite Likelihood (MCL) approach, and then selecting the topology with superior log likelihood value. A discrete Gamma distribution was used to model evolutionary rate differences among sites (5 categories (+G, parameter = 0.1000)). The tree is drawn to scale, with branch lengths measured in the number of substitutions per site. The analysis involved 8 nucleotide sequences. Codon positions included were 1st+2nd+3rd+Noncoding. All positions containing gaps and missing data were eliminated. There were a total of 7044 positions in the final dataset. Evolutionary analyses were conducted in MEGA7[2].

**Table S11.8: Estimates of Evolutionary Divergence between Sequences**

The number of base differences per sequence from between sequences are shown. The analysis involved 8 nucleotide sequences. Codon positions included were 1st+2nd+3rd+Noncoding. All positions containing gaps and missing data were eliminated. There were a total of 7044 positions in the final dataset. Evolutionary analyses were conducted in MEGA7.

| **ST5-NV-7044nt** | | | | | | | |
|---|---|---|---|---|---|---|---|
| MG002634-NV-AU | 17 | | | | | | |
| MG002632-NV-AU | 17 | 16 | | | | | |
| KJ685411-NV-BD | 132 | 129 | 131 | | | | |
| MF140641-NV-NL | 244 | 239 | 241 | 139 | | | |
| MF140642-NV-NL | 250 | 245 | 247 | 147 | 10 | | |
| JX459901-NV-AU | 298 | 295 | 297 | 207 | 212 | 218 | |
| JX459904-NV-AU | 301 | 298 | 300 | 207 | 208 | 214 | 82 |

References

1. Kimura, M. A simple method for estimating evolutionary rates of base substitutions through comparative studies of nucleotide sequences. *J. Mol. Evol.* **16,** 111–20 (1980).

2. Kumar, S., Stecher, G. & Tamura, K. MEGA7: Molecular Evolutionary Genetics Analysis Version 7.0 for Bigger Datasets. *Mol. Biol. Evol.* **33,** 1870–4 (2016).

3. Tamura, K. & Nei, M. Estimation of the number of nucleotide substitutions in the control region of mitochondrial DNA in humans and chimpanzees. *Mol. Biol. Evol.* **10,** 512–26 (1993).

4. Tamura, K. Estimation of the number of nucleotide substitutions when there are strong transition-transversion and G+C-content biases. *Mol. Biol. Evol.* **9,** 678–87 (1992).

5. Hasegawa, M., Kishino, H. & Yano, T. Dating of the human-ape splitting by a molecular clock of mitochondrial DNA. *J. Mol. Evol.* **22,** 160–74 (1985).

6. Le, S. Q. & Gascuel, O. An Improved General Amino Acid Replacement Matrix. *Mol. Biol. Evol.* **25,** 1307–1320 (2008).

7. Nei, M. & Kumar, S. *Molecular evolution and phylogenetics*. (Oxford University Press, 2000).

8. Whelan, S. & Goldman, N. A general empirical model of protein evolution derived from multiple protein families using a maximum-likelihood approach. *Mol. Biol. Evol.* **18,** 691–9 (2001).

9. Jukes, T. H. & Cantor, C. in *Mammalian Protein Metabolism* (ed. Munro, H.) 21–132 (Academic Press, New York., 1969).

# Supplementary Material 3

## Metagenomics detection and characterisation of viruses in faecal samples from Australian wild birds

**Running Title of the Supplementary Material**

Detailed optimised protocol for metagenomics of viruses and microbiome analysis.

**Authors**

Jessy Vibin[1,2], Anthony Chamings[1,2], Fiona Collier[1,3], Marcel Klaassen[2], Tiffanie M Nelson[1,2], Soren Alexandersen*[1,2,3]

1 Geelong Centre for Emerging Infectious Diseases, Geelong, VIC 3220, Australia;

2 Deakin University, Geelong, Victoria, 3220, Australia;

3 Barwon Health, Geelong, VIC 3220, Australia;

*Corresponding Author: soren.alexandersen@deakin.edu.au

**Materials required**:

- 1X PBS
- 2mL microtubes with O-ring
- Microtubes, PCR tubes, 96 well plates
- pipettes and tips
- 0.8µm polyethersulfone (PES) spin filter (Sartorius, Catalogue number: VK01P042)
- Benzonase Nuclease, Purity > 90% (Millipore, Catalogue Number: 707463)
- Micrococcal nuclease (New England Biolabs, Catalogue Number: M0247S)
- 20X buffer {1M Tris pH8, 100mM calcium chloride (CaCl), 30mM magnesium chloride ($MgCl_2$)}
- 500mM ethylenediaminetetraacetic acid (EDTA; pH=8)
- QIAmp viral RNA mini kit (Qiagen; Catalogue number: 52904)
- 100% ethanol
- SeqPlex RNA amplification kit (Sigma, Catalogue number: SEQR)
- GenElute PCR Clean-up Kit (Sigma, Catalogue number: NA1020)
- Agilent High Sensitivity DNA Kit (Agilent, Catalogue number: 5067-4626)
- Ion Plus Fragment Library Kit (Thermo Fisher Scientific; Catalogue number: 4471252)
- Agencourt AMPure XP Kit (Beckman Coulter, Catalogue number: A63880)
- Magnet
- Low TE
- Ion Xpress Barcode Adapters 1-16 Kit (Thermo Fisher Scientific; Catalogue number: 4471250)
- Ion Library TaqMan™ Quantitation Kit (Thermo Fisher Scientific; Catalogue number: 4468802)
- Ion 520 & Ion 530 Kit-Chef (Thermo Fisher Scientific; Catalogue number: A27757)

**Instruments required**:

- small-scale analytical balance
- vortex
- TissueLyser II homogeniser (Qiagen) or equivalent homogeniser
- Microcentrifuge
- Beckman Coulter Airfuge Ultracentrifuge or equivalent ultracentrifuge
- Dry bath or water bath
- Nanodrop spectrophotometer
- Bioanalyzer
- Thermocycler
- QuantStudio™ 6 Flex Real-Time PCR System (Thermo Fisher Scientific; Catalogue number: 4485697) or equivalent real-time PCR instrument.
- Ion S5, Ion Chef Instrument (Thermo Fisher Scientific; Catalogue number: 4484177)
- Ion Torrent Sequencing and genetic analysis software (Thermo Fisher Scientific)

**Step 1: Sample preparation**

- Make 1:10 dilution of the faecal sample using sterile 1X PBS, i.e. 100mg:1ml

**Step 2: Sample homogenization**

- Transfer 1ml * 2 to two 2ml O ring tubes.
- Homogenize for 25Hz for 2min.
- Mix both tubes.
- Transfer 250µL to one tube. Now Tube A (1750µL) and Tube B (250µL).

**Step 3: Centrifugation**

- Centrifuge both tubes for 17000g for 3min at room temperature (RT).
- Collect supernatant.

**Step 4: Filtration**

- Filter both tubes using 0.8µm PES filter at 17000g for 1min or until all of the sample has passed through.
- Collect filtrate. Put Tube B on ice. Proceed Tube A for ultracentrifugation.

**Step 5: Ultracentrifugation**

- The sample was transferred into four 8*20mm ultra-clear (max of 450µL and min of 300µL) straight wall tubes.
- The tubes were placed in the A-95 rotor and balanced.
- The sample was then ultracentrifuged at 178,000g for 1 hour (30psi for 1hour) at RT.
- The supernatant was discarded. The pellet was suspended in a total of 130µL of sterile 1XPBS.

**Step 6: Nuclease treatment**

- To 130µL of the samples (both Tube A and B separately), 7µL of 20X buffer, 2µL of benzonase nuclease and 1µL of micrococcal nuclease was added.
- After mixing gently, the samples were incubated at 37 °C for 2 h.
- The nuclease reaction was stopped by adding 3µL of 500mM EDTA.

**Step 7: Nucleic acid extraction**

Kit used: QIAmp viral RNA mini kit

Note: No carrier RNA was added to the AVL buffer. If the kit is new, add 25ml of absolute ethanol to buffer AW1 and 30ml of absolute ethanol to buffer AW2. All steps performed at RT. All centrifugation performed at RT.

- Take 560µL of buffer AVL in the 1.5ml tube.
- Add 140µL of the sample to the buffer and mix by vortexing for 15s.
- Incubate at RT for 10min.
- Spin down.
- Add 560µL of absolute ethanol to sample and mix by vortexing for 15s. Spin down.
- Apply 630µL to QIAmp Minicolumn.
- Centrifuge 6000g or 8000rpm for 1min.
- Repeat the above two steps.
- Place column in clean collection tube.
- Add 500µL of buffer AW1.
- Centrifuge 6000g or 8000rpm for 1min. Place column in clean collection tube.

- Add 500μL of buffer AW2.
- Centrifuge 20000g or 14000rpm for 1min. Place column in clean collection tube.
- Centrifuge at full speed for 1min. Place column in the clean 1.5ml tube.
- Add 40μL of buffer AVE directly to spin column membrane without touching it. Close the cap and incubate for 1min at RT.
- Centrifuge 6000g or 8000rpm for 1min.
- Take the eluted buffer and place it on spin column membrane (double elution).
- Centrifuge 6000g or 8000rpm for 1min. The filtrate contains the virus nucleic acid.
- Store isolated nucleic acid at -20 °C.

PAUSE POINT

**Step 8: cDNA synthesis, amplification and purification**
Kits used: SeqPlex RNA amplification kit and GenElute PCR Clean-up Kit

- Take 10μL of isolated nucleic acid and incubate for 95 °C for 3min followed by snap cooling in -20 °C cold ethanol.
- Add 2μL of Library Synthesis Solution and 1.2μL of nuclease free water to the nucleic acid making it a total volume of 13.2μL.
- Incubate in a thermocycler for 70 °C for 5min, 18 °C hold. Do not hold for more than 10 min. Remove reaction from thermocycler and place at RT or maintain at 18 °C.
- Add 2μL of Library synthesis buffer, 3.2μL of nuclease free water and 1.6μL of Library synthesis enzyme to the above reaction mixture to make it a total volume of 20μL.
- Incubate in a thermocycler for 18 °C for 10min, 25 °C for 10min, 37 °C for 30min, 42 °C for 10min, 70 °C for 20min and 4 °C hold.
- Preferably proceed further or store at -20 °C.
- To the above mixture add, 39.25μL of nuclease free water, 15μL of 5X amplification mix, 0.75μL of amplification enzyme making it a total volume of 75μL.
- Incubate in a thermocycler for 94 °C for 2min, 25 cycles of 94 °C for 30s and 70 °C for 5min, 1 cycle of 70 °C for 30min followed by 4 °C hold.
- Purify the reaction mixture with GenElute PCR Clean-up Kit.
- After purification quantify the DNA.
- Proceed further or store at -20 °C.

PAUSE POINT

**Step 9: Primer removal and purification**
Kits used: SeqPlex RNA amplification kit, GenElute PCR Clean-up Kit and Agilent High Sensitivity DNA Kit

- Take 2μg of purified DNA from the above and add 7.5μL of 10X primer removal buffer, 1.5μL of primer removal solution, 3.75μL of primer removal enzyme and make the total reaction volume to 75μL using nuclease free water.
- Incubate the reaction in 37 °C for 60min, 65 °C for 20min and 4 °C hold.
- Purify the reaction mixture with GenElute PCR Clean-up Kit.
- After purification quantify the DNA using Agilent High Sensitivity DNA Kit in Bioanalyzer.
- Proceed further or store at -20 °C.

**Step 10: End repair and purification**

Kits used: Ion Plus Fragment Library Kit and Agencourt AMPure XP Kit.

- Take 20µL of primer removed and purified sample DNA in a 1.5mL microtube and add nuclease free water to bring the volume to 79µL.
- Add 20µL of 5X end repair buffer and 1µL of end repair enzyme to make the total reaction mix to 100µL.
- Incubate the mix at RT for 20min.
- After the incubation purify the samples with Agencourt AMPure XP Kit with 180µL of AMPure XP Reagent. After the purification elute the sample in 25µL of Low TE buffer.
- Proceed further or store at -20 °C.

PAUSE POINT

**Step 11: Adapter ligation and Nick repair**

Kits used: Ion Plus Fragment Library Kit, Ion Xpress Barcode Adapters 1-16 Kit and Agencourt AMPure XP Kit.

- To the 25µL of eluted DNA samples add 10µL of 10X ligase buffer, 2µL of Ion P1 Adapter, 2µL of Ion Xpress Barcode X (barcode chosen), 2µL of dNTP mix, 49µL of nuclease free water, 2µL of DNA Ligase and 8µL of Nick Repair Polymerase.
- Incubate the reaction mix at 25 °C for 15min, 72 °C for 5min and 4 °C for up to 1h. Last stage is not a stopping point; continue directly to purification.
- Purify the samples using Agencourt AMPure XP Kit with 150µL of AMPure XP Reagent. After the purification elute the sample in 20µL of Low TE buffer.
- Store the DNA at -30 °C to -10 °C.

**Step 12: Quantify the unamplified library**

Kit used: Ion Library TaqMan™ Quantitation Kit

- Prepare 100-fold and 10000-fold dilution of the library using nuclease free water.
- Prepare 10-fold serial dilution of the *E.coli* DH10B Ion Control Library from 6.8pM to 0.0000068pM.
- To 9µL of the diluted library and control add 10µL of 2X TaqMan Master mix and 1µL of 20X Ion TaqMan Assay.
- Run RT-PCR in QuantStudio™ 6 Flex Real-Time PCR System using the following conditions in standard mode: 50 °C for 2min, 95 °C for 2min, 40 cycles of 95 °C for 15s and 60 °C for 1min followed by 10 °C hold.
- Calculate the concentration of undiluted library and determine the dilution that result in concentration of ~100pM.

PAUSE POINT

**Step 13: Next generation sequencing**

Kit used: Ion 520 & Ion 530 Kit-Chef

- Set up plan run in Ion Torrent Server as per manufacturer instructions.
- Prepare the Ion chip using Ion Chef as per manufacturer instructions.
- Run next generation sequencer Ion S5 as per manufacturer instructions.

**Step 14: Next generation sequencing analysis**

- BLASTN, BLASTX, MEGABLAST, Ion Torrent Server, TMAP, IGV for virus analysis
- Ion Reporter for microbiome analysis.

**Step 15: Partial genome and evolutionary analysis of viruses**

- IGV, BLASTN, BLASTX, MEGA for partial genome and evolutionary analysis.
